# UK Kidney Association Clinical Practice Guideline: Sodium-Glucose Co-transporter-2 (SGLT-2) Inhibition in Adults with Kidney Disease 2023 UPDATE

**DOI:** 10.1186/s12882-023-03339-3

**Published:** 2023-10-25

**Authors:** Alistair J. Roddick, Alexa Wonnacott, David Webb, Angela Watt, Michael A. Watson, Natalie Staplin, Alex Riding, Eirini Lioudaki, Apexa Kuverji, Mohsen El Kossi, Patrick Holmes, Matt Holloway, Donald Fraser, Chris Carvalho, James O. Burton, Sunil Bhandari, William G. Herrington, Andrew H. Frankel

**Affiliations:** 1 The Medical Research Council Population Health Research Unit at the University of Oxford, Oxford, UK; 2 Oxford Kidney Unit, Oxford University Hospitals NHS Foundation Trust, Oxford, UK; 3 Wales Kidney Research Unit, Cardiff University, Cardiff, UK; 4 Diabetes Research Centre, College of Life Sciences, University of Leicester, Leicester, UK; 5 UKKA Patient Representative, Bristol, UK; 6 Royal Free London NHS Foundation Trust, London, UK; 7 Kings College Hospital NHS Trust, London, UK; 8 John Walls Renal Unit, Glenfield Hospital, Leicester, UK; 9 Doncaster Royal Infirmary, Doncaster, UK; 10 Primary Care Diabetes Society, Darlington, UK; 11 East Kent Hospitals University NHS Foundation Trust, Canterbury, UK; 12 North East London Integrated Care Board, London, UK; 13 Clinical Effectiveness Group, Queen Mary University of London, London, UK; 14 Department of Cardiovascular Sciences, University of Leicester, Leicester, UK; 15 Hull University Teaching Hospitals NHS Trust and Hull York Medical School, Hull, UK; 16 Imperial College Healthcare NHS Trust, London, UK

**Keywords:** Guideline, Chronic kidney disease, Acute kidney injury, Gliflozin

## Abstract

Large placebo-controlled trials have demonstrated kidney and cardiovascular clinical benefits of SGLT-2 inhibitors. Data from the EMPA-KIDNEY and DELIVER trials and associated meta-analyses triggered an update to the UK Kidney Association Clinical Practice Guideline on Sodium-Glucose Co-transporter-2 (SGLT-2) Inhibition in Adults with Kidney Disease. We provide a summary of the full guideline and highlight the rationale for recent updates. The use of SGLT-2 inhibitors in people with specific medical conditions, including type 1 diabetes, kidney transplants, and people admitted to hospital with heart failure is also considered, along with Recommendations for future research and Recommendations for implementation. A full “lay” summary of the guidelines is provided as an appendix to ensure that these guidelines are accessible and understandable to people who are not medical professionals.

**Supplementary Information:**

The online version contains supplementary material available at 10.1186/s12882-023-03339-3.

## Introduction

Sodium-glucose co-transporter-2 (SGLT-2) inhibitors represent a major step in the management of chronic kidney disease (CKD), with evidence from several large randomised clinical trials and collaborative meta-analyses indicating that this medication class reduces progression of kidney disease and kidney failure in a broad range of people with CKD irrespective of diabetes (DM) status, level of kidney function, or primary kidney diagnosis [1–4]. Furthermore, SGLT-2 inhibition has been shown to improve outcomes in people with diabetes with high cardiovascular risk, and in people with heart failure across the spectrum of left ventricular ejection fraction [4–6].

**Fig. 1:**
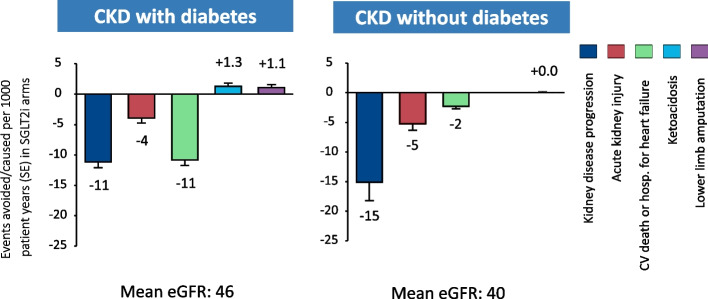
Absolute benefit and risks of SGLT-2 inhibition for people with CKD with and without diabetes, estimated from 13 large randomised clinical trials of SGLT-2 inhibition (adapted from [4]). CKD – chronic kidney disease. eGFR – estimated glomerular filtration rate. SE – standard error. SGLT-2i – sodium-glucose co-transporter 2 inhibitor. Figure licensed under Creative Commons CC-BY license

In addition to well-established efficacy, SGLT-2 inhibition has been shown to be safe, with few associated risks, which include mycotic infections, lower limb amputations and ketoacidosis. Importantly, serious side effects are rare, particularly among people without diabetes [4]. A recent meta-analysis assessing the balance of risk and benefit in CKD indicates that the absolute benefits of SGLT-2 inhibition in terms of kidney disease progression, cardiovascular death or hospitalisation for heart failure, and acute kidney injury substantially outweigh any associated risks in the studied populations (Fig. 1) [4].

Given these findings, there is a need to provide practical and pragmatic guidance for the use of this class of medication to facilitate rapid, effective and safe implementation in clinical practice. In 2021, the UK Kidney Association (UKKA) Clinical Practice Guideline: SGLT-2 Inhibition in Adults with Kidney Disease Working Group published a Clinical Practice Guideline on the use of SGLT-2 inhibitors in adults with kidney disease in order to facilitate this, with emphasis on relevant large-scale randomised evidence for the efficacy and safety of SGLT-2 inhibition. This 2023 guideline presents updated Recommendations in light of new evidence from the recent DELIVER and EMPA-KIDNEY trials [3, 7]. This guideline’s aims were to: (i) provide guidance on use of SGLT-2 inhibitors in people with CKD, focusing on the potential to modify risk of kidney disease progression; and (ii) support safe implementation of SGLT-2 inhibitors into clinical practice in people with CKD.

## Guideline structure

This manuscript provides an overview of the Recommendations made within the guideline with associated rationale. More information regarding the evidence for the efficacy and safety of SGLT-2 inhibition can be found in the full guideline document, together with licencing information, sick day guidance, and a full lay summary of the guideline. The full guideline document is provided as a Supplementary appendix. The guideline Working Group provided four types of Recommendations: (i) Use (who should be offered SGLT-2 inhibition); (ii) Implementation (how should SGLT-2 inhibition be used); (iii) Research (what are areas of ongoing clinical uncertainty); and (iv) Audit (how effective implementation can be demonstrated).

The Recommendations for Use and Recommendations for Implementation presented in this guideline are graded according to the two-tier grading system recommended by the UKKA (Table 1). Throughout the guideline, we use the term “recommend” where Recommendations are based on Grade 1 evidence, and “suggest” for those based on Grade 2 evidence. Recommendations for research are not graded, and we offer Recommendations for audit only for those Recommendations with Grade 1 evidence.

**Table 1:** UK Kidney Association’s grading system for Recommendations’ strength and evidence quality

Level of evidence	Evidence quality
• Grade 1 Recommendation is a strong recommendation to do (or not do) something, where the benefits clearly outweigh the risks (or vice versa) for most, if not all patients (i.e. recommendations) • Grade 2 Recommendation is a weaker recommendation, where the risks and benefits are more closely balanced or are more uncertain (i.e. suggestions)	• Grade A evidence means high-quality evidence that comes from consistent results from well-performed randomised controlled trials, or overwhelming evidence of some other sort • Grade B evidence means moderate-quality evidence from randomised trials that suffer from serious flaws in conduct, inconsistency, indirectness, imprecise estimates, reporting bias, or some combination of these limitations, or from other study designs with special strength • Grade C evidence means low-quality evidence from observational studies, or from controlled trials with several very serious limitations • Grade D evidence is based only on case studies or expert opinion

In this manuscript, we provide Recommendations for Use of SGLT-2 inhibition in people with and without diabetes separately, to acknowledge differences in the amount of available evidence (Table 2). We also provide Recommendations for implementation of SGLT-2 inhibition in practice, with a focus on safety considerations (Table 3). Finally, we discuss the use and implementation of SGLT-2 inhibition in populations of specific interest: people with type 1 diabetes, kidney transplant recipients, and people presenting with acute decompensated heart failure (Table 3). Research Recommendations are detailed in Table 4, and audit Recommendations listed in Table 5. Further information on the use of SGLT-2 inhibition, including a summary of UK licencing and a full lay summary of the guideline, can be found in the full document.

**Table 2:** Summary of Recommendations for Use

RECOMMENDATIONS FOR USE		
	**PEOPLE WITH TYPE 2 DM**	**Grade**
**1**	We recommend initiating SGLT-2 inhibition in people with chronic kidney disease and type 2 diabetes, irrespective of primary kidney disease,^a^ for any of the following 4 clinical scenarios: a) eGFR of 20–45 mL/min/1.73m^2^ b) eGFR of > 45 mL/min/1.73m^2^ and a urinary albumin-to-creatinine ratio (uACR) of ≥ 25 mg/mmol^b^ c) Symptomatic heart failure, irrespective of ejection fraction d) Established coronary disease	**1A**
**2**	We suggest initiating SGLT-2 inhibition to modify cardiovascular risk and slow rate of kidney function decline in people with an eGFR > 45–60 mL/min/1.73m^2^ and a uACR of < 25 mg/mmol, recognising effects on glycaemic control will be limited	**2B**
**3**	We suggest clinicians consider initiating SGLT-2 inhibition in people with an eGFR below 20 mL/min/1.73m^2^ to slow progression of kidney disease	**2B**
	**PEOPLE WITHOUT DM**	
**1**	We recommend initiating SGLT-2 inhibition in people with chronic kidney disease, irrespective of primary kidney disease,^a^ for any of the following clinical scenarios: (a) eGFR of ≥ 20 mL/min/1.73m^2^ and a urinary albumin-to-creatinine ratio (uACR) of ≥ 25 mg/mmol^b^ (b) Symptomatic heart failure, irrespective of ejection fraction	**1A**
**2**	We recommend initiating SGLT-2 inhibition to slow rate of kidney function decline in people with an eGFR of 20–45 mL/min/1.73m^2^ and a uACR of < 25 mg/mmol^b^	**1B**
**3**	We suggest clinicians consider initiating SGLT-2 inhibition in people with an eGFR below 20 mL/min/1.73m^2^ to slow progression of kidney disease	**2B**

^a^excludes people with polycystic kidney disease, type 1 diabetes, or a kidney transplant

^b^urinary protein-to-creatinine ratio of 35 mg/mmol can be considered equivalent

**Table 3:** Summary of Recommendations for Implementation

RECOMMENDATIONS FOR IMPLEMENTATION		
	**PEOPLE WITH OR WITHOUT DM (excluding TYPE 1)**	**Grade**
**1**	We recommend using SGLT-2 inhibitors with demonstrated efficacy for their given indications	**1A**
**2**	We recommend using clinically appropriate single agent renin-angiotensin-system (RAS) blockade in combination with SGLT-2 inhibition, wherever RAS blockade is indicated and tolerated	**1A**
**3**	We suggest following NICE guidelines on use of uACR for screening of albuminuria (NICE NG203). We recognise that more pragmatic approaches to identifying risk of kidney disease progression may be necessary whilst local access to uACR measurement is improved	**2C**
**4**	We recommend that SGLT-2 inhibition can be continued until the need for dialysis or kidney transplantation arises	**1A**
**5**	We suggest that co-prescription of SGLT-2 inhibition with mineralocorticoid receptor antagonists (MRA) can be considered, where each are individually indicated	**2B**
**6**	We suggest the beneficial effects of SGLT-2 inhibition on kidney disease progression or risk of heart failure hospitalisation are likely to be a class effect	**2B**
	**DIABETIC KETOACIDOSIS**	
**1**	We recommend that people with type 1 DM should only have SGLT-2 inhibitors initiated under the strict direction of the diabetes team	**1C**
**2**	We recommend that people with type 2 DM at greater risk of diabetic ketoacidosis (DKA; defined in Table 5a.1 of the supplementary appendix) should have SGLT-2 inhibitors initiated with caution after discussion with the diabetes team	**1C**
**3**	We recommend SGLT-2 inhibitors are discontinued when an individual develops DKA	**1A**
**4**	We suggest that after an episode of DKA and where a clear contributing factor has been identified, there should be discussion with the person and clinical team to establish whether the benefits of re-introducing an SGLT-2 inhibitor outweigh the risks	**2D**
**5**	When initiating SGLT-2 inhibitors, we suggest that individuals should be advised on the signs and symptoms of DKA and be instructed to temporarily withhold SGLT-2 inhibitors and to seek immediate medical advice if symptoms develop	**1C**
**6**	We recommend always offering advice on sick day guidance when initiating SGLT-2 inhibitors and reminding them of this at every medication review	**1C**
**7**	We suggest that individuals taking SGLT-2 inhibitors should be advised against following a ketogenic diet	**2C**
**8**	We suggest that for people who choose to intermittently fast (e.g. for Ramadan), and particularly for those who are elderly, on diuretics or have CKD, consider withholding SGLT-2 inhibitors for the duration of the fasting period and for those people with diabetes ketone testing should be undertaken if unwell	**2D**
	**HYPOGLYCAEMIA**	
**1**	We recommend considering reducing the dose of insulin/sulphonylureas/meglitinides when initiating SGLT-2 inhibitors to reduce the risk of hypoglycaemia	**1C**
**2**	We recommend that when initiating SGLT-2 inhibitors in people taking SUs (e.g. gliclazide) or meglitinides (e.g. repaglinide) when the HbA1c < 58 mmol/mol AND eGFR > 45 mL/min/1.73m^2^, consider reducing dose of SU or meglitinide by 50% to reduce risk of hypoglycaemia	**1C**
**3**	We recommend that when starting SGLT-2 inhibitors in people taking insulin when the HbA1c < 58 mmol/mol AND eGFR > 45 mL/min/1.73m^2^, consider reducing the insulin dose by 20% to avoid hypoglycaemia	**1C**
**4**	We recommend that when starting SGLT-2 inhibitors in people taking only metformin ± pioglitazone ± DPP-4i/gliptins or GLP-1 receptor agonist therapy, no dosage adjustment is necessary	**1C**
	**ACUTE KIDNEY INJURY, HYPOVOLAEMIA AND POTASSIUM**	
**1**	We recommend that individuals initiated on an SGLT-2 inhibitor do not routinely require an early assessment of kidney function or serum potassium following initiation of treatment	**1C**
**2**	We suggest that if an individual has a kidney function assessment within the first few weeks post initiation of an SGLT-2 inhibitor, a decline in eGFR needs to be interpreted with caution and in the context of an expected drug effect to avoid unwarranted discontinuation of treatment	**2B**
**3**	We suggest that individuals on diuretics are counselled on the symptoms of hypovolaemia and advised to seek medical attention if they develop any such symptoms after starting SGLT-2 inhibition	**2B**
**4**	We suggest that clinicians consider an early clinical review and if appropriate a diuretic or antihypertensive dose reduction in individuals they consider at high risk of hypovolaemia	**2C**
**5**	We recommend that SGLT-2 inhibitors are temporarily withheld during acute illness (see sick-day guidance in section 5a.1.2 of the supplementary appendix)	**1C**
	**PERIPHERAL VASCULAR DISEASE AND AMPUTATION RISK**	
**1**	We suggest avoiding initiation of SGLT-2 inhibitors in the presence of active foot disease (infection, ulceration and ischaemia) and withholding treatment in those who develop foot complications whilst taking an SGLT-2 inhibitor	**2B**
**2**	We suggest a shared decision-making approach, with appropriate counselling on risks and benefits of treatment and the importance of routine preventative foot care measures for: • Individuals at high risk of amputation (previous amputations, existing PVD, peripheral neuropathy) • Re-initiation of SGLT-2 inhibitors after treatment and satisfactory resolution of a foot complication that occurred whist taking SGLT-2 inhibitors	**2B**
	**FRACTURE RISK**	
**1**	In people with CKD treated with SGLT-2 inhibitors, we suggest monitoring of bone parameters including calcium, phosphate and PTH should be performed as appropriate for CKD stage (see NICE NG203)	**2D**
	**MULTIMORBIDITY AND FRAILTY**	
**1**	We suggest an approach to care that takes account of frailty and multimorbidity where these apply. This can include: • Establishing the person’s goals, values and priorities • Consideration of the balance of disease and treatment burden (for example, prognostic benefits in people with limited life expectancy or frailty) • Agreeing an individualised management plan	**2D**
	**MYCOTIC GENITAL INFECTIONS AND FOURNIER’S GANGRENE**	
**1**	We recommend that all people are counselled on the risks of mycotic genital infections prior to initiation of SGLT-2 inhibitors	**1D**
**2**	We recommend that all people are counselled on self-care to maintain good genital hygiene	**1C**
**3**	We recommend that all people are counselled on the symptoms of mycotic genital infections and how to seek help including self-management	**1D**
**4**	We suggest that for those individuals with a history of recurrent mycotic genital infections on SGLT-2 inhibition, consideration is given to offering prophylactic anti-fungal treatment, which should be reviewed after 6 months of therapy or earlier if clinically indicated	**2D**
**5**	We suggest that SGLT-2 inhibitor therapy can be continued during the treatment of mycotic genital infections	**2D**
**6**	We highlight the specific MHRA warning and suggest that all people are counselled on the symptoms of Fournier’s gangrene and advised to stop SGLT-2 inhibitors and to seek urgent help if they develop such symptoms	**2D**
	**URINARY TRACT INFECTION**	
**1**	We recommend temporary discontinuation of SGLT-2 inhibitors when treating acute pyelonephritis or urosepsis (see sick-day guidance in Section 5a.1.2 of the supplementary appendix)	**1C**
	**CHILDREN, PREGNANCY AND BREASTFEEDING**	
**1**	We suggest that all women of child-bearing potential are counselled, prior to conception, on the risks of SGLT-2 inhibitors during pregnancy	**2D**
**2**	We suggest SGLT-2 inhibitor therapy is discontinued upon planning, suspicion or confirmation of pregnancy	**2D**
**3**	We suggest SGLT-2 inhibitors are not used in women who are breastfeeding	**2D**
	**PEOPLE WITH TYPE 1 DM**	
**1**	We recommend that SGLT-2 inhibitors be initiated in people with type 1 DM, only under the strict direction of the diabetes team	**1C**
**2**	We suggest considering referring people with type 1 DM to the specialist diabetes team, for consideration of an SGLT-2 inhibitor, if they have an eGFR ≥ 20 mL/min/1.73m^2^, and a uACR ≥ 25 mg/mmol despite being on maximum tolerated ACEi/ARB	**2C**
**3**	We recommend all people with type 1 DM started on SGLT-2 inhibitors be provided with ketone monitoring, be advised on the signs and symptoms of DKA and to seek immediate medical advice if any of these symptoms develop or ketone levels are > 0.6 mmol/L	**1B**
	**KIDNEY TRANSPLANT RECIPIENTS**	
**1**	There is currently insufficient evidence on safety and efficacy to provide Recommendations for use of SGLT-2 inhibition in people with a functioning kidney transplant	***-***
**2**	Any use of SGLT-2 inhibition to treat diabetes mellitus in a kidney transplant recipient should be evaluated by multi-disciplinary discussion	**2D**
	**ACUTE DECOMPENSATED HEART FAILURE**	
**1**	We suggest initiating SGLT-2 inhibition in people with CKD (eGFR ≥ 20 mL/min/1.73m^2^) with acute decompensated heart failure	**2B**

**Table 4:** Clinical research recommendations

**PEOPLE WITH OR WITHOUT TYPE 2 DIABETES**	
We recommend further research including, wherever possible, randomised trials to establish definitively:	
1	The effects of SGLT-2 inhibition on cardiac and kidney outcomes in people with polycystic kidney disease
2	Safety, cardiovascular and kidney effects of SGLT-2 inhibition on kidney outcomes in people with a functioning kidney transplant (see section 7b of the supplementary appendix)
3	Pharmacokinetics, cardiovascular effects and residual kidney function preservation effects of SGLT-2 inhibition in people on dialysis
4	The safety and efficacy of adding MRA to SGLT-2 inhibition in people with CKD (particularly non-steroidal MRAs with proven cardiovascular and kidney-related benefits)
5	The safety and efficacy of combining SGLT-2 inhibition with a glucagon-like peptide-1 (GLP-1) receptor agonists in people with CKD
6	Detailed cost effectiveness analyses of SGLT-2 inhibition in CKD considering effects across the full range of eGFR and uACR categories
**FRACTURE RISK**	
1	Establishing any long-term impact of SGLT-2 inhibition on the development and progression of CKD mineral bone disease (CKD-MBD)
2	Establishing if SGLT-2 inhibition modifies osteoporosis risk posed by thiazolidinediones
**MULTIMORBIDITY AND FRAILTY**	
1	Future trials of SGLT-2 inhibitor use in people with CKD that seek to extend inclusivity to those of advanced age and multimorbid status
**PEOPLE WITH TYPE 1 DIABETES**	
1	To establish whether the cardiovascular and kidney benefits of SGLT-2 inhibitors extend to those with type 1 DM
2	To establish the safety of SGLT-2 inhibitors in people with type 1 DM and chronic kidney disease
**KIDNEY TRANSPLANT RECIPIENTS**	
1	The generation of reliable randomised trial evidence for transplant recipients is a key research Recommendation
**ACUTE DECOMPENSATED HEART FAILURE**	
1	Large randomised placebo-controlled clinical trials powered to assess hard clinical outcomes in people with ADHF

**Table 5:** Audit recommendations

We propose the following audit measures focusing on those guidelines supported by robust randomised evidence:	
1	The proportion of people with each grade 1 Recommendation for use prescribed an SGLT- 2 inhibitor (with exploration of reasons for non-use to direct quality improvement projects)
2	The proportion of people prescribed an SGLT-2 inhibitor not on concomitant RAS blockade
3	The proportion of people with CKD on SGLT-2 inhibitors with evidence of provision of sick day guidance
4	The proportion of people with CKD in whom SGLT-2 inhibitors were withheld during acute illness, and the proportion appropriately re-initiated on recovery
5	The proportion of people on Insulin/SUs with HbA1c < 58 mmol/mol and eGFR > 45 mL/min/1.73m^2^, whose therapy was appropriately reduced when initiating SGLT-2 inhibitors

The Recommendations in this guideline were supported by a series of systematic literature searches for relevant SGLT-2 inhibitor randomised controlled trials, covering the period from database inception to 5^th^ September 2022. Eligible studies were published parallel-group randomised controlled trials of SGLT-2 inhibitor versus placebo, active comparator or control, excluding phase 1 studies, studies in healthy volunteers, and non-English language reports. Trials were further subcategorised into large placebo-controlled trials and into subgroups of interest. Full details regarding search methodology can be found in the full guideline document (Supplementary appendix). The acknowledgements section and full guideline text provide more information on methods used to arrive at a Recommendation.

### Recommendations for use of SGLT-2 inhibitors in people with type 2 diabetes

Due to the benefits of SGLT-2 inhibitors on kidney outcomes (CKD and acute kidney injury (AKI)) and cardiovascular risk:

1. We recommend initiating SGLT-2 inhibition in people with chronic kidney disease and type 2 diabetes, irrespective of primary kidney disease,* for any of the following 4 clinical scenarios (Grade 1A):
  - eGFR of 20–45 mL/min/1.73 m^2^
  - eGFR of > 45 mL/min/1.73m^2^ and a urinary albumin-to-creatinine ratio (uACR) of ≥ 25 mg/mmol†
  - Symptomatic heart failure, irrespective of ejection fraction
  - Established coronary disease

* excludes people with polycystic kidney disease, type 1 diabetes, or a kidney transplant

† urinary protein-to-creatinine ratio of 35 mg/mmol can be considered equivalent

#### Rationale

**Fig. 2:**
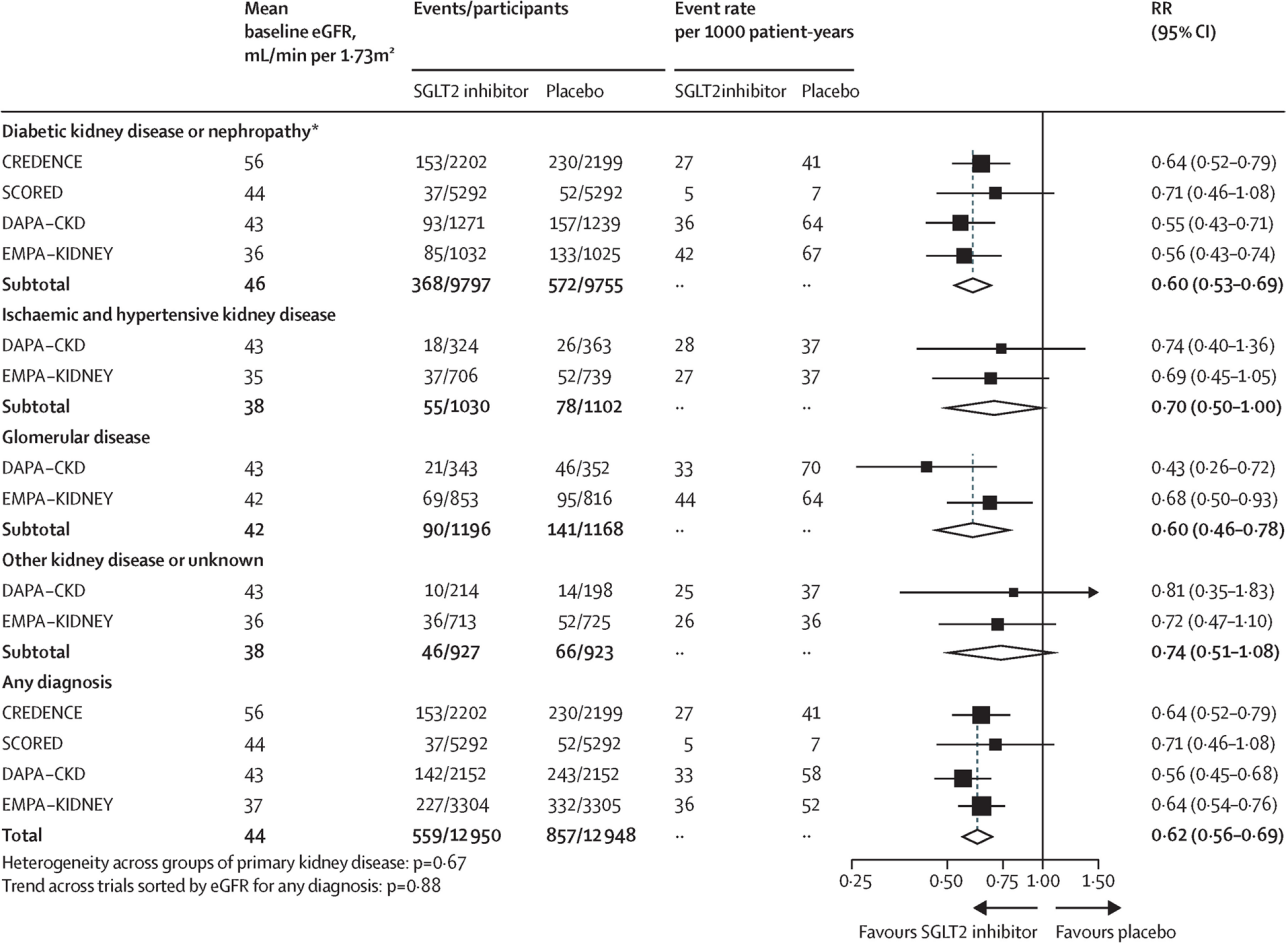
Effects of SGLT-2 inhibition on kidney disease progression by primary kidney diagnosis (adapted from [4]). *RR in the diabetic kidney disease or nephropathy subgroup excluding SCORED (which did not formally assess primary kidney disease) is 0.59 (95% CI 0.52–0.68). Figure licensed under Creative Commons CC-BY license

**Fig. 3:**
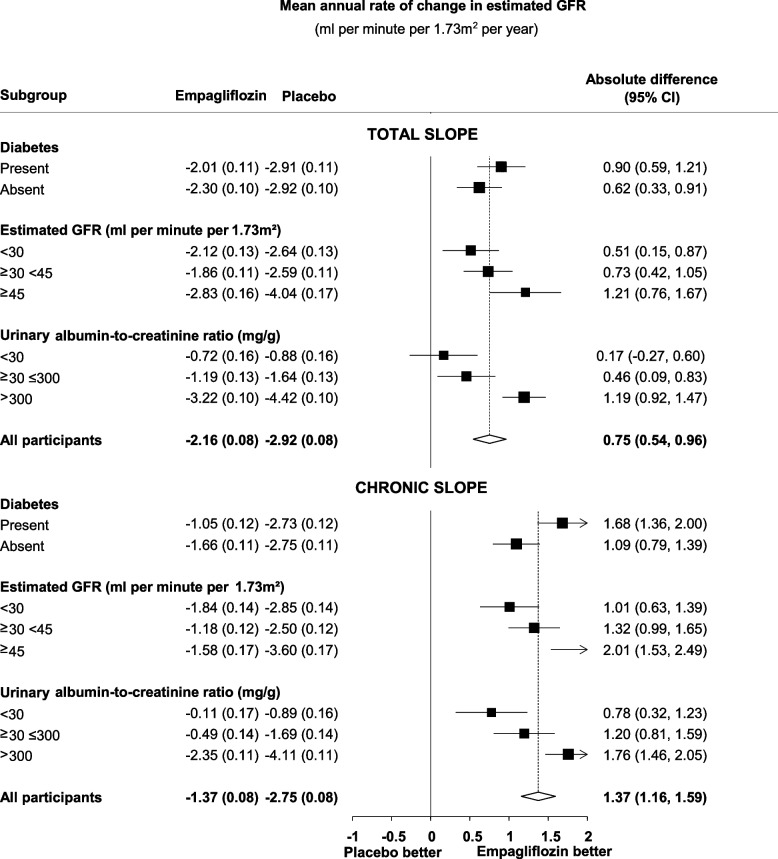
Effects of empagliflozin versus placebo on the rate of eGFR decline in EMPA-KIDNEY, by key subgroups (adapted from [3]). Mean annual rate of change in eGFR (mL/min/1.73m^2^/year) from baseline to final follow-up visit (total slope) and from two months to final follow-up visit (chronic slope). The long-term (i.e. chronic slope) is emphasised as other data has demonstrated that the acute negative eGFR dip on initiation of SGLT2 inhibitors reverses on cessation, which is not accounted for in total slope analyses. This is particularly relevant when studying people whose kidney disease progresses slowly over short periods of time (e.g. 2 years), during which time progression is likely to be less than the acute negative eGFR dip (as was the case in the uACR < 30 mg/g group). Figure licensed under CC BY-ND 4.0 International license

CREDENCE, DAPA-CKD and EMPA-KIDNEY have consistently shown that SGLT-2 inhibition significantly and importantly reduces the risk of progression of CKD in broad ranges of people with CKD, including in people with diabetic kidney disease and kidney disease of non-diabetic aetiology [1–4]. SGLT-2 inhibitors also reduce risk of acute kidney injury and cardiovascular disease [4, 5]. Participants were enrolled down to an estimated glomerular filtration rate (eGFR) of 20 mL/min/1.73m^2^, and there is no evidence that the beneficial effects of SGLT-2 inhibition on kidney disease progression or cardiovascular risk are attenuated across the spectrum of eGFR studied [4]. In EMPA-KIDNEY, participants were enrolled with eGFR 20-45 mL/min/1.73m^2^ irrespective of uACR, or eGFR >45 mL/min/1.73m^2^ with uACR ≥23 mg/mmol [3]. EMPA-KIDNEY and DAPA-CKD both demonstrated consistent benefits irrespective of primary kidney diagnosis subdivided by diabetic kidney disease, ischaemic and hypertensive nephropathy, and glomerular disease (Fig. 2) [1, 3, 4]. Among non-albuminuric kidney disease, data from eGFR slope analyses in EMPA-KIDNEY demonstrates reductions in rate of eGFR decline of a magnitude that would be expected to translate into meaningful reductions in progression of CKD (Fig. 3), which is supported by similar findings in eGFR slope analyses from EMPEROR-REDUCED, EMPEROR-PRESERVED, DAPA-HF and DELIVER [3, 8–12]. We therefore provide grade 1A Recommendation for use in people with eGFR 20-45 mL/min/1.73m^2^ or with eGFR ≥45 mL/min/1.73m^2^ and uACR ≥25 mg/mmol. Note that the 25 mg/mmol threshold for uACR was chosen pragmatically, given that this value is expected to be easier to recall for practicing clinicians than a cut-off of 23 mg/mmol, as is commonly used in the CKD trials.

Consistent findings from five SGLT-2 inhibitor trials in people with symptomatic heart failure have demonstrated that SGLT-2 inhibition reduces the risk of cardiovascular death or hospitalisation for heart failure among this population [4, 6, 7, 11–14]. These findings have been demonstrated in people with reduced and preserved ejection fraction, and in people with recent hospitalisation for worsening heart failure [7, 12, 14]. There has been no evidence that the cardiac benefits of SGLT-2 inhibition are modified by diabetes status or by eGFR [4]. Among CKD populations, cardiovascular death or hospitalisation for heart failure has been shown to be reduced by SGLT-2 inhibition in CREDENCE, SCORED and DAPA-CKD [1, 2, 15]. The totality of evidence indicates a reduction of these outcomes of approximately one quarter in people treated with SGLT-2 inhibition compared to placebo [4]. We therefore provide grade 1A Recommendation for use in people with CKD for this indication. Those with prior coronary disease are at high risk of major adverse cardiovascular events (MACE) and heart failure and are included in this Recommendation based on the totality of the evidence [5].

2. We suggest initiating SGLT-2 inhibition to modify cardiovascular risk and slow rate of kidney function decline in people with an eGFR > 45–60 mL/min/1.73m^2^ and a uACR of < 25 mg/mmol, recognising effects on glycaemic control will be limited (Grade 2B).

#### Rationale

Meta-analysis of large randomised clinical trials of SGLT-2 inhibition indicates that cardiovascular benefits of SGLT-2 inhibition, particularly reducing the risk of cardiovascular death or hospitalisation for heart failure, are present irrespective of trial-level average eGFR [4]. The benefits of SGLT-2 inhibition in terms of reducing progression of kidney disease are also not modified by eGFR in individual trials [1–3]. eGFR slope analyses from heart failure and CKD trials consistently demonstrate reduced rates of kidney function decline among people treated with SGLT-2 inhibition, including those without significant albuminuria, as demonstrated in EMPA-KIDNEY (Fig. 3) [3, 8–12]. EMPEROR-REDUCED, EMPEROR-PRESERVED, DAPA-HF and DELIVER all show reductions in the rate of kidney function decline with SGLT-2 inhibition compared to placebo, while in EMPEROR-REDUCED this reduction in rate of eGFR decline is present in the presence and absence of CKD and across the spectrum of albuminuria [8–12]. Therefore, while this population has not been directly studied in randomised trials, there is indirect evidence to support reduction in cardiovascular risk and reduced rate of kidney function decline in this group, for which we provide a grade 2B Recommendation.

3. We suggest clinicians consider initiating SGLT-2 inhibition in people with an eGFR below 20 mL/min/1.73m^2^ to slow progression of kidney disease (Grade 2B)

#### Rationale

**Fig. 4:**
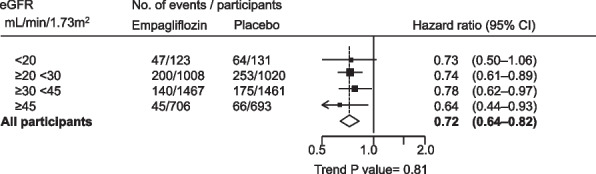
Effects of empagliflozin versus placebo on the primary outcome of EMPA-KIDNEY, by baseline eGFR (post-hoc analysis). Post-hoc analysis of unpublished data from EMPA-KIDNEY

Clinical trials of SGLT-2 inhibition conducted in populations with CKD have continued SGLT-2 inhibition until the initiation of kidney replacement therapy, providing indirect evidence to support the use of this SGLT-2 inhibition in this population [1–3]. Furthermore, data from EMPA-KIDNEY indicates that the benefit of SGLT-2 inhibition in terms of progression of kidney disease is not attenuated in people with an eGFR < 20 mL/min/1.73m^2^ (Fig. 4). There is no suggestion from the data that kidney benefits of SGLT-2 inhibition begin to attenuate down to an eGFR of 15 mL/min/1.73m^2^, and those with very low eGFR are at particularly high risk (Fig. 4). We therefore provide a 2B Recommendation for use in people with eGFR < 20 mL/min/1.73m^2^.

### Recommendations for use of SGLT-2 inhibitors in people without diabetes

1. We recommend initiating SGLT-2 inhibition in people with chronic kidney disease, irrespective of primary kidney disease,* for any of the following clinical scenarios (Grade 1A):
  - eGFR of ≥ 20 mL/min/1.73m^2^ and a urinary albumin-to-creatinine ratio (uACR) of ≥ 25 mg/mmol†
  - Symptomatic heart failure, irrespective of ejection fraction

* excludes people with polycystic kidney disease, type 1 diabetes, or a kidney transplant

† urinary protein-to-creatinine ratio of 35 mg/mmol can be considered equivalent

#### Rationale

SGLT-2 inhibition has been shown to be effective in people with albuminuric chronic kidney disease across broad population, including people with and without type 2 diabetes, and down to an eGFR of 20 mL/min/1.73m^2^ [1–4]. Meta-analysis of CKD trials and all SGLT-2 inhibitor trials demonstrates that the kidney benefits of SGLT-2 inhibition are not modified by the presence or absence of diabetes [4]. Furthermore, DAPA-CKD and EMPA-KIDNEY both indicate consistent benefits irrespective of primary kidney disease, with benefits observed in glomerular disease of a similar magnitude to those seen in diabetic kidney disease [1, 3, 4].

SGLT-2 inhibition has been demonstrated to reduce the risk of heart failure hospitalisation in people with stable established symptomatic heart failure with reduced ejection fraction (HFrEF) by the DAPA-HF and EMPEROR-REDUCED trials, with relative effects similar in people with and without DM [11, 13]. Data from EMPEROR-PRESERVED and DELIVER confirm benefits on heart failure complications in people with heart failure with preserved ejection fraction (HFpEF), including people without DM [7, 12]. The four large trials recruited a substantial proportion of people with CKD, with cardiac benefits appearing to be unmodified by moderately reduced levels of eGFR [4, 6]. We therefore provide a grade 1A Recommendation for use of SGLT-2 inhibition in people without DM with an eGFR ≥20 mL/min/1.73m^2^ and uACR ≥25 mg/mmol, or those with symptomatic heart failure. Note that the 25 mg/mmol threshold for uACR was chosen pragmatically, given that this value is expected to be easier to recall for practicing clinicians than a cut-off of 23 mg/mmol, as is commonly used in the CKD trials.

2. We recommend initiating SGLT-2 inhibition to slow rate of kidney function decline in people with an eGFR of 20–45 mL/min/1.73 m^2^ and a uACR of < 25 mg/mmol* (Grade 1B)

* urinary protein-to-creatinine ratio of 35 mg/mmol can be considered equivalent.

#### Rationale

**Fig. 5:**
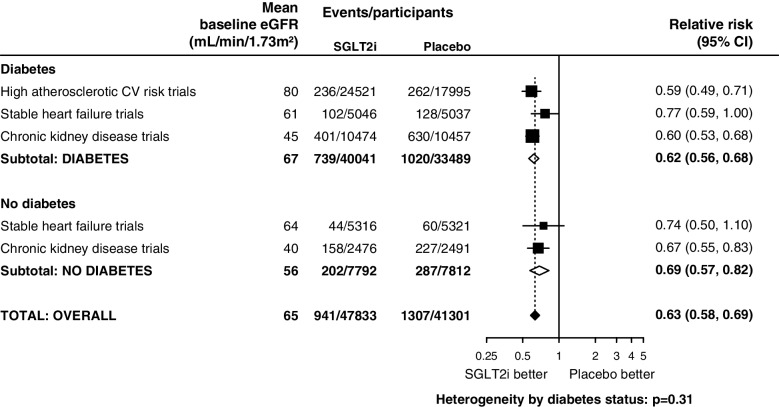
Effects of SGLT-2 inhibitors on kidney disease progression by population (adapted from [4]). Kidney disease progression was defined as a sustained ≥ 50% decline in eGFR from randomisation, kidney failure, or death from kidney failure. Data not available for SOLOIST-WHF. Figure licensed under Creative Commons CC-BY license

The excellent safety profile of SGLT-2 inhibition in people with CKD without DM has been established in nearly 5000 such people from DAPA-CKD and EMPA-KIDNEY [1, 3]. DAPA-CKD and EMPA-KIDNEY demonstrated beneficial effects across the spectrum of eGFR in terms of progression of kidney disease in people with and without diabetes [4] (Fig. 5). Furthermore, EMPA-KIDNEY also showed that among people without albuminuria rate of kidney function decline (chronic eGFR slope) was reduced by 0.78 mL/min/1.73m^2^ per year in participants with A1 levels of albuminuria (<30 mg/g) to a rate of -0.11 mL/min/1.73m^2^ per year, and by 1.20 mL/min/1.73m^2^ per year in participants with A2 levels (30-299 mg/g; Fig. 3) [3]. Such absolute benefits would be predicted to translate into clinically meaningful reductions in progression of kidney disease, even among people with low albuminuria at baseline. Among those with low levels of albuminuria and low eGFR at initiation, such effects on eGFR decline could translate into delay in the onset of kidney failure if used over a period of years. Furthermore, SGLT-2 inhibition has been shown to reduce the risk of AKI and cardiovascular risk [4], as well as hospitalisations from any cause [3] . Given the clear magnitude of benefit of SGLT-2 inhibition in this population and the reassuring safety profile, we provide a grade 1 Recommendation for this statement. However, at present only EMPA-KIDNEY provides direct evidence in this population, with further supporting evidence obtained from eGFR slopes of the trials in heart failure (i.e. grade B level of evidence). Cost-effective analyses in this group would provide valuable additional information.

3. We suggest clinicians consider initiating SGLT-2 inhibition in people with an eGFR below 20 mL/min/1.73m^2^ to slow progression of kidney disease (Grade 2B)

#### Rationale

Two of the four clinical trials of SGLT-2 inhibition conducted in people with CKD have enrolled people without diabetes (DAPA-CKD and EMPA-KIDNEY), and in both trials SGLT-2 inhibition has been continued below an eGFR of 20 mL/min/1.73m^2^ without evidence of increased adverse events [1, 3]. In EMPA-KIDNEY, in which over half of the participants did not have diabetes, SGLT-2 inhibition was shown to have consistent relative benefits across the spectrum of eGFR, including among people with eGFR < 20 mL/min/1.73m^2^ (Fig. 4) [3]. Given that people with low eGFR are at high risk of progression to kidney failure, the absolute benefit is likely to be considerable for this population, irrespective of diabetes status. We therefore provide a grade 2B Recommendation for initiation of use of SGLT-2 inhibition for people without type 2 DM with eGFR < 20 mL/min/1.73m^2^.

### Recommendations for implementation in people with or without type 2 diabetes

1. We recommend using SGLT-2 inhibitors with demonstrated efficacy for their given indications (Grade 1A).

#### Rationale

Government regulators review data from randomised trials and assess their reliability through regulatory inspections. Regulatory licences/indications therefore provide a key guide to which SGLT-2 inhibitors have generated definitive evidence of efficacy and safety for a given use. We therefore recommend selecting SGLT-2 inhibitors according to these licensed indications, wherever possible (summaries of which are provided in section 4 of the Supplementary appendix).

2. We recommend using clinically appropriate single agent renin-angiotensin system (RAS) blockade in combination with SGLT-2 inhibition, wherever RAS blockade is indicated and tolerated (Grade 1A)

#### Rationale

These clinical practice guidelines pertain to use of SGLT-2 inhibition in people with CKD. The standard of care in many forms of CKD is the use of RAS blockers [16, 17], with clear evidence of benefit in diabetic nephropathy [18, 19]. All CREDENCE participants were on stable maximally tolerated RAS blockade [2], as were 97% of DAPA-CKD participants [1] and 85% of participants in EMPA-KIDNEY [3]. We therefore provide a grade 1A Recommendation to prescribe RAS blockade and ensure clinically appropriate dosing alongside any SGLT-2 inhibitor use. Note that it has been suggested that, mechanistically, SGLT-2 inhibition may have the potential to activate RAS [20]. However the large trials in people with type 2 DM at high atherosclerotic cardiovascular risk have been combined in meta- analysis and have raised a hypothesis that the benefits of SGLT-2 inhibitors on kidney disease progression could extend to people with type 2 DM not on RAS blockade [21]. The lack of heterogeneity of effect of empagliflozin on the primary composite outcome stratified by use of RAS inhibition in EMPA-KIDNEY lends further support to this hypothesis [3].

Note that we recommend single agent RAS blockade, as combination therapy (i.e. dual blockade with angiotensin-converting enzyme inhibitor [ACEi] plus angiotensin receptor blocker [ARB]) has been found to increase the risk of serious hyperkalaemia or acute kidney injury, and has not been shown to importantly slow CKD progression [22].

3. We suggest following NICE guidelines on use of uACR for screening of albuminuria (NICE NG203). We recognise that more pragmatic approaches to identifying risk of kidney disease progression may be necessary whilst local access to uACR measurement is improved (Grade 2C).

#### Rationale

Many factors can cause transient increases in albuminuria (including urinary tract infection, exercise, and menstruation) and as such, the National Institute for Health and Care Excellence (NICE) [23] and other international guideline groups [24] recommend that repeat testing should take place within 3 months if a single uACR result is between 3-69 mg/mmol. An early morning sample offers some advantages due to reduced impact of hydration status and exercise [25], but if unavailable, random sampling may still offer a reliable indication of total daily albuminuria [26]. A uACR value ≥70 mg/mmol generally does not require further confirmation, as this is consistent with clinically significant proteinuria [27].

We agree with the statement within the NICE CKD guidelines that reagent strips and protein-to-creatinine ratio measurements should not be used to quantify albuminuria [23]. Large-scale meta-analysis and other observational data have shown that dipstick values using reagent strips are neither sensitive, nor specific enough to predict uACR accurately [28]. However, we recognise that uACR testing may not be regularly undertaken in some areas of the UK, and local methods of assessing risk may need to be used to ensure those at risk are offered SGLT-2 inhibition.

4. We recommend that SGLT-2 inhibition can be continued until the need for dialysis or kidney transplantation arises (Grade 1A).

#### Rationale

Data from CKD trials include many hundreds of participants with an eGFR below 20 mL/min/1.73m^2^ [1–3]. Continued use of SGLT-2 inhibitors until the need for dialysis or kidney transplantation was the practice in these trials, which have confirmed benefits exceed any harms. In subgroup analyses, kidney benefits are unmodified by baseline eGFR. At a population level, people with a low eGFR are at highest absolute risk of kidney failure, and are therefore most likely to benefit (in absolute terms).

5. We suggest that co-prescription of SGLT-2 inhibition with MRA can be considered, where each are individually indicated (Grade 2B).

#### Rationale

Subgroup analyses from the SGLT-2 inhibitor trials in non-CKD populations suggest cardiac and kidney benefits are likely to be maintained in people co-prescribed an MRA with an SGLT-2 inhibitor, with no increased risk of hyperkalaemia caused by SGLT-2 inhibitor use [6, 14, 29–34]. CREDENCE, DAPA-CKD and EMPA-KIDNEY provide reassuring evidence that SGLT-2 inhibition does not usually cause hyperkalaemia in CKD populations [1–3]. We therefore provide a grade 2B suggestion that MRA can be used with SGLT-2 inhibitors. Note that guidance on how to monitor for changes in eGFR and potassium in those on MRA are outside of the scope of this guideline.

6. We suggest the beneficial effects of SGLT-2 inhibition on kidney disease progression or risk of heart failure hospitalisation are likely to be a class effect (Grade 2B)

#### Rationale

We have recommended using SGLT-2 inhibitors with demonstrated efficacy for their given indications, but as more large trials report results testing the available SGLT-2 inhibitors in overlapping populations, it is increasingly apparent that any differences between the individual molecules do not appear to create large differences in clinical efficacy. For example, CREDENCE (canagliflozin), DAPA-CKD (dapagliflozin) and EMPA-KIDNEY (empagliflozin) reported relative risk reductions on their respective kidney disease progression outcomes which were comparable in their respective (sub)populations with type 2 DM [1–4, 35]. Beneficial effects on cardiovascular death or hospitalisation for heart failure were consistent in the CKD trials with the totality of evidence across all SGLT-2 inhibitor trials, including those in heart failure-specific populations [4]. Likewise, the HFrEF trials DAPA-HF (dapagliflozin) and EMPEROR-REDUCED (empagliflozin) [11, 13], and the HFpEF trials DELIVER (dapagliflozin) and EMPEROR-PRESERVED (empagliflozin) [7, 12], share similar designs and results of primary and secondary assessments overall and across subgroups are remarkably consistent [6].

Relative risk reductions on major adverse cardiovascular events (MACE) across key cardiovascular safety trials [36] and trials in dedicated CKD populations are also not statistically different from each other [1, 15]. Meta-analyses demonstrate consistent benefits of SGLT-2 inhibition in terms of cardiovascular and kidney benefits without apparent heterogeneity by class overall and stratified by diabetes status [4, 5]. We are of the opinion that the larger effects of empagliflozin on cardiovascular death in EMPA-REG OUTCOME [37], and the larger effects on non-cardiovascular death in DAPA-CKD compared to other SGLT-2 inhibitor trials [5, 38] are more likely represent the play of chance or be caused by factors other than minor differences in the biological action of different SGLT-2 inhibitors. We therefore suggest there is increasing evidence that the cardiac and kidney benefits of SGLT-2 inhibition represent a class effect.

It should be noted, however, that SGLT-2 inhibitors differ in their respective receptor selectivity and there may be an increased propensity to cause diarrhoea and volume depletion when using SGLT-2 inhibitors that also meaningfully inhibit gut SGLT-1 (e.g. sotagliflozin [14]). Selectivity for SGLT-2 over SGLT-1 ranges from: ~20:1 for the dual SGLT-1/2 inhibitor sotagliflozin [39], and from ~250:1 for canagliflozin to ~2500:1 for empagliflozin [40] for the more selective SGLT-2 inhibitors.

### Recommendations for implementation: Diabetic Ketoacidosis (DKA)

1. We recommend that people with type 1 DM should only have SGLT-2 inhibitors initiated under the strict direction of the diabetes team (see section 7a of the supplementary appendix) (Grade 1C).
2. We recommend that people with type 2 DM at greater risk of DKA (defined in Table 5a.1 of the supplementary appendix) should have SGLT-2 inhibitors initiated with caution after discussion with the diabetes team (Grade 1C).
3. We recommend SGLT-2 inhibitors are discontinued when an individual develops DKA (Grade 1A).
4. We suggest that after an episode of DKA and where a clear contributing factor has been identified, there should be discussion with the person and clinical team to establish whether the benefits of re- introducing an SGLT-2 inhibitor outweigh the risks (Grade 2D).
5. When initiating SGLT-2 inhibitors, we suggest that individuals should be advised on the signs and symptoms of DKA and be instructed to temporarily withhold SGLT-2 inhibitors and to seek immediate medical advice if symptoms develop (Grade 1C).
6. We recommend always offering advice on sick day guidance when initiating SGLT-2 inhibitors and reminding them of this at every medication review (Grade 1C).
7. We suggest that individuals taking SGLT-2 inhibitors should be advised against following a ketogenic diet (Grade 2C).
8. We suggest that for people who choose to intermittently fast (e.g. for Ramadan), and particularly for those who are elderly, on diuretics or have CKD, consider withholding SGLT-2 inhibitors for the duration of the fasting period and for those people with diabetes ketone testing should be undertaken if unwell (Grade 2D).

#### Rationale

The evidence from the studies reviewed indicates that diabetic ketoacidosis (DKA) is a recognised complication in people treated with SGLT-2 inhibitors and that it is more commonly found in conjunction with dehydration or infection [1–4]. DKA is also likely to occur more frequently in people who are insulin deficient which would include people with type 1 DM, people with type 2 DM with a relative insulin deficient phenotype, and situations where people on insulin have their insulin dose reduced substantially [41]. These Recommendations will allow clinicians to use SGLT-2 inhibitors in those who are likely to benefit from this treatment and yet also minimise the risk of the complication of DKA.

### Recommendations for implementation: hypoglycaemia

1. We recommend considering reducing the dose of insulin/SUs/meglitinides when initiating SGLT-2 inhibitors to reduce the risk of hypoglycaemia (Grade 1C).
2. We recommend that when initiating SGLT-2 inhibitors in people taking SUs (e.g. gliclazide) or meglitinides (e.g. repaglinide) when the HbA1c < 58 mmol/mol AND eGFR > 45 mL/min/1.73m^2^, consider reducing dose of SU or meglitinide by 50% to reduce risk of hypoglycaemia (Grade 1C).
3. We recommend that when starting SGLT-2 inhibitors in people taking insulin when the HbA1c < 58 mmol/mol AND eGFR > 45 mL/min/1.73m^2^, consider reducing the insulin dose by 20% to avoid hypoglycaemia (Grade 1C).
4. We recommend that when starting SGLT-2 inhibitors in people taking only metformin ± pioglitazone ± DPP-4i/gliptins or GLP-1RA therapy, no dosage adjustment is necessary (Grade 1C).

#### Rationale

SGLT-2 inhibitors are effective drugs at reducing hyperglycaemia when they are used in people with preserved kidney function (e.g. eGFR >60 mL/min/1.73m^2^), however, their glycaemic effectiveness reduces as the eGFR declines [42, 43]. Where a treatment for DM carries a risk of hypoglycaemia (such as SUs and insulin use), the addition of an SGLT-2 inhibitor may potentiate that risk, particularly if baseline glycaemic control is reasonable at the time of initiation of treatment. There is no evidence that SGLT-2 inhibitors cause significant hypoglycaemia on their own or in addition with DM medicines that are not associated with hypoglycaemia [4, 44, 45].

### Recommendations for implementation: acute kidney injury (AKI), hypovolaemia and potassium

1. We recommend that individuals initiated on an SGLT-2 inhibitor do not routinely require an early assessment of kidney function or potassium following initiation of treatment (Grade 1C).
2. We suggest that if an individual has a kidney function assessment within the first few weeks post initiation of an SGLT-2 inhibitor, a decline in eGFR needs to be interpreted with caution and in the context of an expected drug effect to avoid unwarranted discontinuation of treatment (Grade 2B).
3. We suggest that individuals on diuretics are counselled on the symptoms of hypovolaemia and advised to seek medical attention if they develop any such symptoms after starting SGLT-2 inhibition (Grade 2B).
4. We suggest that clinicians consider an early clinical review and if appropriate a diuretic or antihypertensive dose reduction in individuals they consider at high risk of hypovolaemia (Grade 2C).
5. We recommend that SGLT-2 inhibitors are temporarily withheld during acute illness (Grade 1C).

#### Rationale

SGLT-2 inhibitors have proven benefit in relation to reducing the rate of long-term decline in kidney function in certain groups of people with CKD. The means by which they provide this benefit may involve changes to intraglomerular pressure and reduction in hyperfiltration at an individual glomerulus level. This can result in a reduction in eGFR over the initial few weeks following initiation of SGLT-2 inhibitors, which is relatively small, largely reversible and should not usually be seen as an adverse effect of the drug [46].

None of the major studies have demonstrated an increased risk of acute kidney injury (AKI) in people treated with SGLT-2 inhibitors, and it seems likely they have renal tubular protective effects that reduce risk of AKI [4]. It is therefore important that early changes in eGFR that occur following initiation of SGLT-2 inhibitors do not routinely result in withdrawal of SGLT-2 inhibition when people are likely to gain significant benefit from them.

In addition, SGLT-2 inhibitors have a combined osmotic diuretic and natriuretic effect, so clinicians and the people treated with SGLT-2 inhibitors need to be aware of this effect in order to ensure that any risk of hypovolaemia is minimised.

### Recommendations for implementation: peripheral vascular disease (PVD) and amputation risk

1. We suggest avoiding initiation of SGLT-2 inhibitors in the presence of active foot disease (infection, ulceration and ischaemia) and withholding treatment in those who develop foot complications whilst taking an SGLT-2 inhibitor (Grade 2B).
2. We suggest a shared decision-making approach, with appropriate counselling on risks and benefits of treatment and the importance of routine preventative foot care measures for:
  Individuals at high risk of amputation (previous amputations, existing PVD, peripheral neuropathy)
  Re-initiation of SGLT-2 inhibitors after treatment and satisfactory resolution of a foot complication that occurred whist taking SGLT-2 inhibitors (Grade 2B).

#### Rationale

A significant finding from a single large trial using the SGLT-2 inhibitor canagliflozin alerted clinicians to the possibility that SGLT-2 inhibitors could increase the risk of lower limb amputations [47]. This finding has not been confirmed in other large trials [4] and furthermore it is important to appreciate that people with peripheral vascular disease (PVD) are a group of individuals who have more to gain from the initiation of SGLT-2 inhibitors in relation to protection against risk of cardiovascular death, myocardial infarction, heart failure complications and progression of CKD. It is therefore important not to exclude these individuals from the potential benefits of SGLT-2 inhibitors, but to ensure that these medicines are used appropriately and safely in people at risk, or with evidence of PVD.

### Recommendations for implementation: fracture risk

1. In people with CKD treated with SGLT-2 inhibitors, we suggest monitoring of bone parameters including calcium, phosphate and parathyroid hormone should be performed as appropriate for CKD stage (NICE NG203) (Grade 2D).

#### Rationale

Whilst there has been report of an increased risk of fractures in one trial where participants were treated with canagliflozin, this has not been confirmed in any other study and may represent the play of chance [4, 47]. People with CKD are at increased risk of bone disease and their clinician should be monitoring them to ensure that interventions are utilised to maintain good bone health irrespective of the prescription of SGLT-2 inhibitors. NICE NG203 CKD guidance is available at https://www.nice.org.uk/guidance/ng203.

### Recommendations for implementation: multimorbidity and frailty

1. We suggest an approach to care that takes account of frailty and multimorbidity where these apply. This can include:
  Establishing the person’s goals, values and priorities
  Consideration of the balance of disease and treatment burden (for example, prognostic benefits in people with limited life expectancy or frailty)
  Agreeing an individualised management plan (Grade 2D).

#### Rationale

When making decisions on which individuals would benefit from SGLT-2 inhibition one has to consider the participants included in the relevant trials that provided the evidence for their use. These trials generally excluded people with greater degrees of frailty and certain comorbidities. Therefore, caution must be exercised when extending evidence of safety (and perhaps also benefit) of SGLT-2 inhibitors to such individuals, although one needs to also consider at the same time that many of these individuals, and particularly those with heart failure, are likely to achieve significant benefit from the use of SGLT-2 inhibitors.

### Recommendations for implementation: mycotic genital infections and fournier’s gangrene

1. We recommend that all people are counselled on the risks of mycotic genital infections prior to initiation of SGLT-2 inhibitors (Grade 1D).
2. We recommend that all people are counselled on self-care to maintain good genital hygiene (Grade 1C).
3. We recommend that all people are counselled on the symptoms of mycotic genital infections and how to seek help including self-management (Grade 1D).
4. We suggest that for those individuals with a history of recurrent mycotic genital infections on SGLT-2 inhibition, consideration is given to offering prophylactic anti-fungal treatment, which should be reviewed after 6 months of therapy or earlier if clinically indicated (Grade 2D).
5. We suggest that SGLT-2 inhibitor therapy can be continued during the treatment of mycotic genital infections (Grade 2D).
6. We highlight the specific MHRA warning and suggest that all people are counselled on the symptoms of Fournier’s gangrene and advised to stop SGLT-2 inhibitors and to seek urgent help if they develop such symptoms (Grade 2D).

#### Rationale

Mycotic genital infections are recognised to occur more frequently in people treated with SGLT-2 inhibitors (on average risk is about 3-4-fold higher) and particularly in those individuals with DM [4, 48]. These infections are usually mild and easily treated. Good clinical care should include ensuring that individuals prescribed SGLT-2 inhibitors are aware of this complication, how to reduce the risk of it occurring and appropriate actions should they develop symptoms consistent with mycotic genital infections.

In contrast to mycotic genital infections, Fournier’s gangrene is a rare condition that results from bacterial infection and it requires prompt and intensive medical and surgical management. This disorder is identified in people with DM and whilst the evidence to suggest that it may be increased in people treated with SGLT-2 inhibitors is limited to post-marketing surveillance [49], all people starting SGLT-2 inhibitors should be advised on the symptoms of Fournier’s gangrene and what to do if they develop such symptoms.

### Recommendations for implementation: urinary tract infection

1. We recommend temporary discontinuation of SGLT-2 inhibitors when treating acute pyelonephritis or urosepsis (Grade 1C).

#### Rationale

Randomised data from major trials show the increased risk of urinary tract infections (UTIs) with SGLT-2 inhibitors is small [4]. However, these drugs are being prescribed in people who have a high risk of UTIs and effective prompt management of these infections should be undertaken.

### Recommendations for implementation: children, pregnancy and breastfeeding

1. We suggest that all women of child-bearing potential are counselled, prior to conception, on the risks of SGLT-2 inhibitors during pregnancy (Grade 2D).
2. We suggest SGLT-2 inhibitor therapy is discontinued upon planning, suspicion or confirmation of pregnancy (Grade 2D).
3. We suggest SGLT-2 inhibitors are not used in women who are breastfeeding (Grade 2D).

#### Rationale

There is theoretical evidence to advise against using these drugs in people either planning pregnancy, who become pregnant or who are breastfeeding [50–53]. Clinical trials in the paediatric setting are suggested.

### Recommendations for implementation in specific populations: people with type 1 diabetes

1. We recommend that SGLT-2 inhibitors be initiated in people with type 1 DM, only under the strict direction of the diabetes team (Grade 1C).
2. We suggest considering referring people with type 1 DM to the specialist diabetes team, for consideration of an SGLT-2 inhibitor, if they have an eGFR ≥ 20 mL/min/1.73m^2^, and a uACR ≥ 25 mg/mmol despite being on maximum tolerated ACEi/ARB (Grade 2C).
3. We recommend all people with type 1 DM started on SGLT-2 inhibitors be provided with ketone monitoring, be advised on the signs and symptoms of DKA and to seek immediate medical advice if any of these symptoms develop or ketone levels are > 0.6 mmol/L (Grade 1B).

#### Rationale

There is currently insufficient evidence to recommend the use of SGLT-2 inhibitors as an adjunct to existing therapies in the management of diabetic nephropathy in people with type 1 DM. Evidence of kidney benefits in people with type 2 DM makes this plausible but such results cannot be readily extrapolated to people with type 1 DM. Clinicians may wish to discuss treatment options with their patients and other specialists in cases where proteinuria persists despite current standard treatment. Thresholds for referral to the specialist diabetes team have been updated in the current version of this guideline in line with evidence available from trials of SGLT-2 inhibition irrespective of the presence or absence of diabetes. Furthermore, the grading for Recommendation number 2 has been increased from 2D to 2C, given evidence available from the InTandem3 trial [54].

### Recommendations for implementation in specific populations: kidney transplant recipients

1. There is currently insufficient evidence on safety and efficacy to provide Recommendations for use of SGLT-2 inhibition in people with a functioning kidney transplant.
2. Any use of SGLT-2 inhibition to treat diabetes mellitus in a kidney transplant recipient should be evaluated by multi-disciplinary discussion (Grade 2D)

Note: effects on glycaemic control at an eGFR < 60 mL/min/1.73m^2^ in people with a kidney transplant appear small and potential risk of complications from urinary tract infection should be considered.

### Recommendations for implementation in specific populations: acute decompensated heart failure

We suggest initiating SGLT-2 inhibition in people with CKD (eGFR ≥ 20 mL/min/1.73m^2^) with acute decompensated heart failure. (2B).

#### Rationale

Multiple small-scale randomised controlled trials provide evidence that SGLT-2 inhibition is safe in a population presenting to hospital with acute decompensated heart failure (ADHF) [55–59]. Furthermore, the moderate-sized EMPULSE trial demonstrates that individuals treated with SGLT-2 inhibition for ADHF are more likely to yield clinical benefit than those treated with placebo [60]. Trials in ADHF enrolled people with eGFR > 15 mL/min/1.73m^2^, with the largest trial (EMPULSE) enrolling those with eGFR ≥ 20 mL/min/1.73m^2^. More evidence from large, well-conducted randomised controlled trials (such as the DAPA ACT HF-TIMI 68 trial [61]) will provide more comprehensive evidence to support this Recommendation.

See Section 2 & 3 for Recommendations for use in other forms of heart failure or to modify cardiovascular risk.

### Clinical research and audit recommendations in people with or without type 2 diabetes

A summary of clinical research Recommendations can be found in Table 4, and a summary of audit Recommendations can be found in Table 5.

### Supplementary Information

**Additional file 1.**

## Data Availability

Not applicable.
