# Supplementary material for: UK Kidney Association Clinical Practice Guideline: Sodium-Glucose Co-transporter-2 (SGLT-2) Inhibition in Adults with Kidney Disease 2023 UPDATE
Source: BMC Nephrol. 2023 Oct 25;24:310. doi: 10.1186/s12882-023-03339-3 (PMC10598949; doi:10.1186/s12882-023-03339-3)
Supplement: Supplementary file 1 — Additional file 1. [file 12882_2023_3339_MOESM1_ESM.pdf]

UK Kidney Association Clinical Practice Guideline: Sodium-Glucose Co-transporter-2 (SGLT-2) Inhibition in Adults with Kidney Disease

2023 UPDATE

Final version: April 2023
Review date: April 2028

Authors:

Working Group co-chairs: Assoc. Prof. William G. Herrington & Dr Andrew H. Frankel

Working Group members:
Dr Alexa Wonnacott, Dr David Webb, Mrs Angela Watt, Mr Michael Watson, Mr John Roberts, Assoc. Prof. Natalie Staplin, Dr Alistair Roddick, Dr Alex Riding, Dr Eirini Lioudaki, Dr Apexa Kuverji, Prof. Mohsen El Kossi, Dr Patrick Holmes, Mr Matt Holloway, Prof. Donald Fraser, Dr Chris Carvalho, Prof. James Burton, Prof. Sunil Bhandari

See appendix for list of previous versions/revisions and working group affiliations.

Endorsements

The National Institute for Health and Care Excellence (NICE) has accredited the process used by The UK Kidney Association to produce its Clinical Practice Guidelines. Accreditation is valid until December 2023. More information on accreditation can be viewed at www.nice.org.uk/accreditation.

Method used to arrive at a recommendation

The recommendations for the first draft of this guideline resulted from a collective decision reached by informal discussion by the authors and, whenever necessary, with input from the Chair of the Clinical Practice Guidelines Committee. If no agreement had been reached on the appropriate grading of a recommendation, a vote would have been held and the majority opinion carried. However this was not necessary for this guideline.

Conflicts of Interest Statement

All authors made declarations of interest in line with the policy in the Association's Clinical Practice Guidelines Development Manual. Further details can be obtained on request from the UK Kidney Association.

Contents

Executive summary 5

Summary of recommendations 7

List of abbreviations 12

Section 1: Background, aims and concise methods 14

1.1 Summary 14

1.2 Introduction 14

1.3 Molecular mechanisms of action of SGLT-2 inhibition 15

1.4 Listing of key large-scale placebo-controlled clinical outcome trials 19

1.5 Guideline aims & development 22

1.6 References 25

Section 2: SGLT-2 inhibition and kidney protection in people with CKD in the context of type 2 diabetes mellitus 31

2.1 Background 31

2.2 Recommendations for use 48

2.3 Clinical research recommendations 49

2.4 Recommendations for implementation 50

2.5 Audit measures 52

2.6 References 53

Section 3: SGLT-2 inhibition and kidney protection in people with CKD without diabetes mellitus 58

3.1 Background 58

3.2 Recommendations for use 66

3.3 Clinical research recommendations 67

3.4 Recommendations for implementation 67

3.5 Audit measures 67

3.6 References 68

Section 4: Selection of SGLT-2 inhibitors 70

4.1 Background 70

4.2 Current licensed indications for SGLT-2 inhibitor use 72

4.3 References 74

Section 5: Prescribing SGLT-2 inhibitors safely 75

5a. Diabetic ketoacidosis 76

5b. Hypoglycaemia 80

5c. Acute Kidney Injury, Hypovolaemia and Potassium 82

5d. Peripheral vascular disease and amputation risk 85

5e. Fracture risk 87

5f. Multimorbidity and frailty 89

5g. Mycotic genital infections and Fournier's gangrene 91

5h. Urinary tract infections 94

5i. Children, pregnancy and breast feeding ... 96
5.1 References ... 98
Section 6: Lay summaries and patient information leaflets ... 104
Section 7: Use in special populations of specific interest ... 121
7a Type 1 diabetes mellitus (DM) ... 121
7b Kidney transplant recipients ... 124
7c Acute decompensated heart failure ... 127
Appendix I: Systematic literature review design and results ... 135
Systematic search design ... 135
Appendix figure 1: Summary of systematic search results ... 141
Appendix figure 2: Updated systematic search results ... 142
Appendix figure 3: Risk of bias assessment ... 143
Appendix I references ... 144
Appendix II: Revision history ... 145
Appendix III: Working group membership affiliations ... 146

Executive summary

Sodium glucose co-transporter-2 (SGLT-2) inhibitors, initially developed for the treatment of hyperglycaemia in type 2 diabetes, have been shown in large placebo-controlled randomised trials to be effective at reducing cardiovascular risk and risk of kidney disease progression in people with chronic kidney disease (CKD) irrespective of diabetes status, level of kidney function, or primary kidney diagnosis. SGLT-2 inhibitors have also been shown to be beneficial for people with diabetes at high cardiovascular risk and people with heart failure irrespective of left ventricular ejection fraction. This update to the 2021 guideline was necessary as two further important large trials reported in 2022: DELIVER, which investigated the use of the SGLT-2 inhibitor dapagliflozin in people with heart failure with preserved or mildly reduced ejection fraction, and EMPA-KIDNEY, which investigated the use of empagliflozin in people with chronic kidney disease, including people with and without diabetes and people with and without albuminuria. The results from these trials and meta-analysis of the totality of the evidence greatly expanded our understanding of the uses of SGLT-2 inhibitors and have increased the strength of evidence on which to make recommendations for use.

The guideline retains its previous modular format with individual sections considering evidence in people with and without diabetes separately. For this Executive Summary, however, we provide a simpler quick reference list of the types of individuals with CKD who are likely to benefit from SGLT-2 inhibitors.

We make grade 1 recommendations for use to slow kidney disease progression (or rate of kidney function decline) or to reduce cardiovascular risk. These include the following groups irrespective of diabetes status*:

(a) eGFR of 20-45 ml/min/$1.73m^2$
(b) eGFR of >45 ml/min/$1.73m^2$ & a urinary albumin-to-creatinine ratio (uACR) of ≥25 mg/mmol†
(c) Symptomatic heart failure, irrespective of ejection fraction

* excludes those with polycystic kidney disease, type 1 diabetes, or a kidney transplant;
† urinary protein-to-creatinine ratio of 35 mg/mmol can be considered equivalent.

We recommend initiating SGLT-2 inhibition in people with type 2 diabetes and established coronary disease (if eGFR ≥20 ml/min/$1.73m^2$), and that SGLT-2 inhibition can be continued until the need for dialysis or kidney transplantation arises.

We make grade 2 recommendations to suggest/consider treating the following groups:
(a) eGFR <20 ml/min/$1.73m^2$ to slow progression of kidney disease
(b) Type 2 diabetes, an eGFR 45-60 ml/min/$1.73m^2$ and a uACR of <25 mg/mmol

We also provide a summary of current indications for use of SGLT-2 inhibitors in the UK. We do suggest the kidney and cardiovascular benefits increasingly resemble a class effect. To support implementation we provide a quick reference guide for implementation (Table 1, below), template patient information leaflets, and a full lay summary of the guideline.

We would like to thank the working group for all their contributions.

Associate Prof. Will Herrington   Dr Andrew Frankel (co-chairs)

Working Group members: Dr Alexa Wonnacott, Dr David Webb, Mrs Angela Watt, Mr Michael Watson, Assoc. Prof. Natalie Staplin, Dr Alistair Roddick, Dr Alex Riding, Dr Eirini Lioudaki, Dr Apexa Kuverji, Prof. Mohsen El Kossi, Dr Patrick Holmes, Mr Matt Holloway, Prof. Donald Fraser, Dr Chris Carvalho, Prof. James Burton, Prof. Sunil Bhandari

TABLE 1: QUICK REFERENCE GUIDE FOR IMPLEMENTATION IN PEOPLE WITH CKD WITH OR WITHOUT TYPE 2 DIABETES

SGLT-2 inhibition to reduce risk of kidney disease and cardiovascular outcomes*		Urinary Albumin-to-creatinine ratio (mg/mmol)	
		<25	≥25
eGFR (mL/min/1.73m^2)	≥60	†	Recommended
	≥45 <60	Suggested (in type 2 diabetes)	Recommended
	≥20 <45	Recommended	Recommended
	<20	Suggested	Suggested
	Dialysis	Not recommended‡	Not recommended‡

* People with type 1 diabetes, polycystic kidney disease, or kidney transplant excluded from the definitive trials.
† In this guideline we do not make recommendations on the use of SGLT-2 inhibition to reduce kidney disease progression for people with eGFR ≥60 mL/min/1.73m^2 and uACR <25 mg/mmol as this is outside the scope of this guideline. However, we support the use of SGLT-2 inhibitors in this population for relevant indications, including treatment of people with heart failure and reduction of cardiovascular risk in people with type 2 diabetes at high cardiovascular risk.
‡ We recommend further research in people on kidney replacement therapy to establish the role of SGLT-2 inhibition in these populations.

Summary of recommendations

RECOMMENDATIONS FOR USE		
**Section 2**	**PEOPLE WITH TYPE 2 DM**	**Grade**
**1.**	We recommend initiating SGLT-2 inhibition in people with chronic kidney disease and type 2 diabetes, irrespective of primary kidney disease,* for any of the following 4 clinical scenarios:<br>a) eGFR of 20-45 mL/min/1.73m^2<br>b) eGFR of >45 mL/min/1.73m^2 and a urinary albumin-to-creatinine ratio (uACR) of ≥25 mg/mmol†<br>c) Symptomatic heart failure, irrespective of ejection fraction<br>d) Established coronary disease	**1A**
**2.**	We suggest initiating SGLT-2 inhibition to modify cardiovascular risk and slow rate of kidney function decline in people with an eGFR >45-60 mL/min/1.73m^2 and a uACR of <25 mg/mmol, recognising effects on glycaemic control will be limited.	**2B**
**3.**	We suggest clinicians consider initiating SGLT-2 inhibition in people with an eGFR below 20 mL/min/1.73m^2 to slow progression of kidney disease.	**2B**
**Section 3**	**PEOPLE WITHOUT DM**	
**1.**	We recommend initiating SGLT-2 inhibition in people with chronic kidney disease, irrespective of primary kidney disease,* for any of the following clinical scenarios:<br>(a) eGFR of ≥20 mL/min/1.73m^2 and a urinary albumin-to-creatinine ratio (uACR) of ≥25 mg/mmol†<br>(b) Symptomatic heart failure, irrespective of ejection fraction	**1A**
**2.**	We recommend initiating SGLT-2 inhibition to slow rate of kidney function decline in people with an eGFR of 20-45 mL/min/1.73m^2 and a uACR of <25 mg/mmol†.	**1B**
**3.**	We suggest clinicians consider initiating SGLT-2 inhibition in people with an eGFR below 20 mL/min/1.73m^2 to slow progression of kidney disease.	**2B**
** excludes people with polycystic kidney disease, type 1 diabetes, or a kidney transplant*<br>*† urinary protein-to-creatinine ratio of 35 mg/mmol can be considered equivalent*		

RECOMMENDATIONS FOR IMPLEMENTATION		
**Sections 2 & 3**	**PEOPLE WITH OR WITHOUT DM (excluding TYPE 1)**	**Grade**
**1.**	We recommend using SGLT-2 inhibitors with demonstrated efficacy for their given indications.	**1A**
**2.**	We recommend using clinically appropriate single agent RAS blockade in combination with SGLT-2 inhibition, wherever RAS blockade is indicated and tolerated.	**1A**
**3.**	We suggest following NICE guidelines on use of uACR for screening of albuminuria (NICE NG203). We recognise that more pragmatic approaches to identifying risk of kidney disease progression may be necessary whilst local access to uACR measurement is improved.	**2C**
**4.**	We recommend that SGLT-2 inhibition can be continued until the need for dialysis or kidney transplantation arises.	**1A**
**5.**	We suggest that co-prescription of SGLT-2 inhibition with MRA can be considered, where each are individually indicated.	**2B**

**6.**	We suggest the beneficial effects of SGLT-2 inhibition on kidney disease progression or risk of heart failure hospitalisation are likely to be a class effect.	**2B**
**Section 5a**	**DIABETIC KETOACIDOSIS**	**Grade**
**1.**	We recommend that people with type 1 DM should only have SGLT-2 inhibitors initiated under the strict direction of the diabetes team.	**1C**
**2.**	We recommend that people with type 2 DM at greater risk of DKA (defined in Table 5a.1) should have SGLT-2 inhibitors initiated with caution after discussion with the diabetes team.	**1C**
**3.**	We recommend SGLT-2 inhibitors are discontinued when an individual develops DKA.	**1A**
**4.**	We suggest that after an episode of DKA and where a clear contributing factor has been identified, there should be discussion with the person and clinical team to establish whether the benefits of re-introducing an SGLT-2 inhibitor outweigh the risks.	**2D**
**5.**	When initiating SGLT-2 inhibitors, we suggest that individuals should be advised on the signs and symptoms of DKA and be instructed to temporarily withhold SGLT-2 inhibitors and to seek immediate medical advice if symptoms develop.	**1C**
**6.**	We recommend always offering advice on sick day guidance when initiating SGLT-2 inhibitors and reminding them of this at every medication review.	**1C**
**7.**	We suggest that individuals taking SGLT-2 inhibitors should be advised against following a ketogenic diet.	**2C**
**8.**	We suggest that for people who choose to intermittently fast (e.g. for Ramadan), and particularly for those who are elderly, on diuretics or have CKD, consider withholding SGLT-2 inhibitors for the duration of the fasting period and for those people with diabetes ketone testing should be undertaken if unwell.	**2D**
**Section 5b**	**HYPOGLYCAEMIA**	**Grade**
**1.**	We recommend considering reducing the dose of insulin/SUs/meglitinides when initiating SGLT-2 inhibitors to reduce the risk of hypoglycaemia.	**1C**
**2.**	We recommend that when initiating SGLT-2 inhibitors in people taking SUs (e.g. gliclazide) or meglitinides (e.g. repaglinide) when the HbA1c <58 mmol/mol AND eGFR >45 mL/min/1.73m^2, consider reducing dose of SU or meglitinide by 50% to reduce risk of hypoglycaemia.	**1C**
**3.**	We recommend that when starting SGLT-2 inhibitors in people taking insulin when the HbA1c <58 mmol/mol AND eGFR >45 mL/min/1.73m^2, consider reducing the insulin dose by 20% to avoid hypoglycaemia.	**1C**
**4.**	We recommend that when starting SGLT-2 inhibitors in people taking only metformin ± pioglitazone ± DPP-4i/gliptins or GLP-1RA therapy, no dosage adjustment is necessary.	**1C**
**Section 5c**	**ACUTE KIDNEY INJURY, HYPOVOLAEMIA AND POTASSIUM**	**Grade**
**1.**	We recommend that individuals initiated on an SGLT-2 inhibitor do not routinely require an early assessment of kidney function or serum potassium following initiation of treatment.	**1C**

**2.**	We suggest that if an individual has a kidney function assessment within the first few weeks post initiation of an SGLT-2 inhibitor, a decline in eGFR needs to be interpreted with caution and in the context of an expected drug effect to avoid unwarranted discontinuation of treatment.	**2B**
**3.**	We suggest that individuals on diuretics are counselled on the symptoms of hypovolaemia and advised to seek medical attention if they develop any such symptoms after starting SGLT-2 inhibition.	**2B**
**4.**	We suggest that clinicians consider an early clinical review and if appropriate a diuretic or antihypertensive dose reduction in individuals they consider at high risk of hypovolaemia.	**2C**
**5.**	We recommend that SGLT-2 inhibitors are temporarily withheld during acute illness (see sick-day guidance in section 5a.1.2).	**1C**
**Section 5d**	**PERIPHERAL VASCULAR DISEASE AND AMPUTATION RISK**	**Grade**
**1.**	We suggest avoiding initiation of SGLT-2 inhibitors in the presence of active foot disease (infection, ulceration and ischaemia) and withholding treatment in those who develop foot complications whilst taking an SGLT-2 inhibitor.	**2B**
**2.**	We suggest a shared decision-making approach, with appropriate counselling on risks and benefits of treatment and the importance of routine preventative foot care measures for:<br>• Individuals at high risk of amputation (previous amputations, existing PVD, peripheral neuropathy)<br>• Re-initiation of SGLT-2 inhibitors after treatment and satisfactory resolution of a foot complication that occurred whist taking SGLT-2 inhibitors.	**2B**
**Section 5e**	**FRACTURE RISK**	**Grade**
**1.**	In people with CKD treated with SGLT-2 inhibitors, we suggest monitoring of bone parameters including calcium, phosphate and PTH should be performed as appropriate for CKD stage (see NICE NG203).	**2D**
**Section 5f**	**MULTIMORBIDITY AND FRAILTY**	**Grade**
**1.**	We suggest an approach to care that takes account of frailty and multimorbidity where these apply. This can include:<br>• Establishing the person's goals, values and priorities<br>• Consideration of the balance of disease and treatment burden (for example, prognostic benefits in people with limited life expectancy or frailty)<br>• Agreeing an individualised management plan.	**2D**
**Section 5g**	**MYCOTIC GENITAL INFECTIONS AND FOURNIER'S GANGRENE**	**Grade**
**1.**	We recommend that all people are counselled on the risks of mycotic genital infections prior to initiation of SGLT-2 inhibitors.	**1D**
**2.**	We recommend that all people are counselled on self-care to maintain good genital hygiene.	**1C**
**3.**	We recommend that all people are counselled on the symptoms of mycotic genital infections and how to seek help including self-management.	**1D**
**4.**	We suggest that for those individuals with a history of recurrent mycotic genital infections on SGLT-2 inhibition, consideration is given to offering prophylactic anti-fungal treatment, which should be reviewed after 6 months of therapy or earlier if clinically indicated.	**2D**
**5.**	We suggest that SGLT-2 inhibitor therapy can be continued during the treatment of mycotic genital infections.	**2D**

**6.**	We highlight the specific MHRA warning and suggest that all people are counselled on the symptoms of Fournier's gangrene and advised to stop SGLT-2 inhibitors and to seek urgent help if they develop such symptoms.	**2D**
**Section 5h**	**URINARY TRACT INFECTION**	**Grade**
**1.**	We recommend temporary discontinuation of SGLT-2 inhibitors when treating acute pyelonephritis or urosepsis (see sick-day guidance in section 5a.1.2).	**1C**
**Section 5i**	**CHILDREN, PREGNANCY AND BREASTFEEDING**	**Grade**
**1**	We suggest that all women of child-bearing potential are counselled, prior to conception, on the risks of SGLT-2 inhibitors during pregnancy.	**2D**
**2.**	We suggest SGLT-2 inhibitor therapy is discontinued upon planning, suspicion or confirmation of pregnancy.	**2D**
**3.**	We suggest SGLT-2 inhibitors are not used in women who are breastfeeding.	**2D**
**Section 7a**	**PEOPLE WITH TYPE 1 DM**	**Grade**
**1.**	We recommend that SGLT-2 inhibitors be initiated in people with type 1 DM, only under the strict direction of the diabetes team.	**1C**
**2.**	We suggest considering referring people with type 1 DM to the specialist diabetes team, for consideration of an SGLT-2 inhibitor, if they have an eGFR ≥20 mL/min/1.73m^2, and a uACR ≥25 mg/mmol despite being on maximum tolerated ACEi/ARB.	**2C**
**3.**	We recommend all people with type 1 DM started on SGLT-2 inhibitors be provided with ketone monitoring, be advised on the signs and symptoms of DKA and to seek immediate medical advice if any of these symptoms develop or ketone levels are >0.6 mmol/L.	**1B**
**Section 7b**	**KIDNEY TRANSPLANT RECIPIENTS**	**Grade**
**1.**	There is currently insufficient evidence on safety and efficacy to provide recommendations for use of SGLT-2 inhibition in people with a functioning kidney transplant.	**-**
**2.**	Any use of SGLT-2 inhibition to treat diabetes mellitus in a kidney transplant recipient should be evaluated by multi-disciplinary discussion.	**2D**
**Section 7c**	**ACUTE DECOMPENSATED HEART FAILURE**	**Grade**
**1.**	We suggest initiating SGLT-2 inhibition in people with CKD (eGFR ≥20 mL/min/1.73m^2) with acute decompensated heart failure.	**2B**

Key: Green: Recommendations with Grade 1 evidence ("we recommend" statements); Yellow: Recommendations with Grade 2 evidence ("we suggest" statements). Further details on grading can be found in Section 1, Table 1.2.

- NICE CKD guidance is available at www.nice.org.uk/guidance/ng203
- For sick day rules also see section 6's template Patient Information Leaflets.

Table abbreviations:

ACEi	Angiotensin-Converting Enzyme Inhibitor
ARB	Angiotensin-II Receptor Blocker
CKD	Chronic Kidney Disease
DKA	Diabetic Ketoacidosis
DM	Diabetes Mellitus
DPP-4i	Dipeptidyl Peptidase-4 inhibitors
eGFR	Estimated Glomerular Filtration Rate (mL/min/1.73m^2)
GLP-1RA	Glucagon-Like Peptide-1 Receptor Agonist
HbA1c	Glycosylated Haemoglobin
HFpEF	Heart Failure with Preserved Ejection Fraction
LVEF	Left Ventricular Ejection Fraction
MHRA	Medicines and Healthcare products Regulatory Agency
MRA	Mineralocorticoid Receptor Antagonist
NICE	National Institute for Health and Care Excellence
RAS	Renin Angiotensin System
SGLT-2	Sodium-Glucose Co-transporter-2
SU	Sulphonylurea
uACR	Urinary Albumin-to-Creatinine Ratio

List of abbreviations

Abbreviation	Definition
ABCD-RA	Association of British Clinical Diabetologists-Renal Association
ACEi	Angiotensin-Converting Enzyme Inhibitor
ADA	American Diabetes Association
ADHF	Acute decompensated heart failure
AF	Atrial Fibrillation
AKI	Acute Kidney Injury
ANCA	Anti-Neutrophil Cytoplasmic Antibody
ARB	Angiotensin-II Receptor Blocker
BMI	Body Mass Index
BP	Blood Pressure
CKD	Chronic Kidney Disease
CKD-MBD	Chronic Kidney Disease-Mineral Bone Disease
95%CI	95% Confidence Interval
CrI	Credible Interval
CV	Cardiovascular
DKA	Diabetic Ketoacidosis
DM	Diabetes Mellitus
DPP-4i	Dipeptidyl Peptidase-4 inhibitors
eGFR	Estimated Glomerular Filtration Rate (mL/min/1.73m^2)
EASD	European Association for the Study of Diabetes
ESKD	End-Stage Kidney Disease
EU	European Union
FDA	US Food and Drug Administration
FGF-23	Fibroblast Growth Factor-23
GFR	Glomerular Filtration Rate
GLP-1	Glucagon-Like Peptide-1
GLP-1RA	Glucagon-Like Peptide-1 Receptor Agonist
HbA1c	Glycosylated Haemoglobin
HF	Heart Failure
HFpEF	Heart Failure with Preserved Ejection Fraction
HFrEF	Heart Failure with Reduced Ejection Fraction
HHF	Hospitalisation for Heart Failure
HR	Hazard Ratio
ICER	Incremental Cost-effectiveness Ratio
KCCQ-TSS	Kansas City Cardiomyopathy Questionnaire Total Symptom Score
KDIGO	Kidney Disease Improving Global Outcomes
LVEF	Left Ventricular Ejection Fraction
MACE	Major Adverse/Atherosclerotic Cardiovascular Event
MedDRA	Medical Dictionary for Regulatory Activities
MHRA	Medicines and Healthcare products Regulatory Agency
MRA	Mineralocorticoid Receptor Antagonist
NHS	National Health Service
NICE	National Institute for Health and Care Excellence
NNH	Number Needed to Harm
NNT	Number Needed to Treat
NODAT	New Onset Diabetes After Transplantation

NT-proBNP	N-Terminal Pro-Brain Natriuretic Peptide
OR	Odds Ratio
PADP	Pulmonary Artery Diastolic Pressure
PTH	Parathyroid Hormone
Q1-Q3	Quartile 1-Quartile 3 (i.e. the interquartile cutoffs)
QALY	Quality-Adjusted Life Years
RAS	Renin Angiotensin System
RCT	Randomized Controlled Trials
RR	Relative Risk
RSG	Renal Studies Group
SD	Standard Deviation
SE	Standard Error
SGLT-2	Sodium-Glucose Co-transporter-2
SMART-C	SGLT-2 inhibitor Meta-Analysis Cardio-Renal Trialists' Consortium
SmPC	Summary of Product Characteristics
SU	Sulphonylurea
uACR	Urinary Albumin-to-Creatinine Ratio
UK	United Kingdom
UTI	Urinary Tract infection

Section 1: Background, aims and concise methods

1.1 Summary

Large-scale placebo-controlled trials have demonstrated that sodium glucose co-transporter-2 (SGLT-2) inhibition favourably modifies both risks of cardiovascular disease and kidney disease progression in a range of at-risk populations, including people with chronic kidney disease (CKD). This section provides the background to this guideline by introducing: (i) CKD and the concept of intraglomerular hypertension; (ii) the molecular mechanisms of SGLT-2 inhibition; and (iii) the large placebo-controlled trials that have informed us of its cardio-renal beneficial effects.

1.2 Introduction

1.2.1 CKD is common and associated with risk of progression to kidney replacement therapy

The age-standardised prevalence of CKD in adults in the UK is estimated to be about 6-11% (1). In the absence of effective new interventions, this proportion is predicted to rise as the population ages, premature mortality from cardiovascular and other causes declines further, and type 2 diabetes mellitus (DM) becomes more prevalent (2). Worldwide, diabetic kidney disease accounts for 30-50% of advanced CKD (i.e. stages 4-5) (3, 4). In the UK, currently about 30% of those starting maintenance kidney replacement therapy have diabetic nephropathy as their primary kidney disease, peaking at 38% among those starting at the ages of 55-64 years (5).

CKD can be a progressive condition, with albuminuria representing a significant risk factor for more rapid kidney function decline both in people with and without diabetes (6). The avoidance of progressive CKD is important as end-stage kidney disease (ESKD) has adverse effects on morbidity and quality of life, dialysis or transplantation incur substantial societal costs (7, 8), and low levels of kidney function increase cardiovascular risk (9).

Albuminuria is a marker of intraglomerular hypertension and has been used as a means to select participants at high risk of kidney disease progression into CKD trials. Such trials have often studied diabetic nephropathy separately from other causes of CKD. For example, pharmacological inhibition of the renin-angiotensin system (RAS) reduces efferent arteriolar tone and hence intraglomerular pressure, and large trials have shown this reduces albuminuria and the risk of overt diabetic nephropathy progressing to ESKD (10, 11). However, intraglomerular hypertension is also considered to be a common pathway for kidney disease progression shared by some non-diabetic forms of CKD (12). The concept centres on the idea that reduced nephron numbers induces hyperfiltration in the remaining glomeruli. Support for this concept includes the observations that: (i) for a given level of urinary albumin excretion, the risk of ESKD is relatively independent of the primary kidney diagnosis (13); and (ii) trial meta-analyses show that RAS-inhibition slows progression of a range of proteinuric non-diabetic kidney diseases (14, 15). Single agent RAS-inhibition is therefore the standard of care for proteinuric CKD.

Nevertheless, despite use of appropriate RAS-inhibition alongside suitably intensive glycaemic (16-18) and blood pressure (19-22) control, substantial residual risk of ESKD remains in people with proteinuric CKD (10, 11).

1.2.2 People with CKD are at high risk of structural heart disease and heart failure

In cohorts with appropriate cardiac imaging, structural heart disease is identified in about one-half of people with CKD stage 4-5 (23). CKD often co-exists with heart failure, due to a combination of shared risk factors and integrated pathophysiology (24). This may more commonly present as heart failure with preserved ejection fraction (HFpEF). Many people with CKD consequently die from cardiovascular disease before progression to ESKD. Management of CKD therefore includes modification of risk of both kidney disease progression and associated cardiovascular risk (24, 25). SGLT-2 inhibitors have emerged as a potential therapy to address both the cardiac complications of CKD and risk of kidney disease progression (26-39).

1.3 Molecular mechanisms of action of SGLT-2 inhibition

Everyone has a threshold of renal tubular glucose concentration above which glucose appears in the urine (40). It was recognised that this threshold could be reduced with an apple tree bark extract called phlorizin (41, 42). Following the cloning of the SGLT-1 and SGLT-2 genes, phlorizin was characterised as a non-specific SGLT inhibitor. SGLT-1 is a low-capacity, high-affinity transporter located primarily in the gastrointestinal tract. It functions to absorb dietary glucose and is also expressed in the later renal proximal tubule segment (S3), where it is responsible for reabsorbing only ~3% of filtered glucose under normal physiological conditions. By contrast, SGLT-2 is a high-capacity, low-affinity transporter located mainly in the early renal proximal tubule segments. SGLT-2 is responsible for reabsorbing ~97% of filtered glucose. Inhibition of SGLT-2 therefore has a much larger effect on the glucose threshold than SGLT-1 inhibition. Dual SGLT-1/2 inhibitors have also been developed with the aim of increasing urinary glucose-lowering excretion because SGLT-1 has significant reserve capacity to reabsorb glucose if SGLT-2 is inhibited (43-45), but gut effects include a potential to cause diarrhoea (34, 36).

For each SGLT-2 reabsorbed molecule of glucose, a sodium ion is co-transported. SGLT-2 inhibition therefore increases sodium delivery through the renal tubules to each nephron's macula densa, and subsequently into the urine. The macula densa is the structural area of the early distal convoluted tubule which lies between the afferent and efferent arteriole of the mother glomerulus to that distal tubule. Sodium delivery to the macula densa results in changes to intraglomerular blood flow and pressure by means of changes to the calibre of the afferent arteriole, a homeostatic process referred to as tubuloglomerular feedback. High sodium delivery to the macula densa results in constriction of the afferent arteriole, which results in a decrease in glomerular blood flow and glomerular capillary pressure, whilst decreased sodium delivery has the opposite effect. Therefore, SGLT-2 inhibition augments macula densa tubuloglomerular feedback as well as generating a natriuresis to combine with the glycosuric osmotic diuresis. These sodium effects appear to be central to both the kidney and cardiovascular physiological effects of SGLT-2 inhibition.

1.3.1 SGLT-2 inhibitors' glycaemic effects

SGLT-2 inhibitors (also known as "gliflozins") were initially assessed and licenced for their glucose-lowering potential (46). However, the glycosuric effect of SGLT-2 inhibition linearly attenuates as kidney function declines, and so such licences were initially restricted to people with generally preserved kidney function. These preclusions for use in people with CKD have been progressively relaxed following the results of several large trials (section 4 of this guideline provides a summary of current licencing in CKD).

Large FDA-mandated trials in type 2 DM populations were initiated in order to assess the cardiovascular safety of SGLT-2 inhibitors (47). These trials demonstrated that SGLT-2 inhibitors are non-inferior to placebo with respect to effects on major atherosclerotic (or adverse) cardiovascular events (MACE) with some trials also demonstrating superiority (26-28). Subsequently, the CREDENCE, DAPA-CKD and EMPA-KIDNEY trials have demonstrated SGLT-2 inhibition's particular efficacy at reducing risk of kidney disease progression and heart failure hospitalisation in people with CKD down to an estimated glomerular filtration rate (eGFR) of 20 mL/min/1.73m^2 (29, 35, 39, 48). The realisation from these trials that SGLT-2 inhibition confers cardiac and kidney protection independent of glycaemic effects and kidney function, with substantial benefits also evident in people without DM, has led to a shift in focus from purely lowering glycosylated haemoglobin (HbA1c) to disease risk modification. At the time of writing, there are thirteen reported large placebo-controlled trials in different settings, including trials in populations with type 2 DM, heart failure and CKD (see Table 1.1 for a listing, including key eligibility criteria and outcomes).

1.3.2 SGLT-2 inhibitors' effects on kidney physiology

In people with CKD, the CREDENCE, DAPA-CKD and EMPA-KIDNEY trials have demonstrated SGLT-2 inhibition's particular efficacy in reducing risk of kidney disease progression in people with type 2 DM and albuminuric diabetic kidney disease (29, 35, 48). Analyses of DAPA-CKD and EMPA-KIDNEY have shown that benefits extend to non-diabetic causes of CKD (39, 48, 49). Despite the attenuated ability of SGLT-2 inhibitors to lower glucose at reduced levels of kidney function, the relative benefits of SGLT-2 inhibition on kidney disease progression appear preserved in people without DM, and no threshold has been identified where relative benefits start to attenuate (29, 35, 39, 48, 49).

As described above, the key mechanism for renoprotection is considered to be through SGLT-2 inhibitor's modulation of tubuloglomerular feedback, increased delivery of sodium to the macula densa and the enhancement of glomerular afferent arteriolar vasoconstriction (50, 51). The consequent reduction in kidney glomerular blood flow is believed to be responsible for the acute, reversible dip in kidney function, reductions in albuminuria, and slowing of kidney function decline following initiation of SGLT-2 inhibition (50). This modulation of kidney haemodynamics appears to persist in people with normoglycaemia (although it may be attenuated). Acute dips in eGFR on initiation of SGLT-2 inhibition are apparent in people without DM (52-54). Consequently, SGLT-2 inhibition is hypothesised to reduce intraglomerular hypertension and target the proposed final common pathway for kidney disease progression in people with or without DM (55).

SGLT-2 inhibition also reduces the risk of adverse events attributed to acute kidney injury (AKI) (49, 56), with a protective effect evident in the trials of people with type 2 DM (32, 56), heart failure (30, 34, 57) and CKD populations alike (29, 35, 36). The proposed protective mechanisms are reduced risk of ischaemic-

reperfusion injury or renal tubular hypoxia from the lowered metabolic demand of inhibited co-transporters (58). Conceivably, a reduction in AKI risk may also translate into benefits on CKD progression, providing a mechanistic explanation for suggested beneficial effect of SGLT-2 inhibition on estimated glomerular filtration rate slopes in individuals with heart failure and without albuminuria (33, 57, 59, 60).

Establishing definitively whether or not albuminuria is a pre-requisite for kidney benefits of SGLT-2 inhibition was an important question to address as: (i) the majority of individuals with CKD do not have albuminuria, and (ii) if mechanistic theories about intraglomerular hypertension are correct, kidney benefits may be substantially different in the absence of albuminuria. The EMPA-KIDNEY trial, which included participants with a wide range of kidney function including people with CKD without albuminuria, demonstrated that level of albuminuria modified the effect of SGLT-2 inhibition on kidney disease progression, with large relative benefits in people with higher levels of albuminuria. However, the number of participants without albuminuria whose kidney disease progressed to at least a 40% decline in eGFR during the 2 years of follow-up was low, and exploratory analyses of eGFR slopes were required to assess the effects in this subgroup (39). These analyses demonstrated that the rate of kidney function decline was reduced by a meaningful degree in people both with and without albuminuria, and that longer treatment periods would be expected to lead to reductions in the risk of kidney failure.

Sections 2 and 3 of this guideline will provide a more detailed appraisal of trials assessing the effects of SGLT-2 inhibition on kidney disease, with section 7 also providing special consideration for people with type 1 DM, acute decompensated heart failure, or a functioning kidney transplant.

1.3.3 SGLT-2 inhibitors' effects on cardiovascular physiology

The totality of the trial evidence shows that relative benefits of SGLT-2 inhibition on heart failure outcomes are both larger and more consistent than on MACE. Meta-analyses estimated that the risk of hospitalisation for heart failure was reduced by about one-third compared to placebo, and MACE risk is reduced by about 10% (61). This suggests that the natriuretic, osmotic diuretic and renoprotective effects of SGLT-2 inhibition are more effective at targeting heart failure pathophysiology than atherothrombotic risk. The effects of SGLT-2 inhibition on MACE risk may result from the more modest lowering effects of SGLT-2 inhibition on blood pressure, HbA1c and adiposity. For a more detailed review of the cardiac effects of SGLT-2 inhibition, see a 2021 update from the European Society of Cardiology ad-hoc task force (62).

1.3.4 SGLT-2 inhibitors' effects on metabolism

SGLT-2 inhibitors have broad metabolic effects beyond lowering blood glucose. Glycosuria leads to increased plasma glucagon, which in turn increases hepatic glucose production in part by glycogenolysis (63, 64). Depletion of liver glycogen creates a fasting-like state initiating ketone generation from the liver as an alternative energy source (52). Randomised trials consistently show a dose-dependent reduction in weight (65). Whereas the early weight loss may be due to intra- and extra-vascular volume depletion (66), loss of adipose tissue does occur with longer-term treatment (67).

In addition to inducing a state of ketosis, SGLT-2 inhibition also reduces renal ammoniagenesis. This – in combination with ketosis – leads to urinary loss of bicarbonate which, combined with ketosis, may lower the threshold required to induce ketoacidosis in the presence of an additional insult (e.g. fasting or infection)

(68). This accounts for both the increased risk of ketoacidosis in individuals taking SGLT-2 inhibitors and explains presentations of "euglycaemic ketoacidosis". Ketoacidosis is a particular risk in individuals who have limited endogenous insulin production, and particularly those with type 1 DM (69). The benefit-risk ratio is therefore more finely balanced in people with type 1 DM. While use of certain SGLT-2 inhibitors has previously been granted by regulators at lower doses and under specialist supervision in this population (70, 71), authorisation for two SGLT-2 inhibitors have withdrawn from their indication for type 1 DM (72, 73). Section 7 of this guideline document introduces special consideration for people with type 1 DM.

The risk of severe hypoglycaemia caused by SGLT-2 inhibition is small (26-30, 32-39). Mechanistically, hypoglycaemia would not be expected because of the compensatory effects of intact SGLT-1 activity and hepatic gluconeogenesis (74). Hypoglycaemia on SGLT-2 inhibitors is therefore largely limited to individuals who are on concomitant hypoglycaemia-inducing medication (i.e. insulin or insulin secretagogues).

1.3.5 Potential adverse effects of SGLT-2 inhibitors

Mycotic genital infections are common in individuals with DM, but there is an increased risk associated with SGLT-2 inhibitor-induced glycosuria. The effect of SGLT-2 inhibition on these infections is large enough to have been apparent in the earlier smaller trials focusing on glycaemic control (75-77). Case reports of necrotizing fasciitis of the perineum (Fournier's gangrene) attribute such devastating polymicrobial infections to SGLT-2 inhibitor use (78), but the rarity of the condition means there is insufficient randomised data to confirm or refute this hazard – a causal association remains unproven (28, 32, 35, 79). Large amounts of urinary glucose meant an increased incidence of urinary tract infection were expected and are listed in the labels for all SGLT-2 inhibitors. However, this effect is small and only apparent when results from all the large randomised trials (26-30, 32-39) are combined in meta-analysis (49, 80).

The CANVAS program, which tested canagliflozin in individuals with type 2 DM at high risk of cardiovascular disease, raised two new safety considerations: an excess of bone fractures and separately, an increased risk of lower limb amputation were identified (27). Post hoc biological rationales have been proposed for these effects of canagliflozin (81), but a chance finding is a plausible alternative explanation. Excess of lower limb amputation or bone fracture has not been observed in the other large trials of SGLT-2 inhibition, including the CREDENCE trial, which assessed canagliflozin in individuals with type 2 DM and CKD (29, 49).

Section 5 of this guideline document will provide a more detailed appraisal of the trials assessing the effects of SGLT-2 inhibition on metabolic and safety outcomes, with section 6 providing supporting information for people with CKD being offered SGLT-2 inhibition.

1.4 Listing of key large-scale placebo-controlled clinical outcome trials

Table 1.1: Large placebo-controlled SGLT-2 inhibitor clinical outcome trials, by population

Population<br>**Trial (reference) (drug & daily dose)**	**Size**	**Median follow-up, years**	**Proportion with type 2 DM**	**Average (SD) eGFR, mL/min/1.73m^2**	**Key eligibility criteria**	**Primary outcome(s)**	**Selected secondary and exploratory outcomes**
					Ia. Heart failure population		
DAPA-HF (30)<br>(dapagliflozin 10mg)	4744	1.5	42%	Mean:<br>66 (19)	• Symptomatic chronic HF (class II-IV) with LVEF ≤40%<br>• NT-proBNP ≥600 pg/mL<br>• eGFR ≥30<br>• Appropriate doses of medical therapy and use of medical devices	• CV death or worsening HF (hospitalisation or an urgent visit for intravenous therapy)	• CV death or hospitalisation for HF<br>• Total number of hospitalisation for HF<br>• Sustained ≥50% decline in eGFR, sustained eGFR <15, ESKD, or renal death<br>• Death from any cause
EMPEROR-REDUCED (33)<br>(empagliflozin 10mg)	3730	1.3	50%	Mean:<br>62 (22)	• Class II-IV chronic HF with LVEF ≤40%<br>• NT-proBNP above a certain threshold (stratified by LVEF)<br>• Appropriate doses of medical therapy and use of medical devices<br>• eGFR ≥20	• CV death or hospitalisation for worsening HF	• Total number of hospitalisation for HF<br>• Rate of eGFR decline<br>• Death from any cause
					Ib. Heart failure (preserved or mixed ejection fraction) population		
SOLOIST-WHF (34)<br>(sotagliflozin 200-400mg)	1222	0.8	100%	Median:<br>50	• Type 2 DM<br>• Hospitalised for heart failure requiring intravenous therapy<br>• eGFR ≥30<br>• No recent coronary event	• CV death or total number of worsening HF events (hospitalisation or an urgent visit)	• Total number of worsening HF events (hospitalisation or an urgent visit)<br>• Change in eGFR<br>• Death from any cause
DELIVER (38)<br>(dapagliflozin 10mg)	6263	2.3	45%	Mean:<br>61 (19)	• Symptomatic chronic HF (class II-IV) with LVEF >40% & structural heart disease<br>• Elevated NT-proBNP<br>• eGFR ≥25	• CV death or worsening HF (hospitalisation or an urgent visit)	• Total number of worsening HF events (hospitalisation or an urgent visit)<br>• Death from any cause

Population<br>Trial (reference)<br>(drug & daily dose)	Size	Median follow-up, years	Proportion with type 2 DM	Average (SD) eGFR, mL/min/1.73m^2	Key eligibility criteria	Primary outcome(s)	Selected secondary and exploratory outcomes
EMPEROR-PRESERVED (37) (empagliflozin 10mg)	5988	2.2	49%	Mean:<br>61 (20)	• Symptomatic chronic HF (class II-IV) with LVEF >40% & structural heart disease<br>• NT-proBNP >300 pg/mL (or >900 if in AF)<br>• eGFR ≥20	• CV death or hospitalisation for HF	• eGFR slope<br>• ESKD<br>• All-cause hospitalisation<br>• Death from any cause
II. High cardiovascular risk + type 2 DM population							
EMPA-REG OUTCOME (26)(empagliflozin 10mg or 25mg)	7020	3.1	100%	Mean:<br>74 (21)	• Type 2 DM<br>• History of coronary, cerebral or peripheral vascular disease<br>• eGFR ≥30	• CV death, non-fatal myocardial infarction or non-fatal stroke	• Hospitalisation for HF<br>• Incident or worsening nephropathy: macroalbuminuria, a doubling of the serum creatinine (accompanied by an eGFR of ≤45), ESKD or renal death
CANVAS Program (27) (canagliflozin 100-300mg)	10142	2.4	100%	Mean:<br>76 (20)	• Type 2 DM<br>• History of coronary, cerebral or peripheral vascular disease OR age >50y with at least 2 CV risk factors<br>• eGFR ≥30	• CV death, non-fatal myocardial infarction or non-fatal stroke	• CV death or hospitalisation for HF<br>• 30% increase in albuminuria with change in category<br>• Death from any cause
DECLARE-TIMI 58 (28)(dapagliflozin 10mg)	17160	4.2	100%	Mean:<br>85 (16)	• Type 2 DM<br>• Age 40y + history of coronary, cerebral or peripheral vascular disease OR age ≥55y in men/≥60y in women with at least 1 CV risk factors<br>• Creatinine clearance ≥60 mL/min	Co-primaries<br>• CV death, myocardial infarction or ischaemic stroke<br>• CV death or hospitalisation for worsening HF	• Sustained ≥40% decline in eGFR (to <60), ESKD, or death from kidney or CV causes<br>• Death from any cause
VERTIS CV (32) (ertugliflozin 5 or 15 mg)	8246	3.0	100%	Mean:<br>76 (21)	• Type 2 DM<br>• History of coronary, cerebral or peripheral vascular disease<br>• eGFR ≥30	• CV death, non-fatal myocardial infarction or non-fatal stroke	• Hospitalisation for HF<br>• Doubling of the serum creatinine, ESKD, or renal death

Population Trial (reference) (drug & daily dose)	Size	Median follow-up, years	Proportion with type 2 DM	Average (SD) eGFR, mL/min/1.73m^2	Key eligibility criteria	Primary outcome(s)	Selected secondary and exploratory outcomes
					III. Chronic kidney disease population		
CREDENCE (29) (canagliflozin 100mg)	4401	2.6	100%	Mean: 56 (18)	• Type 2 DM<br>• eGFR 30-90<br>• uACR 300-5000 mg/g<br>• Stable maximally tolerated RAS blockade	• Sustained doubling of creatinine, sustained eGFR <15, ESKD, or death from renal or CV causes	• Hospitalisation for HF<br>• CV death, non-fatal myocardial infarction or non-fatal stroke<br>• Death from any cause
DAPA-CKD (35) (dapagliflozin 10mg)	4304	2.4	68%	Mean: 43 (12)	• eGFR 25-75<br>• uACR 200-5000 mg/g<br>• Stable maximally tolerated RAS blockade, unless documented intolerance	• Sustained ≥50% decline in eGFR, sustained eGFR <15, ESKD, or death from renal or CV causes	• Hospitalisation for HF<br>• Death from any cause
SCORED (36) (sotagliflozin 200-400mg)	10584	1.3	100%	Median: 45	• Type 2 DM<br>• eGFR 25-60<br>• At least 1 CV risk factor	• CV death or total number of worsening HF events (hospitalisation or an urgent visit)	• CV death, non-fatal myocardial infarction or non-fatal stroke<br>• Sustained ≥50% decline in eGFR, sustained eGFR <15, or ESKD<br>• Death from any cause
EMPA-KIDNEY (39) (empagliflozin 10mg)	6609	2.0	46%	Mean: 37 (14)	• eGFR 20-45, or eGFR 45-90 with uACR ≥200 mg/g<br>• Clinically appropriate doses of RAS blockade, unless not tolerated	• Sustained ≥40% decline in eGFR, sustained eGFR <10, ESKD, or death from renal or CV causes	• CV death or hospitalisation for HF<br>• All-cause hospitalisation<br>• Death from any cause

Footnote: AF=atrial fibrillation; CKD=chronic kidney disease; CV=cardiovascular; DM=diabetes mellitus; eGFR=estimated glomerular filtration rate (mL/min/1.73m^2); ESKD=end-stage kidney disease (i.e. maintenance dialysis or receipt of kidney transplant); HF=heart failure; LVEF=left ventricular ejection fraction; RAS=renin angiotensin system; uACR=urinary albumin-to-creatinine ratio
(Table reproduced and updated from an update from the European Society of Cardiology ad-hoc task force on sodium-glucose co-transporter-2 inhibitors (82)) A large trial in people with Type 1 DM (inTandem-3) is not tabulated but considered in section 7 (83).

1.5 Guideline aims & development

1.5.1 Aims & recommendation types

Our overriding objective is to provide practical and pragmatic clinical practice guidelines to facilitate rapid and safe use of SGLT-2 inhibitors in the context of CKD in adults. In assessing the evidence base, we have deliberately focused on the relevant large-scale randomised evidence and have respected the relevant regulatory approvals for individual SGLT-2 inhibitors. More specifically, we aimed to:

(i) Provide guidance on use of SGLT-2 inhibitors in people with CKD, focusing on the potential to modify risk of kidney disease progression; and

(ii) Support safe implementation of SGLT-2 inhibitors into clinical practice in people with CKD.

In order to support both use and implementation, we therefore provide four types of Recommendation. Recommendations for:

(i) Use (who should be offered SGLT-2 inhibition)

(ii) Implementation (how should SGLT-2 inhibition be used)

(iii) Research (what are areas of ongoing clinical uncertainty)

(iv) Audit (how effective implementation can be demonstrated)

1.5.2 Evidence grading

In general, we followed the principles set out in the UK Kidney Association's "Clinical Practice Guideline Development Manual" and grade "Recommendations for Use" and "Recommendations for Implementation" according to its two-tier grading system (see Table 1.2). We use the term "recommend" within the guideline text where Recommendations are based on Grade 1 evidence, and prefer the term "suggest" for those based on Grade 2 evidence. Recommendations for Implementation could be considered a Practice Point but we avoid using this term for clarity. Our Recommendations for Research are not graded, and we offer Audit Measures for Recommendations with Grade 1 levels of evidence.

Table 1.2: UK Kidney Association's grading system for recommendations' strength and evidence quality

Level of evidence	Evidence quality
• Grade 1 recommendation is a strong recommendation to do (or not do) something, where the benefits clearly outweigh the risks (or vice versa) for most, if not all patients (i.e. recommendations)<br><br>• Grade 2 recommendation is a weaker recommendation, where the risks and benefits are more closely balanced or are more uncertain (i.e. suggestions)	• Grade A evidence means high-quality evidence that comes from consistent results from well-performed randomised controlled trials, or overwhelming evidence of some other sort.<br>• Grade B evidence means moderate-quality evidence from randomised trials that suffer from serious flaws in conduct, inconsistency, indirectness, imprecise estimates, reporting bias, or some combination of these limitations, or from other study designs with special strength.<br>• Grade C evidence means low-quality evidence from observational studies, or from controlled trials with several very serious limitations.<br>• Grade D evidence is based only on case studies or expert opinion.

1.5.3 Guideline structure

We recognise that the use of SGLT-2 inhibition is subject to a significant amount of ongoing research and there is likely to be further evidence that may influence the recommendations made within these guidelines. As in the previous version of the guideline, we retain a modular structure to facilitate efficient revisions as the evidence base continues to expand.

Recommendations for Use are provided, separately, for individuals with type 2 DM (section 2) and people without DM (section 3). In the present update, though our recommendations for people with and without diabetes have begun to converge, we maintain this division to acknowledge the differences in available evidence in these two populations. Section 4 summarises the current licensing of SGLT-2 inhibitors to support selection of SGLT-2 inhibitors in people with CKD. Section 5 focuses on the information on safety of SGLT-2 inhibitors, including considerations for older or multi-comorbid individuals. This section provides a series of Recommendations for Implementation. Section 6 provides patients' perspectives and template Patient Information Leaflets. Lastly, section 7 provides consideration for populations of specific interest in which trial evidence is more limited currently. These populations include: (i) type 1 DM, (ii) kidney transplant recipients, and (iii) acute decompensated heart failure.

1.5.4 Evidence synthesis by systematic literature review

The generation of Recommendations was supported by a systematic literature search of relevant SGLT-2 inhibitor randomised controlled trials (see Methodological appendix for full details of the search strategy and results), which was updated ahead of this Guideline update. In brief, a search of MEDLINE and Embase bibliographic databases via OVID from 29th August 2021 to 5th September 2022 was performed and combined with the previous database search, which comprised records from database inception to 28th August 2021. Eligible trials were randomised parallel-group SGLT-2 inhibitor trials irrespective of size or duration. Trials which were placebo-controlled or offered comparisons with non-SGLT-2 inhibitor treatments (e.g. a sulfonylurea) were included, but trials randomizing participants to two different SGLT-2 inhibitors without a control group were excluded. The following other types of trials were also excluded: non-English

language reports, purely pharmacokinetic/pharmacodynamics studies (e.g. in healthy volunteers or in phase 1), and duplicates.

Trials were subcategorised into large placebo-controlled trials (i.e. those with >1000 participants randomised and with >500 participants in each arm), and into groups of specific interest relevant to specific guideline sections (i.e. type 1 DM, kidney transplant recipients, and acute decompensated heart failure). Large trials were subject to a trial quality assessment using the Cochrane Risk of Bias 2 (ROB2) tool, with main and relevant subsidiary publications reviewed. Separate searches of pooled analyses from trials, meta-analyses and registries of ongoing trials were also performed, and relevant guidelines from UK stakeholders and elsewhere (e.g. KDIGO) reviewed along with regulatory licences for SGLT-2 inhibitors. Note that the 2021 Association of British Clinical Diabetologists-Renal Association (ABCD-RA) Clinical Practice Guidelines for the Management of Hyperglycaemia in Adults with Diabetic Kidney Disease is particularly relevant to this guideline, and we cross-reference relevant sections. However, our guidance was developed independently, and is consequently not identical.

1.5.5 Guideline update methodology

In the present updated guideline, guideline sections were reviewed in light of new evidence from published trials and updated database search. Updated evidence-based recommendations were drafted by a subgroup of the Guideline Working Group and presented to the wider Guideline Working Group. Once consensus was reached among Working Group members, relevant modular sections were revised and circulated to the Working Group. All members had the opportunity to review proposed updates and final revised guidelines.

1.5.6 Terminology used in this guideline

In this guideline, terminology used to describe kidney disease and relevant outcomes has been chosen in line with KDIGO guidelines on nomenclature for kidney function and disease, which emphasises patient-centred and precise language (84). Where possible, the term ‘kidney’ has been used in preference to the term ‘renal’, and the term ‘kidney failure’ has been used in preference to ‘end-stage kidney disease’. However, where such terms are used to describe outcomes studied in clinical trials, the terms originally used by the trial authors have been preserved as presented in published trial reports.

1.6 References

1. Stevens PE, O'Donoghue DJ, de Lusignan S, Van Vlymen J, Klebe B, Middleton R, et al. Chronic kidney disease management in the United Kingdom: NEOERICA project results. Kidney Int. 2007;72(1):92-9.
2. Hoerger TJ, Simpson SA, Yarnoff BO, Pavkov ME, Rios Burrows N, Saydah SH, et al. The future burden of CKD in the United States: a simulation model for the CDC CKD Initiative. American journal of kidney diseases : the official journal of the National Kidney Foundation. 2015;65(3):403-11.
3. Jha V, Garcia-Garcia G, Iseki K, Li Z, Naicker S, Plattner B, et al. Chronic kidney disease: global dimension and perspectives. Lancet. 2013;382(9888):260-72.
4. USRDS. International Comparison. 2014 Annual Data Report: Atlas of End-Stage Renal Disease in the United States. , National Institutes of Health, National Institute of Diabetes and Digestive and Kidney Diseases, Bethesda, MD. 2014;2.
5. UK Renal Registry 22nd Annual Report. https://renal.org/about-us/who-we-are/uk-renal-registry (accessed 17th May 2021).
6. Fox CS, Matsushita K, Woodward M, Bilo HJ, Chalmers J, Heerspink HJ, et al. Associations of kidney disease measures with mortality and end-stage renal disease in individuals with and without diabetes: a meta-analysis. Lancet. 2012;380(9854):1662-73.
7. Kent S, Schlackow I, Lozano-Kuhne J, Reith C, Emberson J, Haynes R, et al. What is the impact of chronic kidney disease stage and cardiovascular disease on the annual cost of hospital care in moderate-to-severe kidney disease? BMC nephrology. 2015;16:65.
8. Kerr M, Bray B, Medcalf J, O'Donoghue DJ, Matthews B. Estimating the financial cost of chronic kidney disease to the NHS in England. Nephrology, dialysis, transplantation : official publication of the European Dialysis and Transplant Association - European Renal Association. 2012;27 Suppl 3:iii73-80.
9. Matsushita K, van der Velde M, Astor BC, Woodward M, Levey AS, de Jong PE, et al. Association of estimated glomerular filtration rate and albuminuria with all-cause and cardiovascular mortality in general population cohorts: a collaborative meta-analysis. Lancet. 2010;375(9731):2073-81.
10. Brenner BM, Cooper ME, de Zeeuw D, Keane WF, Mitch WE, Parving HH, et al. Effects of losartan on renal and cardiovascular outcomes in patients with type 2 diabetes and nephropathy. The New England journal of medicine. 2001;345(12):861-9.
11. Lewis EJ, Hunsicker LG, Clarke WR, Berl T, Pohl MA, Lewis JB, et al. Renoprotective effect of the angiotensin-receptor antagonist irbesartan in patients with nephropathy due to type 2 diabetes. The New England journal of medicine. 2001;345(12):851-60.
12. Brenner BM, Lawler EV, Mackenzie HS. The hyperfiltration theory: a paradigm shift in nephrology. Kidney Int. 1996;49(6):1774-7.
13. Haynes R, Staplin N, Emberson J, Herrington WG, Tomson C, Agodoa L, et al. Evaluating the contribution of the cause of kidney disease to prognosis in CKD: results from the Study of Heart and Renal Protection (SHARP). American journal of kidney diseases : the official journal of the National Kidney Foundation. 2014;64(1):40-8.
14. Jafar TH, Schmid CH, Landa M, Giatras I, Toto R, Remuzzi G, et al. Angiotensin-converting enzyme inhibitors and progression of nondiabetic renal disease. A meta-analysis of patient-level data. Ann Intern Med. 2001;135(2):73-87.
15. Perico N, Ruggenenti P, Remuzzi G. ACE and SGLT2 inhibitors: the future for non-diabetic and diabetic proteinuric renal disease. Current opinion in pharmacology. 2017;33:34-40.

16. Zoungas S, Arima H, Gerstein HC, Holman RR, Woodward M, Reaven P, et al. The effects of intensive glucose control on microvascular outcomes in patients with type 2 diabetes: a meta-analysis of individual participant data from randomised controlled trials. Lancet Diabetes & Endocrinololgy. 2017;5(6):431-7.

17. Herrington WG, Preiss D. Tightening our understanding of intensive glycaemic control. The Lancet Diabetes & Endocrinology. 2017;5(6):405-7.

18. de Boer IH, Group DER. Kidney disease and related findings in the diabetes control and complications trial/epidemiology of diabetes interventions and complications study. Diabetes Care. 2014;37(1):24-30.

19. Klahr S, Levey AS, Beck GJ, Caggiula AW, Hunsicker L, Kusek JW, et al. The effects of dietary protein restriction and blood-pressure control on the progression of chronic renal disease. Modification of Diet in Renal Disease Study Group. The New England journal of medicine. 1994;330(13):877-84.

20. Lv J, Ehteshami P, Sarnak MJ, Tighiouart H, Jun M, Ninomiya T, et al. Effects of intensive blood pressure lowering on the progression of chronic kidney disease: a systematic review and meta-analysis. CMAJ : Canadian Medical Association journal = journal de l'Association medicale canadienne. 2013;185(11):949-57.

21. Wright JT, Jr., Bakris G, Greene T, Agodoa LY, Appel LJ, Charleston J, et al. Effect of blood pressure lowering and antihypertensive drug class on progression of hypertensive kidney disease: results from the AASK trial. Jama. 2002;288(19):2421-31.

22. Ettehad D, Emdin CA, Kiran A, Anderson SG, Callender T, Emberson J, et al. Blood pressure lowering for prevention of cardiovascular disease and death: a systematic review and meta-analysis. Lancet. 2016;387(10022):957-67.

23. Park M, Hsu CY, Li Y, Mishra RK, Keane M, Rosas SE, et al. Associations between kidney function and subclinical cardiac abnormalities in CKD. Journal of the American Society of Nephrology : JASN. 2012;23(10):1725-34.

24. House AA, Wanner C, Sarnak MJ, Pina IL, McIntyre CW, Komenda P, et al. Heart failure in chronic kidney disease: conclusions from a Kidney Disease: Improving Global Outcomes (KDIGO) Controversies Conference. Kidney Int. 2019;95(6):1304-17.

25. Perkovic V, Agarwal R, Fioretto P, Hemmelgarn BR, Levin A, Thomas MC, et al. Management of patients with diabetes and CKD: conclusions from a "Kidney Disease: Improving Global Outcomes" (KDIGO) Controversies Conference. Kidney Int. 2016;90(6):1175-83.

26. Zinman B, Wanner C, Lachin JM, Fitchett D, Bluhmki E, Hantel S, et al. Empagliflozin, Cardiovascular Outcomes, and Mortality in Type 2 Diabetes. The New England journal of medicine. 2015;373(22):2117-28.

27. Neal B, Perkovic V, Mahaffey KW, de Zeeuw D, Fulcher G, Erondu N, et al. Canagliflozin and Cardiovascular and Renal Events in Type 2 Diabetes. The New England journal of medicine. 2017;377(7):644-57.

28. Wiviott SD, Raz I, Bonaca MP, Mosenzon O, Kato ET, Cahn A, et al. Dapagliflozin and Cardiovascular Outcomes in Type 2 Diabetes. The New England journal of medicine. 2019;380(4):347-57.

29. Perkovic V, Jardine MJ, Neal B, Bompoint S, Heerspink HJL, Charytan DM, et al. Canagliflozin and Renal Outcomes in Type 2 Diabetes and Nephropathy. The New England journal of medicine. 2019;380(24):2295-306.

30. McMurray JJV, Solomon SD, Inzucchi SE, Kober L, Kosiborod MN, Martinez FA, et al. Dapagliflozin in Patients with Heart Failure and Reduced Ejection Fraction. The New England journal of medicine. 2019;381(21):1995-2008.

31. Seferovic PM, Fragasso G, Petrie M, Mullens W, Ferrari R, Thum T, et al. Sodium-glucose co-transporter 2 inhibitors in heart failure: beyond glycaemic control. A position paper of the Heart Failure Association of the European Society of Cardiology. Eur J Heart Fail. 2020;22(9):1495-503.

32. Cannon CP, Pratley R, Dagogo-Jack S, Mancuso J, Huyck S, Masiukiewicz U, et al. Cardiovascular Outcomes with Ertugliflozin in Type 2 Diabetes. The New England journal of medicine. 2020;383(15):1425-35.

33. Packer M, Anker SD, Butler J, Filippatos G, Pocock SJ, Carson P, et al. Cardiovascular and Renal Outcomes with Empagliflozin in Heart Failure. The New England journal of medicine. 2020;383(15):1413-24.

34. Bhatt DL, Szarek M, Steg PG, Cannon CP, Leiter LA, McGuire DK, et al. Sotagliflozin in Patients with Diabetes and Recent Worsening Heart Failure. The New England journal of medicine. 2021;384(2):117-28.

35. Heerspink HJL, Stefansson BV, Correa-Rotter R, Chertow GM, Greene T, Hou FF, et al. Dapagliflozin in Patients with Chronic Kidney Disease. The New England journal of medicine. 2020;383(15):1436-46.

36. Bhatt DL, Szarek M, Pitt B, Cannon CP, Leiter LA, McGuire DK, et al. Sotagliflozin in Patients with Diabetes and Chronic Kidney Disease. The New England journal of medicine. 2021;384(2):129-39.

37. Anker SD, Butler J, Filippatos G, Ferreira JP, Bocchi E, Böhm M, et al. Empagliflozin in Heart Failure with a Preserved Ejection Fraction. New England Journal of Medicine. 2021;385(16):1451-61.

38. Solomon SD, McMurray JJV, Claggett B, de Boer RA, DeMets D, Hernandez AF, et al. Dapagliflozin in Heart Failure with Mildly Reduced or Preserved Ejection Fraction. New England Journal of Medicine. 2022;387(12):1089-98.

39. EMPA-KIDNEY Collaborative Group. Empagliflozin in Patients with Chronic Kidney Disease. New England Journal of Medicine. 2022.

40. Himsworth HP. The relation of glycosuria to glycaemia and the determination of the renal threshold for glucose. Biochem J. 1931;25(4):1128-46.

41. Hjarne U. A Study of Orthoglycaemic Glycosuria with Particular Reference to it Hereditability. Acta Medica Scandinavica. 1927;67(1):495-571.

42. Poulsson LT. On the mechanism of sugar elimination in phlorrhizin glycosuria. A contribution to the filtration-reabsorption theory on kidney function. J Physiol. 1930;69(4):411-22.

43. Song P, Onishi A, Koepsell H, Vallon V. Sodium glucose cotransporter SGLT1 as a therapeutic target in diabetes mellitus. Expert Opin Ther Targets. 2016;20(9):1109-25.

44. Cefalo CMA, Cinti F, Moffa S, Impronta F, Sorice GP, Mezza T, et al. Sotagliflozin, the first dual SGLT inhibitor: current outlook and perspectives. Cardiovasc Diabetol. 2019;18(1):20.

45. Grempler R, Thomas L, Eckhardt M, Himmelsbach F, Sauer A, Sharp DE, et al. Empagliflozin, a novel selective sodium glucose cotransporter-2 (SGLT-2) inhibitor: characterisation and comparison with other SGLT-2 inhibitors. Diabetes Obes Metab. 2012;14(1):83-90.

46. Lee WS, Kanai Y, Wells RG, Hediger MA. The high affinity Na+/glucose cotransporter. Re-evaluation of function and distribution of expression. J Biol Chem. 1994;269(16):12032-9.

47. Food and Drug Administration. Guidance for Industry on Diabetes Mellitus-Evaluating Cardiovascular Risk in New Antidiabetic Therapies to Treat Type 2 Diabetes. A Notice by the Food and Drug Administration on 12/19/2008. https://www.federalregister.gov/documents/2008/12/19/E8-30086/guidance-for-industry-on-diabetes-mellitus-evaluating-cardiovascular-risk-in-new-antidiabetic (accessed 1st January 2021).

48. Wheeler DC, Stefansson BV, Jongs N, Chertow GM, Greene T, Hou FF, et al. Effects of dapagliflozin on

major adverse kidney and cardiovascular events in patients with diabetic and non-diabetic chronic kidney disease: a prespecified analysis from the DAPA-CKD trial. Lancet Diabetes Endocrinol. 2021;9(1):22-31.

49. Nuffield Department of Population Health Renal Studies Group and the SGLT2 inhibitor Meta-Analysis Cardio-Renal Trialists' Consortium. Impact of diabetes on the effects of sodium glucose co-transporter-2 inhibitors on kidney outcomes: collaborative meta-analysis of large placebo-controlled trials. The Lancet. 2022;400(10365):1788-801.

50. Wanner C, Inzucchi SE, Lachin JM, Fitchett D, von Eynatten M, Mattheus M, et al. Empagliflozin and Progression of Kidney Disease in Type 2 Diabetes. The New England journal of medicine. 2016;375(4):323-34.

51. Vallon V, Thomson SC. The tubular hypothesis of nephron filtration and diabetic kidney disease. Nat Rev Nephrol. 2020;16(6):317-36.

52. Ferrannini E, Baldi S, Frascerra S, Astiarraga B, Heise T, Bizzotto R, et al. Shift to Fatty Substrate Utilization in Response to Sodium-Glucose Cotransporter 2 Inhibition in Subjects Without Diabetes and Patients With Type 2 Diabetes. Diabetes. 2016;65(5):1190-5.

53. Al-Jobori H, Daniele G, Cersosimo E, Triplitt C, Mehta R, Norton L, et al. Empagliflozin and Kinetics of Renal Glucose Transport in Healthy Individuals and Individuals With Type 2 Diabetes. Diabetes. 2017;66(7):1999-2006.

54. Bays HE, Weinstein R, Law G, Canovatchel W. Canagliflozin: effects in overweight and obese subjects without diabetes mellitus. Obesity. 2014;22(4):1042-9.

55. Herrington WG, Preiss D, Haynes R, von Eynatten M, Staplin N, Hauske SJ, et al. The potential for improving cardio-renal outcomes by sodium-glucose co-transporter-2 inhibition in people with chronic kidney disease: a rationale for the EMPA-KIDNEY study. Clin Kidney J. 2018;11(6):749-61.

56. Neuen BL, Young T, Heerspink HJL, Neal B, Perkovic V, Billot L, et al. SGLT2 inhibitors for the prevention of kidney failure in patients with type 2 diabetes: a systematic review and meta-analysis. Lancet Diabetes Endocrinol. 2019;7(11):845-54.

57. Zannad F, Ferreira JP, Pocock SJ, Zeller C, Anker SD, Butler J, et al. Cardiac and Kidney Benefits of Empagliflozin in Heart Failure Across the Spectrum of Kidney Function: Insights From EMPEROR-Reduced. Circulation. 2021;143(4):310-21.

58. Sridhar VS, Tuttle KR, Cherney DZI. We Can Finally Stop Worrying About SGLT2 Inhibitors and Acute Kidney Injury. American journal of kidney diseases : the official journal of the National Kidney Foundation. 2020;76(4):454-6.

59. Jhund PS, Solomon SD, Docherty KF, Heerspink HJL, Anand IS, Bohm M, et al. Efficacy of Dapagliflozin on Renal Function and Outcomes in Patients With Heart Failure With Reduced Ejection Fraction: Results of DAPA-HF. Circulation. 2021;143(4):298-309.

60. Basile DP, Bonventre JV, Mehta R, Nangaku M, Unwin R, Rosner MH, et al. Progression after AKI: Understanding Maladaptive Repair Processes to Predict and Identify Therapeutic Treatments. Journal of the American Society of Nephrology : JASN. 2016;27(3):687-97.

61. McGuire DK, Shih WJ, Cosentino F, Charbonnel B, Cherney DZI, Dagogo-Jack S, et al. Association of SGLT2 Inhibitors With Cardiovascular and Kidney Outcomes in Patients With Type 2 Diabetes: A Meta-analysis. JAMA Cardiol. 2021;6(2):148-58.

62. Herrington WG, Savarese G, Haynes R, Marx N, Mellbin L, Lund LH, et al. Cardiac, renal, and metabolic effects of sodium-glucose co-transporter 2 inhibitors: a position paper from the European Society of Cardiology ad-hoc task force on sodium-glucose co-transporter 2 inhibitors. Eur J Heart Fail. 2021;23(8):1260-75.

63. Ferrannini E, Muscelli E, Frascerra S, Baldi S, Mari A, Heise T, et al. Metabolic response to sodium-glucose cotransporter 2 inhibition in type 2 diabetic patients. J Clin Invest. 2014;124(2):499-508.

64. Ferrannini E. Sodium-Glucose Co-transporters and Their Inhibition: Clinical Physiology. Cell Metab. 2017;26(1):27-38.

65. Zaccardi F, Webb DR, Htike ZZ, Youssef D, Khunti K, Davies MJ. Efficacy and safety of sodium-glucose co-transporter-2 inhibitors in type 2 diabetes mellitus: systematic review and network meta-analysis. Diabetes Obes Metab. 2016;18(8):783-94.

66. Jensen J, Omar M, Kistorp C, Tuxen C, Gustafsson I, Kober L, et al. Effects of empagliflozin on estimated extracellular volume, estimated plasma volume, and measured glomerular filtration rate in patients with heart failure (Empire HF Renal): a prespecified substudy of a double-blind, randomised, placebo-controlled trial. Lancet Diabetes Endocrinol. 2021;9(2):106-16.

67. Bolinder J, Ljunggren O, Kullberg J, Johansson L, Wilding J, Langkilde AM, et al. Effects of dapagliflozin on body weight, total fat mass, and regional adipose tissue distribution in patients with type 2 diabetes mellitus with inadequate glycemic control on metformin. J Clin Endocrinol Metab. 2012;97(3):1020-31.

68. Palmer BF, Clegg DJ. Electrolyte and Acid-Base Disturbances in Diabetes Mellitus. The New England journal of medicine. 2015;373(25):2482-3.

69. Rosenstock J, Marquard J, Laffel LM, Neubacher D, Kaspers S, Cherney DZ, et al. Empagliflozin as Adjunctive to Insulin Therapy in Type 1 Diabetes: The EASE Trials. Diabetes Care. 2018;41(12):2560-9.

70. Dapagliflozin 5mg Summary of Product Characteristics. https://www.medicines.org.uk/emc/medicine/27188#gref (accessed 2nd January 2021).

71. Sotagliflozin Summary of Product Characteristics: https://www.ema.europa.eu/en/documents/product-information/zynquista-epar-product-information_en.pdf (accessed 2nd January 2021).

72. Zynquista: https://www.ema.europa.eu/en/medicines/human/EPAR/zynquista (accessed 14th December 2022).

73. MHRA: Dapagliflozin (Forxiga): no longer authorised for treatment of type 1 diabetes mellitus: https://www.gov.uk/drug-safety-update/dapagliflozin-forxiga-no-longer-authorised-for-treatment-of-type-1-diabetes-mellitus (accessed 14th December 2022).

74. Vallon V, Thomson SC. Targeting renal glucose reabsorption to treat hyperglycaemia: the pleiotropic effects of SGLT2 inhibition. Diabetologia. 2017;60(2):215-25.

75. Ferrannini E, Ramos SJ, Salsali A, Tang W, List JF. Dapagliflozin monotherapy in type 2 diabetic patients with inadequate glycemic control by diet and exercise: a randomized, double-blind, placebo-controlled, phase 3 trial. Diabetes Care. 2010;33(10):2217-24.

76. Bailey CJ, Gross JL, Pieters A, Bastien A, List JF. Effect of dapagliflozin in patients with type 2 diabetes who have inadequate glycaemic control with metformin: a randomised, double-blind, placebo-controlled trial. Lancet. 2010;375(9733):2223-33.

77. Rosenstock J, Seman LJ, Jelaska A, Hantel S, Pinnetti S, Hach T, et al. Efficacy and safety of empagliflozin, a sodium glucose cotransporter 2 (SGLT2) inhibitor, as add-on to metformin in type 2 diabetes with mild hyperglycaemia. Diabetes Obes Metab. 2013;15(12):1154-60.

78. Bersoff-Matcha SJ, Chamberlain C, Cao C, Kortepeter C, Chong WH. Fournier Gangrene Associated With Sodium-Glucose Cotransporter-2 Inhibitors: A Review of Spontaneous Postmarketing Cases. Ann Intern Med. 2019;170(11):764-9.

79. Zannad F, Ferreira JP, Pocock SJ, Anker SD, Butler J, Filippatos G, et al. SGLT2 inhibitors in patients with heart failure with reduced ejection fraction: a meta-analysis of the EMPEROR-Reduced and DAPA-HF trials. Lancet. 2020;396(10254):819-29.

80. Staplin N, Roddick AJ, Emberson J, Reith C, Riding A, Wonnacott A, et al. Net effects of sodium-glucose co-transporter-2 inhibition in different patient groups: a meta-analysis of large placebo-controlled randomized trials. eClinicalMedicine. 2021;41.

81. Secker PF, Beneke S, Schlichenmaier N, Delp J, Gutbier S, Leist M, et al. Canagliflozin mediated dual inhibition of mitochondrial glutamate dehydrogenase and complex I: an off-target adverse effect. Cell Death Dis. 2018;9(2):226.

82. Herrington WG, Savarese G, Haynes R, Marx N, Mellbin L, Lund LH, et al. Cardiac, renal, and metabolic effects of sodium-glucose co-transporter-2 inhibitors: a position paper from the European Society of Cardiology ad-hoc task force on sodium-glucose co-transporter-2 inhibitors European Journal of Heart Failure (in Press). 2021.

83. Garg SK, Henry RR, Banks P, Buse JB, Davies MJ, Fulcher GR, et al. Effects of Sotagliflozin Added to Insulin in Patients with Type 1 Diabetes. New England Journal of Medicine. 2017;377(24):2337-48.

84. Levey AS, Eckardt K-U, Dorman NM, Christiansen SL, Hoorn EJ, Ingelfinger JR, et al. Nomenclature for kidney function and disease: report of a Kidney Disease: Improving Global Outcomes (KDIGO) Consensus Conference. Kidney International. 2020;97(6):1117-29.

Section 2: SGLT-2 inhibition and kidney protection in people with CKD in the context of type 2 diabetes mellitus

2.1 Background

2.1.1 2023 Update to guideline

Since publication of the 2021 version of this guideline, two large trials of SGLT-2 inhibition have been reported (1, 2), including the EMPA-KIDNEY trial (1). The guidelines have been reviewed and revised in light of the new data.

2.1.2 Summary of trial evidence on kidney disease progression

The ‘Recommendations for Use’ covered in this section are evidence-based where sufficient data is available, with a focus on large, high-quality randomised trials. Of particular importance are the three reported ‘kidney outcome trials’ with specifically address the use of SGLT-2 inhibition to reduce progression of kidney disease in a population with CKD (CREDENCE, DAPA-CKD, and EMPA-KIDNEY (1, 3, 4)). All three of these trials investigated a primary outcome comprising a composite of death from cardiovascular causes, death from kidney failure (i.e. renal death), or progression of kidney disease. The latter component of the composite outcome has been variably defined in trials, but consistently combined end-stage kidney disease (ESKD; defined as initiation of maintenance dialysis, kidney transplantation) and eGFR-based components. Categorical outcomes based on eGFR decline have been shown to be valid surrogate markers for progression to kidney failure, supporting their use in the large SGLT-2 inhibitor trials (5, 6). However, for some trial participants in whom the rate of kidney disease progression is expected to be low, categorical assessments of decline in kidney function may fail to detect meaningful differences between SGLT-2 inhibition and placebo, particularly in the context of randomised trials of relatively short duration. For this reason, eGFR slope analyses have been reviewed alongside the categorical outcomes to ensure the totality of the available information on effects on kidney outcomes are considered. Note that in CKD, methods for eGFR slope analyses have been reviewed with regulators and shown to be potentially valid surrogates for kidney disease progression. Absolute differences in the order of 0.5 to 1 mL/min/1.73m^2 per year have been shown to predict a hazard ratio of at least 0.70 on kidney disease progression (7).

The first clear demonstration of the potential for SGLT-2 inhibitors to modify risk of CKD progression was based on these categorical outcomes and emerged from a sub-analysis of the EMPA-REG OUTCOME trial in a type 2 DM population with prior cardiovascular disease. Initial analyses plotting mean eGFR against time showed a modest reversible reduction in eGFR on initiating SGLT-2 inhibition compared to placebo, followed by a substantial decrease in the subsequent rate of chronic eGFR decline over time (Figure 2.1). The retardation of eGFR decline brought about a 46% reduction in the risk of the categorical composite kidney disease progression outcome of ESKD, renal death and a doubling of serum creatinine (hazard ratio [HR]=0.54, 95% confidence interval [CI] 0.40-0.75) (8). Subsequent trials have confirmed these findings, with information on renoprotection now available from trials conducted in a range of different types of people with several different SGLT-2 inhibitors.

Figure 2.1: Effect of allocation to empagliflozin 10 or 25mg versus placebo on CKD-EPI eGFR by time in EMPA-REG OUTCOME (reproduced from (9))

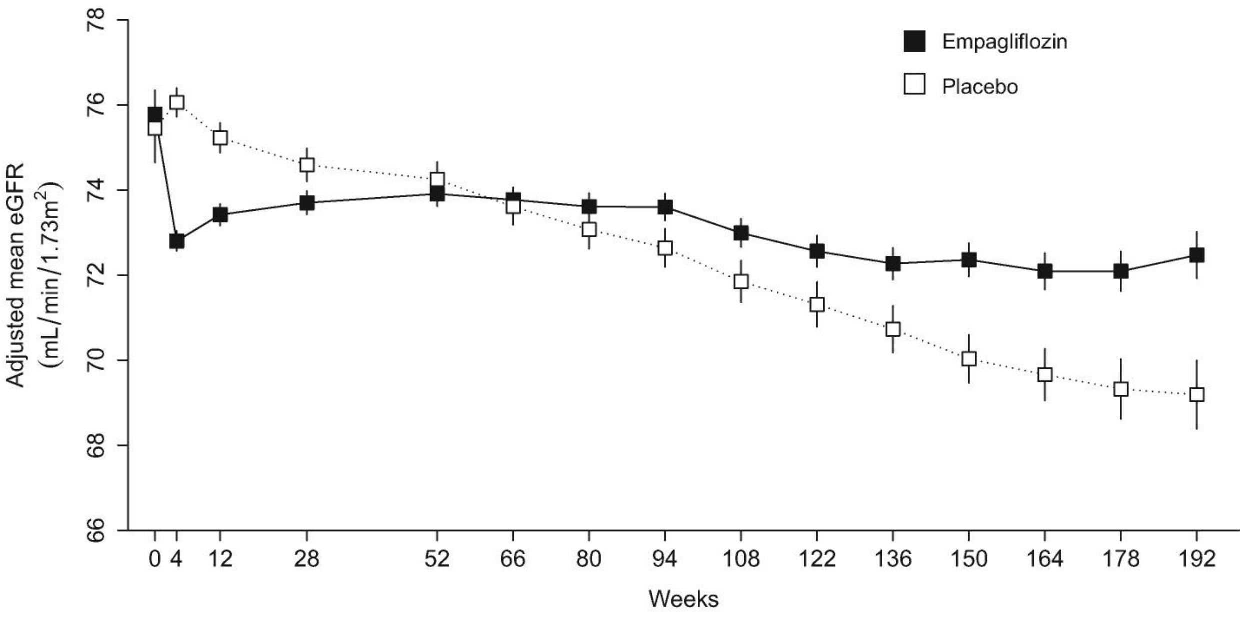

Number of patients analysed

Empagliflozin	4644	4533	4451	4318	4225	4018	4131	3710	3103	2654	2387	2087	1623	1037
Placebo	2323	2267	2205	2121	2064	1927	1981	1763	1479	1262	1123	977	731	448

Pre-specified mixed model repeated measures analysis in patients treated with at least one dose of study drug who had a baseline and post-baseline measurement.

Kidney disease progression results from CREDENCE, DAPA-CKD & EMPA-KIDNEY

The key large trials designed to definitively test the effect of an SGLT-2 inhibitor versus placebo on CKD progression conducted in CKD populations are CREDENCE, DAPA-CKD and EMPA-KIDNEY (1, 3, 4). Their key design features are summarised in Table 1.4 (in Section 1).

CREDENCE recruited people with type 2 DM with the following kidney-related inclusion criteria: eGFR 30-90 mL/min/1.73m^2 plus a urinary albumin-to-creatinine ratio (uACR) of 300-5000 mg/g [UK units: 34-566 mg/mmol]. Participation required treatment with an angiotensin-converting-enzyme inhibitor (ACEi) or angiotensin-II receptor blocker (ARB) for ≥4 weeks at either the maximum labelled dose or a dose not associated with unacceptable side effects. Combined use of ACEi with ARB, or with a direct renin inhibitor, or with a mineralocorticoid-receptor antagonist (MRA) was excluded, as were people with a suspected non-diabetic cause of kidney disease.

DAPA-CKD recruited people with and without type 2 DM. Kidney-related inclusion criteria were an eGFR 25-75 mL/min/1.73m^2 plus a uACR 200-5000 mg/g [23-566 mg/mmol] in participants who had received a stable dose of an ACEi or ARB for ≥4 weeks. Key exclusion criteria were polycystic kidney disease, lupus nephritis, or anti-neutrophil cytoplasmic antibody (ANCA) associated vasculitis, and immunotherapy for kidney disease within 6 months before enrolment (10).

EMPA-KIDNEY identified a broad range of participants at risk of CKD progression using simple kidney

inclusion criteria: an eGFR 20-45 mL/min/1.73m² or an eGFR ≥45-<90 mL/min/1.73m² plus uACR ≥200 mg/g (≥23 mg/mmol, or protein-to-creatinine ratio ≥300 mg/g [≥34 mg/mmol]). Those on intravenous immunosuppression therapy in last 3 months or receiving a dose of >45 mg/day prednisolone were excluded, as were kidney transplant recipients. Polycystic kidney disease was the only excluded primary kidney diagnosis. Note that all these large CKD trials of SGLT-2 inhibitors have excluded people with a history of kidney transplantation (see section 7b for the guideline group's considerations for use in people with a functioning kidney transplant).

A summary of the key characteristics of the populations with type 2 DM from CREDENCE, DAPA-CKD and EMPA-KIDNEY are provided in Table 2.1.

Table 2.1: Kidney characteristics of people with DM in CREDENCE (3),DAPA-CKD (4), and EMPA-KIDNEY (1)

		CREDENCE	DAPA-CKD (DM only)	EMPA-KIDNEY (DM only)
**N with DM (% of total population)**		**4401 (100%)**	**2906 (68%)**	**3039 (46%)**
**eGFR, mL/min/1.73m²**	**Mean (SD)**	**56 (18)**	**44 (13)**	**36 (14)**
≥60		1809 (41%)	348 (12%)	532 (18%)
45-59		1279 (29%)	918 (32%)	
30-45		1313 (30%)	1239 (43%)	1359 (45%)
<30		-	401 (14%)	1148 (38%)*
**Albuminuria (uACR cut-off)**	**Median (Q1-Q3)**	**927 (463-1833)**	**1017 (~475-1900)**	**348 (68-1293)**
Normoalbuminuria (<30mg/g)		31 (1%)	1 (0%)	649 (21%)
Microalbuminuria (30-300 mg/g)		496 (11%)	307 (11%)	941 (31%)
Macroalbuminuria (>300 mg/g)		3874 (88%)	2597 (89%)	1449 (48%)

Data are n (%), or mean (standard deviation) or median (Q1-Q3) where stated. DM=diabetes mellitus; uACR=urinary albumin-to-creatinine ratio. *Includes 254 participants with eGFR <20 mL/min/1.73m² at randomisation.

All three dedicated CKD trials were stopped early for efficacy by their respective data monitoring committees. In the CREDENCE trial, canagliflozin reduced the risk its primary composite outcome, defined as a sustained doubling of creatinine, ESKD, or death from renal or cardiovascular causes, by 30% compared to placebo (245/2202 vs 340/2199: HR=0.70, 95%CI 0.59-0.82). Importantly, there were reductions in the risk of kidney disease progression, including ESKD. Risk of receipt of maintenance dialysis, kidney transplantation or a renal death was significantly reduced by 28% (3). These benefits were unmodified by baseline level of eGFR and glycosylated haemoglobin (HbA1c). Figure 2.2 provides the effects on the kidney disease progression component of the primary outcome for CREDNCE and the other CKD progression trials, split by diabetes status.

DAPA-CKD demonstrated a reduction in the primary composite outcome, defined as a sustained 50% decline in eGFR, ESKD, or death from renal or cardiovascular causes, by 39% with dapagliflozin compared to placebo (197/2152 vs 312/2152: HR=0.61, 95%CI 0.51-0.72). Importantly, these relative risks were again apparent for the kidney disease progression component of the primary composite (see Figure 2.2), and ESKD. They were also similar when analyses were performed separately in people with and without type 2 DM, and in pre-specified subgroups defined by eGFR and uACR (4). There was also a clear reduction in risk of kidney disease progression, with fewer initiations of maintenance dialysis overall and among those with DM considered in isolation. DAPA-CKD therefore reinforced the findings on albuminuric diabetic kidney disease

from CREDENCE (3).

Figure 2.2: Effects of SGLT-2 inhibitors on kidney disease progression by population (adapted from (11))

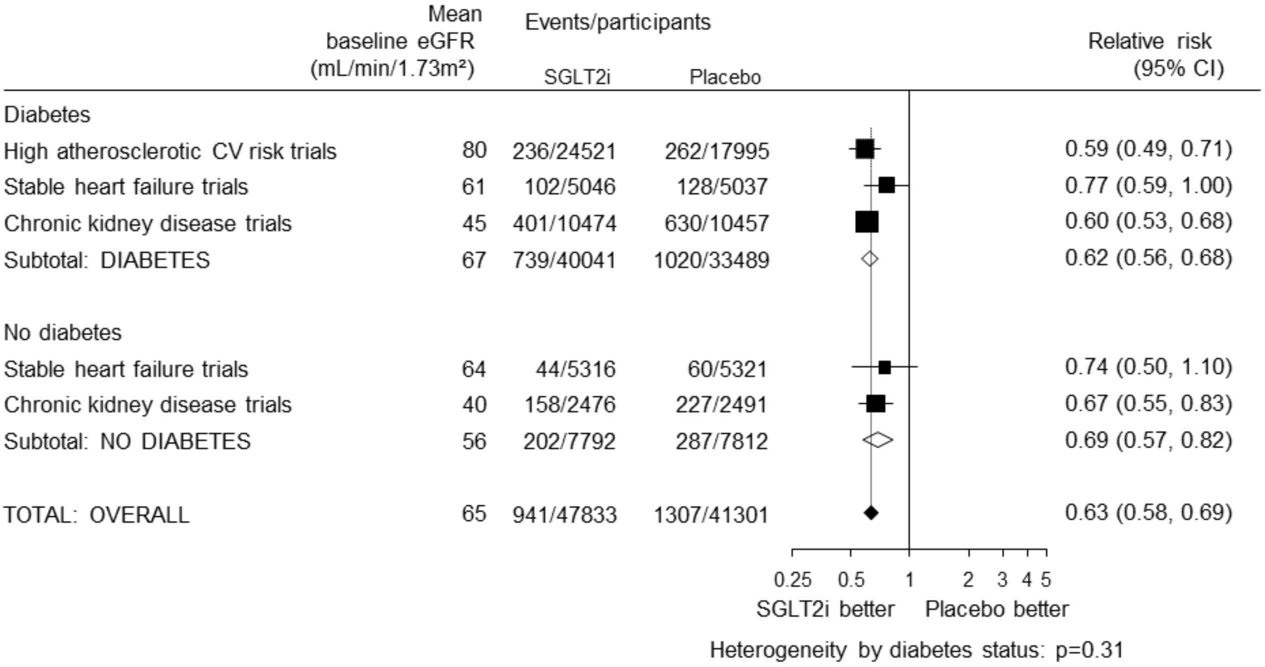

Kidney disease progression was defined as a sustained ≥50% decline in eGFR from randomisation, kidney failure, or death from kidney failure. Data not available for SOLOIST-WHF.

EMPA-KIDNEY reported a reduction in the primary outcome, defined as a composite of progression of kidney disease (defined as end-stage kidney disease, a sustained decrease in eGFR to <10 mL/min/1.73m^2, a sustained decrease in eGFR of ≥40% from baseline, or death from renal causes) or death from cardiovascular causes, by 28% (432/3304 vs 558/3305, HR=0.72, 95%CI 0.64-0.82) (1). As with CREDENCE and DAPA-CKD, similar effects were observed for the subcomponents of kidney disease progression including a significant reduction in a secondary composite outcome of ESKD or death from cardiovascular causes. The relative effects on the primary outcomes were broadly similar in people with and without diabetes and across the spectrum of eGFR studied, with no evidence any attenuation of effect even in the post-hoc subgroup analyses including 111 primary outcomes in 254 participants with an eGFR below 20 (see Figure 2.5 below). There was evidence, however, that the effect on primary outcome was larger in participants with a higher baseline levels of uACR (p-value for trend = 0.02). There were few kidney disease progression events in people with lesser degrees of albuminuria, particularly those with normoalbuminuria due to their slower rate of progression, and exploring pre-specified eGFR-slope-based analyses was important to help interpret these subgroup findings and identify slowing of progression in those with lower levels of uACR (see below).

In a comprehensive meta-analysis of the large randomised trials in SGLT-2 inhibition conducted by the Nuffield Department of Population Health Renal Studies Group (RSG) and the SGLT2 inhibitor Meta-Analysis Cardio-Renal Trialists' Consortium (SMART-C), a unified definition of kidney disease progression was adopted (11). Kidney disease progression was defined as a sustained ≥50% decline in eGFR from randomisation, kidney failure, or death from kidney failure. The meta-analysis demonstrated an overall 37% reduction in risk of disease progression (HR=0.63, 95%CI 0.58-0.69), which was similar among participants with and without diabetes ($P_{interaction}$=0.31; Figure 2.2). In participants with diabetes, the HR for this kidney disease progression outcome was 0.64 (95%CI 0.52-0.79) in CREDENCE, 0.57 (95%CI 0.45-0.73) in DAPA-CKD, and 0.55 (95%CI

0.44-0.71) in EMPA-KIDNEY (11). Such similar treatment effects lends support to the supposition that the kidney disease progression benefits are a class effect.

Among the CKD trials (1, 3, 4, 12), the key clinical outcome of kidney failure (excluding the surrogate categorical eGFR decline thresholds) was similarly reduced by 33% (HR=0.67, 95%CI 0.59-0.77) without interaction by diabetes status (11).

Among trials that reported primary kidney diagnosis (CREDENCE, SCORED, DAPA-CKD and EMPA-KIDNEY for diabetic kidney disease; DAPA-CKD and EMPA-KIDNEY for other kidney diagnoses), there was no evidence that the benefits of SGLT-2 inhibition varied by primary diagnosis (Figure 2.3). Given the lack of interaction by diabetes status or primary kidney diagnosis, the best estimate of the relative benefits of SGLT-2 inhibition in an individual with diabetes is the overall estimate derived from meta-analysis, irrespective of underlying cause of kidney disease. Importantly, each of the different tested SGLT-2 inhibitors had a similar sized relative effect on kidney disease progression in people with diabetes (see Figure 2.3 result for diabetic kidney disease or nephropathy).

Figure 2.3: Effects of SGLT-2 inhibition on kidney disease progression by primary kidney diagnosis (adapted from (11))

	Mean baseline eGFR, mL/min per 1·73m²	Events/participants		Event rate per 1000 patient-years		RR (95% CI)
		SGLT2 inhibitor	Placebo	SGLT2inhibitor	Placebo	
**Diabetic kidney disease or nephropathy***						
CREDENCE	56	153/2202	230/2199	27	41	0·64 (0·52–0·79)
SCORED	44	37/5292	52/5292	5	7	0·71 (0·46–1·08)
DAPA-CKD	43	93/1271	157/1239	36	64	0·55 (0·43–0·71)
EMPA-KIDNEY	36	85/1032	133/1025	42	67	0·56 (0·43–0·74)
**Subtotal**	**46**	**368/9797**	**572/9755**	··	··	**0·60 (0·53–0·69)**
**Ischaemic and hypertensive kidney disease**						
DAPA-CKD	43	18/324	26/363	28	37	0·74 (0·40–1·36)
EMPA-KIDNEY	35	37/706	52/739	27	37	0·69 (0·45–1·05)
**Subtotal**	**38**	**55/1030**	**78/1102**	··	··	**0·70 (0·50–1·00)**
**Glomerular disease**						
DAPA-CKD	43	21/343	46/352	33	70	0·43 (0·26–0·72)
EMPA-KIDNEY	42	69/853	95/816	44	64	0·68 (0·50–0·93)
**Subtotal**	**42**	**90/1196**	**141/1168**	··	··	**0·60 (0·46–0·78)**
**Other kidney disease or unknown**						
DAPA-CKD	43	10/214	14/198	25	37	0·81 (0·35–1·83)
EMPA-KIDNEY	36	36/713	52/725	26	36	0·72 (0·47–1·10)
**Subtotal**	**38**	**46/927**	**66/923**	··	··	**0·74 (0·51–1·08)**
**Any diagnosis**						
CREDENCE	56	153/2202	230/2199	27	41	0·64 (0·52–0·79)
SCORED	44	37/5292	52/5292	5	7	0·71 (0·46–1·08)
DAPA-CKD	43	142/2152	243/2152	33	58	0·56 (0·45–0·68)
EMPA-KIDNEY	37	227/3304	332/3305	36	52	0·64 (0·54–0·76)
**Total**	**44**	**559/12 950**	**857/12 948**	··	··	**0·62 (0·56–0·69)**

Heterogeneity across groups of primary kidney disease: p=0·67
Trend across trials sorted by eGFR for any diagnosis: p=0·88

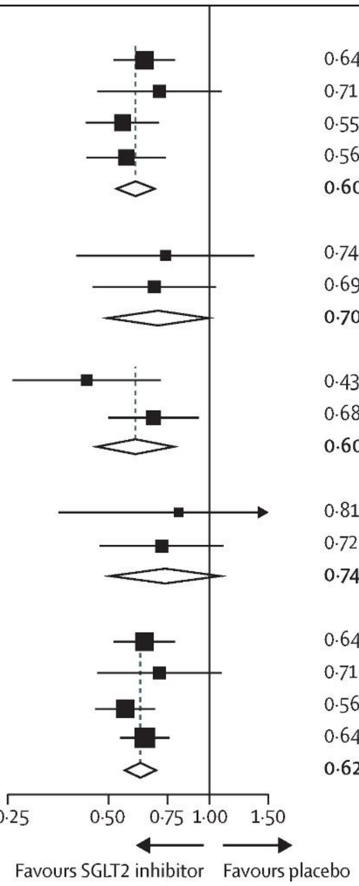

*RR in the diabetic kidney disease or nephropathy subgroup excluding SCORED (which did not formally assess primary kidney disease) is 0.59 (95% CI 0.52–0.68).

Kidney disease progression results from trials including people without albuminuria

While the evidence for use of SGLT-2 inhibition for the treatment of albuminuric kidney disease is now well-established, evidence for treatment of people with CKD without albuminuria is more limited. Given that mitigation of hyperfiltration is a proposed mechanism of renoprotection with SGLT-2 inhibition, it is plausible that benefits of treatment are greater among individuals with higher degrees of albuminuria. Both CREDENCE and DAPA-CKD excluded people with low-to-moderate levels of albuminuria (<300mg/g and <200mg/g, respectively), and around 90% of participants of both studies had A3 levels of albuminuria (i.e. macroalbuminuria) (Table 2.1) (3, 4).

The largest body of evidence for people with CKD with A1-2 levels of albuminuria is provided by EMPA-KIDNEY, which did not stipulate any albuminuria requirements for inclusion for people with eGFR 20-45 mL/min/1.73m^2, and in which ~20% of included participants had A1 levels (uACR <30 mg/g) (1). Participants without albuminuria were at substantially lower risk of the primary outcome. In the placebo arm, 42/663 [6.3%] experienced a kidney disease progression outcome among those with uACR <30 mg/g, vs 438/1705 [25.7%] among those with uACR >300 mg/g, and fewer than 10% of the trial's total accrued kidney disease progression outcomes occurred in participants without albuminuria at baseline. There was evidence that the beneficial effect of SGLT-2 inhibition was attenuated among those with lesser degrees of albuminuria (kidney disease progression outcome subgroup analyses trend p value=0.02). Exploratory eGFR slope analyses were used to assess if this represented attenuated benefit or no effect on kidney progression. The chronic rate of decline in eGFR (from 2 months to final follow-up) was reduced by empagliflozin in all albuminuria groups, with the largest benefit noted in people with A3 levels of albuminuria (absolute difference in rate of eGFR decline: 1.76 mL/min/1.73m^2 per year [95%CI 1.46-2.05]). Among people with A1 levels of albuminuria (<30 mg/g), rate of eGFR decline was low among placebo-allocated participants (-0.89 mL/min/1.73m^2 per year) and was significantly reduced by empagliflozin by 0.78 mL/min/1.73m^2 per year (95%CI 0.32-1.23), with a rate of kidney function decline in empagliflozin-treated participants of -0.11 mL/min/1.73m^2 per year (Figure 2.4). Similarly, in people with A2 levels of albuminuria (≥30 to ≤300 mg/g), empagliflozin reduced the rate of kidney function decline by 1.20 mL/min/1.73m^2 per year (95%CI 0.81-1.59) (Figure 2.4). These absolute differences meet or exceed the 0.5-1 mL/min/1.73m^2 per year threshold identified as a valid surrogate for progression to kidney failure (7), and represent substantial relative reductions in the annual rate of progression. A limitation of EMPA-KIDNEY was that follow-up was only for two years. As there was about a 2-3 mL/min/1.73m^2 acute negative dip in eGFR on initiation of empagliflozin, and those with A1 levels of albuminuria in the placebo arm progressed at just only about 1 mL/min/1.73m^2/year. For this specific subpopulation, the trial was too short to be able to demonstrate effects on the kidney disease progression component of the primary outcome.

Figure 2.4: Effects of empagliflozin versus placebo on the rate of eGFR decline in EMPA-KIDNEY, by key subgroups at baseline (adapted from (1))

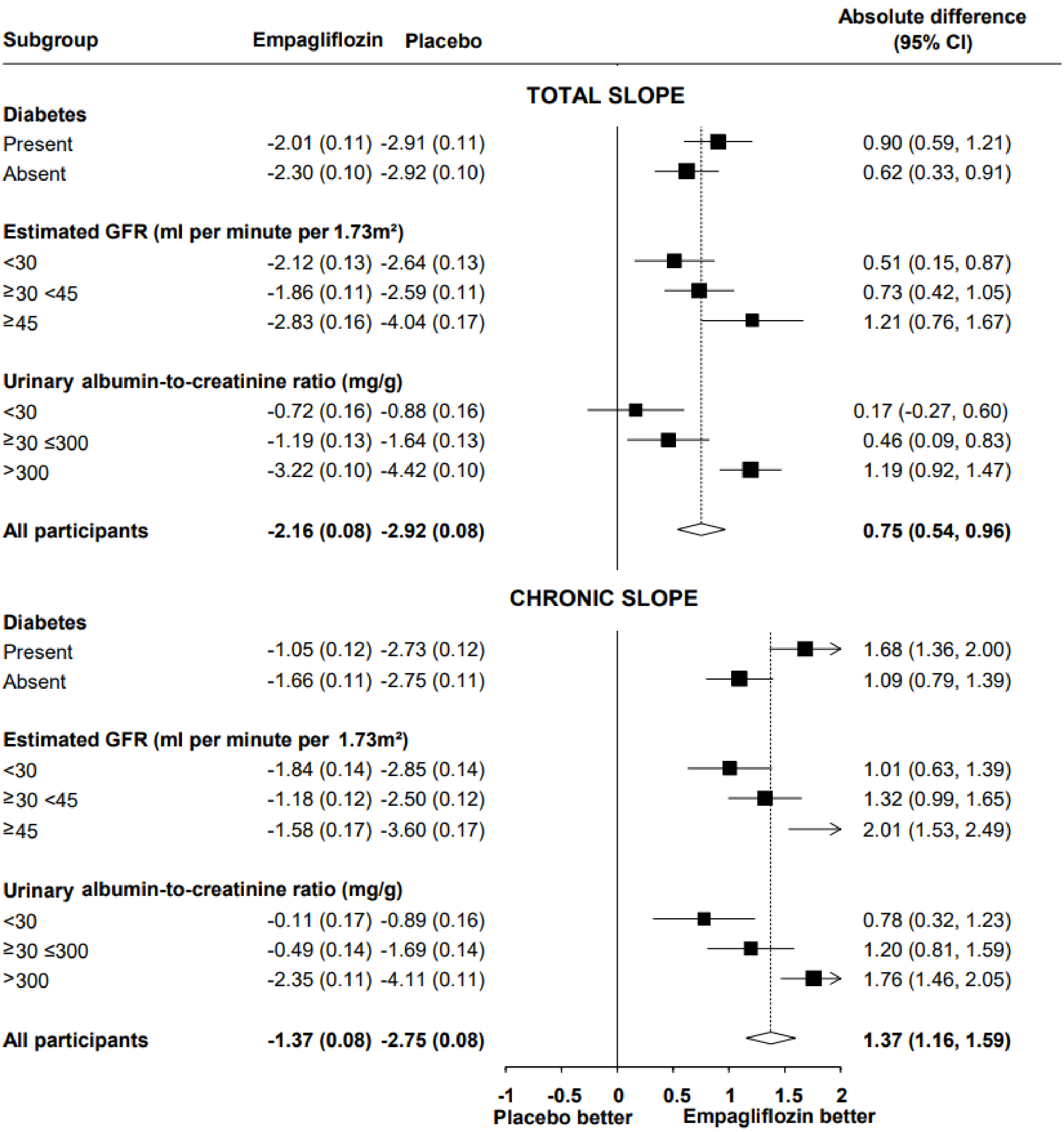

Mean annual rate of change in eGFR (mL/min/1.73m^2/year) from baseline to final follow-up visit (“total slope”) and from two months to final follow-up visit (“chronic slope”).

More data in people with low levels of albuminuria are available from the heart failure trials (some of which prespecified eGFR slope analyses as secondary outcomes). eGFR slope analyses from the EMPEROR program, DAPA-HF and DELIVER suggest that the renoprotective effects of SGLT-2 inhibition extend to heart failure

populations (13-16). In EMPEROR-REDUCED, the rate of decline in eGFR was reduced by 1.73 mL/min/1.73m^2 per year, and was similar across the spectrum of uACR categories, though this analysis included participants with and without CKD (14). Rate of eGFR decline was attenuated with empagliflozin in participants both with and without CKD. Both EMPEROR-PRESERVED and DELIVER demonstrated a significant reduction in rate of eGFR decline in the SGLT-2 inhibitor group vs placebo (difference in total eGFR slope for EMPEROR-PRESERVED: 1.36 mL/min/1.73m^2 per year, 95%CI 1.06-1.66; DELIVER: 0.5 mL/min/1.73m^2, 95%CI 0.1-0.9 [chronic slope analyses not presented]) (13, 17). In both trials the absolute numbers of categorical kidney disease progression outcomes was low (152/6262 in DELIVER, 220/5988 in EMPEROR-PRESERVED), and given the slow rate of progression in the trial cohorts, the duration of follow up may have been too short for meaningful differences between treatment groups to emerge (median follow up of 21 and 28 months in EMPEROR-PRESERVED and DELIVER, respectively).

Kidney disease progression in people with eGFR <20 mL/min/1.73m^2 and on maintenance dialysis

EMPA-KIDNEY, DAPA-CKD and CREDENCE enrolled participants with eGFR ≥20, ≥25, and ≥30 mL/min/1.73m^2, respectively (1, 3, 4). However, in these trials treatment with SGLT-2 inhibitor (or placebo) was continued at least until initiation of kidney replacement therapy (initiation of maintenance dialysis or kidney transplantation), providing indirect evidence regarding the safety of SGLT-2 inhibition among people with eGFR <20 mL/min/1.73m^2. Furthermore, in EMPA-KIDNEY a number of participants with eGFR ≥20 at screening visit experienced further decline in eGFR between screening and randomisation. In total, 254 participants in EMPA-KIDNEY were randomised with an eGFR between 15-20 mL/min/1.73m^2. In an exploratory post-hoc analysis, the hazard ratio for the primary cardiorenal outcome among participants at all strata of eGFR, including 111 events among those with eGFR <20 mL/min/1.73m^2, was of a similar magnitude to the overall effect estimate, with no evidence that the relative benefits of empagliflozin attenuated to any degree among people with low eGFR <20 (P value for trend=0.81; Figure 2.5) (1, 18). There is no evidence of an eGFR threshold below which kidney benefits start to attenuate down to CKD stage 5. Importantly, people with markedly decreased eGFR are likely to be at highest absolute risk of kidney failure (indeed, in EMPA-KIDNEY approximately one-half of placebo-treated participants with eGFR<20 mL/min/1.73m^2 experienced a primary outcome). Therefore initiating SGLT2 inhibitors in people with CKD with an eGFR <20 would be expected to yield larger absolute benefits at a population level than treating people with higher eGFR (for a given level of albuminuria). Even among people with slow rate of progression of CKD (such as those without significant albuminuria), treatment of those with low eGFR with SGLT-2 inhibition may delay the onset of kidney failure if used over years. Given the presence of an acute dip in eGFR following initiation of SGLT-2 inhibition, some caution is advised in people who are about to need kidney replacement therapy.

The CKD trials generally discontinued SGLT-2 inhibitors after receipt of a kidney transplant (see Section 7b for further details on the use of SGLT-2 inhibition in people with a kidney transplant). DAPA-CKD, which permitted participants to continue dapagliflozin after initiation of chronic maintenance dialysis, presented data on 167 participants on maintenance dialysis, in whom mortality rate was much greater than the non-dialysis population, and numerically lower with dapagliflozin treatment than placebo (19). There is currently insufficient data to make recommendations on the use of SGLT-2 inhibition in people receiving maintenance dialysis. The large RENAL-LIFECYCLE trial (NCT05374291) will investigate the use of dapagliflozin vs placebo for prevention of death, kidney failure or hospitalisation for heart failure in people with kidney failure, maintenance dialysis, or kidney transplantation and will provide further information on the use of SGLT-2 inhibition in this population (20).

Figure 2.5: Effects of empagliflozin versus placebo on the primary outcome of EMPA-KIDNEY, by baseline eGFR (post-hoc analysis)

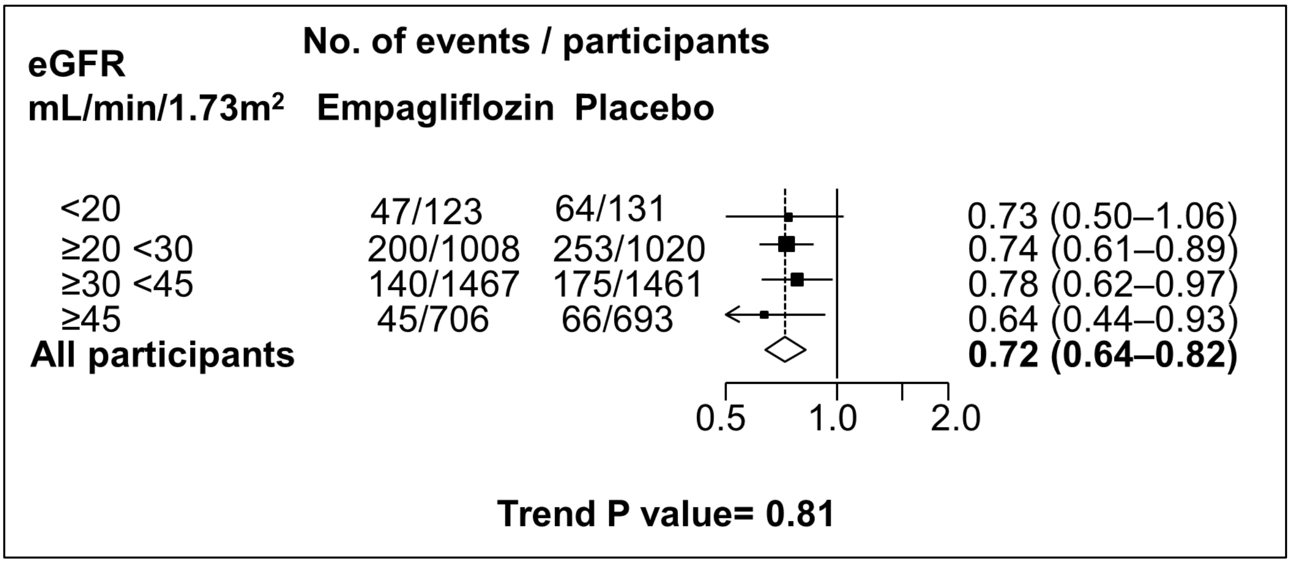

Acute kidney injury

Given the mechanisms of action of SGLT-2 inhibitors, including the acute decline of eGFR on initiation and induction of osmotic diuresis and natriuresis, early concerns were raised regarding risk of acute kidney injury (AKI) in people treated with SGLT-2 inhibition. However, such concerns were not borne out of trial evidence, which consistently provided reassuring data on the risk of AKI. No individual trial demonstrated increased risk, and several early trials among people with diabetes at high cardiovascular risk suggested a lower rate of AKI events among SGLT-2 inhibitor-treated participants (8, 21). This has been confirmed by meta-analyses, including the RSG and SMART-C meta-analysis, which showed that SGLT-2 inhibition reduces the risk of AKI by about a quarter (HR=0.77, 95%CI 0.70-0.84), irrespective of diabetes status (11, 22). While the mechanism underlying renoprotection in the context of AKI is not fully understood, these beneficial effects may in part explain some of the benefits of SGLT-2 inhibition on progression of kidney disease. Section 5 discusses the evidence regarding acute kidney injury with SGLT-2 inhibition in more detail.

Co-prescription with mineralocorticoid receptor antagonism

There is likely to be increasing use of MRA in people with albuminuric CKD and type 2 diabetes due to recent positive results from finerenone trials and updated guideline recommendations. FIDELIO-DKD demonstrated the efficacy of finerenone compared to placebo at reducing risk of kidney progression in people with albuminuric diabetic kidney disease and type 2 DM, while the complementary FIGARO-DKD trial demonstrated cardiovascular benefits (primarily driven by reduced hospitalisation for heart failure) among people with CKD and diabetes with moderately or severely increased levels of albuminuria and preserved eGFR (23, 24). A European Society of Cardiology position paper has recommended early use of SGLT-2 inhibition in people with heart failure with reduced ejection fraction (HFrEF) in addition to class IA recommended medications (i.e. beta-blockers, ACEi/ARBs and MRAs) (25), and 2022 KDIGO guidelines for the management of type 2 diabetes in CKD now suggest initiation of a non-steroidal MRA in people with albuminuric CKD (26).

There are limited data regarding co-prescription of SGLT-2 inhibition and MRAs in the large trials of CKD. CREDENCE excluded use of MRA at baseline (3), while only 229 (5.3%) participants of DAPA-CKD were co-prescribed an MRA (27). In DAPA-CKD, MRA use did not modify the effects of SGLT-2 inhibition on kidney disease progression outcomes or risk of hyperkalaemia (27). Similarly, univariable subgroup analyses by baseline MRA co-prescription from several of the non-CKD trials have found that MRA use did not modify the key findings from these trials (12, 28-34).

Hyperkalaemia may result from use of MRA, but combining SGLT-2 inhibition with renin-angiotensin-system (RAS) blockade does not have the same potential as dual RAS blockade to cause hyperkalaemia (3, 4, 36, 37). This also appears true in people co-prescribed MRA with SGLT-2 inhibitors (31-33, 38). Data from individual trials and meta-analyses raise the hypothesis that SGLT-2 inhibition may reduce the risk of serious hyperkalaemia (32, 33, 38).

The knowledge gap regarding the safety of co-prescription of finerenone and SGLT-2 inhibition in CKD will be addressed by the ongoing phase 2 CONFIDENCE trial, which is planned to randomise around 800 people with albuminuric CKD to finerenone, empagliflozin, or their combination (with matching placebos as necessary) (35). The primary efficacy outcome will be change in uACR from baseline to 180 days (in combination therapy group vs empagliflozin and finerenone, respectively), with secondary and safety outcomes including the total number of AKI events and total number of hyperkalaemia events on treatment. The trial, which is expected to complete in 2024, will provide important safety information to support combination therapy with SGLT-2 inhibition and non-steroidal MRAs.

2.1.3 Summary of trial evidence on cardiovascular risk

Heart failure

Randomised clinical trials and meta-analyses have consistently shown that SGLT-2 inhibition reduces cardiovascular death or hospitalization for heart failure across the studied populations, including people with type 2 diabetes at high risk of atherosclerotic cardiovascular disease (11, 21, 39, 40), people with heart failure across the spectrum of ejection fraction (2, 11, 16, 17, 41), and in CKD (3, 4, 11, 12) (Figure 2.6). In a meta-analysis of large RCTs in SGLT-2 inhibition, SGLT-2 inhibition reduced cardiovascular death or hospitalisation for heart failure by 23% compared to placebo (HR=0.77, 95%CI 0.74-0.81) (11).

Figure 2.6: Effects of SGLT-2 inhibitors on hospitalisation for heart failure by trial and diabetes status (adapted from (11))

	Average baseline eGFR (mL/min/1.73m²)	Events/participants SGLT2i	Events/participants Placebo	Rate per 1000 patient years SGLT2i	Rate per 1000 patient years Placebo	Relative risk (95% CI)	Trend across trials sorted by eGFR
**Diabetes**							
DECLARE-TIMI 58	85	417/8582	496/8578	12	15	0.83 (0.73, 0.95)	
CANVAS Program	77	364/5795	288/4347	16	21	0.78 (0.67, 0.91)	
VERTIS CV	76	444/5499	250/2747	23	27	0.88 (0.75, 1.03)	
EMPA-REG OUTCOME	74	265/4687	198/2333	20	30	0.66 (0.55, 0.79)	
DAPA-HF	63	213/1075	268/1064	144	191	0.75 (0.63, 0.90)	
EMPEROR-REDUCED	61	200/927	265/929	177	246	0.72 (0.60, 0.87)	
EMPEROR-PRESERVED	60	239/1466	291/1472	83	102	0.79 (0.67, 0.94)	p=0.22
DELIVER	60	271/1578	330/1572	83	104	0.80 (0.68, 0.93)	
CREDENCE	56	179/2202	253/2199	32	45	0.69 (0.57, 0.83)	
SOLOIST-WHF	51	NA/608	NA/614	-	-	0.71 (0.56, 0.89)	
SCORED	44	283/5292	357/5292	41	52	0.77 (0.66, 0.91)	
DAPA-CKD	44	85/1455	119/1451	27	38	0.70 (0.53, 0.92)	
EMPA-KIDNEY	36	96/1525	118/1515	32	40	0.78 (0.60, 1.03)	
**Subtotal: DIABETES**	**67**	**3056/40691**	**3233/34113**			**0.77 (0.73, 0.81)**	
**No diabetes**							
DAPA-HF	68	169/1298	227/1307	91	124	0.73 (0.60, 0.89)	
EMPEROR-REDUCED	63	161/936	197/938	139	176	0.78 (0.64, 0.97)	
DELIVER	63	204/1551	246/1558	62	76	0.82 (0.68, 0.99)	p=0.28
EMPEROR-PRESERVED	62	176/1531	220/1519	56	72	0.78 (0.64, 0.95)	
DAPA-CKD	42	15/697	19/701	11	13	0.79 (0.40, 1.55)	
EMPA-KIDNEY	39	35/1779	34/1790	10	9.9	1.04 (0.65, 1.67)	
**Subtotal: NO DIABETES**	**56**	**760/7792**	**943/7813**			**0.79 (0.72, 0.87)**	
**TOTAL: OVERALL**	**65**	**3816/48483**	**4176/41926**			**0.77 (0.74, 0.81)**	

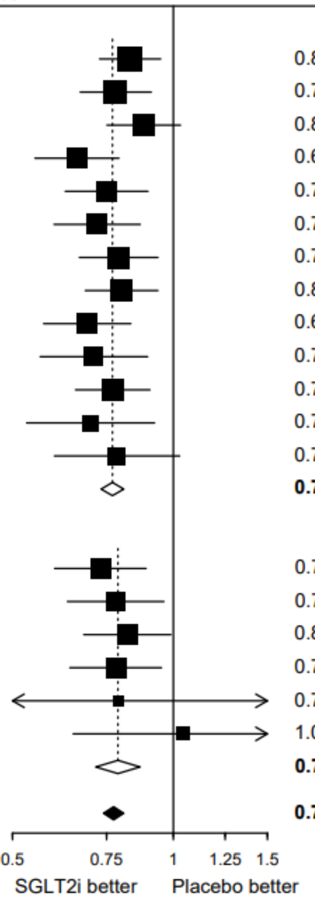

Heterogeneity by diabetes status: p=0.67

Excludes urgent heart failure visits. EMPA-REG OUTCOME cardiovascular death definition excluded stroke. CI = confidence interval; eGFR = estimated glomerular filtration rate; SGLT2i = sodium-glucose cotransporter-2 inhibitor.

Five large trials of SGLT-2 inhibition in heart failure populations have now reported, which together enrolled participants across the spectrum of left ventricular ejection fraction (LVEF). DAPA-HF and EMPEROR-REDUCED demonstrated consistent benefits of SGLT-2 inhibition in people with HFrEF, reducing cardiovascular death or worsening heart failure (including urgent visits in the case of DAPA-HF) by 26%

and 25%, respectively (DAPA-HF: 386/2373 vs 502/2371, HR=0.74, 95%CI 0.65-0.85; EMPEROR-REDUCED: 361/1863 vs 462/1867, HR=0.75, 95%CI 0.65-0.86) (16, 41). In both trials, the beneficial effect of SGLT-2 inhibition was present irrespective of diabetes status or heart failure aetiology (16, 41). Subsequent data from EMPEROR-PRESERVED demonstrated a reduction in cardiovascular death or hospitalisation for heart failure of 21% (415/2997 vs 511/2991, HR=0.79, 95%CI 0.60-0.90) in participants with heart failure with preserved ejection fraction (HFpEF) treated with empagliflozin, compared to placebo (17). The efficacy of SGLT-2 inhibition in HFpEF was confirmed by the DELIVER trial, which demonstrated an 18% reduction in cardiovascular death or worsening heart failure among 6263 participants with heart failure with mildly reduced or preserved ejection fraction randomised to dapagliflozin or placebo (512/3131 vs 610/3132, HR=0.82, 95%CI 0.73-0.92) (2). The beneficial effects of SGLT-2 inhibition in terms of kidney function were present across the range of kidney function all of the in the above trials, which enrolled participants with eGFR down to 20 mL/min/1.73m^2 in the EMPEROR trials, 25 mL/min/1.73m^2 in DELIVER, and 30 mL/min/1.73m^2 in DAPA-HF (13-15, 17).

SOLOIST-WHF tested the dual SGLT-1/-2 inhibitor sotagliflozin in people with recent hospitalisation for worsening heart failure. Sotagliflozin reduced the risk of the trial's revised primary composite of cardiovascular death or total hospitalisations/urgent visits for heart failure by 33% (HR=0.67, 95%CI 0.52-0.85). Benefits were observed irrespective of ejection fraction at recruitment, including those with an ejection fraction ≥50% (28). The use of SGLT-2 inhibition during admission for acute decompensated heart failure (ADHF) represents an area of current interest, and ongoing trials will further clarify the role of SGLT-2 inhibition for this indication. Section 7c discusses the role of SGLT-2 inhibition in ADHF in more detail.

Atherosclerotic cardiovascular disease

For major atherosclerotic/adverse cardiovascular events (MACE), meta-analysis of the key cardiovascular safety trials performed in people with type 2 DM show SGLT-2 inhibitors affords approximately a 10% relative risk reduction compared to placebo (42). Results for the MACE outcome from CREDENCE, DAPA-CKD and SCORED are also consistent with a similar sized relative risk reduction (3, 4, 12), suggesting that the size of relative benefits on MACE are equivalent in people with CKD (Figure 2.7). Benefits on MACE result primarily from reduced risk of cardiovascular death and myocardial infarction with no clear effect on stroke (42).

Figure 2.7: Effects of SGLT-2 inhibitors on MACE by population and trial (3, 4, 12, 21, 29, 39, 40) (adapted from (43))

	Number of events/ participants	Rate per 1000 patient years		Hazard ratio (95% CI)
		SGLT2i	Placebo	
**High cardiovascular risk and type 2 diabetes mellitus**				
EMPA–REG OUTCOME	772/7020	37	44	0.86 (0.74–0.99)
CANVAS Program	1011/10142	27	32	0.86 (0.75–0.97)
DECLARE–TIMI 58	1559/17160	23	24	0.93 (0.84–1.03)
VERTIS CV	980/8238	39	40	0.97 (0.85–1.11)
**Chronic kidney disease**				
CREDENCE	486/4401	39	49	0.80 (0.67–0.95)
DAPA–CKD	275/4304	29	31	0.92 (0.72–1.16)
SCORED	620/10584	40	47	0.84 (0.72–0.99)

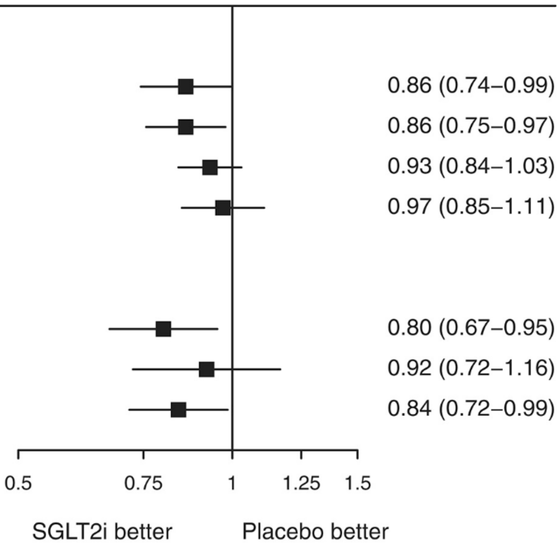

Effect of sodium–glucose co-transporter 2 inhibition on major atherosclerotic/adverse cardiovascular events (MACE), by population. MACE is a composite outcome including cardiovascular death, myocardial infarction or stroke. MACE results from heart failure population trials are unavailable. Rate of MACE was calculated from number of events and other information for SCORED. EMPA-REG OUTCOME also included unstable angina in the composite. VERTIS CV used a non-inferiority population. CI=confidence interval; SGLT2i=sodium–glucose co-transporter 2 inhibitor.

Following the publication of DECLARE-TIMI 58 results (21), it was hypothesised that relative reductions in MACE risk might be larger among people with prior atherosclerotic cardiovascular disease than individuals without (44). However, with the availability of more data from subsequent trials, the evidence of any effect modification by pre-existing disease is less convincing (42). Nevertheless, given the larger relative risk reductions for heart failure than MACE, it is plausible that any cardiovascular deaths which include chronic heart failure in the train of morbid events leading to death may be more likely to be prevented by SGLT-2 inhibition than deaths which are purely atherothrombotic in origin. Recent meta-analyses across different trial populations, including type 2 diabetes at high atherosclerotic cardiovascular risk, have demonstrated that SGLT-2 inhibition reduces the risk of cardiovascular death (11, 45). The totality of the evidence does not confirm benefits on non-cardiovascular death.

2.1.4 Benefits and risks of SGLT-2 inhibition in people with type 2 DM

The benefits of SGLT-2 inhibition in people with type 2 DM and CKD include reduction in the risk of progression of kidney disease, acute kidney injury, and cardiovascular death or hospitalisation for heart failure. However, recognised harms of SGLT-2 inhibition include complications particularly relevant to people with diabetes, including an increase in the risk of diabetic ketoacidosis (DKA), and a potential increase in the risk of lower limb amputation (11). Such medical complications are rare in the populations studied in the completed large placebo-controlled SGLT-2 inhibitor trials, and the estimated absolute benefits of SGLT-2 inhibition in studied individuals clearly outweigh the harms associated with DKA and amputation (Figure 2.8). Further detail regarding risks associated with SGLT-2 inhibition and related recommendations can be found

in Section 5.

Figure 2.8: Absolute benefit and risks of SGLT-2 inhibition for people with CKD with and without diabetes, estimated from 13 large randomised clinical trials of SGLT-2 inhibition (adapted from (11))

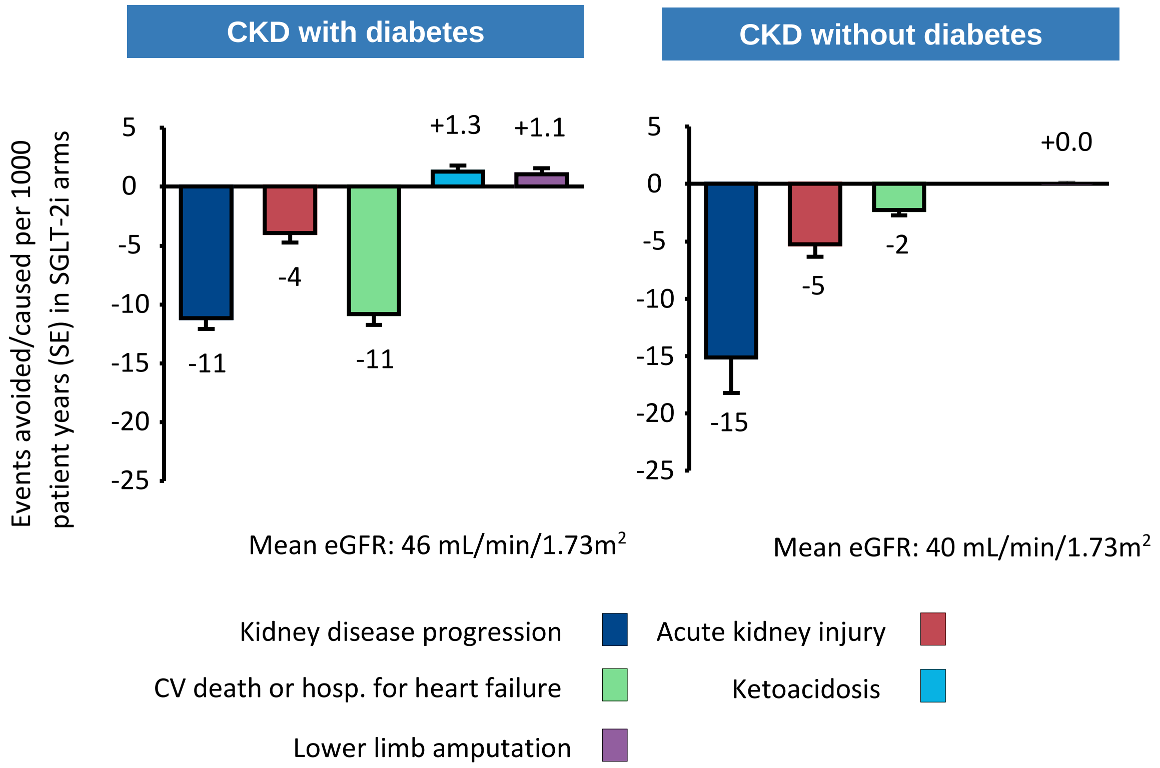

Negative numbers represent events avoided in SGLT-2 inhibitor arms, while positive numbers indicate events caused. CKD – chronic kidney disease. eGFR – estimated glomerular filtration rate. SE – standard error. SGLT-2i – sodium-glucose co-transporter 2 inhibitor.

2.1.5 Summary of trial evidence on glucose-lowering effects

Two pooled analyses have evaluated the effects of SGLT-2 inhibitors on HbA1c by baseline eGFR (46, 47). Both sets of analyses indicate that at lower eGFRs, the effect of SGLT-2 inhibition on HbA1c are diminished, with no good evidence for a clinically meaningful reduction in HbA1c at eGFRs <30 mL/min/1.73m^2. Despite this, there are still beneficial effects of SGLT-2 inhibition on blood pressure, weight and albuminuria at low eGFR. Table 2.2 also highlights several smaller randomised trials (48-52) which have reported similar findings with respect to HbA1c and eGFR, mirroring pharmacodynamics studies showing the linear reduction in measured urinary glucose excretion as eGFR falls (53). Analogous data exist from a trial in post-transplant DM, in which reductions in HbA1c were substantially attenuated, and arguably not clinically meaningful, in those with an eGFR <45 mL/min/1.73m^2 (54) (see section 7b for more details). The large placebo-controlled trials of SGLT-2 inhibition in CKD have demonstrated the expected modest effects among people with diabetes, with estimated reductions in HbA1c attenuated at low eGFR to 0.31% (95%CI 0.26-0.37) in CREDENCE, 0.42% (95%CI 0.38-0.47) in SCORED, 0.1% (95%CI 0.0-0.2) in DAPA-CKD, and 0.08% (95%CI 0.15- -0.01) in EMPA-KIDNEY (1, 3, 12, 55). Section 5 of this guideline provides more detail on management of hypoglycaemic agents in people initiating SGLT-2 inhibition, and on the risks of hypoglycaemia.

Table 2.2: Randomised trial results assessing the effect of SGLT-2 inhibitors on %HbA1c reductions by level of kidney function (46-52)

**POOLED ANALYSES**				
**Author, year (No. participants & trials)**	**SGLT-2- inhibitor**	**Duration**	**eGFR range mL/min/1.73m²**	**Change in %HbA1c compared to placebo (95%CI or SE)**
Cherney et al., 2017 (n=2286, 11 trials)	Empagliflozin	24 weeks	≥90<br>≥60 to <90<br>≥30 to <60<br><30	-0.84% (-0.95, -0.72)<br>-0.60% (-0.70, 0.51)<br>-0.38% (-0.52, -0.24)<br>-0.04% (-0.37, -0.29)
Petrikyv et al., 2017 (n=4404, 11 trials)	Dapagliflozin	24 weeks	≥90<br>≥60 to <90<br>≥45 to <60	-0.57% (-0.66, -0.47)<br>-0.47% (-0.54, -0.40)<br>-0.27% (-0.43, -0.11)
**TRIALS**				
**Author, year (No. participants & trials)**	**SGLT-2- inhibitor**	**Duration**	**eGFR range mL/min/1.73m²**	**Change in %HbA1c compared to placebo (95%CI or SE)**
Allegretti et al., 2019 (n=312)	Bexagliflozin	24 weeks	45 to <60<br>30 to <45	-0.31% (-0.09, -0.53)<br>-0.43% (-0.16, -0.69)
Barnett et al., 2014 (n=290)	Empagliflozin	24 weeks	≥60 to <90<br>≥30 to <60<br>15 to <30	-0.68% (-0.88, -0.49)<br>-0.42% (-0.56, -0.28)<br>No reduction
Fioretto et al., 2014 (n=321)	Dapagliflozin	24 weeks	45 to <60	-0.34% (-0.53, -0.15)
Kohan et al., 2014 (n=252)	Empagliflozin	24 weeks	All: <60<br>≥45 to <60<br>≥30 to <45	-0.32% (SE 0.17)<br>-0.33% (SE 0.24)<br>0.07% (SE 0.21)
Yale et al., 2014 (n=269)	Canagliflozin	52 weeks	≥30 to <50	-0.41% (-0.68, -0.14)

eGFR= estimated glomerular filtration rate; HbA1c= glycosylated haemoglobin; CI=confidence interval; SE=standard error

ABCD-RA Clinical Practice Guidelines for Management of Hyperglycaemia in Adults with Diabetic Kidney Disease: 2021 Update

This SGLT-2 inhibitor guideline does not consider glycaemic targets, as the cardiac and kidney benefits of SGLT-2 inhibition appear to be preserved in those with CKD and an eGFR ≥20 mL/min/1.73m² (1, 3, 4), despite their attenuated effect on blood glucose lowering in CKD (46). Nevertheless, we recognise that the care of people with diabetic kidney disease is often shared between nephrology and diabetes clinical teams, as highlighted by the 2021 joint ABCD-RA guideline: Clinical Practice Guidelines for Management of Hyperglycaemia in Adults with Diabetic Kidney Disease (56). We therefore provide a brief summary of the joint ABCD-RA's considerations and recommendations in the text and Table 2.3 below.

The ABCD-RA guideline group recognised that early intensive diabetic management leads to reduction in risk of subsequent diabetic kidney disease. A meta-analysis of randomised trials has demonstrated that more

intensive glycaemic control reduces the risk of a composite kidney outcome of ESKD, renal death, decline in eGFR to <30 mL/min/1.73m^2 or development of macroalbuminuria (A3), by about 20% compared to more standard control (HR=0.80, 95%CI 0.72-0.88) (57). About two-thirds of this result constituted the albuminuria-based component of the composite, with more limited information available for the eGFR and ESKD-based components. This contrasts the quality of information available in kidney outcome trials of SGLT-2 inhibition, which had larger numbers of eGFR-based and kidney failure outcomes and larger relative risk reductions than the intensive glycaemic control trials (1, 3, 4).

The ABCD-RA group highlighted that there are challenges with respect to increased risk of hypoglycaemia (both treatment and kidney-related) and reliability of HbA1c monitoring required to achieve intensive glycaemic control in people with moderate-to-advanced CKD. They therefore provide glycaemic targets stratified by age, CKD stage and diabetic therapy to try and safely achieve tight glycaemic control targets in people with diabetic kidney disease.

Table 2.3: Summary of the glycaemic recommendations for people with type 2 DM and CKD adapted from the ABCD-RA clinical practice guidelines for management of hyperglycaemia in adults with diabetic kidney disease: 2021 update (56)

Glycaemic target mmol/mol (%HbA1c)	CKD stage	Age & anti-diabetic regimen
**Glycaemic targets should be individualised**		
48-58 mmol/mol (6.5-7.5%)<br>Aim <52 mmol/mol (6.9%)	1-2	- <40 years<br>- Any age, if diet controlled*
52-58 mmol/mol (6.9-7.5%)	3-4	- Any age treated with a predominately oral hypoglycaemic regimen (i.e. non- insulin dominant)
58-68 mmol/mol (7.5-8.5%)	3-4	- Any age on an insulin-dominant regimen (aim 58 mmol/mol).<br>- Age >75 years with stage 4 CKD on any regimen
58-68 mmol/mol (7.5-8.5%)	5, including dialysis	- Any age or any regimen

* Aim for HbA1c 58 mmol/mol if hypoglycaemia and/or anaemia occurs, and consider blood glucose or flash glucose monitoring. CKD= chronic kidney disease

2.1.6 Quality of the evidence

For the large SGLT-2 inhibitor trials providing the majority of evidence underpinning our guidance, risk of bias is low, as assessed using the Cochrane Risk of Bias 2 (ROB2) tool. All trials employed strong randomization and blinding procedures, and compared efficacy to matching placebo. Intention-to-treat analyses were used (with modifications where appropriate for safety outcomes), and clearly-defined testing processes for secondary outcomes were used, minimising the risk of bias in all assessed domains. Their size and large number of outcomes enable precise estimates of treatment effect and ability to assess effects in subpopulations (particularly when the trials are combined in meta-analysis by diabetes status).

Although risk of bias was low across assessed ROB2 domains for included trials, potential small sources of bias remain. CREDENCE, DAPA-CKD and EMPA-KIDNEY were closed early, which may lead to over-estimation of the relative treatment effects (particularly where the data was locked shortly after interim review). However, point estimates for the kidney disease progression outcomes from the other completed trials of SGLT-2 inhibitors are broadly consistent with those identified in these trials. The key limitation of the trials was the short duration of follow-up for participants with low levels of albuminuria, meaning assessments of effects on CKD progression are limited to eGFR slope-based analyses in some groups. The two sotagliflozin trials (SCORED and SOLOIST-WHF) underwent modification of their primary assessments prior to unblinding (12, 28). Due to withdrawal of funding, both trials were also unable to complete endpoint adjudication, instead relying on investigator-reported events. In SOLOIST-WHF, 73% of events that were sent for adjudication matched the events reported by investigators (balanced between trial arms) (28).

2.1.7 Summary of published cost-effectiveness analyses

We identified two UK-specific cost-effectiveness analyses of SGLT-2 inhibition in CKD. The CREDEM-CKD group estimated UK-specific cost-effectiveness in diabetic kidney disease based on extrapolations from CREDENCE data (58). In their primary analysis, a gain of 0.28 quality-adjusted life years (QALYs) per individual treated with canagliflozin for 10 years was found. This benefit was determined primarily by longer survival in the canagliflozin arm and reduced progression through CKD stages. Cost-effectiveness was driven, in large part, by reduced need for dialysis. Canagliflozin was associated with overall cost savings of £4,706 per individual over the course of 10 years compared to placebo. Savings on reductions in cardiovascular risk were largely offset by greater costs due to longer survival.

A cost-effectiveness analysis conducted by the DAPA-CKD group modelled the cost-effectiveness of dapagliflozin use in CKD in health systems in the UK, Germany, and Spain using individual participant data from DAPA-CKD. Based on the UK-specific analysis, there was a predicted lifetime gain 0.82 QALYs (95% credible interval [CrI] 0.34-1.17), with a corresponding cost of £6,020/QALY (59). Individuals were predicted to spend more time in the eGFR range 89-15 mL/min/1.73m^2, and to have a longer life expectancy. Increased costs were driven by drug acquisition costs and increased costs of CKD management (due to the greater life expectancy afforded by dapagliflozin), but were offset by reduction in hospitalisations for heart failure, abrupt declines in kidney function, and progression to kidney failure. Similar to the CREDEM-CKD analysis, analyses of fewer than 10 years duration demonstrated dominance of dapagliflozin over placebo (i.e. dapagliflozin was both less costly and more effective than placebo).

2.2 Recommendations for use

Due to the benefits of SGLT-2 inhibitors on kidney outcomes (CKD and AKI) and cardiovascular risk:

1. **We recommend initiating SGLT-2 inhibition in people with chronic kidney disease and type 2 diabetes, irrespective of primary kidney disease,* for any of the following 4 clinical scenarios (Grade 1A):**
   - **(a) eGFR of 20-45 mL/min/1.73m^2**
   - **(b) eGFR of >45 mL/min/1.73m^2 and a urinary albumin-to-creatinine ratio (uACR) of ≥25 mg/mmol†**
   - **(c) Symptomatic heart failure, irrespective of ejection fraction**
   - **(d) Established coronary disease**

*** excludes people with polycystic kidney disease, type 1 diabetes, or a kidney transplant**
† urinary protein-to-creatinine ratio of 35 mg/mmol can be considered equivalent

Rationale: CREDENCE, DAPA-CKD and EMPA-KIDNEY have consistently shown that SGLT-2 inhibition significantly and importantly reduces the risk of progression of CKD in a broad CKD population, including in people with diabetic kidney disease and kidney disease of non-diabetic aetiology. SGLT-2 inhibitors also reduce risk of acute kidney injury and cardiovascular disease. Participants were enrolled down to an eGFR of 20 mL/min/1.73m^2, and there is no evidence that the beneficial effects of SGLT-2 inhibition on kidney disease progression or cardiovascular risk are attenuated across the spectrum of eGFR studied. In EMPA-KIDNEY, participants were enrolled with eGFR 20-45 mL/min/1.73m^2 irrespective of uACR, or eGFR >45 mL/min/1.73m^2 with uACR ≥23 mg/mmol. EMPA-KIDNEY and DAPA-CKD both demonstrated consistent benefits irrespective of primary kidney diagnosis subdivided by diabetic kidney disease, ischaemic and hypertensive nephropathy, and glomerular disease. Among non-albuminuric kidney disease, data from eGFR slope analyses in EMPA-KIDNEY demonstrates reductions in rate of eGFR decline of a magnitude that would be expected to translate into meaningful reductions in progression of CKD, which is supported by similar findings in eGFR slope analyses from EMPEROR-REDUCED, EMPEROR-PRESERVED, DAPA-HF and DELIVER. We therefore provide grade 1A recommendation for use in people with eGFR 20-45 mL/min/1.73m^2 or with eGFR ≥45 mL/min/1.73m^2 and uACR ≥25 mg/mmol. Note that the 25 mg/mmol threshold for uACR was chosen pragmatically, given that this value is expected to be easier to recall for practicing clinicians than a cut-off of 23 mg/mmol, as is commonly used in the CKD trials.

Consistent findings from five SGLT-2 inhibitor trials in people with symptomatic heart failure have demonstrated that SGLT-2 inhibition reduces the risk of cardiovascular death or hospitalisation for heart failure among this population. These findings have been demonstrated in people with reduced and preserved ejection fraction, and in people with recent hospitalisation for worsening heart failure. There has been no evidence that the cardiac benefits of SGLT-2 inhibition are modified by diabetes status or by eGFR. Among CKD populations, cardiovascular death or hospitalisation for heart failure has been shown to be reduced by SGLT-2 inhibition in CREDENCE, SCORED and DAPA-CKD. The totality of evidence indicates a reduction of these outcomes of approximately one quarter in people treated with SGLT-2 inhibition compared to placebo. We therefore provide grade 1A recommendation for use in people with CKD for this indication. Those with prior coronary disease are at high risk of MACE and heart failure and are included in this recommendation based on the totality of the evidence (see Figure 2.7).

2. **We suggest initiating SGLT-2 inhibition to modify cardiovascular risk and slow rate of kidney function decline in people with an eGFR >45-60 mL/min/1.73m^2 and a uACR of <25 mg/mmol, recognising effects on glycaemic control will be limited (Grade 2B)**

Rationale: Meta-analysis of large randomised clinical trials of SGLT-2 inhibition indicates that cardiovascular benefits of SGLT-2 inhibition, particularly reducing the risk of cardiovascular death or hospitalisation for heart failure, are present irrespective of trial-level average eGFR. The benefits of SGLT-2 inhibition in terms of reducing progression of kidney disease are also not modified by eGFR in individual trials. eGFR slope analyses from heart failure and CKD trials consistently demonstrate reduced rates of kidney function decline among people treated with SGLT-2 inhibition, including those without significant albuminuria, as demonstrated in EMPA-KIDNEY. EMPEROR-REDUCED, EMPEROR-PRESERVED, DAPA-HF and DELIVER all show reductions in the rate of kidney function decline with SGLT-2 inhibition compared to placebo, while in EMPEROR-REDUCED this reduction in rate of eGFR decline is present in the presence and absence of CKD and across the spectrum of albuminuria. Therefore, while this population has not been directly studied in randomised trials, there is indirect evidence to support reduction in cardiovascular risk and reduced rate of kidney function decline in this group, for which we provide a grade 2B recommendation.

3. **We suggest clinicians consider initiating SGLT-2 inhibition in people with an eGFR below 20 mL/min/1.73m^2 to slow progression of kidney disease (Grade 2B)**

Rationale: Clinical trials of SGLT-2 inhibition conducted in populations with CKD have continued SGLT-2 inhibition until the initiation of kidney replacement therapy, providing indirect evidence to support the use of this SGLT-2 inhibition in this population. Furthermore, data from EMPA-KIDNEY indicates that the benefit of SGLT-2 inhibition in terms of progression of kidney disease is not attenuated in people with an eGFR <20 mL/min/1.73m^2. There is no suggestion from the data that kidney benefits of SGLT-2 inhibition begin to attenuate down to an eGFR of 15 mL/min/1.73m^2, and those with decreased eGFR are at particularly high risk. We therefore provide a 2B recommendation for use in people with eGFR <20 mL/min/1.73m^2.

Section 2: Recommendations for Use Footnote

All recommendations in sections 2 & 3 exclude people with type 1 DM (see section 7a) and exclude those with a kidney transplant (see section 7b).

2.3 Clinical research recommendations

We recommend further research including, wherever possible, randomised trials to establish definitively:

1. The effects of SGLT-2 inhibition on cardiac and kidney outcomes in people with polycystic kidney disease.
2. Safety, cardiovascular and kidney effects of SGLT-2 inhibition on kidney outcomes in people with a functioning kidney transplant (see section 7b).
3. Pharmacokinetics, cardiovascular effects and residual kidney function preservation effects of SGLT-2

inhibition in people on dialysis.

4. The safety and efficacy of adding MRA to SGLT-2 inhibition in people with CKD (particularly non-steroidal MRAs with proven cardiovascular and kidney-related benefits).

5. The safety and efficacy of combining SGLT-2 inhibition with a glucagon-like peptide-1 (GLP-1) receptor agonists in people with CKD.

6. Detailed cost effectiveness analyses of SGLT-2 inhibition in CKD considering effects across the full range of eGFR and uACR categories.

2.4 Recommendations for implementation

1. We recommend using SGLT-2 inhibitors with demonstrated efficacy for their given indications (Grade 1A)

Rationale: Government regulators review data from randomised trials and assess their reliability through regulatory inspections. Regulatory licences/indications therefore provide a key guide to which SGLT-2 inhibitors have generated definitive evidence of efficacy and safety for a given use. We therefore recommend selecting SGLT-2 inhibitors according to these licensed indications, wherever possible (summaries of which are provided in section 4).

2. We recommend using clinically appropriate single agent renin-angiotensin system (RAS) blockade in combination with SGLT-2 inhibition, wherever RAS blockade is indicated and tolerated (Grade 1A)

Rationale: These clinical practice guidelines pertain to use of SGLT-2 inhibition in people with CKD. The standard of care in many forms of CKD is the use of RAS blockers (60, 61), with clear evidence of benefit in diabetic nephropathy (62, 63). All CREDENCE participants were on stable maximally tolerated RAS blockade (3), as were 97% of DAPA-CKD participants (4) and 85% of participants in EMPA-KIDNEY (1). We therefore provide a grade 1A recommendation to prescribe RAS blockade and ensure clinically appropriate dosing alongside any SGLT-2 inhibitor use. Note that it has been suggested that, mechanistically, SGLT-2 inhibition may have the potential to activate RAS (64). However the large trials in people with type 2 DM at high atherosclerotic cardiovascular risk have been combined in meta-analysis and have raised a hypothesis that the benefits of SGLT-2 inhibitors on kidney disease progression could extend to in people with type 2 DM not on RAS blockade (22). The lack of heterogeneity of effect of empagliflozin on the primary composite outcome stratified by use of RAS inhibition in EMPA-KIDNEY lends further support to this hypothesis (1).

Note that we recommend single agent RAS blockade, as combination therapy (i.e. dual blockade with ACEi plus ARB) has been found to increase the risk of serious hyperkalaemia or acute kidney injury, and has not been shown to importantly slow CKD progression (36).

3. We suggest following NICE guidelines on use of uACR for screening of albuminuria (NICE NG203). We recognise that more pragmatic approaches to identifying risk of kidney disease progression may be necessary whilst local access to uACR measurement is improved (Grade 2C)

Rationale: Many factors can cause transient increases in albuminuria (including urinary tract infection, exercise, and menstruation) and as such, NICE (65) and other international guideline groups (66) recommend that repeat testing should take place within 3 months if a single uACR result is between 3-69 mg/mmol. An early morning sample offers some advantages due to reduced impact of hydration status and exercise (67), but if unavailable, random sampling may still offer a reliable indication of total daily albuminuria (68). A uACR value ≥70 mg/mmol generally does not require further confirmation, as this is consistent with clinically significant proteinuria (69).

We agree with the statement within the NICE CKD guidelines that reagent strips and PCR measurements should not be used to quantify albuminuria (65). Large-scale meta-analysis and other observational data have shown that dipstick values using reagent strips are neither sensitive, nor specific enough to predict uACR accurately (70). However, we recognise that uACR testing may not be regularly undertaken in some areas of the UK, and local methods of assessing risk may need to be used to ensure those at risk are offered SGLT-2 inhibition.

4. We recommend that SGLT-2 inhibition can be continued until the need for dialysis or kidney transplantation arises (Grade 1A)

Rationale: Data from CKD trials include many hundreds of participants with an eGFR below 20 mL/min/1.73m^2. Continued use of SGLT-2 inhibitors until the need for dialysis or kidney transplantation was the practice in these trials, which have confirmed benefits exceed any harms. In subgroup analyses, kidney benefits are unmodified by baseline eGFR. At a population level, people with a low eGFR are at highest absolute risk of kidney failure, and are therefore most likely to benefit (in absolute terms).

5. We suggest that co-prescription of SGLT-2 inhibition with MRA can be considered, where each are individually indicated (Grade 2B)

Rationale: Subgroup analyses from the SGLT-2 inhibitor trials in non-CKD populations suggest cardiac and kidney benefits are likely to be maintained in people co-prescribed an MRA with an SGLT-2 inhibitor, with no increased risk of hyperkalaemia caused by SGLT-2 inhibitor use. CREDENCE, DAPA-CKD and EMPA-KIDNEY provide reassuring evidence that SGLT-2 inhibition does not usually cause hyperkalaemia in CKD populations. We therefore provide a grade 2B suggestion that MRA can be used with SGLT-2 inhibitors. Note that guidance on how to monitor for changes in eGFR and potassium in those on MRA are outside of the scope of this guideline.

6. We suggest the beneficial effects of SGLT-2 inhibition on kidney disease progression or risk of heart failure hospitalisation are likely to be a class effect (Grade 2B)

Rationale: We have recommended using SGLT-2 inhibitors with demonstrated efficacy for their given indications, but as more large trials report results testing the available SGLT-2 inhibitors in overlapping populations, it is increasingly apparent that any differences between the individual molecules do not appear to create large differences in clinical efficacy. For example, CREDENCE (canagliflozin), DAPA-CKD (dapagliflozin) and EMPA-KIDNEY (empagliflozin) reported relative risk reductions on their respective kidney disease progression outcomes which were comparable in their respective (sub)populations with type 2 DM

(1, 3, 4, 11, 71). Beneficial effects on cardiovascular death or hospitalisation for heart failure were consistent in the CKD trials with the totality of evidence across all SGLT-2 inhibitor trials, including those in heart failure-specific populations (11). Likewise, the HFrEF trials DAPA-HF (dapagliflozin) and EMPEROR-REDUCED (empagliflozin), and the HFpEF trials DELIVER (dapagliflozin) and EMPEROR-PRESERVED (empagliflozin), share similar designs and results of primary and secondary assessments overall and across subgroups are remarkably consistent (34).

Relative risk reductions on MACE across key cardiovascular safety trials (42) and trials in dedicated CKD populations are also not statistically different from each other (4, 12). Meta-analyses demonstrate consistent benefits of SGLT-2 inhibition in terms of cardiovascular and kidney benefits without apparent heterogeneity by class overall and stratified by diabetes status (11, 45). We are of the opinion that the larger effects of empagliflozin on cardiovascular death in EMPA-REG OUTCOME (39), and the larger effects on non-cardiovascular death in DAPA-CKD compared to other SGLT-2 inhibitor trials (19, 45) are more likely represent the play of chance or be caused by factors other than minor differences in the biological action of different SGLT-2 inhibitors. We therefore suggest there is increasing evidence that the cardiac and kidney benefits of SGLT-2 inhibition represent a class effect.

It should be noted, however, that SGLT-2 inhibitors differ in their respective receptor selectivity and there may be an increased propensity to cause diarrhoea and volume depletion when using SGLT-2 inhibitors that also meaningfully inhibit gut SGLT-1 (e.g. sotagliflozin (28)). Selectivity for SGLT-2 over SGLT-1 ranges from: ~20:1 for the dual SGLT-1/2 inhibitor sotagliflozin (72), and from ~250:1 for canagliflozin to ~2500:1 for empagliflozin (73) for the more selective SGLT-2 inhibitors.

2.5 Audit measures

We propose the following audit measures focusing on those guidelines supported by robust randomised evidence:

1. The proportion of people with each grade 1 recommendation for use prescribed an SGLT-2 inhibitor (with exploration of reasons for non-use to direct quality improvement projects).

2. The proportion of people prescribed an SGLT-2 inhibitor not on concomitant RAS blockade.

2.6 References

1. EMPA-KIDNEY Collaborative Group. Empagliflozin in Patients with Chronic Kidney Disease. New England Journal of Medicine. 2022.
2. Solomon SD, McMurray JJV, Claggett B, de Boer RA, DeMets D, Hernandez AF, et al. Dapagliflozin in Heart Failure with Mildly Reduced or Preserved Ejection Fraction. New England Journal of Medicine. 2022;387(12):1089-98.
3. Perkovic V, Jardine MJ, Neal B, Bompoint S, Heerspink HJL, Charytan DM, et al. Canagliflozin and Renal Outcomes in Type 2 Diabetes and Nephropathy. N Engl J Med. 2019;380(24):2295-306.
4. Heerspink HJL, Stefansson BV, Correa-Rotter R, Chertow GM, Greene T, Hou FF, et al. Dapagliflozin in Patients with Chronic Kidney Disease. N Engl J Med. 2020;383(15):1436-46.
5. Coresh J, Turin TC, Matsushita K, Sang Y, Ballew SH, Appel LJ, et al. Decline in estimated glomerular filtration rate and subsequent risk of end-stage renal disease and mortality. Jama. 2014;311(24):2518-31.
6. Levey AS, Inker LA, Matsushita K, Greene T, Willis K, Lewis E, et al. GFR decline as an end point for clinical trials in CKD: a scientific workshop sponsored by the National Kidney Foundation and the US Food and Drug Administration. American journal of kidney diseases : the official journal of the National Kidney Foundation. 2014;64(6):821-35.
7. Levey AS, Gansevoort RT, Coresh J, Inker LA, Heerspink HL, Grams ME, et al. Change in Albuminuria and GFR as End Points for Clinical Trials in Early Stages of CKD: A Scientific Workshop Sponsored by the National Kidney Foundation in Collaboration With the US Food and Drug Administration and European Medicines Agency. American journal of kidney diseases : the official journal of the National Kidney Foundation. 2020;75(1):84-104.
8. Wanner C, Inzucchi SE, Lachin JM, Fitchett D, von Eynatten M, Mattheus M, et al. Empagliflozin and Progression of Kidney Disease in Type 2 Diabetes. N Engl J Med. 2016;375(4):323-34.
9. Herrington WG, Preiss D, Haynes R, von Eynatten M, Staplin N, Hauske SJ, et al. The potential for improving cardio-renal outcomes by sodium-glucose co-transporter-2 inhibition in people with chronic kidney disease: a rationale for the EMPA-KIDNEY study. Clin Kidney J. 2018;11(6):749-61.
10. Wheeler DC, Stefansson BV, Batiushin M, Bilchenko O, Cherney DZI, Chertow GM, et al. The dapagliflozin and prevention of adverse outcomes in chronic kidney disease (DAPA-CKD) trial: baseline characteristics. Nephrol Dial Transplant. 2020;35(10):1700-11.
11. Nuffield Department of Population Health Renal Studies Group and the SGLT2 inhibitor Meta-Analysis Cardio-Renal Trialists' Consortium. Impact of diabetes on the effects of sodium glucose co-transporter-2 inhibitors on kidney outcomes: collaborative meta-analysis of large placebo-controlled trials. The Lancet. 2022;400(10365):1788-801.
12. Bhatt DL, Szarek M, Pitt B, Cannon CP, Leiter LA, McGuire DK, et al. Sotagliflozin in Patients with Diabetes and Chronic Kidney Disease. N Engl J Med. 2021;384(2):129-39.
13. Mc Causland FR, Claggett BL, Vaduganathan M, Desai AS, Jhund P, de Boer RA, et al. Dapagliflozin and Kidney Outcomes in Patients With Heart Failure With Mildly Reduced or Preserved Ejection Fraction: A Prespecified Analysis of the DELIVER Randomized Clinical Trial. JAMA Cardiology. 2022.
14. Zannad F, Ferreira JP, Pocock SJ, Zeller C, Anker SD, Butler J, et al. Cardiac and Kidney Benefits of Empagliflozin in Heart Failure Across the Spectrum of Kidney Function: Insights From EMPEROR-Reduced. Circulation. 2021;143(4):310-21.
15. Jhund PS, Solomon SD, Docherty KF, Heerspink HJL, Anand IS, Bohm M, et al. Efficacy of Dapagliflozin on Renal Function and Outcomes in Patients With Heart Failure With Reduced Ejection Fraction:

Results of DAPA-HF. Circulation. 2021;143(4):298-309.

16. Packer M, Anker SD, Butler J, Filippatos G, Pocock SJ, Carson P, et al. Cardiovascular and Renal Outcomes with Empagliflozin in Heart Failure. N Engl J Med. 2020;383(15):1413-24.

17. Anker SD, Butler J, Filippatos G, Ferreira JP, Bocchi E, Böhm M, et al. Empagliflozin in Heart Failure with a Preserved Ejection Fraction. New England Journal of Medicine. 2021;385(16):1451-61.

18. Matthews JNS, Altman DG. Statistics Notes: Interaction 2: compare effect sizes not P values. BMJ. 1996;313(7060):808.

19. Heerspink HJL, Sjöström CD, Jongs N, Chertow GM, Kosiborod M, Hou FF, et al. Effects of dapagliflozin on mortality in patients with chronic kidney disease: a pre-specified analysis from the DAPA-CKD randomized controlled trial. Eur Heart J. 2021;42(13):1216-27.

20. The RENAL LIFECYCLE Trial: A RCT to Assess the Effect of Dapagliflozin on Renal and Cardiovascular Outcomes in Patients With Severe CKD: U.S. National Library of Medicine; [Available from: https://clinicaltrials.gov/ct2/show/NCT05374291].

21. Wiviott SD, Raz I, Bonaca MP, Mosenzon O, Kato ET, Cahn A, et al. Dapagliflozin and Cardiovascular Outcomes in Type 2 Diabetes. N Engl J Med. 2019;380(4):347-57.

22. Neuen BL, Young T, Heerspink HJL, Neal B, Perkovic V, Billot L, et al. SGLT2 inhibitors for the prevention of kidney failure in patients with type 2 diabetes: a systematic review and meta-analysis. Lancet Diabetes Endocrinol. 2019;7(11):845-54.

23. Bakris GL, Agarwal R, Anker SD, Pitt B, Ruilope LM, Rossing P, et al. Effect of Finerenone on Chronic Kidney Disease Outcomes in Type 2 Diabetes. N Engl J Med. 2020;383(23):2219-29.

24. Pitt B, Filippatos G, Agarwal R, Anker SD, Bakris GL, Rossing P, et al. Cardiovascular Events with Finerenone in Kidney Disease and Type 2 Diabetes. New England Journal of Medicine. 2021;385(24):2252-63.

25. Seferovic PM, Fragasso G, Petrie M, Mullens W, Ferrari R, Thum T, et al. Sodium-glucose co-transporter 2 inhibitors in heart failure: beyond glycaemic control. A position paper of the Heart Failure Association of the European Society of Cardiology. Eur J Heart Fail. 2020;22(9):1495-503.

26. Rossing P, Caramori ML, Chan JCN, Heerspink HJL, Hurst C, Khunti K, et al. Executive summary of the KDIGO 2022 Clinical Practice Guideline for Diabetes Management in Chronic Kidney Disease: an update based on rapidly emerging new evidence. Kidney Int. 2022;102(5):990-9.

27. Provenzano M, Jongs N, Vart P, Stefánsson BV, Chertow GM, Langkilde AM, et al. The Kidney Protective Effects of the Sodium–Glucose Cotransporter-2 Inhibitor, Dapagliflozin, Are Present in Patients With CKD Treated With Mineralocorticoid Receptor Antagonists. Kidney International Reports. 2022;7(3):436-43.

28. Bhatt DL, Szarek M, Steg PG, Cannon CP, Leiter LA, McGuire DK, et al. Sotagliflozin in Patients with Diabetes and Recent Worsening Heart Failure. N Engl J Med. 2021;384(2):117-28.

29. Cannon CP, Pratley R, Dagogo-Jack S, Mancuso J, Huyck S, Masiukiewicz U, et al. Cardiovascular Outcomes with Ertugliflozin in Type 2 Diabetes. N Engl J Med. 2020;383(15):1425-35.

30. Docherty KF, Jhund PS, Inzucchi SE, Kober L, Kosiborod MN, Martinez FA, et al. Effects of dapagliflozin in DAPA-HF according to background heart failure therapy. Eur Heart J. 2020;41(25):2379-92.

31. Ferreira JP, Zannad F, Pocock SJ, Anker SD, Butler J, Filippatos G, et al. Interplay of Mineralocorticoid Receptor Antagonists and Empagliflozin in Heart Failure: EMPEROR-Reduced. J Am Coll Cardiol. 2021;77(11):1397-407.

32. Shen L, Kristensen SL, Bengtsson O, Bohm M, de Boer RA, Docherty KF, et al. Dapagliflozin in HFrEF

Patients Treated With Mineralocorticoid Receptor Antagonists: An Analysis of DAPA-HF. JACC Heart Fail. 2021;9(4):254-64.

33. Ferreira JP, Butler J, Zannad F, Filippatos G, Schueler E, Steubl D, et al. Mineralocorticoid Receptor Antagonists and Empagliflozin in Patients With Heart Failure and Preserved Ejection Fraction. Journal of the American College of Cardiology. 2022;79(12):1129-37.

34. Vaduganathan M, Docherty KF, Claggett BL, Jhund PS, de Boer RA, Hernandez AF, et al. SGLT-2 inhibitors in patients with heart failure: a comprehensive meta-analysis of five randomised controlled trials. The Lancet. 2022;400(10354):757-67.

35. A Study to Learn How Well the Treatment Combination of Finerenone and Empagliflozin Works and How Safe it is Compared to Each Treatment Alone in Adult Participants With Long-term Kidney Disease (Chronic Kidney Disease) and Type 2 Diabetes (CONFIDENCE) [Available from: https://clinicaltrials.gov/ct2/show/NCT05254002].

36. Fried LF, Emanuele N, Zhang JH, Brophy M, Conner TA, Duckworth W, et al. Combined angiotensin inhibition for the treatment of diabetic nephropathy. N Engl J Med. 2013;369(20):1892-903.

37. Weir MR, Slee A, Sun T, Balis D, Oh R, de Zeeuw D, Perkovic V. Effects of canagliflozin on serum potassium in the CANagliflozin cardioVascular Assessment Study (CANVAS) Program. Clinical Kidney Journal, sfaa133, https://doi.org/10.1093/ckj/sfaa133 (accessed 2nd January 2021).

38. Neuen BL, Oshima M, Agarwal R, Arnott C, Cherney DZ, Edwards R, et al. Sodium-Glucose Cotransporter 2 Inhibitors and Risk of Hyperkalemia in People With Type 2 Diabetes: A Meta-Analysis of Individual Participant Data From Randomized, Controlled Trials. Circulation. 2022;145(19):1460-70.

39. Zinman B, Wanner C, Lachin JM, Fitchett D, Bluhmki E, Hantel S, et al. Empagliflozin, Cardiovascular Outcomes, and Mortality in Type 2 Diabetes. N Engl J Med. 2015;373(22):2117-28.

40. Neal B, Perkovic V, Mahaffey KW, de Zeeuw D, Fulcher G, Erondu N, et al. Canagliflozin and Cardiovascular and Renal Events in Type 2 Diabetes. N Engl J Med. 2017;377(7):644-57.

41. McMurray JJV, Solomon SD, Inzucchi SE, Kober L, Kosiborod MN, Martinez FA, et al. Dapagliflozin in Patients with Heart Failure and Reduced Ejection Fraction. N Engl J Med. 2019;381(21):1995-2008.

42. McGuire DK, Shih WJ, Cosentino F, Charbonnel B, Cherney DZI, Dagogo-Jack S, et al. Association of SGLT2 Inhibitors With Cardiovascular and Kidney Outcomes in Patients With Type 2 Diabetes: A Meta-analysis. JAMA Cardiol. 2021;6(2):148-58.

43. Herrington WG, Savarese G, Haynes R, Marx N, Mellbin L, Lund LH, et al. Cardiac, renal, and metabolic effects of sodium-glucose co-transporter-2 inhibitors: a position paper from the European Society of Cardiology ad-hoc task force on sodium-glucose co-transporter-2 inhibitors European Journal of Heart Failure (in Press). 2021.

44. Zelniker TA, Wiviott SD, Raz I, Im K, Goodrich EL, Bonaca MP, et al. SGLT2 inhibitors for primary and secondary prevention of cardiovascular and renal outcomes in type 2 diabetes: a systematic review and meta-analysis of cardiovascular outcome trials. Lancet. 2019;393(10166):31-9.

45. Staplin N, Roddick AJ, Emberson J, Reith C, Riding A, Wonnacott A, et al. Net effects of sodium-glucose co-transporter-2 inhibition in different patient groups: a meta-analysis of large placebo-controlled randomized trials. eClinicalMedicine. 2021;41.

46. Cherney DZI, Cooper ME, Tikkanen I, Pfarr E, Johansen OE, Woerle HJ, et al. Pooled analysis of Phase III trials indicate contrasting influences of renal function on blood pressure, body weight, and HbA1c reductions with empagliflozin. Kidney Int. 2018;93(1):231-44.

47. Petrykiv S, Sjostrom CD, Greasley PJ, Xu J, Persson F, Heerspink HJL. Differential Effects of Dapagliflozin on Cardiovascular Risk Factors at Varying Degrees of Renal Function. Clinical journal of

the American Society of Nephrology : CJASN. 2017;12(5):751-9.

48. Yale JF, Bakris G, Cariou B, Yue D, David-Neto E, Xi L, et al. Efficacy and safety of canagliflozin in subjects with type 2 diabetes and chronic kidney disease. Diabetes, obesity & metabolism. 2013;15(5):463-73.

49. Barnett AH, Mithal A, Manassie J, Jones R, Rattunde H, Woerle HJ, et al. Efficacy and safety of empagliflozin added to existing antidiabetes treatment in patients with type 2 diabetes and chronic kidney disease: a randomised, double-blind, placebo-controlled trial. Lancet Diabetes Endocrinol. 2014;2(5):369-84.

50. Kohan DE, Fioretto P, Tang W, List JF. Long-term study of patients with type 2 diabetes and moderate renal impairment shows that dapagliflozin reduces weight and blood pressure but does not improve glycemic control. Kidney Int. 2014;85(4):962-71.

51. Fioretto P, Del Prato S, Buse JB, Goldenberg R, Giorgino F, Reyner D, et al. Efficacy and safety of dapagliflozin in patients with type 2 diabetes and moderate renal impairment (chronic kidney disease stage 3A): The DERIVE Study. Diabetes, obesity & metabolism. 2018;20(11):2532-40.

52. Allegretti AS, Zhang W, Zhou W, Thurber TK, Rigby SP, Bowman-Stroud C, et al. Safety and Effectiveness of Bexagliflozin in Patients With Type 2 Diabetes Mellitus and Stage 3a/3b CKD. American journal of kidney diseases : the official journal of the National Kidney Foundation. 2019;74(3):328-37.

53. Macha S, Mattheus M, Halabi A, Pinnetti S, Woerle HJ, Broedl UC. Pharmacokinetics, pharmacodynamics and safety of empagliflozin, a sodium glucose cotransporter 2 (SGLT2) inhibitor, in subjects with renal impairment. Diabetes, obesity & metabolism. 2014;16(3):215-22.

54. Halden TAS, Kvitne KE, Midtvedt K, Rajakumar L, Robertsen I, Brox J, et al. Efficacy and Safety of Empagliflozin in Renal Transplant Recipients With Posttransplant Diabetes Mellitus. Diabetes Care. 2019;42(6):1067-74.

55. Persson F, Rossing P, Vart P, Chertow GM, Hou FF, Jongs N, et al. Efficacy and Safety of Dapagliflozin by Baseline Glycemic Status: A Prespecified Analysis From the DAPA-CKD Trial. Diabetes Care. 2021;44(8):1894-7.

56. ABCD/RA guideline: Clinical Practice Guidelines for Management of Hyperglycaemia in Adults with Diabetic Kidney Disease: 2021 Update. https://abcd.care/sites/abcd.care/files/site_uploads/Resources/Position-Papers/Management-of-hyperglycaemia-in-adults%20-with-DKD.pdf (accessed 7th December 2022).

57. Zoungas S, Arima H, Gerstein HC, Holman RR, Woodward M, Reaven P, et al. Effects of intensive glucose control on microvascular outcomes in patients with type 2 diabetes: a meta-analysis of individual participant data from randomised controlled trials. The Lancet Diabetes & Endocrinology. 2017;5(6):431-7.

58. Willis M, Nilsson A, Kellerborg K, Ball P, Roe R, Traina S, et al. Cost-Effectiveness of Canagliflozin Added to Standard of Care for Treating Diabetic Kidney Disease (DKD) in Patients with Type 2 Diabetes Mellitus (T2DM) in England: Estimates Using the CREDEM-DKD Model. Diabetes Ther. 2021;12(1):313-28.

59. McEwan P, Darlington O, Miller R, McMurray JJV, Wheeler DC, Heerspink HJL, et al. Cost-Effectiveness of Dapagliflozin as a Treatment for Chronic Kidney Disease. A Health-Economic Analysis of DAPA-CKD. 2022:CJN.03790322.

60. Kidney Disease: Improving Global Outcomes (KDIGO) 2012 Clinical Practice Guideline for the Evaluation and Management of Chronic Kidney Disease (CKD) https://kdigo.org/guidelines/ckd-evaluation-and-management/ (accessed 28th December 2020).

61. Jafar TH, Schmid CH, Landa M, Giatras I, Toto R, Remuzzi G, et al. Angiotensin-converting enzyme inhibitors and progression of nondiabetic renal disease. A meta-analysis of patient-level data. Ann Intern Med. 2001;135(2):73-87.

62. Lewis EJ, Hunsicker LG, Bain RP, Rohde RD. The effect of angiotensin-converting-enzyme inhibition on diabetic nephropathy. The Collaborative Study Group. N Engl J Med. 1993;329(20):1456-62.

63. Lewis EJ, Hunsicker LG, Clarke WR, Berl T, Pohl MA, Lewis JB, et al. Renoprotective effect of the angiotensin-receptor antagonist irbesartan in patients with nephropathy due to type 2 diabetes. N Engl J Med. 2001;345(12):851-60.

64. Cherney DZ, Perkins BA, Soleymanlou N, Xiao F, Zimpelmann J, Woerle HJ, et al. Sodium glucose cotransport-2 inhibition and intrarenal RAS activity in people with type 1 diabetes. Kidney Int. 2014;86(5):1057-8.

65. National Institute for Health and Care Excellence. Chronic kidney disease in adults: assessment and management. London: NICE; 2014. p. CG182. https://www.nice.org.uk/guidance/cg182 (accessed 02 June 2021).

66. KDIGO. KDIGO 2012 clinical practice guideline for the evaluation and management of chronic kidney disease. Kidney Int Suppl 2013; 3: 1–150.2013. p. 1-150.

67. Witte EC, Lambers Heerspink HJ, de Zeeuw D, Bakker SJ, de Jong PE, Gansevoort R. First morning voids are more reliable than spot urine samples to assess microalbuminuria. Journal of the American Society of Nephrology : JASN. 2009;20(2):436-43.

68. Ginsberg JM, Chang BS, Matarese RA, Garella S. Use of single voided urine samples to estimate quantitative proteinuria. N Engl J Med. 1983;309(25):1543-6.

69. UK Kidney Association. The UK eCKD Guide. https://renal.org/health-professionals/information-resources/uk-eckd-guide/proteinuria (accessed 02 June 2021).

70. Park JI, Baek H, Kim BR, Jung HH. Comparison of urine dipstick and albumin:creatinine ratio for chronic kidney disease screening: A population-based study. PLoS One. 2017;12(2):e0171106.

71. Wheeler DC, Stefansson BV, Jongs N, Chertow GM, Greene T, Hou FF, et al. Effects of dapagliflozin on major adverse kidney and cardiovascular events in patients with diabetic and non-diabetic chronic kidney disease: a prespecified analysis from the DAPA-CKD trial. Lancet Diabetes Endocrinol. 2021;9(1):22-31.

72. Cefalo CMA, Cinti F, Moffa S, Impronta F, Sorice GP, Mezza T, et al. Sotagliflozin, the first dual SGLT inhibitor: current outlook and perspectives. Cardiovasc Diabetol. 2019;18(1):20.

73. Grempler R, Thomas L, Eckhardt M, Himmelsbach F, Sauer A, Sharp DE, et al. Empagliflozin, a novel selective sodium glucose cotransporter-2 (SGLT-2) inhibitor: characterisation and comparison with other SGLT-2 inhibitors. Diabetes, obesity & metabolism. 2012;14(1):83-90.

Section 3: SGLT-2 inhibition and kidney protection in people with CKD without diabetes mellitus

3.1 Background

3.1.1 2023 Update to guidelines

Since publication of the previous guideline in 2021, there has been an increase in the available evidence for use of SGLT-2 inhibition in people without diabetes mellitus (DM). Of the two large placebo-controlled trials of SGLT-2 inhibition reported in 2022, EMPA-KIDNEY reported data from 3569 people with CKD without diabetes (1), while the DELIVER trial reported on 3453 people with heart failure without diabetes (2). An updated meta-analysis comprising all available data from the reported large SGLT-2 inhibitor randomised clinical trials (~90,000 participants) included ~10,000 participants with heart failure without DM and ~5000 participants with CKD without DM (3). Guideline updates in this section therefore reflect the increased strength of evidence in this previously understudied group.

3.1.2 Summary of trial evidence on kidney disease progression

The large-scale placebo-controlled trials of SGLT-2 inhibitors were not powered to assess effects on kidney disease progression in people without DM considered in isolation (see Table 1.1 in section 1). Information on the efficacy and safety of SGLT-2 inhibitors in people without DM is currently reliant on subgroup analyses of the large SGLT-2 inhibitor trials, with the evidence for CKD provided by DAPA-CKD and EMPA-KIDNEY (1, 4). Interpretation of subgroup analyses mandates additional considerations due to their more limited power compared to any primary assessment, the potential for multiplicity of testing to increase the likelihood of chance findings, and where relevant, their post-hoc nature. One advised approach to address some of these issues is statistical tests for effect modification. These assess whether or not the overall trial result for a given outcome is significantly different in a subgroup, and are often referred to as heterogeneity or interaction tests. In the absence of statistical evidence for heterogeneity, the most reliable quantitative estimate of the relative effect of the test intervention is the overall relative risk, with little weight given to relative risks calculated directly from a subgroup of participants considered in isolation. Despite such approaches, cautious interpretation is still required in underpowered situations. Further examples of considerations on subgroup analyses are provided in this introductory review (5).

Kidney disease progression results from DAPA-CKD

DAPA-CKD recruited people with albuminuric CKD with and without type 2 DM. Kidney-related inclusion criteria were an eGFR 25-75 mL/min/1.73m^2 plus a urinary albumin-to-creatinine ratio (uACR) of 200-5000 mg/g (23-566 mg/mmol) on a stable dose of an angiotensin-converting enzyme inhibitor (ACEi) or angiotensin-II receptor blockade (ARB) for ≥4 weeks. Key exclusion criteria were polycystic kidney disease, lupus nephritis, or anti- neutrophil cytoplasmic antibody (ANCA) associated vasculitis, and immunotherapy for kidney disease within 6 months before enrolment (6). DAPA-CKD randomised 4304 people to dapagliflozin 10mg versus matching placebo, including 1398 (32%) without DM. Overall, mean eGFR was 43

mL/min/1.73m^2 with ~1 gram per day of albuminuria (median uACR=949 mg/g [107 mg/mmol]) and 97% prescribed an ACEi or ARB. Those without DM had similar levels of eGFR and albuminuria to those with DM, but represented a much wider range of primary kidney diagnoses (Table 3.1) (4, 6).

Table 3.1: Kidney-related characteristics of DAPA-CKD and EMPA-KIDNEY participants by DM status (1, 3, 4, 6, 7)

		DAPA-CKD				EMPA-KIDNEY			
		DM		No DM		DM		No DM	
**Number of participants**		2906	(68%)	1398	(32%)	3039*	(46%)	3570	(54%)
**eGFR (mL/min/1.73 m^2)**	**Mean (SD)**	**43.8**	**(12.6)**	**41.7**	**(11.7)**	**36**	**(13.9)**	**38.7**	**(15.4)**
<30		401	(14%)	223	(16%)	1148	(38%)	1132	(32%)
≥30–<45		1239	(43%)	659	(47%)	1359	(45%)	1546	(43%)
≥45		1266	(44%)	516	(37%)	532	(18%)	892	(25%)
**uACR (mg/g)**	**Median (IQR)**	**1017**	**-**	**861**	**-**	**348**	**(68–1293)**	**461**	**(128–1117)**
<30		1	(0%)	0	(0%)	649	(21%)	683	(19%)
≥30–<300		307	(11%)	136	(9.7%)	941	(31%)	921	(26%)
≥300		2597	(89%)	1262	(90%)	1449	(48%)	1966	(55%)
**Primary kidney diagnosis**									
Diabetic nephropathy/diabetic kidney disease		2510	86%	0	0.0%	2057	(68%)	0	(0%)
Hypertensive/ renovascular disease		203	7.0%	494	35%	401	(13%)	1044	(29%)
Any glomerular disease		97	3.3%	598	43%	172	(6%)	1497	(42%)
- IgA nephropathy		38	1.3%	232	16.6%	59	(2%)	758	(21%)
- Focal segmental glomerulosclerosis		22	0.8%	93	6.7%	34	(1%)	161	(5%)
- Membranous nephropathy		10	0.3%	33	2.4%	13	(0%)	83	(2%)
- Minimal change disease		2	0.1%	9	0.6%	4	(0%)	10	(0%)
- Other glomerular disease		25	0.9%	231	16.5%	62	(2%)	485	(14%)
Other		49	1.7%	139	9.9%	203	(7%)	605	(17%)
Unknown		47	1.6%	167	11.9%	206	(7%)	424	(12%)

DAPA-CKD was stopped early due to efficacy, with allocation to dapagliflozin reducing its primary cardiorenal composite outcome (a sustained 50% decline in eGFR, ESKD, or death from renal or cardiovascular causes) by 39% compared to placebo (197/2152 vs 312/2152: hazard ratio [HR]=0.61, 95% confidence interval [CI] 0.51-0.72). This included a 44% reduction in the risk of the kidney disease progression component of this composite (142 vs 243: HR=0.56, 95%CI 0.45-0.68) and a 34% reduction in the risk of initiation of maintenance dialysis, receipt of a kidney transplant or a renal death (71 vs 103: HR=0.66, 95%CI 0.49- 0.90).

Importantly, the relative risk reductions for the primary outcome were similar when analyses were performed separately in people with and without DM, and between eGFR and uACR subgroupings, with nominally significant relative risk reductions in each of these subgroups (all heterogeneity test p>0.05) (4).

Kidney disease progression results from EMPA-KIDNEY

EMPA-KIDNEY enrolled a broad range of participants with CKD at risk of progression (7). Participants were required to have either an eGFR of 20-45 mL/min/1.73m^2 irrespective of uACR, or an eGFR ≥45 mL/min/1.73m^2 and uACR ≥200mg/g (≥23 mg/mmol) or protein-to-creatinine ratio ≥300 mg/g (≥34 mg/mmol), and be treated with a clinically appropriate dose of a renin-angiotensin system (RAS) inhibitor if indicated and tolerated. Compared to DAPA-CKD, EMPA-KIDNEY included a broad range of primary kidney diagnoses, excluding only those with polycystic kidney disease or kidney transplant. Participants were also not eligible if they required intravenous immunosuppression or daily dose of prednisolone (or equivalent) greater than 45mg. Although type 1 diabetes was not an exclusion criterion at the start of enrolment, a protocol amendment prevented further inclusion of people with type 1 diabetes at the request of the funder, after 68 people with type 1 diabetes had been enrolled (7).

Compared to DAPA-CKD, participants enrolled to EMPA-KIDNEY had a lower average uACR (median 412 mg/g in EMPA-KIDNEY vs 949 mg/g in DAPA-CKD), and a lower baseline eGFR (mean 37.3 mL/min/1.73m^2 vs 43.1 mL/min/1.73m^2). Within EMPA-KIDNEY, participants without diabetes had a similar eGFR to those with diabetes (mean eGFR 36.0 vs 38.7), but participants without diabetes had a greater average uACR (median 461 in people without diabetes vs 348 in people with diabetes; Table 3.1). Similar to DAPA-CKD, people without diabetes represented a broad range of primary kidney diagnoses, with 42% and 29% of participants diagnosed with glomerular disease and hypertensive or ischaemic nephropathy, respectively (Table 3.1).

As with DAPA-CKD, EMPA-KIDNEY was stopped early due to evidence of efficacy in the primary outcome. Empagliflozin significantly reduced the rate of the primary composite outcome, defined as a sustained decline of 40% in eGFR, ESKD, or death from cardiovascular or kidney causes, by 28% (432/3304 vs 558/3305, HR=0.72, 95%CI 0.64-0.82) (1). This was primarily driven by a 29% reduction in the rate of kidney disease progression (384/3304 vs 504/3305, HR=0.71, 95%CI 0.62-0.81), while death from cardiovascular causes occurred in 1.8% and 2.1% of placebo- or empagliflozin-treated participants, respectively (HR=0.84, 95%CI 0.60-1.19). Empagliflozin reduced the rate of the primary composite outcome in people with and without diabetes and across the spectrum of eGFR, without evidence of interaction (P for interaction >0.05 in both cases). There was a significant interaction by uACR status for the primary outcome (P=0.02), with greater benefit observed among participants with higher levels of albuminuria. As discussed in Section 2, the small number of outcomes in the normoalbuminuria subgroup, and low rate of decline in kidney function may have diminished the power of EMPA-KIDNEY to detect meaningful differences in the primary outcome in this subgroup.

eGFR slope analyses were used to explore effects by level of albuminuria in more detail. There was a reduction in the rate of decline of kidney function, both from the start of treatment to final follow-up visit (the “total slope”) and from 2 months of treatment to final follow-up visit (the “chronic slope”). The chronic slope excludes the acute reversible eGFR dip which may be a manifestation of the reduction in intraglomerular pressure caused by SGLT-2 inhibition (8), and therefore may be more representative of the true effect of SGLT-2 inhibition on preservation of glomerular kidney function. Overall, empagliflozin reduced the absolute rate of decline in eGFR from month 2 to final follow-up visit by 1.37 mL/min/1.73m^2 per year

(95%CI 1.16-1.59), representing ~50% reduction in rate of decline (in relative terms) (1). Importantly, reductions in the chronic eGFR slopes were observed for empagliflozin across all key pre-specified subgroups, including by the presence or absence of diabetes, by eGFR, or by baseline uACR, and the difference in rate of decline exceeded the threshold at which this would be expected to translate into clinical benefit (9).

Meta-analysis of trials in people without diabetes

The recent collaborative meta-analysis conducted by the Nuffield Department of Population Health Renal Studies Group (RSG) and SGLT-2 inhibitor Meta-Analysis Cardio-Renal Trialists' Consortium (SMART-C) aimed to investigate the question of efficacy of SGLT-2 inhibition in people with and without diabetes, including in a population with CKD (3). Combining data from all 13 large SGLT-2 inhibitor trials, including 6 trials enrolling almost 16,000 participants without diabetes, the analysis identified a 37% (95%CI 31% to 42%) reduction in the risk of progression of kidney disease (using a standardised definition comprising a ≥50% decline in eGFR, kidney failure, or death from kidney failure), which was of a similar magnitude in participants with and without diabetes (among people without diabetes: HR=0.69, 95%CI 0.57-0.82, 489 events; P value for interaction by diabetes status p=0.31). As discussed in Section 2 and Section 3.1.2, the best estimate of effect in people without diabetes is therefore the overall treatment estimate from the meta-analysis (see figure 2.2). Meta-analysis of the kidney disease progression outcome subdivided by primary kidney diagnosis (including previously unpublished data from EMPA-KIDNEY) included 133 outcomes in 2132 people with ischaemic or hypertensive nephropathy and 231 outcomes in 2364 people with glomerular disease, demonstrating that there was no significant subgroup interaction by primary kidney diagnosis (Figure 3.1). Furthermore, meta-analysis of DAPA-CKD and EMPA-KIDNEY by subtype of glomerular disease demonstrated no evidence of interaction by glomerular disease type, though numbers of events were small for focal segmental glomerulosclerosis (13/151 vs 15/159 in empagliflozin and placebo arms, respectively). This analysis included 205 kidney disease progression outcomes in 1087 people with IgA nephropathy, representing one of the largest studies of an intervention in this population to date (Figure 3.2). The meta-analysis also suggested kidney benefits in people without diabetes who were included in the heart failure trials (3).

Figure 3.1: Effect of SGLT-2 inhibition on kidney disease progression, by primary kidney diagnosis (adapted from (3))

	Mean baseline eGFR, mL/min per 1·73m²	Events/participants		Event rate per 1000 patient-years		RR (95% CI)
		SGLT2 inhibitor	Placebo	SGLT2inhibitor	Placebo	
**Diabetic kidney disease or nephropathy***						
CREDENCE	56	153/2202	230/2199	27	41	0·64 (0·52–0·79)
SCORED	44	37/5292	52/5292	5	7	0·71 (0·46–1·08)
DAPA-CKD	43	93/1271	157/1239	36	64	0·55 (0·43–0·71)
EMPA-KIDNEY	36	85/1032	133/1025	42	67	0·56 (0·43–0·74)
**Subtotal**	**46**	**368/9797**	**572/9755**	..	..	**0·60 (0·53–0·69)**
**Ischaemic and hypertensive kidney disease**						
DAPA-CKD	43	18/324	26/363	28	37	0·74 (0·40–1·36)
EMPA-KIDNEY	35	37/706	52/739	27	37	0·69 (0·45–1·05)
**Subtotal**	**38**	**55/1030**	**78/1102**	..	..	**0·70 (0·50–1·00)**
**Glomerular disease**						
DAPA-CKD	43	21/343	46/352	33	70	0·43 (0·26–0·72)
EMPA-KIDNEY	42	69/853	95/816	44	64	0·68 (0·50–0·93)
**Subtotal**	**42**	**90/1196**	**141/1168**	..	..	**0·60 (0·46–0·78)**
**Other kidney disease or unknown**						
DAPA-CKD	43	10/214	14/198	25	37	0·81 (0·35–1·83)
EMPA-KIDNEY	36	36/713	52/725	26	36	0·72 (0·47–1·10)
**Subtotal**	**38**	**46/927**	**66/923**	..	..	**0·74 (0·51–1·08)**
**Any diagnosis**						
CREDENCE	56	153/2202	230/2199	27	41	0·64 (0·52–0·79)
SCORED	44	37/5292	52/5292	5	7	0·71 (0·46–1·08)
DAPA-CKD	43	142/2152	243/2152	33	58	0·56 (0·45–0·68)
EMPA-KIDNEY	37	227/3304	332/3305	36	52	0·64 (0·54–0·76)
**Total**	**44**	**559/12950**	**857/12948**	..	..	**0·62 (0·56–0·69)**

Heterogeneity across groups of primary kidney disease: p=0·67
Trend across trials sorted by eGFR for any diagnosis: p=0·88

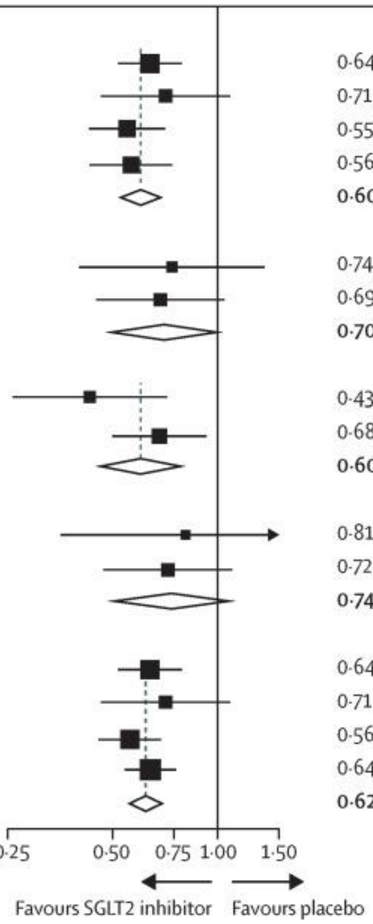

CI=confidence interval. eGFR=estimated glomerular filtration rate. RR=relative risk. SGLT2i=sodium glucose co-transporter-2 inhibitor.

Figure 3.2: Effect of SGLT-2 inhibition on kidney disease progression, by type of glomerular disease (adapted from (3))

	Average baseline eGFR (mL/min/1.73m²)	Events/participants SGLT2i	Events/participants Placebo	Rate per 1000 patient years SGLT2i	Rate per 1000 patient years Placebo	Relative risk (95% CI)
**IgA nephropathy**						
DAPA-CKD	44	5/137	20/133	21	88	0.24 (0.09, 0.65)
EMPA-KIDNEY	43	32/413	48/404	43	65	0.56 (0.36, 0.89)
**Subtotal**	**43**	**37/550**	**68/537**			**0.49 (0.32, 0.74)**
**Focal segmental glomerulosclerosis**						
DAPA-CKD	42	4/53	7/62	37	57	0.52 (0.15, 1.83)
EMPA-KIDNEY	41	9/98	8/97	46	43	1.24 (0.47, 3.25)
**Subtotal**	**41**	**13/151**	**15/159**			**0.89 (0.42, 1.92)**
**Other glomeronephritis**						
DAPA-CKD	43	12/153	19/157	33	50	0.65 (0.33, 1.29)
EMPA-KIDNEY	42	28/342	39/315	44	68	0.70 (0.43, 1.15)
**Subtotal**	**42**	**40/495**	**58/472**			**0.68 (0.46, 1.02)**
**ANY GLOMERULAR DISEASE**						
DAPA-CKD	43	21/343	46/352	33	70	0.43 (0.26, 0.72)
EMPA-KIDNEY	42	69/853	95/816	44	64	0.68 (0.50, 0.93)
**TOTAL**	**42**	**90/1196**	**141/1168**			**0.60 (0.46, 0.78)**

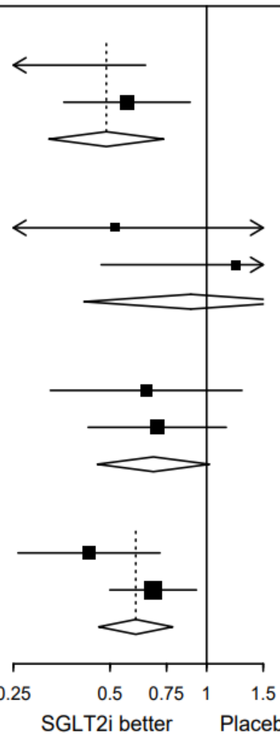

Heterogeneity across three subtypes of glomerular disease: p=0.30

CI=confidence interval. eGFR=estimated glomerular filtration rate. RR=relative risk. SGLT2i=sodium glucose co-transporter-2 inhibitor.

Acute kidney injury in people without DM

SGLT-2 inhibitors have been shown not to increase the risk of acute kidney injury (AKI), as discussed in Section 2. The RSG and SMART-C collaborative meta-analysis assessed the effect of SGLT-2 inhibition on AKI stratified by diabetes status (3). SGLT-2 inhibition was found to reduce the risk of AKI overall by 23% (HR=0.77, 95%CI 0.70-0.84), and there was no evidence that the benefit in terms of AKI was different in people with or without diabetes (people without diabetes: HR=0.66, 95%CI 0.54-0.81; heterogeneity by diabetes status P=0.12), and was similar in the CKD and HF trial populations.

3.1.3 Summary of trial evidence on cardiovascular risk

Few participants without diabetes died from cardiovascular disease or were hospitalised for heart failure during follow up in the CKD trials. Among the 1398 DAPA-CKD participants without DM, 34 experienced a cardiovascular death or hospitalisation for heart failure event, while in EMPA-KIDNEY 69 participants experienced such an event. Consequently, the majority of evidence for the effect of SGLT-2 inhibitors on cardiovascular risk in people without diabetes comes from the heart failure trials, where the rate of cardiovascular death or hospitalisation for heart failure was approximately 5-10-fold that in DAPA-CKD or

EMPA-KIDNEY (3).

Figure 3.3: Effect of SGLT-2 inhibition on cardiovascular death or hospitalisation for heart failure, by diabetes status (adapted from (3))

	Average baseline eGFR (mL/min/1.73m²)	Events/participants SGLT2i	Events/participants Placebo	Rate per 1000 patient years SGLT2i	Rate per 1000 patient years Placebo	Relative risk (95% CI)	Trend across trials sorted by eGFR
**Diabetes**							
DECLARE-TIMI 58	85	417/8582	496/8578	12	15	0.83 (0.73, 0.95)	
CANVAS Program	77	364/5795	288/4347	16	21	0.78 (0.67, 0.91)	
VERTIS CV	76	444/5499	250/2747	23	27	0.88 (0.75, 1.03)	
EMPA-REG OUTCOME	74	265/4687	198/2333	20	30	0.66 (0.55, 0.79)	
DAPA-HF	63	213/1075	268/1064	144	191	0.75 (0.63, 0.90)	
EMPEROR-REDUCED	61	200/927	265/929	177	246	0.72 (0.60, 0.87)	
EMPEROR-PRESERVED	60	239/1466	291/1472	83	102	0.79 (0.67, 0.94)	p=0.22
DELIVER	60	271/1578	330/1572	83	104	0.80 (0.68, 0.93)	
CREDENCE	56	179/2202	253/2199	32	45	0.69 (0.57, 0.83)	
SOLOIST-WHF	51	NA/608	NA/614	-	-	0.71 (0.56, 0.89)	
SCORED	44	283/5292	357/5292	41	52	0.77 (0.66, 0.91)	
DAPA-CKD	44	85/1455	119/1451	27	38	0.70 (0.53, 0.92)	
EMPA-KIDNEY	36	96/1525	118/1515	32	40	0.78 (0.60, 1.03)	
**Subtotal: DIABETES**	**67**	**3056/40691**	**3233/34113**			**0.77 (0.73, 0.81)**	
**No diabetes**							
DAPA-HF	68	169/1298	227/1307	91	124	0.73 (0.60, 0.89)	
EMPEROR-REDUCED	63	161/936	197/938	139	176	0.78 (0.64, 0.97)	
DELIVER	63	204/1551	246/1558	62	76	0.82 (0.68, 0.99)	p=0.28
EMPEROR-PRESERVED	62	176/1531	220/1519	56	72	0.78 (0.64, 0.95)	
DAPA-CKD	42	15/697	19/701	11	13	0.79 (0.40, 1.55)	
EMPA-KIDNEY	39	35/1779	34/1790	10	9.9	1.04 (0.65, 1.67)	
**Subtotal: NO DIABETES**	**56**	**760/7792**	**943/7813**			**0.79 (0.72, 0.87)**	
**TOTAL: OVERALL**	**65**	**3816/48483**	**4176/41926**			**0.77 (0.74, 0.81)**	

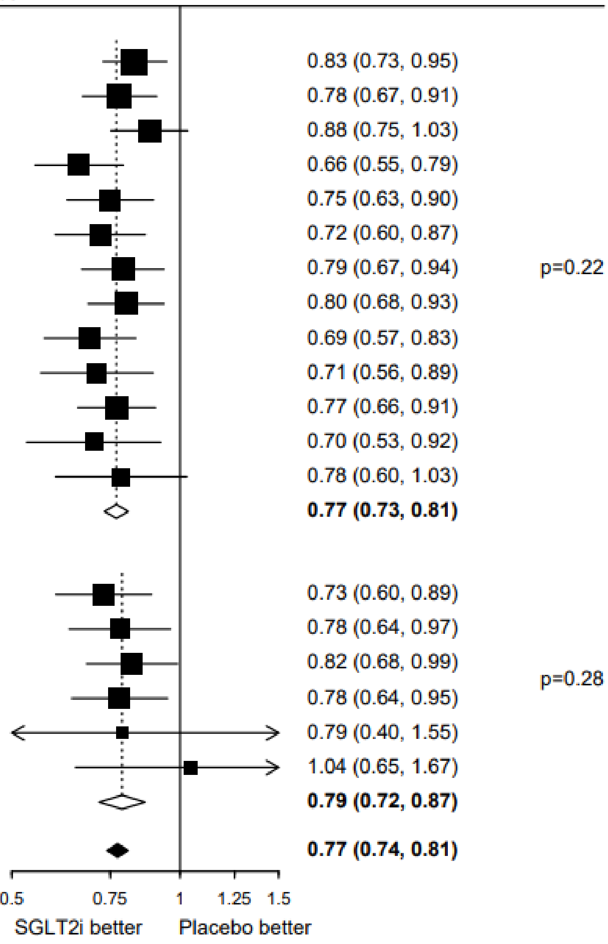

Heterogeneity by diabetes status: p=0.67

*Excludes urgent heart failure visits. EMPA-REG OUTCOME cardiovascular death definition excluded stroke. CI=confidence interval; eGFR=estimated glomerular filtration rate.

From the heart failure trials, DAPA-HF included people with an eGFR down to 30 mL/min/1.73m^2, and 1926 (41%) of the trial population had an eGFR <60 mL/min/1.73m^2. EMPEROR-REDUCED and EMPEROR-PRESERVED included people with an eGFR down to 20 mL/min/1.73m^2, about one-half of both trials representing people with an eGFR <60 mL/min/1.73m^2 (10-13). In DELIVER, which included participants to an eGFR of 25 mL/min/1.73m^2, around 50% of participants had eGFR <60 mL/min/1.73m^2, and 23% had an eGFR <45 mL/min/1.73m^2 (2, 14). All four trials reported that SGLT-2 inhibition versus placebo reduced the risk of their primary composite outcomes based on cardiovascular death or hospitalisation for heart failure, with relative risk reductions appearing similar in size and nominally significant in people with and without DM (Figure 3.3) (2, 3, 10-15). Cardiovascular benefits were also unmodified among those with evidence of CKD (2, 3, 10-15).

3.1.4 Benefits and risks of SGLT-2 inhibition in people without type 2 DM

As discussed in Section 2, serious risks of SGLT-2 inhibition, including ketoacidosis and lower limb amputation, are rare, and the absolute risk of harm due to these complications is greatly exceeded by the absolute benefits derived from reduction in kidney disease progression, AKI, and cardiovascular death or hospitalisation for heart failure. In people without diabetes, such complications are exceedingly rare, and the ratio of benefit to risk is likely to be even greater than among those with type 2 DM (Section 2, Figure 2.8). Section 5 discusses the risks of SGLT-2 inhibition in more detail.

3.1.5 Quality of the evidence

See section 2.1.5 for details of quality of evidence of the large placebo-controlled trials in SGLT-2 inhibitors. Briefly, both DAPA-CKD and EMPA-KIDNEY were found to be low risk of bias as assessed using the Cochrane Risk of Bias 2 tool, due to high quality of randomization, blinding, outcome assessment and reporting. It should be noted that both trials were stopped early due to evidence of efficacy, which may lead to overestimation of the effect on the primary outcome overall or in subgroups (and may explain the observed reduction in non-cardiovascular mortality in DAPA-CKD, which is a heterogeneous result compared to other SGLT-2 inhibitor trials (16)).

3.1.6 Summary of published cost-effective analyses

The DAPA-CKD investigators undertook a cost-effectiveness analysis of dapagliflozin in people with CKD using primary data from the DAPA-CKD trial, with cost estimates presented for a UK-specific population (discussed in detail in Section 2) (17). This analysis, which demonstrated lifetime cost-effectiveness of dapagliflozin in CKD with an incremental cost-effectiveness ratio (ICER) of £6,020 per quality-adjusted life year (QALY) additionally conducted several subgroup analyses including cost effectiveness stratified by diabetes status. In this subgroup analysis, dapagliflozin was marginally more cost effective among people without diabetes than people with diabetes at a cost of $7,897/QALY for people without diabetes vs $8,444/QALY for people with diabetes (data in pound sterling not presented).

At the time of writing, we were unable to identify peer-reviewed publications of cost-effectiveness of SGLT-2 inhibition that were specifically undertaken in people with CKD without type 2 DM.

3.2 Recommendations for use

In people without type 2 DM:

1. **We recommend initiating SGLT-2 inhibition in people with chronic kidney disease, irrespective of primary kidney disease,* for any of the following clinical scenarios (Grade 1A):**

    a. **eGFR of ≥20 mL/min/1.73m^2 and a urinary albumin-to-creatinine ratio (uACR) of ≥25 mg/mmol†**
    b. **Symptomatic heart failure, irrespective of ejection fraction**

*** excludes people with polycystic kidney disease, type 1 diabetes, or a kidney transplant**
† urinary protein-to-creatinine ratio of 35 mg/mmol can be considered equivalent

Rationale: SGLT-2 inhibition has been shown to be effective in people with albuminuric chronic kidney disease across broad population, including people with and without type 2 diabetes, and down to an eGFR of 20 mL/min/1.73m^2. Meta-analysis of CKD trials and all SGLT-2 inhibitor trials demonstrates that the kidney benefits of SGLT-2 inhibition are not modified by the presence or absence of diabetes. Furthermore, DAPA-CKD and EMPA-KIDNEY both indicate consistent benefits irrespective of primary kidney disease, with benefits observed in glomerular disease of a similar magnitude to those seen in diabetic kidney disease.

SGLT-2 inhibition has been demonstrated to reduce the risk of heart failure hospitalisation in people with stable established symptomatic HFrEF by the DAPA-HF and EMPEROR-REDUCED trials, with relative effects similar in people with and without DM. Data from EMPEROR-PRESERVED and DELIVER confirm benefits on heart failure complications in people with HFpEF, including people without DM. The four large trials recruited a substantial proportion of people with CKD, with cardiac benefits appearing to be unmodified by moderately reduced levels of eGFR. We therefore provide a grade 1A recommendation for use of SGLT-2 inhibition in people without DM with an eGFR ≥20 mL/min/1.73m^2 and uACR ≥25 mg/mmol, or those with symptomatic heart failure. Note that the 25 mg/mmol threshold for uACR was chosen pragmatically, given that this value is expected to be easier to recall for practicing clinicians than a cut-off of 23 mg/mmol, as is commonly used in the CKD trials.

2. **We recommend initiating SGLT-2 inhibition to slow rate of kidney function decline in people with an eGFR of 20-45 mL/min/1.73m^2 and a uACR of <25 mg/mmol* (Grade 1B)**

*** urinary protein-to-creatinine ratio of 35 mg/mmol can be considered equivalent**

Rationale: The excellent safety profile of SGLT-2 inhibition in people with CKD without DM has been established in nearly 5000 such people from DAPA-CKD and EMPA-KIDNEY (see Section 5 for further details). DAPA-CKD and EMPA-KIDNEY demonstrated beneficial effects across the spectrum of eGFR in terms of progression of kidney disease in people with and without diabetes. Furthermore, EMPA-KIDNEY also showed that among people without albuminuria rate of kidney function decline (chronic eGFR slope) was reduced by 0.78 mL/min/1.73m^2 per year in participants with A1 levels of albuminuria (to a rate of -0.11 mL/min/1.73m^2 per year), and by 1.20 mL/min/1.73m^2 per year in participants with A2 levels. Such absolute benefits would be predicted to translate into clinically meaningful reductions in progression of kidney disease, even among

people with low albuminuria at baseline (9). Among those with low levels of albuminuria and low eGFR at initiation, such effects on eGFR decline could translate into delay in the onset of kidney failure if used for a few/several years. Furthermore, SGLT-2 inhibition has been shown to reduce the risk of AKI and cardiovascular risk. Given the clear magnitude of benefit of SGLT-2 inhibition in this population and the reassuring safety profile, we provide a grade 1 recommendation for this statement. However, at present only EMPA-KIDNEY provides direct evidence in this population, with further supporting evidence obtained from eGFR slopes of the trials in heart failure (i.e. grade B level of evidence).

3. We suggest clinicians consider initiating SGLT-2 inhibition in people with an eGFR below 20 mL/min/1.73m^2 to slow progression of kidney disease (Grade 2B)

Rationale: Two of the four clinical trials of SGLT-2 inhibition conducted in people with CKD have enrolled people without diabetes (DAPA-CKD and EMPA-KIDNEY), and in both trials SGLT-2 inhibition has been continued below an eGFR of 20 mL/min/1.73m^2 without evidence of increased adverse events. In EMPA-KIDNEY, in which over half of the participants did not have diabetes, SGLT-2 inhibition was shown to have consistent relative benefits across the spectrum of eGFR, including among people with eGFR <20 mL/min/1,73m^2 (see section 2 for details; Figure 2.5). Given that people with low eGFR are at high risk of progression to kidney failure, the absolute benefit is likely to be considerable for this population, irrespective of diabetes status. We therefore provide a grade 2B recommendation for initiation of use of SGLT-2 inhibition for people without type 2 DM with eGFR <20 mL/min/1.73m^2.

Section 3: Recommendations for Use Footnote

All recommendations in sections 2 & 3 exclude people with type 1 DM (see section 7a) and exclude those with a kidney transplant (see section 7b).

**** See section 4 for summary of indications/licenced uses***

3.3 Clinical research recommendations

See section 2.3 which includes Recommendations for Research irrespective of DM status.

3.4 Recommendations for implementation

See section 2.4 which includes Recommendations for Implementation irrespective of DM status.

3.5 Audit measures

See section 2.5 which provides audit measures irrespective of DM status.

3.6 References

1. EMPA-KIDNEY Collaborative Group. Empagliflozin in Patients with Chronic Kidney Disease. New England Journal of Medicine. 2022.
2. Solomon SD, McMurray JJV, Claggett B, de Boer RA, DeMets D, Hernandez AF, et al. Dapagliflozin in Heart Failure with Mildly Reduced or Preserved Ejection Fraction. New England Journal of Medicine. 2022;387(12):1089-98.
3. Nuffield Department of Population Health Renal Studies Group and the SGLT2 inhibitor Meta-Analysis Cardio-Renal Trialists' Consortium. Impact of diabetes on the effects of sodium glucose co-transporter-2 inhibitors on kidney outcomes: collaborative meta-analysis of large placebo-controlled trials. The Lancet. 2022;400(10365):1788-801.
4. Heerspink HJL, Stefansson BV, Correa-Rotter R, Chertow GM, Greene T, Hou FF, et al. Dapagliflozin in Patients with Chronic Kidney Disease. N Engl J Med. 2020;383(15):1436-46.
5. Peto R. Current misconception 3: that subgroup-specific trial mortality results often provide a good basis for individualising patient care. Br J Cancer. 2011;104(7):1057-8.
6. Wheeler DC, Stefansson BV, Batiushin M, Bilchenko O, Cherney DZI, Chertow GM, et al. The dapagliflozin and prevention of adverse outcomes in chronic kidney disease (DAPA-CKD) trial: baseline characteristics. Nephrol Dial Transplant. 2020;35(10):1700-11.
7. EMPA-KIDNEY Collaborative Group. Design, recruitment, and baseline characteristics of the EMPA-KIDNEY trial. Nephrology Dialysis Transplantation. 2022;37(7):1317-29.
8. Jongs N, Chertow GM, Greene T, McMurray JJV, Langkilde AM, Correa-Rotter R, et al. Correlates and Consequences of an Acute Change in eGFR in Response to the SGLT2 Inhibitor Dapagliflozin in Patients with CKD. Journal of the American Society of Nephrology. 2022;33(11):2094-107.
9. Levey AS, Gansevoort RT, Coresh J, Inker LA, Heerspink HL, Grams ME, et al. Change in Albuminuria and GFR as End Points for Clinical Trials in Early Stages of CKD: A Scientific Workshop Sponsored by the National Kidney Foundation in Collaboration With the US Food and Drug Administration and European Medicines Agency. Am J Kidney Dis. 2020;75(1):84-104.
10. McMurray JJV, Solomon SD, Inzucchi SE, Kober L, Kosiborod MN, Martinez FA, et al. Dapagliflozin in Patients with Heart Failure and Reduced Ejection Fraction. N Engl J Med. 2019;381(21):1995-2008.
11. Anker SD, Butler J, Filippatos G, Ferreira JP, Bocchi E, Böhm M, et al. Empagliflozin in Heart Failure with a Preserved Ejection Fraction. New England Journal of Medicine. 2021;385(16):1451-61.
12. Packer M, Anker SD, Butler J, Filippatos G, Pocock SJ, Carson P, et al. Cardiovascular and Renal Outcomes with Empagliflozin in Heart Failure. N Engl J Med. 2020;383(15):1413-24.
13. Zannad F, Ferreira JP, Pocock SJ, Anker SD, Butler J, Filippatos G, et al. SGLT2 inhibitors in patients with heart failure with reduced ejection fraction: a meta-analysis of the EMPEROR-Reduced and DAPA-HF trials. Lancet. 2020;396(10254):819-29.
14. Mc Causland FR, Claggett BL, Vaduganathan M, Desai AS, Jhund P, de Boer RA, et al. Dapagliflozin and Kidney Outcomes in Patients With Heart Failure With Mildly Reduced or Preserved Ejection Fraction: A Prespecified Analysis of the DELIVER Randomized Clinical Trial. JAMA Cardiology. 2022.
15. Zannad F, Ferreira JP, Pocock SJ, Zeller C, Anker SD, Butler J, et al. Cardiac and Kidney Benefits of Empagliflozin in Heart Failure Across the Spectrum of Kidney Function: Insights From EMPEROR-Reduced. Circulation. 2021;143(4):310-21.
16. Staplin N, Roddick AJ, Emberson J, Reith C, Riding A, Wonnacott A, et al. Net effects of sodium-glucose co-transporter-2 inhibition in different patient groups: a meta-analysis of large placebo-controlled randomized trials. eClinicalMedicine. 2021;41.

17. McEwan P, Darlington O, Miller R, McMurray JJV, Wheeler DC, Heerspink HJL, et al. Cost-Effectiveness of Dapagliflozin as a Treatment for Chronic Kidney Disease. A Health-Economic Analysis of DAPA-CKD. 2022;17(12):1730-41.

Section 4: Selection of SGLT-2 inhibitors

(A summary of current UK licences)

4.1 Background

There are currently four sodium-glucose co-transporter-2 (SGLT-2) inhibitors that have a licence for use within the UK: canagliflozin, dapagliflozin, empagliflozin and ertugliflozin.

Licences for medications in the UK are issued by the Medicines and Healthcare products Regulatory Agency (MHRA). A product licence will set out the criteria for which a medication has been approved for use and is granted based on review of clinical trial efficacy and safety data. The pharmaceutical company responsible for the manufacture of the medication will produce a document called the Summary of Product Characteristics (SmPC) outlining the properties, conditions for use and licensing information of the product.

The term ‘off-label’ describes the use of a medication outside of the criteria defined within the licence. The term ‘unlicensed’ refers to the use of a medication that has not had a licence granted for use by the MHRA in the UK.

SGLT-2 inhibitors are prescription-only medicines (POM). The UK licences for the SGLT-2 inhibitors were initially focused on glycaemic effectiveness, however they are continually updated in response to any new data published on the individual medications. For example, the empagliflozin (Jardiance) licence was updated in 2017 after the cardiovascular benefit data from the EMPA-REG OUTCOME trial were published (1).
These data resulted in a subsequent amendment to the wording of the therapeutic indication, removing ‘glycaemic control’ as the only treatment goal of empagliflozin therapy. In June 2020, canagliflozin also underwent a licence update expanding its indications based on trial data from CREDENCE (2).

In late 2020, dapagliflozin had a new therapeutic indication added to the licence as a consequence of the DAPA-HF trial to include the treatment of symptomatic chronic heart failure with reduced ejection fraction (HFrEF), closely followed by empagliflozin in July 2021 also adding HFrEF as a licensed indication after review of the EMPEROR-REDUCED data (3, 4).

In August 2021, an additional update to the dapagliflozin licence based on review of the results from the DAPA-CKD study gave dapagliflozin an indication to be used for the treatment of chronic kidney disease (CKD) (5).

The most recent updates to the SGLT-2 inhibitor licensed indications have expanded the heart failure licences of empagliflozin and dapagliflozin to preserved ejection fraction after the addition of EMPEROR-PRESERVED and DELIVER studies to the evidence base (6, 7).

Upon publication of the EMPA-KIDNEY Trial in November 2022 (8), a licence update for empagliflozin to include the treatment of CKD is expected.

An additional medication to consider is sotagliflozin; a combination SGLT-1/SGLT-2 inhibitor. Sotagliflozin, is not licensed or currently available in the UK. It was initially approved for use in the EU by the European Medicines Agency, for people with type 1 diabetes mellitus (DM) with a BMI of ≥27 kg/m^2, where optimal insulin therapy has failed to adequately maintain glycaemic control - this approval has now been

withdrawn (9). The National Institute for Health and Care Excellence (NICE) pre-emptively published a technology appraisal (NICE TA622) recommending the use of sotagliflozin with the same indication as the EMA criteria above, pending any UK licensing (10). A summary for the use of the different SGLT-2 inhibitors in relation to CKD stage can be found in Table 4.1, below:

Table 4.1 Dosing of SGLT-2 Inhibitors based on current UK regulatory licences (all doses are once daily)

**SGLT-2 inhibitor**	**Indication**	**eGFR, mL/min/1,73m²**					
		**>90**	**60 - <90**	**45 - <60**	**30 - <45**	**15 - <30**	**<15**
**Canagliflozin**	**Type 2 DM**	✓ 100-300 mg	✓ 100-300 mg	✓ 100 mg	✓ 100 mg !	– 100 mg ! *Continue if initiated for albuminuria*	– 100 mg ! *Continue if initiated for albuminuria*
**Dapagliflozin**	**Type 2 DM**	✓ 10 mg	✓ 10 mg	✓ 10 mg	✓ 10 mg !	✓ 10 mg !	–
	**CKD**	✓ 10 mg	✓ 10 mg	✓ 10 mg	✓ 10 mg	✓ 10 mg *Limited experience at eGFR <25*	–
	**HF**	✓ 10 mg	✓ 10 mg	✓ 10 mg	✓ 10 mg	✓ 10 mg *Limited experience at eGFR <25*	–
**Empagliflozin**	**Type 2 DM**	✓ 10-25 mg	✓ 10-25 mg	✓ 10 mg *Initiate if type 2 DM AND established cardiovascular disease*	✓ 10 mg ! *Initiate if type 2 DM AND established cardiovascular disease*	✗	✗
	**HF**	✓ 10 mg	✓ 10 mg	✓ 10 mg	✓ 10 mg	✓ (if eGFR ≥20) 10 mg ✗ (if eGFR <20)	✗
**Ertugliflozin**	**Type 2 DM**	✓ 5-15 mg	✓ 5-15 mg	✓ 5-15 mg	– 5-15 mg !	✗	✗

CKD=chronic kidney disease; DM=diabetes mellitus; eGFR=estimated glomerular filtration rate (mL/min/1.73m^2); HF=heart failure.

! In people with diabetes mellitus, the glucose lowering efficacy of SGLT-2 inhibition is reduced when eGFR is < 45 mL/min/1.73m^2, and is likely absent in people with severe kidney impairment. Therefore, if eGFR falls below 45 mL/min/1.73m^2, additional glucose lowering treatment should be considered in people with diabetes mellitus

✓ Initiate
– Continue, not for initiation
✗ Discontinue

4.2 Current licensed indications for SGLT-2 inhibitor use

Based on major clinical trial evidence of SGLT-2 inhibitors for cardiorenal protection, the smallest labelled dose of SGLT-2 inhibitors would be sufficient to achieve this target.

The current indications of the UK licensed SGLT-2 inhibitors are stated below:

Canagliflozin (Invokana) (11)

1. Canagliflozin is indicated for the treatment of adults with insufficiently controlled type 2 DM as an adjunct to diet and exercise:
   - As monotherapy when metformin is considered inappropriate due to intolerance or contraindications
   - In addition to other medicinal products for the treatment of diabetes

   For this indication, canagliflozin should be initiated at a dose of 100 mg. If further glycaemic control is required, the dose can be increased to 300 mg a day if estimated glomerular filtration rate (eGFR) of above 60 mL/min/1.73m^2.Once the eGFR has moved below 60 mL/min/1.73m^2 the dose should be reduced to the 100 mg dose and the treatment stopped if the eGFR drops below 30 mL/min/1.73m^2. Furthermore because of the low glycaemic effectiveness if the eGFR drops below 45 mL/min/1.73m^2 then additional glycaemic lowering medication may be required.

2. For treatment of diabetic kidney disease as add on to standard of care (e.g. angiotensin-converting enzyme inhibitors [ACEi] or angiotensin-II receptor blockers [ARBs]).

   Initiation can occur down to an eGFR 30 mL/min/1.73m^2. If urinary albumin-to-creatinine ratio (uACR) is >33.9 mg/mmol, canagliflozin can be continued, if started for this indication, down to the need to commence dialysis or renal transplantation.

Dapagliflozin (Forxiga) (12)

1. Dapagliflozin is indicated in adults for the treatment of insufficiently controlled type 2 DM as an adjunct to diet and exercise:
   - As monotherapy when metformin is considered inappropriate due to intolerance
   - In addition to other medicinal products for the treatment of type 2 DM

   For this indication, dapagliflozin can be initiated at a dose of 10 mg. In people with diabetes mellitus, the glucose lowering efficacy of dapagliflozin is reduced when eGFR is < 45 mL/min/1.73m^2, and is likely absent in people with severe renal impairment. Therefore, if eGFR falls below 45 mL/min/1.73m^2, additional glucose lowering treatment should be considered in people with diabetes mellitus.

2. Dapagliflozin is indicated in adults for the treatment of symptomatic chronic heart failure.

3. Dapagliflozin is indicated in adults for the treatment of CKD.

   It is not recommended to initiate treatment with dapagliflozin in people with an eGFR < 15 mL/min/1.73m^2.

Empagliflozin (Jardiance) (13)

1. Empagliflozin is indicated in adults for the treatment of insufficiently controlled type 2 DM as an adjunct to diet and exercise:

   - As monotherapy when metformin is considered inappropriate due to intolerance
   - In addition to other medicinal products for the treatment of diabetes

   When used for treatment of insufficiently controlled type 2 DM, empagliflozin should be initiated at a dose of 10mg. If further glycaemic control is required, the dose can be increased to 25 mg a day if above an eGFR of 60 mL/min/1.73m^2. If the eGFR drops between 30 and 60 mL/min/1.73m^2 then the dose needs to be reduced to 10 mg a day, and the treatment stopped when the eGFR drops below 30 mL/min/1.73m^2.

2. Empagliflozin is indicated in adults for the treatment of symptomatic chronic heart failure.

   For treatment of heart failure in people with or without type 2 DM, empagliflozin 10 mg may be initiated or continued down to an eGFR of 20 mL/min/1.73 m^2

Ertugliflozin (Steglatro) (14)

1. Ertugliflozin is indicated in adults aged 18 years and older with type 2 DM as an adjunct to diet and exercise:
   - As monotherapy in people for whom the use of metformin is considered inappropriate due to intolerance or contraindications
   - In addition to other medicinal products for the treatment of diabetes

   Currently ertugliflozin can be initiated at a dose of 5mg. If further glycaemic control is required, the dose can be increased to 15 mg a day if above an eGFR of 45 mL/min/1.73m^2. Ertugliflozin should not be initiated at an eGFR of <45 mL/min/1.73m^2, but if already established on treatment, it may be continued down to an eGFR of 30 mL/min/1.73m^2. Treatment should be stopped when the eGFR drops below 30 mL/min/1.73m^2.

4.3 References

1. Wanner C, Inzucchi SE, Lachin JM, Fitchett D, von Eynatten M, Mattheus M, et al. Empagliflozin and Progression of Kidney Disease in Type 2 Diabetes. New England Journal of Medicine. 2016;375(4):323-34.
2. Perkovic V, Jardine MJ, Neal B, Bompoint S, Heerspink HJL, Charytan DM, et al. Canagliflozin and Renal Outcomes in Type 2 Diabetes and Nephropathy. New England Journal of Medicine. 2019;380(24):2295-306.
3. McMurray JJV, Solomon SD, Inzucchi SE, Køber L, Kosiborod MN, Martinez FA, et al. Dapagliflozin in Patients with Heart Failure and Reduced Ejection Fraction. New England Journal of Medicine. 2019;381(21):1995-2008.
4. Packer M, Anker SD, Butler J, Filippatos G, Pocock SJ, Carson P, et al. Cardiovascular and Renal Outcomes with Empagliflozin in Heart Failure. New England Journal of Medicine. 2020;383(15):1413-24.
5. Heerspink HJL, Stefánsson BV, Correa-Rotter R, Chertow GM, Greene T, Hou F-F, et al. Dapagliflozin in Patients with Chronic Kidney Disease. New England Journal of Medicine. 2020;383(15):1436-46.
6. Anker SD, Butler J, Filippatos G, Ferreira JP, Bocchi E, Böhm M, et al. Empagliflozin in Heart Failure with a Preserved Ejection Fraction. New England Journal of Medicine. 2021;385(16):1451-61.
7. Solomon SD, McMurray JJV, Claggett B, de Boer RA, DeMets D, Hernandez AF, et al. Dapagliflozin in Heart Failure with Mildly Reduced or Preserved Ejection Fraction. New England Journal of Medicine. 2022;387(12):1089-98.
8. EMPA-KIDNEY Collaborative Group. Empagliflozin in Patients with Chronic Kidney Disease. New England Journal of Medicine. 2022.
9. Zynquista: https://www.ema.europa.eu/en/medicines/human/EPAR/zynquista (accessed 14th December 2022).
10. National Institute for Health and Care Excellence (NICE). Sotagliflozin with insulin for treating type 1 diabetes (TA622). Available at: https://www.nice.org.uk/guidance/ta622 (Accessed 3rd January 2023).
11. Napp Pharmaceutical Limited, 01 Jan 2021. Summary of Product Characteristics - Invokana (Canagliflozin). (Online). Updated:26 Nov 2021. Available at: https://www.medicines.org.uk/emc/product/8855/smpc (Accessed 08 December 2022).
12. AstraZeneca, 01 Jan 2021. Summary of Product Characteristics - Forxiga (Dapagliflozin). (Online). Updated: 06 Dec 2022. Available at: https://www.medicines.org.uk/emc/product/7607/smpc (Accessed 08 December 2022).
13. Boehringer Ingelheim, 01 Jan 2021. Summary of Product Characteristics - Jardiance (Empagliflozin). (Online). Updated Oct 2022. Available at: https://www.medicines.org.uk/emc/product/5441/smpc (Accessed 08 December 2022).
14. Merck Sharp & Dohme (UK) Limited, 21 Mar 2018. Summary of Product Characteristics – Steglatro (Ertugliflozin). (Online). Updated 05 Apr 2022. Available at: https://www.medicines.org.uk/emc/product/10099/smpc (Accessed 08 December 2022).

Section 5: Prescribing SGLT-2 inhibitors safely

All medications have both beneficial and adverse effects. This section is designed to highlight the key adverse effects identified to result from use of sodium glucose co-transporter-2 (SGLT-2) inhibitors and guide how these medicines can be initiated and continued safely, minimising the risk of harm.

Since the publication of the 2021 version of the UKKA guidelines for use of SGLT-2 inhibition, findings from two large randomised controlled trials (EMPA-KIDNEY (1) and DELIVER (2)) and an updated meta-analysis (3) have been reported, providing important new information on the efficacy and safety of SGLT-2 inhibition among people with chronic kidney disease (CKD).

A summary of absolute benefits and risks of SGLT-2 inhibition in people with CKD with or without diabetes recruited into the CKD trials of SGLT-2 inhibitors are presented in the collaborative meta-analysis co-authored by the Nuffield Department of Population Health Renal Studies Group (RSG) and the SGLT-2 Inhibitor Meta-Analysis Cardio-Renal Trialists Consortium (SMART-C) (3). This summary, which presents absolute effects of SGLT-2 inhibition on kidney disease progression, hospitalisation for heart failure or death from cardiovascular causes, acute kidney injury, ketoacidosis, and amputation, is presented below. Importantly, the benefits of SGLT-2 inhibitors in terms of cardiovascular and renal outcomes consistently outweigh the risks of amputation or ketoacidosis among the types of people with CKD studied in the completed trials to date (3). A more detailed summary of evidence for the safety outcomes will be discussed this section.

Figure 5.1: Absolute benefit and risks of SGLT-2 inhibition for people with CKD with and without diabetes, estimated from 13 large randomised clinical trials of SGLT-2 inhibition (adapted from (3)).

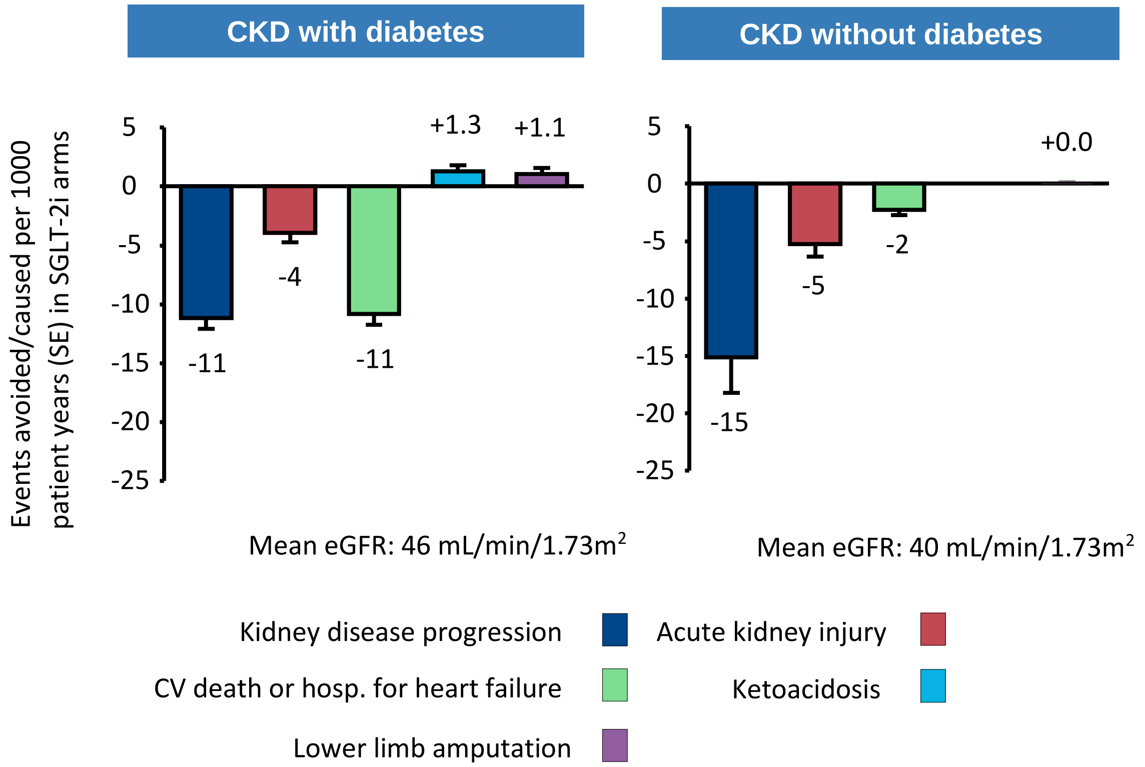

CKD – chronic kidney disease. eGFR – estimated glomerular filtration rate. SE – standard error. SGLT-2i – sodium-glucose co-transporter 2 inhibitor.

5a. Diabetic ketoacidosis

5a.1 Background and evidence review

Pathophysiology

SGLT-2 inhibition induces glycosuria, which causes widespread changes in metabolism, including an increase in lipid mobilisation, free fatty acid oxidisation and increased plasma ketone levels, in particular β-hydroxybutyrate and acetoacetate (4, 5). States of relative insulin deficiency or reduced carbohydrate intake augment hepatic ketogenesis. Therefore factors such as infection, fasting or reduction in insulin levels can precipitate diabetic ketoacidosis (DKA) in people treated with SGLT-2 inhibitors. Risk is highest in those who require prescription of insulin, and incredibly low among people without DM.

Relative insulin deficiency and reduced carbohydrate intake, with concomitant carbohydrate deficit related to glycosuria contributes to normal or near normal glycaemia (6), making it possible for ketoacidosis to be present with normal or low capillary blood glucose levels (sometimes referred to as "euglycaemic" ketoacidosis) (5).

Large trial evidence

In the RSG and SMART-C meta-analysis of large randomised trials of SGLT-2 inhibition, the risk of DKA was increased approximately two-fold by SGLT-2 inhibition (3). Because the baseline risk of ketoacidosis was so low in people with type 2 diabetes, the excess absolute risk of DKA among people with CKD and diabetes was around 1 DKA event per 1000 person-years treated with an SGLT-2 inhibitor, an approximate order of magnitude less than the benefits in terms of kidney disease progression in this population (for whom 1000 person-years of treatment with an SGLT-2 inhibitor would on average prevent 11 kidney disease progression events)(see figure 5.1 above). Among the large SGLT-2 inhibitor trials, type 1 DM was generally excluded (with the exception of EMPA-KIDNEY, which recruited a small number of participants with type 1 DM (1)). More information regarding risk of DKA in people with type 1 DM is discussed in Section 7a.

People with chronic kidney disease

The CREDENCE trial included participants with CKD with estimated glomerular filtration rate (eGFR) of 30-90 mL/min/1.73m^2 and reported a statistically significant increased risk of DKA. Although the relative risk for DKA was 10.8 (95%CI 1.39-83.7), DKA was rare (canagliflozin 11/2200 vs placebo 1/2197), and the absolute excess risk in the canagliflozin group was ~2.0 per 1000 person years (7), meaning the absolute benefits clearly exceeded the DKA risk.

The DAPA-CKD trial included participants with eGFR between 25-75 mL/min/1.73m^2, 68% of whom had type 2 DM (8). There was no increased risk of DKA in the SGLT-2 inhibitor arm of this trial (dapagliflozin 0/2152 vs placebo 2/2152).

The EMPA-KIDNEY trial included 6609 participants with eGFR between 20-65 mL/min/1.73m^2 of whom 46% had diabetes (including 68 participants with type 1 diabetes) (1). DKA was numerically more frequent in the empagliflozin group (6/3304, vs 1/3305 in the placebo group), though there were too few events to assess statistical significance. One DKA event occurred in a participant with type 1 DM, while 4 events occurred in

people with insulin-treated type 2 DM.

Physiologically, as eGFR reduces, the amount of glucose filtered by the glomeruli also reduces (9). The glucose-lowering action of SGLT-2 inhibition is therefore limited by eGFR and one can argue that this could protect people from the glycosuria-induced metabolic changes that increase the risk of DKA. However, it is also established that as eGFR reduces, insulin and sulphonylurea (SU) clearance is reduced and the risk of hypoglycaemia from these medications increases (10, 11). Reactive reductions in insulin doses in order to reduce the risk of hypoglycaemia could conceivably have contributed to the risk of DKA seen in CREDENCE and other trials.

People without diabetes mellitus

Starvation ketoacidosis can occur in people without diabetes, but only one such case has been reported during about 30,000 participant years of follow-up of people with heart failure or CKD treated with an SGLT-2 inhibitor in the reported trials within the meta-analysis (1, 3).

Factors increasing risk of DKA

The Association of British Clinical Diabetologists have identified characteristics of people with type 2 DM that may place them at greater risk of developing DKA when using SGLT-2 inhibitors (15). These characteristics are highlighted in Table 5a.1. People with these characteristics may benefit from ketone monitoring, therefore, we suggest discussing with the diabetes team prior to initiating SGLT-2 inhibitors in such people (See Recommendation 5a.2.2).

Table 5a.1. People with type 2/3 DM at higher risk of DKA

People with HbA1c >86 mmol/mol (10%)	People with past history of DKA
BMI ≤27 kg/m^2 (adjusted for ethnicity)	The possibility of Latent Autoimmune Diabetes in Adults (known as LADA)*
Excess alcohol consumption/ dependence	Known pancreatic exocrine/endocrine dysfunction – particularly if DM is a result of pancreatic disease (Type 3 DM)
People who have rapidly progressed to requiring Insulin (within 1 year of diagnosis)	

Footnote: * Latent Autoimmune Diabetes in Adults – suspect if type of DM unclear, type 2 DM and responding poorly to oral hypoglycaemic drug therapy or low BMI. These people may benefit from specialist input and glutamic acid decarboxylase antibody testing (16). BMI=Body Mass Index; DKA=Diabetic ketoacidosis; DM=Diabetes mellitus; HbA1c=Glycosylated haemoglobin.

5a.1.2 Sick day guidance

Medicines and Healthcare products Regulatory Agency (MHRA) reports of DKA suggest that concomitant illnesses such as vomiting, dehydration, reduced food intake, infection or a surgical procedure preceded some DKA events (17). Similar to the MHRA reports, the U.S Food and Drug Administration (FDA) statement in 2015 stated that 50% of the cases presenting with DKA were associated with precipitating events (18).

Following appropriate sick day guidance may significantly reduce the risk of developing DKA on SGLT-2 inhibitors.

It is therefore important that individuals initiated on SGLT-2 inhibitors are given sick day guidance on what to do in these situations, including people without diabetes.

Sick day guidance is highlighted below:

- Hold SGLT-2 inhibitor if unwell, restricted food intake, or dehydration
- Individuals on insulin treatment should always be advised never to stop or significantly reduce their insulin as part of the sick day response
- SGLT-2 inhibitor treatment should be interrupted in people who are hospitalised for surgical procedures or serious medical illnesses
- Treatment should be restarted when the person's condition has recovered

During periods of planned restricted food intake (for example, fasting for Ramadan), we suggest following the guidance by the ADA/EASD 2020 consensus update on the management of DM during Ramadan. If unwell during fasting, ketone testing should be considered, and for the elderly, those with CKD or those on diuretics, consider stopping or reducing dose of SGLT-2 inhibitor during the period of fasting (19).

5a.2 Recommendations for implementation

1. **We recommend that people with type 1 DM should only have SGLT-2 inhibitors initiated under the strict direction of the diabetes team (see section 7a) (Grade 1C).**

2. **We recommend that people with type 2 DM at greater risk of DKA (defined in Table 5a.1) should have SGLT-2 inhibitors initiated with caution after discussion with the diabetes team (Grade 1C).**

3. **We recommend SGLT-2 inhibitors are discontinued when an individual develops DKA (Grade 1A).**

4. **We suggest that after an episode of DKA and where a clear contributing factor has been identified, there should be discussion with the person and clinical team to establish whether the benefits of re-introducing an SGLT-2 inhibitor outweigh the risks (Grade 2D).**

5. **When initiating SGLT-2 inhibitors, we suggest that individuals should be advised on the signs and symptoms of DKA and be instructed to temporarily withhold SGLT-2 inhibitors and to seek immediate medical advice if symptoms develop (Grade 1C).**

6. **We recommend always offering advice on sick day guidance when initiating SGLT-2 inhibitors and reminding them of this at every medication review (Grade 1C).**

7. **We suggest that individuals taking SGLT-2 inhibitors should be advised against following a ketogenic diet (Grade 2C).**

8. **We suggest that for people who choose to intermittently fast (e.g. for Ramadan), and particularly for those who are elderly, on diuretics or have CKD, consider withholding SGLT-2 inhibitors for the duration of the fasting period and for those people with diabetes ketone testing should be undertaken if unwell (Grade 2D).**

Rationale: The evidence from the studies reviewed indicates that DKA is a recognised complication in people treated with SGLT-2 inhibitors and that it is more commonly found in conjunction with dehydration or infection. DKA is also likely to occur more frequently in people who are insulin deficient which would include people with type 1 DM, people with type 2 DM with a relative insulin deficient phenotype, and situations where people on insulin have their insulin dose reduced substantially. These recommendations will allow clinicians to use SGLT-2 inhibitors in those who are likely to benefit from this treatment and yet also minimise the risk of the complication of DKA.

5a.3 Audit measures

1. The proportion of people with CKD on SGLT-2 inhibitors with evidence of provision of sick day guidance.

2. The proportion of people with CKD in whom SGLT-2 inhibitors were withheld during acute illness, and the proportion appropriately re-initiated on recovery.

5b. Hypoglycaemia

5b.1 Background and evidence review

Pathophysiology and trial data

SGLT-2 inhibition does not increase the risk of hypoglycaemia when used in isolation or when combined with metformin, pioglitazone, dipeptidyl peptidase-4 inhibitors (DPP-4i) or glucagon-like peptide-1 receptor agonist (GLP-1RA) therapy (20). Results from the large placebo-controlled trials suggest they may even help reduce risk of hypoglycaemia. DECLARE-TIMI 58 and DAPA-CKD found that severe hypoglycaemia (plasma glucose <70 mg/dL or <3.9 mmol/L or hypoglycaemia requiring assistance) was more frequent in placebo than in the SGLT-2 inhibitor groups (8, 21), whilst results from EMPA-REG OUTCOME (22), VERTIS CV (23) and the other trials conducted in CKD populations (CREDENCE, EMPA-KIDNEY and SCORED) found no significant excess of hypoglycaemia (1, 7, 24).

The CREDENCE trial reported all hypoglycaemia (of any severity) and found no excess in its population of people with type 2 DM and albuminuric diabetic nephropathy (hazard ratio [HR]=0.92, 95%CI 0.77-1.11; absolute difference -4.6 per 1000 person years) (7). DAPA-CKD reported a reduction in the risk of severe hypoglycaemia (14/2149 vs 28/2149: an absolute excess among those allocated placebo of about 2.7 per 1000 person years) (8). Despite the additional effect of sotagliflozin inhibiting SGLT-1, there was no suggestion of increased risk of severe hypoglycaemia in SCORED (53/5291 vs 55/5286) (24). EMPA-KIDNEY reported no difference in severe hypoglycaemia between groups (77/3304 vs 77/3305) (1).

It is possible that reductions in doses of other hypoglycaemia-inducing therapies (i.e. insulin/sulphonylurea [SU]) to mitigate against perceived risk of hypoglycaemia associated with an additional glycaemia-lowering agent may explain these modest benefits on severe hypoglycaemia observed in the placebo-controlled large SGLT-2 inhibitor trials.

No incidence of severe hypoglycaemia was reported by the 5877 people without DM included in EMPEROR-REDUCED, DAPA-HF or DAPA-CKD (DAPA-CKD included 1398 people with CKD without DM) (8, 12, 13). Among people without diabetes recruited to EMPEROR-PRESERVED (representing 51% of the total population) there were 4 cases of hypoglycaemia episodes requiring assistance (2/1531 vs 2/1518 in empagliflozin and placebo arms respectively) (25). DELIVER reported similar incidence of major hypoglycaemia in the treatment and placebo groups (6/3126 vs 7/3127 respectively), but data from the DELIVER trial stratified by diabetes status are not currently available (2).

Insulin in combination with SGLT-2 inhibition

Insulin therapy is associated with an increased risk of hypoglycaemia in people with DM. Meta-analyses and observational data suggest that, when added to insulin therapy, SGLT-2 inhibition does not increase this risk of hypoglycaemia following a 10-20% reduction in total daily insulin dose (26, 27). Insulin doses were also reduced by up to 20% to prevent hypoglycaemia in people with type 1 DM in the SGLT-2 inhibitor arm of the DEPICT trials (28, 29). Any further insulin dose reduction (beyond 20%) should be cautious and targeted at avoiding hypoglycaemia (30), as excessive insulin dose reduction may increase risk of DKA. Those with more labile blood glucose control may benefit from discussion with the diabetes team for consideration of ketone monitoring (see section 5a).

Sulphonylurea (SU) and meglitinide in combination with SGLT-2 inhibition

Insulin secretagogues, whether used as monotherapy or in combination with other glucose-lowering drugs, are associated with an increased risk of hypoglycaemia. Meta-analyses of relatively short-term phase 3 placebo-controlled studies found an excess risk of hypoglycaemia when SGLT-2 inhibitors are added to metformin and SU (Odds Ratio=1.75, 95%CI 1.43-2.15) (31), but did not consider the impact of CKD. Conversely, the large clinical outcome trials found no excess risk of severe hypoglycaemia with SGLT-2 inhibition (with several reporting reductions in risk). This has generated some clinical uncertainty leading to variation in clinical practice, with some clinicians recommending SU doses are reduced when starting SGLT-2 inhibitors, and others proposing SUs should be stopped altogether (30). It should be remembered that in people with an eGFR <45 mL/min/1.73m^2, SGLT-2 inhibition has only modest effects on glucose lowering (32).

There is very little evidence for the use of SGLT-2 inhibitors in combination with meglitinides. However, as the risk of hypoglycaemia with meglitinide use is increased in people with advanced CKD (33), we do recommend consideration of meglitinide dose reductions when initiating SGLT-2 inhibitors.

5b.2 Recommendations for implementation

1. **We recommend considering reducing the dose of insulin/SUs/meglitinides when initiating SGLT-2 inhibitors to reduce the risk of hypoglycaemia (Grade 1C).**

2. **We recommend that when initiating SGLT-2 inhibitors in people taking SUs (e.g. gliclazide) or meglitinides (e.g. repaglinide) when the HbA1c <58 mmol/mol AND eGFR >45 mL/min/1.73m^2, consider reducing dose of SU or meglitinide by 50% to reduce risk of hypoglycaemia (Grade 1C).**

3. **We recommend that when starting SGLT-2 inhibitors in people taking insulin when the HbA1c <58 mmol/mol AND eGFR >45 mL/min/1.73m^2, consider reducing the insulin dose by 20% to avoid hypoglycaemia (Grade 1C).**

4. **We recommend that when starting SGLT-2 inhibitors in people taking only metformin ± pioglitazone ± DPP-4i/gliptins or GLP-1RA therapy, no dosage adjustment is necessary (Grade 1C).**

Rationale: SGLT-2 inhibitors are effective drugs at reducing hyperglycaemia when they are used in people with preserved kidney function (e.g. eGFR >60 mL/min/1.73m^2), however, their glycaemic effectiveness reduces as the eGFR declines. Where a treatment for DM carries a risk of hypoglycaemia (such as SUs and insulin use), the addition of an SGLT-2 inhibitor may potentiate that risk, particularly if baseline glycaemic control is reasonable at the time of initiation of treatment. There is no evidence that SGLT-2 inhibitors cause significant hypoglycaemia on their own or in addition with DM medicines that are not associated with hypoglycaemia.

5b.3 Audit measures

1. The proportion of people on Insulin/SUs with HbA1c <58 mmol/mol and eGFR >45 mL/min/1.73m^2, whose therapy was appropriately reduced when initiating SGLT-2 inhibitors.

5c. Acute Kidney Injury, Hypovolaemia and Potassium

5c.1 Background and evidence review

Acute kidney injury

The introduction of SGLT-2 inhibitors was accompanied by early concerns that their use may be linked to an increased risk of acute kidney injury (AKI) and volume depletion. This was largely driven by specific features of their mechanism of action, including an initial reduction in eGFR (which is, thereafter, followed by stabilisation of eGFR slope and improved kidney outcomes compared to placebo), induction of osmotic diuresis and natriuresis, alongside post-marketing reports of AKI events following their initiation. Of note, more than half of these reported AKI events occurred within the first 4 weeks of initiation. The US FDA dictated caution to health care professionals with regards to their use, especially in the context of other factors that may predispose to AKI such as CKD, heart failure and certain pharmacological agents such as angiotensin-converting enzyme inhibitors (ACEi), angiotensin-II receptor blockers (ARBs) and diuretics (34).

Since then, randomised placebo-controlled trials, including participants considered at high risk of AKI, have consistently demonstrated that SGLT-2 inhibition reduces, rather than increases, the risk of AKI. The recent meta-analysis by the RSG and SMART-C suggest reduced risk of AKI is now counted as a beneficial effect of SGLT-2 inhibition. After standardising analyses (wherever possible) to the MedDRA Preferred Term of "Acute Kidney Injury", the meta-analysis demonstrated that SGLT-2 inhibition reduced the risk of AKI by 23% compared to placebo, independently of diabetes status, inclusive of people with heart failure and CKD, and unmodified by trial-level average eGFR (3). Such a reduction in risk translated to an estimated 4 fewer AKI events per 1000 person-years of SGLT-2 inhibition treatment among people with diabetes, or 5 fewer AKI events per 1000 person-years among people without diabetes (see Figure 5.1 above).

Initiation of SGLT-2 inhibitors is followed by a reduction in eGFR which is inherent to their mechanism of action (7, 8, 35, 38, 40, 41), accompanied by stabilisation of the eGFR slope within weeks (7, 35, 40, 41), and appears to be largely reversible upon discontinuation (36). In CREDENCE, the reported average eGFR decrease at 3 weeks was 3.72 ±0.25 mL/min/1.73m^2 versus 0.55 ±0.25 mL/min/1.73m^2 in the canagliflozin and placebo group, respectively, while in DAPA-CKD the eGFR decline at 2 weeks was 3.97 ±0.15 mL/min/1.73m^2 in the dapagliflozin versus 0.82 ±0.15 mL/min/1.73m^2 in the placebo group (7, 8). This initial eGFR decline, also referred to as 'eGFR dip', does not appear to have any clinical impact on AKI risk and does not appear to modify benefits or risks of treatment. In a post-hoc analysis of EMPA-REG OUTCOME, factors like diuretic use and worsening KDIGO CKD stage appear to predispose to a larger (>10%) eGFR dip 4 weeks after initiation of empagliflozin (42). Regardless, eGFR stabilised after 4 weeks and in the study the eGFR dip resolved upon discontinuation of study treatment. The treatment-mediated cardiovascular and renal benefits were not modified by the presence of a more pronounced eGFR dip, and eGFR remained stable from week 12 onward in all 'eGFR dipping' categories. In CREDENCE, the extent of eGFR drop did not affect the long-term change in eGFR slope, or the safety and tolerability of treatment (43). Likewise, an exploratory analysis of DAPA-CKD did not show any relationship between the magnitude of eGFR dip and kidney disease progression, hospitalisation for heart failure or cardiovascular death, or all-cause mortality, though there was evidence of attenuation of the chronic eGFR slope among dapagliflozin-treated participants in the highest category of eGFR dip (>10% reduction in eGFR) (44).

Hypovolaemia

CANVAS reported an increased rate of volume depletion events with canagliflozin compared to placebo (26.0 vs 18.5/1000 person years; p=0.009) (37), while CREDENCE found no significant excess in people with albuminuric diabetic kidney disease (28.4 vs 23.5/1000 person years) (7). More frequent episodes of volume depletion were reported with the use of dapagliflozin compared to placebo in DAPA-CKD (5.9 vs 4.2%; p=0.001) (8), but no significant excess was apparent in DECLARE-TIMI 58 (2.5 vs 2.4%) (21). Rates of adverse events consistent with hypovolaemia did not differ between the empagliflozin and placebo arms in EMPA-REG OUTCOME overall (35) or by eGFR categories (eGFR <60 vs ≥60 mL/min/1.73m^2) (36), with similar findings for ertugliflozin in VERTIS CV (23). Serious or symptomatic dehydration events were comparable between empagliflozin (30/3304, 0.9%) and placebo (24/3305, 0.7%) arms in EMPA-KIDNEY (1).

In most of the large heart failure trials (2, 12, 13, 45, 46), hypovolaemia-related adverse event rates were similar between the treatment and placebo arms, with the vast majority of participants concurrently prescribed other diuretics. EMPEROR-PRESERVED found that hypotension (10.4% vs 8.6%) and symptomatic hypotension (6.6% vs 5.2%) were higher in the empagliflozin group than in the placebo group (14) , while the DELIVER trial showed similar rates of serious adverse events or adverse events leading to discontinuation suggestive of dehydration in the dapagliflozin and placebo arms (1.3% vs 1.0%) (2). In a subgroup analysis of data from DAPA- HF by diuretic dosage, volume depletion events were more common with dapagliflozin than with placebo in participants on the higher dose diuretics (47), however, there was no increase in renal adverse events.

Sotagliflozin, which also inhibits gut SGLT-1 and can cause diarrhoea, resulted in significantly more frequent volume-related adverse events than placebo in the SCORED trial in CKD and type 2 DM (24) (5.3 vs 4.0%; p=0.003), but with no significant excess reported in those with recent hospitalisation for worsening heart failure in SOLOIST-WHF (45) (9.4% vs 8.8%).

In a meta-analysis of the data from some of these large trials combined with a series of smaller trials, there was an increased risk of hypovolaemia-related trial adverse event reports with use of SGLT-2 inhibitors (OR=1.20, 95%CI 1.10-1.31) (48).

Potassium

Combining an SGLT-2 inhibitor with an ACEi or ARB does not have the same potential as dual renin-angiotensin system (RAS) blockade to cause hyperkalaemia (12, 13, 49). There were no meaningful differences in potassium between treatment groups on serial measurements in the CANVAS trial (49). There were also no reported significant differences in adverse events for hyperkalaemia between treatment groups in the large placebo-controlled CKD trials of SGLT-2 inhibitors (in which the majority of participants were treated with single RAS blockade). CREDENCE reported hyperkalaemia event rates of 29.7/1000 person-years among people allocated canagliflozin vs 36.9 events/1000 person-years for those allocated placebo (7). In DAPA-CKD, there were 6 (0.3%) serious adverse events of hyperkalaemia among those allocated dapagliflozin versus 12 (0.6%) among those allocated placebo (8). EMPA-KIDNEY reported serious hyperkalaemia among 92/3304 and 109/3305 participants treated with empagliflozin and placebo respectively (HR 0.83, 95%CI 0.63-1.09) (1).

Data from HFrEF populations are similarly reassuring, with no effect of SGLT-2 inhibitors on laboratory measurements of potassium or clinical events of hyperkalaemia overall, or among those co-prescribed

mineralocorticoid receptor antagonists (MRA) (50, 51). These subanalyses from DAPA-HF and EMPEROR-REDUCED have generated hypotheses that SGLT-2 inhibition may even reduce the risk of severe hyperkalaemia among MRA users or lead to fewer discontinuations of MRA. Consistent with these findings, a large individual participant data meta-analysis of participants with diabetes from six SGLT-2 inhibitor trials, including four trials in enrolling participants with diabetes and high cardiovascular risk and two trials enrolling participants with CKD, demonstrated a significant 16% reduction in serious hyperkalaemia (95%CI 7% to 24%) among people treated with SGLT-2 inhibitors (52). The reduction in hyperkalaemia was present and of a similar magnitude across the spectrum of kidney function, including among people with eGFR <45 mL/min/1.73m^2, and was not accompanied by an increased risk of hypokalaemia. Section 2 provides further details of data on hyperkalaemia with SGLT-2 inhibition among those with heart failure, including MRA users.

5c.2 Recommendations for implementation

1. **We recommend that individuals initiated on an SGLT-2 inhibitor do not routinely require an early assessment of kidney function or serum potassium following initiation of treatment (Grade 1C).**
2. **We suggest that if an individual has a kidney function assessment within the first few weeks post initiation of an SGLT-2 inhibitor, a decline in eGFR needs to be interpreted with caution and in the context of an expected drug effect to avoid unwarranted discontinuation of treatment (Grade 2B).**
3. **We suggest that individuals on diuretics are counselled on the symptoms of hypovolaemia and advised to seek medical attention if they develop any such symptoms after starting SGLT-2 inhibition (Grade 2B).**
4. **We suggest that clinicians consider an early clinical review and if appropriate a diuretic or antihypertensive dose reduction in individuals they consider at high risk of hypovolaemia (Grade 2C).**
5. **We recommend that SGLT-2 inhibitors are temporarily withheld during acute illness (Grade 1C).**

Rationale: SGLT-2 inhibitors have proven benefit in relation to reducing the rate of long-term decline in kidney function in certain groups of people with CKD. The means by which they provide this benefit may involve changes to intraglomerular pressure and reduction in hyperfiltration at an individual glomerulus level. This can result in a reduction in eGFR over the initial few weeks following initiation of SGLT-2 inhibitors, which is relatively small, largely reversible and should not usually be seen as an adverse effect of the drug.

None of the major studies have demonstrated an increased risk of AKI in people treated with SGLT-2 inhibitors, and it seems likely they have renal tubular protective effects that reduce risk of AKI. It is therefore important that early changes in eGFR that occur following initiation of SGLT-2 inhibitors do not routinely result in withdrawal of SGLT-2 inhibition when people are likely to gain significant benefit from them.

In addition, SGLT-2 inhibitors have a combined osmotic diuretic and natriuretic effect, so clinicians and the people treated with SGLT-2 inhibitors need to be aware of this effect in order to ensure that any risk of hypovolaemia is minimised.

5d. Peripheral vascular disease and amputation risk

5d.1 Background and evidence review

An interim safety analysis of the CANVAS trial (37) raised concern over an increased amputation signal with canagliflozin which led to protocol amendments to the contemporaneously recruiting CREDENCE trial (7) to exclude those with recent amputation history and to interrupt therapy in the event of foot disease onset. However, whilst the CANVAS trial reported an almost two-fold increased risk of amputation with canagliflozin (HR=1.97, 95%CI 1.41-2.75), no significantly increased amputation risk was detected in CREDENCE (HR=1.11, 95%CI 0.79-1.56). Amputation events in the CANVAS trial were significantly increased for both major (ankle and above) and minor amputation, but the majority (71%) were minor (predominantly toe amputations).

Relative risks of amputation in CANVAS were also similar across a range of subgroups, including history of peripheral vascular disease (PVD), prior amputation, and eGFR <60 vs ≥60 mL/min.1.73m^2. Secondary analyses of CANVAS and CREDENCE have failed to identify any participant or trial factors to explain the difference in reported amputation risk, with the aforementioned late protocol amendment (implemented about three quarters of the way through recruitment) not thought to have contributed to the absence of amputation signal in CREDENCE (53, 54).

The RSG and SMART-C meta-analysis (3) investigated the risk of lower limb amputation overall and by diabetes status. After excluding the hypothesis-generating CANVAS trial, there was no significant increase in risk of amputation identified in the other 12 trials (relative risk 1.06, 95%CI 0.93-1.21) with evidence for statistical heterogeneity between CANVAS vs these other 12 trials (p=0.0007). There was also no evidence that the risk of lower limb amputation was modified by trial-level eGFR or by diabetes status. When assessing absolute risks of SGLT-2 treatment using the conservative 15% increased risk (representing a pessimistic estimate of risk from analysis of all 13 trials including CANVAS), SGLT-2 inhibition would cause an estimated 1.1 amputation events per 1000 person years of treatment among people with CKD and diabetes, while an estimated 11 kidney disease progression events, 4 AKI events, and 11 hospitalisations for heart failure or cardiovascular events would be avoided (Figure 5.1). Due to the considerably lower absolute risk of amputation among people without diabetes, the absolute number of amputations caused per 1000 person years is estimated to be small compared to the absolute benefits in the studied populations where there are indications for use of SGLT-2 inhibitors.

Our recommendation is to avoid initiation of SGLT-2 inhibitors in individuals with active foot disease and withhold SGLT-2 inhibitors should this complication arise. We also stress the importance of shared decision making in initiating SGLT-2 inhibitors, and reinstating use following resolution of foot complications, recognising that individuals at high risk of amputation may also stand to gain significant cardiorenal benefit from these agents as outlined above. Given the absence of amputation signal seen in CREDENCE, there is insufficient evidence to disadvantage one of the SGLT-2 inhibitors over the other for individuals at high risk of amputation events. However, attention to routine preventative foot care should be advised for all people with DM initiated on SGLT-2 inhibitors.

5d.2 Recommendations for implementation

1. **We suggest avoiding initiation of SGLT-2 inhibitors in the presence of active foot disease (infection, ulceration and ischaemia) and withholding treatment in those who develop foot complications whilst taking an SGLT-2 inhibitor (Grade 2B).**

2. **We suggest a shared decision-making approach, with appropriate counselling on risks and benefits of treatment and the importance of routine preventative foot care measures for:**

   - **Individuals at high risk of amputation (previous amputations, existing PVD, peripheral neuropathy)**
   - **Re-initiation of SGLT-2 inhibitors after treatment and satisfactory resolution of a foot complication that occurred whist taking SGLT-2 inhibitors (Grade 2B).**

Rationale: A significant finding from a single large trial using the SGLT-2 inhibitor canagliflozin alerted clinicians to the possibility that SGLT-2 inhibitors could increase the risk of lower limb amputations. This finding has not been confirmed in other large trials and furthermore it is important to appreciate that people with PVD are a group of individuals who have more to gain from the initiation of SGLT-2 inhibitors in relation to protection against risk of cardiovascular death, myocardial infarction, heart failure complications and progression of CKD. It is therefore important not to exclude these individuals from the potential benefits of SGLT-2 inhibitors, but to ensure that these medicines are used appropriately and safely in people at risk, or with evidence of PVD.

5e. Fracture risk

5e.1 Background and evidence review

A safety notice for fracture risk with SGLT-2 inhibitors was published following increased incidence of upper and lower limb fracture in the CANVAS trial of canagliflozin (HR=1.26, 95%CI 1.04-1.52). The excess fracture risk was detected in only one of the two large subcohorts that comprise the CANVAS Program of trials (i.e. it was not apparent in CANVAS-R), for reasons that could not be explained by baseline participant demographic or protocol heterogeneity, and could conceivably represent a finding which resulted from the play of chance (37). A fracture risk with canagliflozin was not identified in the CREDENCE trial of canagliflozin, nor has it been identified in large placebo-controlled trials of other SGLT-2 inhibitors (including those inclusive of participants with low eGFR) (1-3, 8, 12, 13, 21-24, 45). Outside of trial populations, a recent systematic review of 37 large population-based studies found no association between SGLT-2 inhibitor prescription and fractures (55). A hypothesised link to hypovolaemia-related falls has not been substantiated, although the incidence of non-serious falls is often not recorded in large outcome trials (56).

Data from preclinical and phase I studies have reported short-term alterations in mineral biochemistry, including increases in phosphate, FGF-23 and parathyroid hormone (PTH) levels with SGLT-2 inhibitor use (57, 58), which were replicated in a cohort of 31 participants with type 2 DM and albuminuria treated with dapagliflozin (59). A trial of canagliflozin versus placebo in older individuals with type 2 DM demonstrated significantly reduced bone mineral density at the hip, but at no other site, with canagliflozin treatment over 104 weeks (60).

Whilst experimental data indicate that SGLT-2 inhibitors may modify bone mineral metabolism, precise mechanisms have not been elucidated. Based on current evidence, it is likely that either these bone metabolism changes do not translate into increased fracture risk, or that any small increased fracture risk with canagliflozin is outweighed by the cardiorenal benefits in populations at risk of heart failure or progressive CKD. Our recommendation therefore highlights the importance of routine CKD-mineral bone disease (MBD) monitoring and management in these individuals. More research is required into the mechanisms of SGLT-2 inhibitor-induced bone biochemical alterations, potential interactions with other drugs that modify osteoporosis risk (e.g. thiazolidinediones) and the clinical significance of this in a CKD +/- type 2 DM population already at risk of bone disease.

5e.2 Recommendations for implementation

1. **In people with CKD treated with SGLT-2 inhibitors, we suggest monitoring of bone parameters including calcium, phosphate and PTH should be performed as appropriate for CKD stage (NICE NG203) (Grade 2D).**

Rationale: Whilst there has been report of an increased risk of fractures in one trial where participants were treated with canagliflozin, this has not been confirmed in any other study and may represent the play of chance. People with CKD are at increased risk of bone disease and their clinician should be monitoring them to ensure that interventions are utilised to maintain good bone health irrespective of the prescription of SGLT-2 inhibitors. NICE NG203 CKD guidance is available at https://www.nice.org.uk/guidance/ng203.

5e.3 Clinical research recommendations

1. Establishing any long-term impact of SGLT-2 inhibition on the development and progression of CKD-MBD.

2. Establishing if SGLT-2 inhibition modifies osteoporosis risk posed by thiazolidinediones.

5f. Multimorbidity and frailty

5f.1 Background and evidence review

Multimorbidity and frailty are interrelated but distinct conditions that are important to consider when individualising treatment decisions for SGLT-2 inhibitor prescription. Multimorbidity, (defined as the presence of two or more long-term health conditions) is common in the UK. In a cross-sectional study of 1.75 million people registered with a general practitioner in Scotland, 40% of people had single health conditions and 23% were multimorbid (61). In UK Biobank, which recruited half a million people between the ages of 40 and 69 years living in the UK, 19% of people were multimorbid (62).

The exclusion criteria of trials often result in underrepresentation of people with certain types of comorbidity from large placebo-controlled trials. This is arguably the case for many of the SGLT-2 inhibitor trials. The CANVAS, CREDENCE, EMPA-REG OUTCOME, DAPA-CKD, DAPA-HF, DECLARE-TIMI 58 and VERTIS CV trials all excluded participation where there was evidence of certain conditions (e.g. liver disease, cancer, and haematologic conditions). Such approaches may be justified by the need to ensure participants will survive long enough to be at risk of the studied outcomes. For example, progression of CKD can take years, and so exclusion of people with active cancer is necessary to ensure the trial can address its primary question. However, these exclusion criteria pose a challenge when aiming to generalise the evidence from large placebo-controlled trials to people with these conditions/multimorbidity. Of note, individual trials had additional exclusions which also limit generalisability. For example, people with prior or current immunosuppressive therapy, or people affected by endocrine diseases other than DM, and potentially at risk of certain safety outcomes (e.g. at risk of DKA or amputation) were excluded from many of the SGLT-2 inhibitor trials.

Two of the large SGLT-2 inhibitor trials in heart failure, DAPA-HF and DELIVER, investigated the role of frailty on treatment effects of dapagliflozin in heart failure with reduced, and mildly reduced or preserved, ejection fraction, respectively (63, 64). Both of these trials, in which ~50% and ~60% of individuals were categorised as frail respectively, demonstrated consistent results; specifically, that the risk of key clinical endpoints (including cardiovascular death or worsening heart failure) was higher among people with a higher frailty score, while the relative benefits of dapagliflozin were unmodified by frailty. Consequently, the estimated absolute benefits of dapagliflozin would be expected to be greater among more frail individuals. Adverse events were similar between dapagliflozin and placebo arms across the spectrum of frailty. However, such analyses do not mitigate the limitations of stringent exclusion criteria in randomised controlled trials, with the most frail individuals still likely to be excluded from such trials. Evidence among highly frail or multimorbid individuals therefore remains limited.

People with multimorbidity and frailty may suffer from a particularly high burden of treatment. They are at high risk for adverse drug reactions and, in the case of multimorbid people, may be considered for treatment with multiple different drugs for their different health conditions. Conversely, they may also be at high absolute risk of a trial's key efficacy outcomes and therefore particularly benefit from the effects of SGLT-2 inhibition on risk of cardiovascular death, heart failure complications, AKI and the reduced risk of hospitalisation observed in some of the SGLT-2 inhibitor trials.

For some indications for SGLT-2 inhibition, such as for the treatment of CKD in people without albuminuria, the benefits of SGLT-2 inhibition on CKD progression may be expected to accrue over a few years. Benefits on cardiac disease, however, emerge early. Therefore among frail individuals, the ratio of benefit to risk may

need to be considered, with particular emphasis on the *absolute* benefits and the duration of treatment anticipated for meaningful benefit to accrue.

Our recommendations for this group are in line with the UK guidelines for multimorbidity and frailty (Multimorbidity: clinical assessment and management, NICE Guideline NG56, Published September 2016) which places emphasis on individual preference, awareness of the potential burden of polypharmacy and consideration of life expectancy in balancing risks and benefits of SGLT-2 inhibitor treatment.

5f.2 Recommendations for implementation

1. We suggest an approach to care that takes account of frailty and multimorbidity where these apply. This can include:

- **Establishing the person's goals, values and priorities**
- **Consideration of the balance of disease and treatment burden (for example, prognostic benefits in people with limited life expectancy or frailty)**
- **Agreeing an individualised management plan (Grade 2D).**

Rationale: When making decisions on which individuals would benefit from SGLT-2 inhibition one has to consider the participants included in the relevant trials that provided the evidence for their use. These trials generally excluded people with greater degrees of frailty and certain comorbidities. Therefore, caution must be exercised when extending evidence of safety (and perhaps also benefit) of SGLT-2 inhibitors to such individuals, although one needs to also consider at the same time that many of these individuals, and particularly those with heart failure, are likely to achieve significant benefit from the use of SGLT-2 inhibitors.

5f.3 Clinical research recommendations

1. Future trials of SGLT-2 inhibitor use in people with CKD that seek to extend inclusivity to those of advanced age and multimorbid status.

5g. Mycotic genital infections and Fournier's gangrene

5g.1 Background and evidence review: Mycotic genital infections

SGLT-2 inhibitors reduce blood glucose in individuals with DM by causing urinary excretion of glucose (65). The presence of an increased concentration of glucose in the urine results in a 2.5-6-fold increase in the risk of mycotic genital infections in those on an SGLT-2 inhibitor compared to control (66-68). In women, this presents as candida vulvovaginitis and in men as balanitis.

The large placebo-controlled trials reported an increased incidence of mycotic genital infections in the SGLT-2 inhibitor treatment arms compared to placebo (Table 5g.1)

Table 5g.1 Mycotic genital infections in SGLT-2 inhibitor trials

Trial	SGLT-2 inhibitor (per 1000 person years)	Placebo (per 1000 person years)	p value
CANVAS Program (n = 10142)	68.8	17.5	<0.001
CREDENCE (n = 4397)	Male 5.8<br>Female 14	Male 0.8<br>Female 5.2	-
DAPA-CKD (n = 4298)	-	-	-
DAPA-HF (n = 4744)	-	-	-
DECLARE-TIMI 58 (n = 17143)	76	9.0	<0.001
DELIVER (n = 6263)	-	-	-
EMPA-KIDNEY (n = 6609)	0.2*	0.2*	-
EMPA-REG OUTCOME (n = 7020)	Male 13.1<br>Female 39.6	Male 4.8<br>Female 8.4	Male <0.001<br>Female <0.001
EMPEROR-PRESERVED (n = 5988)	67 (2.2)**	22 (0.7)**	-
EMPEROR-REDUCED (n = 3726)	12.5	4.8	-
SCORED (n = 10577)	17.7	6.4	<0.001
SOLOIST-WHF (n = 1216)	11.0	2.2	-
VERTIS CV (n = 8238)	Male 7.0-9.7<br>Female 30.7-33.6	Male 3.4<br>Female 6.3	Male <0.001<br>Female <0.001

*rate per 1000 person years estimated by multiplying rate per 100 person years by 10. ** Data presented as n (%); event rates not available. - = not reported.

People with type 2 DM are at an increased risk of recurrent mycotic genital infections compared with the general population. Given this increased incidence, propensity to mycotic genital infection is an important consideration prior to initiation of SGLT-2 inhibitors. Factors which may predispose an individual to mycotic genital infection include female sex, pregnancy, hormonal contraception, recent antibiotic use, and immunosuppression. People with DM not achieving their HbA1c target may be immunosuppressed as hyperglycaemia has effects on the immune system resulting in an increased susceptibility to infection (69).

When these risks factors are considered together in multivariate models, female gender, higher BMI and previous genital infections are independently associated with a greater risk of mycotic genital infections, whilst a high HbA1c is not consistently associated with greater risk (70, 71). Development of mycotic genital infections is a common reason to stop SGLT-2 inhibitors, particularly if they occurred early after treatment initiation (72). Counselling on the risks and prevention of mycotic genital infections may help improve adherence.

Prevention

Prevention comes mainly in the form of managing risk factors. Personal hygiene education has been shown to reduce incidence of genital infection in a small study of 250 people where 5% of participants who were advised on personal hygiene developed a genital infection compared to 41% of those in the control group. Personal hygiene strategies include rinsing after voiding (but not douching), loose fitting absorbent underwear and cleaning under foreskin (73).

Treatment

Despite good preventative measures, some individuals may still develop mycotic genital infections. Prescribing guidance suggests discontinuation of the SGLT-2 inhibitors is not necessary (74). People can self-manage with over the counter treatments or be prescribed appropriate antifungal therapy such as topical creams or a single dose of an oral antifungal (73). For those with recurrent infections, prophylactic or maintenance therapy is suggested (73).

5g.1.2 Background and evidence review: Fournier's gangrene

Fournier's gangrene is an acute polymicrobial infection of the scrotum, penis or perineum with necrosis. It presents with scrotal or perineal pain and redness and has a rapid progression to gangrene (with pain being a key feature). Treatment is surgical debridement and broad-spectrum intravenous antibiotics. It is rare, with an overall incidence of about 1.6 per 100,000/year (75). The large placebo-controlled trials of SGLT-2 inhibitors have reported only a few cases of Fournier's gangrene, with no suggestion of an increased incidence in the SGLT-2 inhibitor treatment arms compared to placebo (3) .

The MHRA advised of a risk of developing Fournier's gangrene whilst treated with SGLT-2 inhibitors in February 2019, following an EU review and an FDA safety announcement (76). Six yellow card reports were received corresponding to 548,565 person years of treatment. Warnings have been added to product information and letters were sent to health professionals. These warnings advise that people should be informed of the signs and symptoms and when to seek help. In particular, they should be advised to be alert for symptoms of severe pain, tenderness, erythema, or swelling in the genital or perineal area accompanied by fever or malaise. In addition, people on SGLT-2 inhibitors should be advised to stop their treatment on suspicion of Fournier's gangrene and treatment started urgently.

5g.2 Recommendations for implementation

1. **We recommend that all people are counselled on the risks of mycotic genital infections prior to initiation of SGLT-2 inhibitors (Grade 1D).**
2. **We recommend that all people are counselled on self-care to maintain good genital hygiene (Grade 1C).**
3. **We recommend that all people are counselled on the symptoms of mycotic genital infections and how to seek help including self-management (Grade 1D).**
4. **We suggest that for those individuals with a history of recurrent mycotic genital infections on SGLT-2 inhibition, consideration is given to offering prophylactic anti-fungal treatment, which should be reviewed after 6 months of therapy or earlier if clinically indicated (Grade 2D).**
5. **We suggest that SGLT-2 inhibitor therapy can be continued during the treatment of mycotic genital infections (Grade 2D).**
6. **We highlight the specific MHRA warning and suggest that all people are counselled on the symptoms of Fournier's gangrene and advised to stop SGLT-2 inhibitors and to seek urgent help if they develop such symptoms (Grade 2D).**

Rationale: Mycotic genital infections are recognised to occur more frequently in people treated with SGLT-2 inhibitors (on average risk is about 3-4-fold higher) and particularly in those individuals with DM. These infections are usually mild and easily treated. Good clinical care should include ensuring that individuals prescribed SGLT-2 inhibitors are aware of this complication, how to reduce the risk of it occurring and appropriate actions should they develop symptoms consistent with mycotic genital infections. In contrast to mycotic genital infections, Fournier's gangrene is a rare condition that results from bacterial infection and it requires prompt and intensive medical and surgical management. This disorder is identified in people with DM and whilst the evidence to suggest that it may be increased in people treated with SGLT-2 inhibitors is limited to post-marketing surveillance, all people starting SGLT-2 inhibitors should be advised on the symptoms of Fournier's gangrene and what to do if they develop such symptoms.

5h. Urinary tract infections

5h.1 Background and evidence review

DM is a known risk factor for urinary tract infections (UTIs), and this may be attributable to glycosuria enhancing bacterial growth in the urinary tract or to bladder dysfunction impairing complete bladder emptying (77, 78). The mechanism of action of SGLT-2 inhibitors suggests a theoretical increased risk of UTIs through the enhancement of glycosuria. In 2015, the FDA reported 19 cases of urosepsis and pyelonephritis in individuals on SGLT-2 inhibitor therapy between March 2013 and October 2014, and subsequently issued a warning surrounding the risk of UTIs with SGLT-2 inhibitor use (18).

Except for VERTIS CV and EMPEROR-PRESERVED, this finding is not reflected in the randomised data from the large placebo-controlled trials (1, 2, 7, 13, 14, 21, 23, 24, 35, 37, 45). It is notable that VERTIS CV did report a statistically significant increase in any UTIs in the ertugliflozin arm (absolute increase in risk with ertugliflozin 5mg vs placebo +2.1% over 3.5 years, 95%CI 0.4-3.7%; 15mg vs placebo, +1.8%, 95%CI 0.2-3.5%), but found no difference in the subset of these infections which were serious (<10% of UTIs were serious). In EMPEROR-PRESERVED, absolute UTI risk was increased by ~0.8% per year (3, 14).

Meta-analysis of the 13 large randomised trials in SGLT-2 inhibition demonstrate a small excess risk for UTI (RR 1.08, 95%CI 1.02-1.15), contributing to about 1 in 14 UTIs experienced in a population treated with SGLT-2 inhibitors (3). Meta-analysis limited only to serious UTI did not show a significant increase in risk (RR 1.07, 95%CI 0.90-1.27).

The lack of serious UTI risk with SGLT-2 inhibition might be explained by the hypothesis that any effects of glycosuria on potentiating bacterial growth are countered by those of diuresis and polyuria that prevent bacterial ascension of the urinary tract (79). Alternatively, glycosuria per se is actually not a common precipitant for UTIs.

In CKD, there is reassuring data (80). In CREDENCE, the event rate for UTI per 1000 person-years was 48.3 in the canagliflozin-treated group versus 45.1 in the placebo arm (HR=1.08, 0.90–1.29), and in SCORED, the event rate for UTI per 1000 person years was 86 in the sotagliflozin-treated group versus 83 in those allocated placebo (HR=1.04, 0.94-1.16) (7, 24). Rates of serious UTI in EMPA-KIDNEY were similar in empagliflozin and placebo groups (0.81 vs 0.84 events per 100 person years, respectively) (1).

5h.2 Recommendations for implementation

1. **We recommend temporary discontinuation of SGLT-2 inhibitors when treating acute pyelonephritis or urosepsis (see sick-day guidance 5a.1.2) (Grade 1C).**

Rationale: Randomised data from major trials show the increased risk of UTIs with SGLT-2 inhibitors is small. However, these drugs are being prescribed in people who have a high risk of UTIs and effective prompt management of these infections should be undertaken.

5i. Children, pregnancy and breast feeding

5i.1 Background and evidence review

Children

There are no data available for the use of SGLT-2 inhibitors in children under 18 years of age, and therefore risks of use posed to this population are unknown.

Pregnancy

There are no human data for the use of SGLT-2 inhibitors during pregnancy. Standard practice has been to switch SGLT-2 inhibitors to insulin in the preconception period and for the duration of pregnancy, hence the lack of safety and efficacy data in this group (81).

In animal studies, at higher than recommended human doses, there have been class-wide toxicity effects highlighting potential links to ossification delays, renal maturation and tubular dilatations (74, 82-84). UK manufacturers are consistent in their advice that, due to a lack of human safety data, SGLT-2 inhibitors should not be used during pregnancy.

Breastfeeding

There are no human data for the use of SGLT-2 inhibitors whilst breastfeeding. Given the highly significant protein binding of SGLT-2 inhibitors, excretion into breast milk is unlikely to be in clinically important quantities (85, 86). Nevertheless, based on data from juvenile toxicity studies in rats whereby renal pelvic and tubular dilatations were observed through exposure via breastmilk, the manufacturers of all UK licensed SGLT-2 inhibitors are consistent in their advice that extent of excretion in human milk is unknown and therefore a risk to breastfeeding infants/newborns cannot be excluded (74, 82-84).

5i.2 Recommendations for implementation

1. **We suggest that all women of child-bearing potential are counselled, prior to conception, on the risks of SGLT-2 inhibitors during pregnancy (Grade 2D).**

2. **We suggest SGLT-2 inhibitor therapy is discontinued upon planning, suspicion or confirmation of pregnancy (Grade 2D).**

3. **We suggest SGLT-2 inhibitors are not used in women who are breastfeeding (Grade 2D).**

Rationale: There is theoretical evidence to advise against using these drugs in people either planning pregnancy, who become pregnant or who are breastfeeding. Clinical trials in the paediatric setting are suggested.

5.1 References

1. EMPA-KIDNEY Collaborative Group. Empagliflozin in Patients with Chronic Kidney Disease. New England Journal of Medicine. 2022.
2. Solomon SD, McMurray JJV, Claggett B, de Boer RA, DeMets D, Hernandez AF, et al. Dapagliflozin in Heart Failure with Mildly Reduced or Preserved Ejection Fraction. New England Journal of Medicine. 2022;387(12):1089-98.
3. Nuffield Department of Population Health Renal Studies Group and the SGLT2 inhibitor Meta-Analysis Cardio-Renal Trialists' Consortium. Impact of diabetes on the effects of sodium glucose co-transporter-2 inhibitors on kidney outcomes: collaborative meta-analysis of large placebo-controlled trials. The Lancet. 2022;400(10365):1788-801.
4. Qiu H, Novikov A, Vallon V. Ketosis and diabetic ketoacidosis in response to SGLT2 inhibitors: Basic mechanisms and therapeutic perspectives. Diabetes Metab Res Rev. 2017;33(5).
5. Pfützner A, Klonoff D, Heinemann L, Ejskjaer N, Pickup J. Euglycemic ketosis in patients with type 2 diabetes on SGLT2-inhibitor therapy-an emerging problem and solutions offered by diabetes technology. Endocrine. 2017;56(1):212-6.
6. Milder DA, Milder TY, Kam PCA. Sodium-glucose co-transporter type-2 inhibitors: pharmacology and peri-operative considerations. Anaesthesia. 2018;73(8):1008-18.
7. Perkovic V, Jardine MJ, Neal B, Bompoint S, Heerspink HJL, Charytan DM, et al. Canagliflozin and Renal Outcomes in Type 2 Diabetes and Nephropathy. The New England journal of medicine. 2019;380(24):2295-306.
8. Heerspink HJL, Stefansson BV, Correa-Rotter R, Chertow GM, Greene T, Hou FF, et al. Dapagliflozin in Patients with Chronic Kidney Disease. The New England journal of medicine. 2020;383(15):1436-46.
9. Fioretto P, Zambon A, Rossato M, Busetto L, Vettor R. SGLT2 Inhibitors and the Diabetic Kidney. Diabetes Care. 2016;39 Suppl 2:S165-71.
10. Mak RH, DeFronzo RA. Glucose and insulin metabolism in uremia. Nephron. 1992;61(4):377-82.
11. Bodmer M, Meier C, Krähenbühl S, Jick SS, Meier CR. Metformin, sulfonylureas, or other antidiabetes drugs and the risk of lactic acidosis or hypoglycemia: a nested case-control analysis. Diabetes Care. 2008;31(11):2086-91.
12. McMurray JJV, Solomon SD, Inzucchi SE, Kober L, Kosiborod MN, Martinez FA, et al. Dapagliflozin in Patients with Heart Failure and Reduced Ejection Fraction. The New England journal of medicine. 2019;381(21):1995-2008.
13. Packer M, Anker SD, Butler J, Filippatos G, Pocock SJ, Carson P, et al. Cardiovascular and Renal Outcomes with Empagliflozin in Heart Failure. The New England journal of medicine. 2020;383(15):1413-24.
14. Anker SD, Butler J, Filippatos G, Ferreira JP, Bocchi E, Böhm M, et al. Empagliflozin in Heart Failure with a Preserved Ejection Fraction. New England Journal of Medicine. 2021;385(16):1451-61.
15. Association of British Clinical Diabetologists (ABCD) position statement on the risk of diabetic ketoacidosis associated with the use of sodium-glucose cotransporter-2 inhibitors, 2016. http://www.diabetologists-abcd.org.uk/Position_Papers/ABCD_DKA_SGLT2.pdf (accessed 15th December 2022).
16. Maruyama T, Nakagawa T, Kasuga A, Murata M. Heterogeneity among patients with latent autoimmune diabetes in adults. Diabetes Metab Res Rev. 2011;27(8):971-4.
17. MHRA. SGLT2 inhibitors: updated advice on the risk of diabetic ketoacidosis, 2016. Available at

https://www.gov.uk/drug-safety-update/sglt2-inhibitors-updated-advice-on-the-risk-of-diabetic-ketoacidosis (accessed 15th December 2022).

18. FDA. Drug Safety Communication: FDA revises labels of SGLT2 inhibitors for diabetes to include warnings about too much acid in the blood and serious urinary tract infections. 2015. Available at https://www.fda.gov/drugs/drug-safety-and-availability/fda-revises-labels-sglt2-inhibitors-diabetes-include-warnings-about-too-much-acid-blood-and-serious (Accessed 15th December 2022).

19. Ibrahim M, Davies MJ, Ahmad E, Annabi FA, Eckel RH, Ba-Essa EM, et al. Recommendations for management of diabetes during Ramadan: update 2020, applying the principles of the ADA/EASD consensus. BMJ Open Diabetes Res Care. 2020;8(1).

20. van Baar MJB, van Ruiten CC, Muskiet MHA, van Bloemendaal L, RG IJ, van Raalte DH. SGLT2 Inhibitors in Combination Therapy: From Mechanisms to Clinical Considerations in Type 2 Diabetes Management. Diabetes Care. 2018;41(8):1543-56.

21. Wiviott SD, Raz I, Bonaca MP, Mosenzon O, Kato ET, Cahn A, et al. Dapagliflozin and Cardiovascular Outcomes in Type 2 Diabetes. The New England journal of medicine. 2019;380(4):347-57.

22. Zinman B, Wanner C, Lachin JM, Fitchett D, Bluhmki E, Hantel S, et al. Empagliflozin, Cardiovascular Outcomes, and Mortality in Type 2 Diabetes. The New England journal of medicine. 2015;373(22):2117-28.

23. Cannon CP, Pratley R, Dagogo-Jack S, Mancuso J, Huyck S, Masiukiewicz U, et al. Cardiovascular Outcomes with Ertugliflozin in Type 2 Diabetes. The New England journal of medicine. 2020;383(15):1425-35.

24. Bhatt DL, Szarek M, Pitt B, Cannon CP, Leiter LA, McGuire DK, et al. Sotagliflozin in Patients with Diabetes and Chronic Kidney Disease. The New England journal of medicine. 2021;384(2):129-39.

25. Filippatos G, Butler J, Farmakis D, Zannad F, Ofstad AP, Ferreira JP, et al. Empagliflozin for Heart Failure With Preserved Left Ventricular Ejection Fraction With and Without Diabetes. Circulation. 2022;146(9):676-86.

26. Tang H, Cui W, Li D, Wang T, Zhang J, Zhai S, et al. Sodium-glucose co-transporter 2 inhibitors in addition to insulin therapy for management of type 2 diabetes mellitus: A meta-analysis of randomized controlled trials. Diabetes Obes Metab. 2017;19(1):142-7.

27. Harris SB, Mequanint S, Miller K, Reichert SM, Spaic T. When Insulin Therapy Fails: The Impact of SGLT2 Inhibitors in Patients With Type 2 Diabetes. Diabetes Care. 2017;40(10):e141-e2.

28. Dandona P, Mathieu C, Phillip M, Hansen L, Griffen SC, Tschöpe D, et al. Efficacy and safety of dapagliflozin in patients with inadequately controlled type 1 diabetes (DEPICT-1): 24 week results from a multicentre, double-blind, phase 3, randomised controlled trial. The lancet Diabetes & endocrinology. 2017;5(11):864-76.

29. Mathieu C, Dandona P, Gillard P, Senior P, Hasslacher C, Araki E, et al. Efficacy and Safety of Dapagliflozin in Patients With Inadequately Controlled Type 1 Diabetes (the DEPICT-2 Study): 24-Week Results From a Randomized Controlled Trial. Diabetes Care. 2018;41(9):1938-46.

30. Gomez-Peralta F, Abreu C, Lecube A, Bellido D, Soto A, Morales C, et al. Practical Approach to Initiating SGLT2 Inhibitors in Type 2 Diabetes. Diabetes Ther. 2017;8(5):953-62.

31. Li J, Shao YH, Wang XG, Gong Y, Li C, Lu Y. Efficacy and safety of sodium-glucose cotransporter 2 inhibitors as add-on to metformin and sulfonylurea treatment for the management of type 2 diabetes: a meta-analysis. Endocr J. 2018;65(3):335-44.

32. Cherney DZI, Cooper ME, Tikkanen I, Pfarr E, Johansen OE, Woerle HJ, et al. Pooled analysis of Phase III trials indicate contrasting influences of renal function on blood pressure, body weight, and HbA1c reductions with empagliflozin. Kidney international. 2018;93(1):231-44.

33. Wu PC, Wu VC, Lin CJ, Pan CF, Chen CY, Huang TM, et al. Meglitinides increase the risk of hypoglycemia in diabetic patients with advanced chronic kidney disease: a nationwide, population-based study. Oncotarget. 2017;8(44):78086-95.

34. FDA: FDA Drug Safety Communication: FDA strengthens kidney warnings for diabetes medicines canagliflozin (Invokana, Invokamet) and dapagliflozin (Farxiga, Xigduo XR). Available at https://www.fda.gov/drugs/drug-safety-and-availability/fda-drug-safety-communication-fda-strengthens-kidney-warnings-diabetes-medicines-canagliflozin#:~:text=Safety%20Announcement,(Farxiga%2C%20Xigduo%20XR). (Accessed 15th December 2022).

35. Wanner C, Inzucchi SE, Lachin JM, Fitchett D, von Eynatten M, Mattheus M, et al. Empagliflozin and Progression of Kidney Disease in Type 2 Diabetes. The New England journal of medicine. 2016;375(4):323-34.

36. Wanner C, Inzucchi SE, Zinman B. Empagliflozin and Progression of Kidney Disease in Type 2 Diabetes. The New England journal of medicine. 2016;375(18):1801-2.

37. Neal B, Perkovic V, Mahaffey KW, de Zeeuw D, Fulcher G, Erondu N, et al. Canagliflozin and Cardiovascular and Renal Events in Type 2 Diabetes. The New England journal of medicine. 2017;377(7):644-57.

38. Wiviott SD, Raz I, Bonaca MP, Mosenzon O, Kato ET, Cahn A, et al. Dapagliflozin and Cardiovascular Outcomes in Type 2 Diabetes. The New England journal of medicine. 2019;380(4):347-57.

39. Zannad F, Ferreira JP, Pocock SJ, Zeller C, Anker SD, Butler J, et al. Cardiac and Kidney Benefits of Empagliflozin in Heart Failure Across the Spectrum of Kidney Function: Insights From EMPEROR-Reduced. Circulation. 2021;143(4):310-21.

40. Heerspink HJ, Desai M, Jardine M, Balis D, Meininger G, Perkovic V. Canagliflozin Slows Progression of Renal Function Decline Independently of Glycemic Effects. Journal of the American Society of Nephrology : JASN. 2017;28(1):368-75.

41. Mosenzon O, Wiviott SD, Cahn A, Rozenberg A, Yanuv I, Goodrich EL, et al. Effects of dapagliflozin on development and progression of kidney disease in patients with type 2 diabetes: an analysis from the DECLARE-TIMI 58 randomised trial. The lancet Diabetes & endocrinology. 2019;7(8):606-17.

42. Kraus BJ, Weir MR, Bakris GL, Mattheus M, Cherney DZI, Sattar N, et al. Characterization and implications of the initial estimated glomerular filtration rate 'dip' upon sodium-glucose cotransporter-2 inhibition with empagliflozin in the EMPA-REG OUTCOME trial. Kidney international. 2021;99(3):750-62.

43. Oshima M, Jardine MJ, Agarwal R, Bakris G, Cannon CP, Charytan DM, et al. Insights from CREDENCE trial indicate an acute drop in estimated glomerular filtration rate during treatment with canagliflozin with implications for clinical practice. Kidney international. 2021;99(4):999-1009.

44. Jongs N, Chertow GM, Greene T, McMurray JJV, Langkilde AM, Correa-Rotter R, et al. Correlates and Consequences of an Acute Change in eGFR in Response to the SGLT2 Inhibitor Dapagliflozin in Patients with CKD. Journal of the American Society of Nephrology : JASN. 2022;33(11):2094-107.

45. Bhatt DL, Szarek M, Steg PG, Cannon CP, Leiter LA, McGuire DK, et al. Sotagliflozin in Patients with Diabetes and Recent Worsening Heart Failure. The New England journal of medicine. 2021;384(2):117-28.

46. Anker SD, Butler J, Filippatos G, Khan MS, Marx N, Lam CSP, et al. Effect of Empagliflozin on Cardiovascular and Renal Outcomes in Patients With Heart Failure by Baseline Diabetes Status: Results From the EMPEROR-Reduced Trial. Circulation. 2021;143(4):337-49.

47. Jackson AM, Dewan P, Anand IS, Bělohlávek J, Bengtsson O, Boer RAd, et al. Dapagliflozin and

Diuretic Use in Patients With Heart Failure and Reduced Ejection Fraction in DAPA-HF. Circulation. 2020;142(11):1040-54.

48. Menne J, Dumann E, Haller H, Schmidt BMW. Acute kidney injury and adverse renal events in patients receiving SGLT2-inhibitors: A systematic review and meta-analysis. PLoS Med. 2019;16(12):e1002983.

49. Weir MR, Slee A, Sun T, Balis D, Oh R, de Zeeuw D, et al. Effects of canagliflozin on serum potassium in the CANagliflozin cardioVascular Assessment Study (CANVAS) Program. Clinical Kidney Journal. 2020;14(5):1396-402.

50. Shen L, Kristensen SL, Bengtsson O, Böhm M, de Boer RA, Docherty KF, et al. Dapagliflozin in HFrEF Patients Treated With Mineralocorticoid Receptor Antagonists: An Analysis of DAPA-HF. JACC Heart Fail. 2021;9(4):254-64.

51. Ferreira JP, Zannad F, Pocock SJ, Anker SD, Butler J, Filippatos G, et al. Interplay of Mineralocorticoid Receptor Antagonists and Empagliflozin in Heart Failure: EMPEROR-Reduced. J Am Coll Cardiol. 2021;77(11):1397-407.

52. Neuen BL, Oshima M, Agarwal R, Arnott C, Cherney DZ, Edwards R, et al. Sodium-Glucose Cotransporter 2 Inhibitors and Risk of Hyperkalemia in People With Type 2 Diabetes: A Meta-Analysis of Individual Participant Data From Randomized, Controlled Trials. Circulation. 2022;145(19):1460-70.

53. Matthews DR, Li Q, Perkovic V, Mahaffey KW, de Zeeuw D, Fulcher G, et al. Effects of canagliflozin on amputation risk in type 2 diabetes: the CANVAS Program. Diabetologia. 2019;62(6):926-38.

54. Arnott C, Huang Y, Neuen BL, Di Tanna GL, Cannon CP, Oh R, et al. The effect of canagliflozin on amputation risk in the CANVAS program and the CREDENCE trial. Diabetes Obes Metab. 2020;22(10):1753-66.

55. Caparrotta TM, Greenhalgh AM, Osinski K, Gifford RM, Moser S, Wild SH, et al. Sodium-Glucose Co-Transporter 2 Inhibitors (SGLT2i) Exposure and Outcomes in Type 2 Diabetes: A Systematic Review of Population-Based Observational Studies. Diabetes Ther. 2021;12(4):991-1028.

56. Zhou Z, Jardine M, Perkovic V, Matthews DR, Mahaffey KW, de Zeeuw D, et al. Canagliflozin and fracture risk in individuals with type 2 diabetes: results from the CANVAS Program. Diabetologia. 2019;62(10):1854-67.

57. Thrailkill KM, Clay Bunn R, Nyman JS, Rettiganti MR, Cockrell GE, Wahl EC, et al. SGLT2 inhibitor therapy improves blood glucose but does not prevent diabetic bone disease in diabetic DBA/2J male mice. Bone. 2016;82:101-7.

58. Blau JE, Bauman V, Conway EM, Piaggi P, Walter MF, Wright EC, et al. Canagliflozin triggers the FGF23/1,25-dihydroxyvitamin D/PTH axis in healthy volunteers in a randomized crossover study. JCI Insight. 2018;3(8).

59. de Jong MA, Petrykiv SI, Laverman GD, van Herwaarden AE, de Zeeuw D, Bakker SJL, et al. Effects of Dapagliflozin on Circulating Markers of Phosphate Homeostasis. Clin J Am Soc Nephrol. 2019;14(1):66-73.

60. Bilezikian JP, Watts NB, Usiskin K, Polidori D, Fung A, Sullivan D, et al. Evaluation of Bone Mineral Density and Bone Biomarkers in Patients With Type 2 Diabetes Treated With Canagliflozin. J Clin Endocrinol Metab. 2016;101(1):44-51.

61. Barnett K, Mercer SW, Norbury M, Watt G, Wyke S, Guthrie B. Epidemiology of multimorbidity and implications for health care, research, and medical education: a cross-sectional study. Lancet. 2012;380(9836):37-43.

62. Zemedikun DT, Gray LJ, Khunti K, Davies MJ, Dhalwani NN. Patterns of Multimorbidity in Middle-

Aged and Older Adults: An Analysis of the UK Biobank Data. Mayo Clin Proc. 2018;93(7):857-66.

63. Efficacy and Safety of Dapagliflozin According to Frailty in Heart Failure With Reduced Ejection Fraction. Annals of Internal Medicine. 2022;175(6):820-30.

64. Butt JH, Jhund PS, Belohlávek J, de Boer RA, Chiang C-E, Desai AS, et al. Efficacy and Safety of Dapagliflozin According to Frailty in Patients With Heart Failure: A Prespecified Analysis of the DELIVER Trial. Circulation. 2022;146(16):1210-24.

65. Brown E, Rajeev SP, Cuthbertson DJ, Wilding JPH. A review of the mechanism of action, metabolic profile and haemodynamic effects of sodium-glucose co-transporter-2 inhibitors. Diabetes Obes Metab. 2019;21 Suppl 2:9-18.

66. Zaccardi F, Webb DR, Htike ZZ, Youssef D, Khunti K, Davies MJ. Efficacy and safety of sodium-glucose co-transporter-2 inhibitors in type 2 diabetes mellitus: systematic review and network meta-analysis. Diabetes Obes Metab. 2016;18(8):783-94.

67. Geerlings S, Fonseca V, Castro-Diaz D, List J, Parikh S. Genital and urinary tract infections in diabetes: impact of pharmacologically-induced glucosuria. Diabetes Res Clin Pract. 2014;103(3):373-81.

68. Lega IC, Bronskill SE, Campitelli MA, Guan J, Stall NM, Lam K, et al. Sodium glucose cotransporter 2 inhibitors and risk of genital mycotic and urinary tract infection: A population-based study of older women and men with diabetes. Diabetes Obes Metab. 2019;21(11):2394-404.

69. Peleg AY, Weerarathna T, McCarthy JS, Davis TM. Common infections in diabetes: pathogenesis, management and relationship to glycaemic control. Diabetes Metab Res Rev. 2007;23(1):3-13.

70. Johnsson KM, Ptaszynska A, Schmitz B, Sugg J, Parikh SJ, List JF. Vulvovaginitis and balanitis in patients with diabetes treated with dapagliflozin. J Diabetes Complications. 2013;27(5):479-84.

71. Nyirjesy P, Sobel JD, Fung A, Mayer C, Capuano G, Ways K, et al. Genital mycotic infections with canagliflozin, a sodium glucose co-transporter 2 inhibitor, in patients with type 2 diabetes mellitus: a pooled analysis of clinical studies. Curr Med Res Opin. 2014;30(6):1109-19.

72. McGovern AP, Hogg M, Shields BM, Sattar NA, Holman RR, Pearson ER, et al. Risk factors for genital infections in people initiating SGLT2 inhibitors and their impact on discontinuation. BMJ Open Diabetes Research & Care. 2020;8(1):e001238.

73. NICE, 2017 May. Clinical Knowledge Summary – Candida Recurrent Infection. Available at: http://cks.nice.org.uk/topics/candida-female-genital/management/recurrent-infection (Accessed 15th December 2022).

74. Napp Pharmaceutical Limited, 15 Nov 2013. Summary of Product Characteristics - Invokana (Canagliflozin). [Online]. Updated: 07 Dec 2021. Available at: https://www.medicines.org.uk/emc/product/8855/smpc (Accessed 15th December 2022).

75. Sorensen MD, Krieger JN, Rivara FP, Broghammer JA, Klein MB, Mack CD, et al. Fournier's Gangrene: population based epidemiology and outcomes. J Urol. 2009;181(5):2120-6.

76. MHRA. SGLT2 inhibitors: reports of Fournier’s gangrene (necrotising fasciitis of the genitalia or perineum). 2019. Available at https://www.gov.uk/drug-safety-update/sglt2-inhibitors-reports-of-fournier-s-gangrene-necrotising-fasciitis-of-the-genitalia-or-perineum. (Accessed 15th December 2022).

77. Diabetes UK, 2021. Diabetes and Sexual Problems. [Online]. Available at: https://www.diabetes.org.uk/guide-to-diabetes/complications/sexual-problems-men (Accessed 15th December 2022).

78. de Lastours V, Foxman B. Urinary tract infection in diabetes: epidemiologic considerations. Curr Infect Dis Rep. 2014;16(1):389.

79. Fralick M, MacFadden DR. A hypothesis for why sodium glucose co-transporter 2 inhibitors have been found to cause genital infection, but not urinary tract infection. Diabetes Obes Metab. 2020;22(5):755-8.

80. Staplin N, Roddick AJ, Emberson J, Reith C, Riding A, Wonnacott A, et al. Net effects of sodium-glucose co-transporter-2 inhibition in different patient groups: a meta-analysis of large placebo-controlled randomized trials. eClinicalMedicine. 2021;41.

81. NICE, 2015. Diabetes in pregnancy: management from preconception to the postnatal period (NG3). Updated: 16 Dec 2020. Available at: https://www.nice.org.uk/guidance/ng3 (Accessed 15th December 2022).

82. AstraZeneca, 12 Nov 2012. Summary of Product Characteristics - Forxiga (Dapagliflozin). (Online). Updated: 09 Dec 2022. Available at: https://www.medicines.org.uk/emc/product/7607/smpc (Accessed 15th December 2022).

83. Boehringer Ingelheim, 22 May 2014. Summary of Product Characteristics - Jardiance (Empagliflozin). (Online). Updated 04 Nov 2022. Available at: https://www.medicines.org.uk/emc/product/5441/smpc (Accessed 15th December 2022).

84. Merck Sharp & Dohme (UK) Limited, 01 Jan 2021. Summary of Product Characteristics – Steglatro (Ertugliflozin). (Online) Updated 13 April 2022. Available at: https://www.medicines.org.uk/emc/product/10099/smpc (Accessed 15th December 2022).

85. National Library of Medicine (US), 2018. Drugs and Lactation Database (LactMed). (Online). Updated: 03 Dec 2018. Available at: https://www.ncbi.nlm.nih.gov/books/NBK501922/ (Accessed 15th December 2022).

86. Briggs, G., Freeman, R., Towers, C. & Forinash, A. Drugs in Pregnancy and Lactation. 11th ed. 2017. Lippincott Williams and Wilkins, pp. 200-201, 485-486.

Section 6: Lay summaries and patient information leaflets

This section is designed for patients and carers and for healthcare workers who wish to obtain a lay summary of this guideline. The section contains a one-page executive lay summary followed by a full lay guideline summary. The section also contains examples of patient information leaflets that can be used when initiating sodium-glucose co-transporter-2 (SGLT-2) inhibitors for people with diabetes and also for those without diabetes. It is anticipated that by providing a clear description of the contents of this guideline there will be greater understanding of the benefits and the risks of using SGLT-2 inhibitors for people with chronic kidney disease (CKD).

6.1 Lay executive summary

CKD is a significant medical problem affecting anything between 6 to 11% of the UK adult population. It is a disorder in which the kidneys are damaged causing a reduction in their ability to clean the blood. If the CKD is progressive (which means it slowly deteriorates over time) the person suffering this disorder is at risk of kidney failure and the need to start a treatment to replace their kidney function in the form of either dialysis or kidney transplant. Importantly, as well as the issue of suffering kidney failure, people with CKD have a much higher chance of suffering cardiovascular diseases in the form of heart attacks, heart failure, strokes and damage to the blood supply to the legs and feet.

The treatment of CKD has been centred around control of blood pressure, the reduction in other cardiovascular risk factors (for example, stopping smoking and managing cholesterol) and the use of a group of medications known as angiotensin-converting enzyme inhibitors (or ACE inhibitors - the names of which usually end with "-pril") or angiotensin-II receptor blockers (ARBs - the names of which usually end with "-sartan"). However, even with the use of these interventions, people with CKD still suffer considerable harm related to their underlying kidney disorder.

The SGLT-2 inhibitors is the name given to a group of medications that were initially developed to treat people with diabetes by providing them with better control of their blood glucose (sugar) and can be recognised by the drug name ending in "-gliflozin". As part of the developmental program for this group of medications, each of the individual SGLT-2 inhibitors underwent a large study to ensure that, not only are they effective in helping people with diabetes reduce blood glucose, but that they also did not cause any increased risk of cardiovascular disease. The findings from these large studies, which have been undertaken and reported over the last six years, has had a significant impact on the care of people with heart disease and CKD. This is because all of these medications have been shown to have unexpected beneficial effects in relation to reducing the rate of progression of CKD and reducing the risk of heart failure complications. In relation to CKD, this benefit was seen even though the study participants were already being treated with current best practice and even in this circumstance the SGLT-2 inhibitor provided very significant additional benefit.

As a result of these studies and the further specific studies directly examining the effects of SGLT-2 inhibitors in people with kidney disease and heart failure, it is recognised that these medications need to be offered to people who are likely to experience benefit. It is the purpose of this guideline to review the evidence related to the benefits and the potential adverse risks of SGLT-2 inhibitors, in order to provide clear recommendations as to which people with CKD are most likely to benefit from this medication, and in order to encourage the healthcare system to ensure that those individuals receive this beneficial treatment as

speedily as possible.

All medical interventions can have side effects and for SGLT-2 inhibitors, we are clear as to the nature of the side effects and their frequency. These medications have a low risk of side effects but it is also possible to reduce that risk further by making careful choices about which individuals receive the medications and by informing people prescribed SGLT-2 inhibitors how to take specific actions to reduce the risk of coming to any harm should they suffer from any side effects. People with CKD should only be offered an SGLT-2 inhibitor if the benefits significantly outweigh the risks. Therefore, this guideline also provides information around the side effects of SGLT-2 inhibitors and how those side-effect risks can be reduced.

6.2 Full summary of guideline

6.2.1 Introduction

Between 6 to 11% of the adult population of the United Kingdom is thought to have CKD. These individuals are not just at risk of progressive decline of kidney function resulting in them suffering symptoms related to poor kidney function and requiring them to be considered for end-stage kidney disease treatment (dialysis or transplant) but they also have a greatly increased risk of cardiovascular disease (heart attacks, heart failure, strokes and narrowing of the arteries to the legs termed peripheral vascular disease). This represents a significant burden for both the health economy of the United Kingdom and, more importantly, for the individuals themselves.

Treatment for CKD aims to halt or slow down progression of declining kidney function and reduce cardiovascular risk. These treatments have up until now been centred on a group of medications known as the inhibitors of the renin angiotensin system, which is a system that has a controlling influence on blood pressure and fluid status. In addition, control of blood pressure, blood glucose and blood lipids (or fats) remain key to reducing progression of CKD and the associated poor cardiovascular outcome. However, these treatments are only partially effective and there has been a pressing need to identify new treatments to help the large number of people with CKD avoid the requirement for dialysis or transplant, or from suffering cardiovascular harm.

SGLT-2 inhibitors are medications that were initially developed as a treatment for diabetes because they effectively reduce blood glucose (sugar). New research has found that these medications provide significant benefit to people with CKD both in terms of reducing decline in kidney function and also reducing the poor cardiovascular outcomes people with CKD suffer.

The purpose of this guideline document is to produce practical advice for clinicians caring for people with kidney disease in relation to when and how to use SGLT-2 inhibitors.

It is the purpose of this section to provide lay individuals a greater understanding of the nature of SGLT-2 inhibitors, the benefits they offer, in which individuals they are most likely to be effective and to obtain a greater appreciation of the risks of these medications and how these risks can be reduced.

6.2.2 SGLT-2 inhibitors: what are they?

The kidneys function to clean the blood and control the concentration of many constituents of the blood. They do this by "filtering" the blood through individual filtering units (called glomeruli, of which there are approximately 1 million within each kidney in healthy adults) and thereafter the filtered fluid or "filtrate" passes into small pipes or "tubules" within the kidneys where its content is adjusted. The filtrate eventually becomes the urine, which is passed from the kidneys to the bladder and removed when we pass urine.

Glucose is freely filtered from the blood but normally all of this filtered glucose is returned back into the blood within the early part of the tubule of the kidneys called the proximal tubule. This return is undertaken by particular transporter proteins called SGLT-2 which sit in the wall of the proximal tubule. Every person has a maximal amount of glucose that their kidney can reabsorb and in individuals who have high amounts of filtered glucose (typically in people with diabetes) the SGLT-2 co-transporters become flooded and residual glucose is lost in the urine (and in fact it is the resulting sweet urine which gives diabetes mellitus its name).

SGLT-2 inhibitors are medicines that block the activity of the SGLT-2 co-transporter and by blocking this protein's actions, SGLT-2 inhibitors cause a loss of glucose into the urine and is the reason these medicines have been developed as a treatment to help reduce blood glucose in people with diabetes.

In addition to SGLT-2 co-transporters there are, within the body, a group of related proteins called SGLT-1 co-transporters. These proteins are found more predominantly in the gut where they are involved in the uptake of glucose from the food into the bloodstream. They are also found, albeit to a much lesser extent, in the kidney tubules where they make only a very minor contribution to total glucose reuptake in the kidneys.

6.2.3 Benefits of SGLT-2 inhibitors

All new medications being introduced to treat diabetes are required to demonstrate that not only do they improve blood glucose control but they do not have an adverse effect on cardiovascular outcomes in people with diabetes. This is because there has been an example of a previous medication which provided significant benefit in relation to reducing blood glucose but at the same time was associated with an increased risk of heart attacks. Therefore, all new medications being introduced to treat diabetes are required to undergo what are termed cardiovascular outcome trials.

All the major SGLT-2 inhibitor medications have reported on their cardiovascular outcomes and the findings from these studies has provided significant information in relation to additional benefits that these medications provide.

It was already known that these medications have benefits over and above their glucose reducing effect, which included a reduction in weight of around 2 to 4 kg and a small reduction in blood pressure. These additional effects were believed to result from the loss of salt in the urine (called the diuretic effect) and the loss of calories in the form of glucose within the urine. In addition to these effects, the cardiovascular outcome studies identified significant benefits in relation to reducing cardiovascular harm, most particularly in relation to reducing admission to hospital with heart failure and in reducing the progression of CKD.

6.2.4 How this guideline was developed

This guideline has been developed by a writing group containing a broad range of healthcare clinicians with experience in kidney disease, diabetes and primary care who have worked together to review the evidence for the use of SGLT-2 inhibitors in people with kidney disease. In addition writing group has also included people with kidney disease to provide the patients' perspective. As a group, they have followed good practice in relation to reviewing the evidence and using that evidence to provide recommendations for the use of SGLT-2 inhibitors in people with CKD.

In generating the recommendations, the guideline writing committee gave the greatest priority to the results of trials that were most effective at discriminating both beneficial and adverse effects of SGLT-2 inhibitors. These were trials comparing people who were allocated to take SGLT-2 inhibitors at random (like a toss of a coin) to those who were allocated to take a dummy pill (known as the placebo group) and containing large (greater than 1000) numbers of participants.

From this evidence base, the guideline writing committee has developed summaries of evidence and proposed draft recommendations which were discussed at a consensus meeting of all members before final recommendations were made.

When making recommendations, the evidence that supported each recommendation was graded according to the UK Kidney Association's recommended grading system, which defines the level of evidence and the quality of evidence for each recommendation. Broadly, a grade 1 recommendation is a strong recommendation while a grade 2 recommendation is a weaker one. In addition, there is a letter designating the quality of the evidence that supports that recommendation.

Where the evidence to support a recommendation is strong (grade 1) we use the term "recommend" and where it is weaker (grade 2) we use the term "suggest".

We have also subdivided our recommendations into the following categories:

a) Recommendations for Use which defines who should be offered SGLT-2 inhibitors
b) Recommendations for Implementation which defines how SGLT-2 inhibitors should be used
c) Recommendations for Clinical Research which defines where there is ongoing clinical uncertainty
d) Recommendations for Audit which defines how to demonstrate effective implementation of grade 1 Recommendations

6.2.5 Benefits of SGLT-2 inhibitors: cardiovascular benefits

The cardiovascular outcome trials identified the fact that SGLT-2 inhibitors had a small effect on reducing the incidence of heart attack which varied between the individual SGLT-2 inhibitors. However, all the trials demonstrated a significant reduction in heart failure hospitalizations that pointed to a significant benefit in people with heart failure. This benefit has now been confirmed in studies that have specifically looked at the use of SGLT-2 inhibitors in people with heart failure including people with and without diabetes.

6.2.6 Benefits of SGLT-2 inhibitors: kidney protection

An unexpected and consistent finding from the cardiovascular outcome trials was that individuals treated with SGLT-2 inhibitors as opposed to placebo or dummy medications had improved kidney outcome in terms of a reduction in decline of kidney function, the need to commence treatment for end-stage kidney failure and death due to kidney causes.

These findings have been tested in further studies called CREDENCE, DAPA-CKD and EMPA KIDNEY which have specifically looked at kidney outcomes in people with evidence of chronic kidney disease and for DAPA-CKD and EMPA KIDNEY this kidney beneficial effect was seen in both people with and without diabetes.

6.2.7 Separation of glucose from cardiac and kidney benefit

The benefits of SGLT-2 inhibitors in relation to the improvement in blood glucose control is known to be related to the glomerular filtration rate (GFR). This is a measure of global kidney filtering function and is the measure that progressively reduces as kidney function declines in CKD. A normal GFR should be approximately 90 mL/min/1.73m^2, but there is a progressive normal decline with ageing. Excessive decline down to single figures (i.e. less than 10 mL/min/1.73m^2) is usually an indicator of the need to commence kidney failure treatment.

As the GFR declines so also does the ability of SGLT-2 inhibitors to improve glucose control in diabetes, such that by the time the GFR has reduced to 45 mL/min/1.73m^2, the glucose reducing effect of SGLT-2 inhibitors virtually disappears in people with diabetes treated with these agents. It is for this reason that these medications have not been recommended for use as a treatment for diabetes in people whose kidney function is already deranged because they have CKD.

In the kidney specific outcome studies and studies looking at SGLT-2 inhibitors in people with heart failure, people with kidney function down to 20 mL/min/1.73m^2 have now been enrolled. Indeed there is evidence from analysis of all these studies to suggest that the heart and kidney protection continues even at the lowest levels of kidney function perhaps down to the level at which dialysis or transplantation is required.

This separation of the glucose and cardiac and kidney protective effect was exemplified further by the fact that, in the DAPA-CKD, EMPA KIDNEY and the heart failure specific studies, SGLT-2 inhibitors were beneficial even in people without diabetes.

6.2.8 Identifying individuals where there is benefit in prescribing SGLT-2 inhibitors

SGLT-2 inhibitors can only be recommended as a treatment if there is sufficient evidence to support that use and this is usually obtained from randomized controlled trials where participants in the trials are split randomly into those given the treatment and those given a dummy treatment. There are now a significant number of trials with SGLT-2 inhibitors that were designed first of all to assess the cardiovascular safety of these medications (cardiovascular outcome trials) as well as trials looking at these medications in people with heart failure and in people with kidney disease.

As these results have emerged, the licences (which determines how the treatment can be prescribed in the UK) for a number of SGLT-2 inhibitors have been broadened to allow the use of that specific SGLT-2 inhibitor

for the purpose not just of glucose control, but also as protection against cardiovascular or kidney disease.

In assessing the evidence for benefit, one also needs to be clear about the outcomes that are being measured. For kidney disease there are many different potential outcomes that have been used in previous studies; however, not all of these clearly define outcomes that truly benefit people with CKD. Therefore, determination of benefit of SGLT-2 inhibitors for the purpose of generating recommendations in this guideline is based on real evidence for the reduction in progression of kidney disease which has been measured by the need to commence any form of kidney replacement therapy, death caused by kidney disease and significant reduction in decline in kidney function.

In the major cardiovascular outcome trials of all four of the SGLT-2 inhibitors, kidney effects were monitored and in all of these studies there was a decline in progression of kidney disease, need to commence kidney replacement therapy or death due to kidney disease. However, these effects were not the primary purpose of the studies and therefore there is a risk that the findings could have been identified by chance. Therefore, further studies have been undertaken that have looked primarily at the effect of SGLT-2 inhibitors in people with kidney disease. These studies include:

1) CREDENCE which tested the SGLT-2 inhibitor canagliflozin in people with type 2 diabetes and evidence of diabetic kidney disease in the form of some reduction in GFR and the presence of protein in the urine
2) DAPA-CKD which tested the SGLT-2 inhibitor dapagliflozin in people with evidence of kidney disease in the form of a reduction in GFR and presence of protein in the urine but, importantly, not just in people with diabetes as a cause for their kidney disease.
3) EMPA KIDNEY which tested the SGLT-2 inhibitor empagliflozin in a wide range of people with evidence of kidney disease at risk of progression, in the form of reduction in GFR both with and without the presence of protein in the urine and in both people with and without diabetes.

CREDENCE, DAPA-CKD and EMPA KIDNEY have confirmed the safety findings from the cardiovascular outcome trials and demonstrated significant kidney benefits of SGLT-2 inhibitors in people with CKD. These benefits included clear reduction in progression of diabetic kidney disease by approximately 30 to 50%. Overall, they also reduced occurrences of heart attacks and deteriorations of heart failure in people with CKD.

Because of the findings from review of the studies this guideline recommends the initiation of SGLT-2 inhibitors in people both with and without diabetes who have evidence of increased albumin in the urine (at a level of greater than 25 mg/mmol) down to an eGFR of 20 or if they have an eGFR between 20 and 45 mL/min/1.73m^2 even in the absence of albuminuria. In addition, the guideline recommends initiation of SGLT-2 inhibitors in people with CKD and a history of heart failure.

Once initiated, this guideline recommends that the SGLT-2 inhibitor can be continued until the individual reaches end-stage kidney disease.

Practically, the SGLT-2 inhibitor that would be utilised would be dependent on the current licence for that individual SGLT-2 inhibitor and whether primary care (general practitioners) are able to prescribe that specific medicine.

6.2.9 Groups where there is uncertainty

As described, it is only possible to recommend SGLT-2 inhibitor treatment for cardiac and kidney benefit in people who were represented in the studies of these medications, and in whom there is clear evidence of benefit.

There are a number of groups of people in whom it is not yet possible to be specific about the cardiac and kidney benefits of SGLT-2 inhibitors.

Currently, this includes people who have CKD caused by the genetically inherited condition called adult polycystic kidney disease. Clinical trials to date that have studied SGLT-2 inhibitors have not included people with adult polycystic kidney disease, meaning that there is not enough evidence to recommend this medication class.

Type 1 Diabetes Mellitus

There are two main types of diabetes which include type 1 diabetes (which occurs more usually at a younger age and in which there is loss of the ability to produce insulin) and the much more common type 2 diabetes. Type 2 diabetes usually occurs in the older age group and is related to the ability of insulin to be effective, with the most common reason for this being association with obesity.

Whilst there have been some studies of SGLT-2 inhibitors in type 1 diabetes, these have not been sufficient to make a clear recommendation on the use of SGLT-2 inhibitors in people with type 1 diabetes. There may be benefits in people with type 1 diabetes, but there is a risk of a condition called diabetic ketoacidosis which is particularly high in people with type 1 diabetes. This means any potential benefits of SGLT-2 inhibitors may be finely balanced with potential risk of harm (more details below).

People with functioning kidney transplants

Whilst it would be appealing to assume that the benefits that SGLT-2 inhibitors provide in relation to reduction in progression of CKD and protection against cardiovascular disease is present in all people with abnormal kidney function, kidney transplant recipients were not included in any of the trials. There is currently insufficient evidence at this time to recommend that people with a kidney transplant should be included in those initiated on SGLT-2 inhibitors. Whilst there are ongoing studies in this area, SGLT-2 inhibitors should only be offered to people with a kidney transplant after careful consideration and discussion between the kidney transplant team and the diabetes teams and with clear discussion undertaken with the individual with the kidney transplant.

6.2.10 Side effects of SGLT-2 inhibitors and how to avoid them

Every medication has potential to result in adverse events and it is important that these are appropriately understood in order for people to be advised appropriately about the risks of the medication and how it is possible for them to take steps to reduce any harm that could occur from taking a medication.

All people who are being prescribed new medications need information given to them that allows them to

make an informed choice as to whether they wish to commence the treatment. This information needs to include a balance between both the risks of the medication and the potential benefit to them as an individual. Furthermore, the information needs to include advice on actions that would reduce the chance of harm coming to them by taking a particular medication.

SGLT-2 inhibitors have been found to have a number of adverse effects that people need to be informed about prior to initiation of this therapy. It is important to also appreciate that the likelihood of suffering an adverse effect may depend on the individual as well as the medication, and this would include factors such as whether they have diabetes, their age and their frailty.

6.2.11 Diabetes specific: diabetic ketoacidosis

SGLT-2 inhibition has an effect on the breakdown of carbohydrates and fats, which results in an excess of a group of substances known as ketones. These molecules are not dangerous of themselves, however, if the level of these ketones increases this can result in a dangerous situation in which the blood becomes very acidic which is termed ketoacidosis.

Ketoacidosis is a dangerous complication, seen most particularly in people with type 1 diabetes, but can occur (although rarely) in people with type 2 diabetes and there is evidence that SGLT-2 inhibitors increase the risk of this happening. Diabetic ketoacidosis usually occurs in conjunction with high levels of glucose, however, when it occurs in a person taking an SGLT- 2 inhibitor, the excess glucose can be lost in the urine and this dangerous complication can occur in conjunction with normal levels of glucose which has the potential to confuse both the person with diabetes and the healthcare worker assessing the person.

The risk of diabetic ketoacidosis increases in the presence of infection or if the individual becomes dehydrated because of diarrhoea, vomiting or fasting. It also occurs in situations where there is not enough insulin, such as in people with type 2 diabetes who have low levels of their own insulin production and in situations when people who are treated with insulin have their insulin reduced, or even stopped.

A further factor that can be associated with an increased risk of diabetic ketoacidosis in people prescribed SGLT-2 inhibitors is the use of specific diets which are termed “very low carbohydrate” or ”ketogenic” diets and which increase the blood levels of ketones (such as the Atkins diet).

Because of their greater risk of suffering diabetic ketoacidosis, people with type 1 diabetes should only be commenced on SGLT-2 inhibitors under strict direction of the diabetes team, and may be offered lower doses.

It is also recognised that there are a group of people with type 2 diabetes who are at greater risk of diabetic ketoacidosis because they have lower levels of insulin. One can identify this group of individuals by features associated with low insulin levels, of which the most important are the rapid requirement for insulin treatment following diagnosis (within one year) and the presence of type 2 diabetes and low body weight. It is recommended that for these people with type 2 diabetes who are at greater risk of diabetic ketoacidosis, SGLT-2 inhibitors should only be initiated after discussion with the diabetes team.

If an individual prescribed SGLT-2 inhibitors develops diabetic ketoacidosis, it is recommended that the SGLT-2 inhibitor should be stopped and that individual should be reviewed by a member of their clinical team to determine whether treatment could be re-initiated in the future. That decision will be dependent on

the analysis of the reasons why the diabetic ketoacidosis occurred, and whether with changes to treatment, or better advice on management, the risk of future diabetic ketoacidosis can be significantly reduced. That decision should be discussed between the clinical team and that individual themselves.

Because of the risk of diabetic ketoacidosis, people started on SGLT-2 inhibitors need to be told about diabetic ketoacidosis and in particular the signs and symptoms of this disorder and the importance of seeking immediate medical advice if those symptoms develop.

Perhaps one of the most effective ways of preventing the occurrence of diabetic ketoacidosis is to use what is termed “sick day guidance”. This is where a medication, which ordinarily has a significant benefit to the individual, can cause an adverse effect if it is continued when they become unwell with features of a fever or inability to maintain their fluid status (such as vomiting or diarrhoea). In this instance, it is important to miss out the specific medication (such as the SGLT-2 inhibitors) if they become unwell or if they are hospitalized. The medication should be resumed once the illness has passed or the person has been discharged from hospital. If a person stops their SGLT-2 inhibitor because of ill health and there is no improvement beyond a period of 48 hours they should seek medical attention. It is for this reason that all people prescribed SGLT-2 inhibitors should be taught about sick day guidance to be used if they become unwell and that this advice should be reiterated at every medication review.

Because of the relationship of diabetic ketoacidosis to specific diets and to situations where the individual is taking a reduced fluid intake such as when fasting, the individual should be advised not to follow these particular diets when on an SGLT-2 inhibitor and be given specific advice if they do choose to fast. This might include missing out the SGLT-2 inhibitors on fast days or testing for the presence of ketones if they become unwell.

6.2.12 Diabetes specific: Low blood glucose or hypoglycaemia

SGLT-2 inhibitors have beneficial effects in improving blood glucose control, which is dependent on good kidney function. Hypoglycaemia is a situation where the person’s blood glucose drops to a low level causing harm that varies from mild symptoms to profoundly significant symptoms including coma. Furthermore, severe hypoglycaemia can be associated with long-term damage affecting both the cardiovascular system and the brain. People who have suffered episodes of hypoglycaemia may have limitations on their ability to drive or to undertake certain occupational activities. Therefore, hypoglycaemia is a complication of diabetes that needs to be avoided and certainly minimised.

Whilst SGLT-2 inhibitors on their own do not produce hypoglycaemia, this can occur if they are used with a diabetes agent that has such a risk, such as insulin or the group of diabetes medications that work by directly stimulating insulin release (these are termed insulin secretagogues and include gliclazide, glimeprimide, glipizide, repaglinide etc.).

In people treated for their diabetes with insulin or insulin secretagogues, consideration needs to be given as to whether to reduce the current diabetes treatment when commencing an SGLT-2 inhibitor. The decision on whether to make this change should be discussed by the clinician prescribing the SGLT-2 inhibitor and the individual who is receiving it. This decision should be based on a number of factors and should include an assessment of the underlying blood glucose control of that individual. For example, someone who has poor

blood glucose control as assessed by their HbA1c (this is the blood test that is used to assess average blood glucose control over the preceding 8 to 12 weeks), may benefit from the addition of the SGLT-2 inhibitor without the need for any reduction in their insulin or insulin secretagogues dose. Conversely, an individual with better controlled HbA1c may need a reduction in their insulin or insulin secretagogue dose. As has been described, if the kidney function is poor (GFR <45 mL/min/1.73m^2), then the blood glucose reducing effect of the SGLT-2 inhibitor will be significantly reduced and there may therefore be no reason to make that reduction in insulin or insulin secretagogue.

It is therefore recommended that where the person being prescribed the SGLT-2 inhibitor has diabetes and is on insulin or an insulin secretagogue, the dose of these medications should be reviewed together with their prescribing clinician. If they are on an insulin secretagogue and their HbA1c is <58 mmol/mol and their eGFR >45 mL/min/1.73m^2, the insulin secretagogue dose should be reduced by approximately 50% and the insulin by approximately 20% in order to avoid the risk of hypoglycaemia.

Where the person with type 2 diabetes is being prescribed an SGLT-2 inhibitor and they are not on an insulin secretagogue or insulin then there is no need to adjust any other diabetes medications.

There is no evidence to suggest that there is a risk of low blood glucose in people prescribed SGLT-2 inhibitors who do not have type 2 diabetes.

6.2.13 Acute kidney injury

Because of their mechanism of action, there can be an initial small, reversible reduction in kidney function over the first few weeks after commencing an SGLT-2 inhibitor. This results from changes to the blood flow in the kidneys with a small reduction in the blood flow going through the filtering units (glomeruli). This effect is believed to be protective rather than an indicator of harm. In all the major cardiovascular outcome trials, there was an initial small fall in GFR (of the order of 2 to 5% but with wide variation within the individual studies) and thereafter stabilisation of the GFR in participants given an SGLT-2 inhibitor. In the placebo group, there was a slow progressive decline in GFR such that the kidney function of the placebo-treated participants was significantly lower than the SGLT-2 inhibitor treated participants at the end of the study.

Further reassurance of the lack of harm and indeed possible beneficial effect of SGLT-2 inhibitors in relation to acute kidney injury is the fact that in all the major cardiovascular outcome trials, kidney studies and heart failure studies, the incidence of acute kidney injury (this is where there is a significant drop in kidney function which is usually reversible) was always greater in the placebo-treated groups compared to the SGLT-2 inhibitor-treated groups. It may even be that SGLT-2 inhibitors protect against acute kidney injury.

It is important that this effect is properly understood so that people who are prescribed SGLT- 2 inhibitors and who may gain significant benefit from these medications do not have them stopped because of changes in GFR if measured in the period following prescription of these agents.

It is therefore recommended that when a person is commenced on an SGLT-2 inhibitor, there is no need to routinely check the kidney function in the early period following initiation of treatment, but this should be undertaken at the next usual review appointment for that individual. Furthermore, it is also recommended that if kidney function is assessed for another reason within the first few weeks following initiation of an SGLT-2 inhibitor, the result needs to be interpreted carefully and unwarranted discontinuation of treatment

should not occur.

6.2.14 Dehydration issues

SGLT-2 inhibitors do cause an increase in urine output (diuretic effect), both because of the fact they result in loss of sodium (salt) in the urine but also because the extra glucose in the urine pulls in water to the urine. As a result, people started on these agents can experience an increased frequency of passing urine which is most prominent in the first few weeks after commencing the medication.

For the vast majority of people this is nothing more than a minor issue which reduces over time but there are occasional individuals who are at risk of dehydration when starting an SGLT-2 inhibitor. It is difficult to be certain how often this occurs because in all the trials that have been undertaken with SGLT-2 inhibitors there is variation in how this side effect is reported. In studies where there has been an increased frequency of dehydration issues reported, the increase has been small, affecting an extra 1-2 people out of 100. It is, however, most probable that it is only really relevant to individuals who are already taking "water-tablets" (diuretics) and particularly if they are taking these at high doses.

It is therefore recommended that if a person who is prescribed an SGLT-2 inhibitor is already on either diuretic or blood pressure medication, the prescriber should consider whether an early review to assess for dehydration or low blood pressure is undertaken and if either of these are identified, a reduction in the dose of either of these additional diuretic or blood pressure medications is required. Furthermore, if a person being commenced on an SGLT-2 inhibitor is already on a diuretic, they should be counselled on symptoms of dehydration or low blood pressure so they can seek medical attention if they develop such symptoms.

In addition it is important again to remind people being treated with SGLT-2 inhibitors to use good sick day guidance and omit SGLT-2 inhibitors if they are unwell.

6.2.15 Peripheral vascular disease

People with CKD, and especially those with diabetes, are at an increased risk of suffering disorders of their cardiovascular system which includes disorders that affect the blood supply to their legs, termed “peripheral vascular disease”.

In all the cardiovascular outcome trials for SGLT-2 inhibitors, issues relating to peripheral vascular disease were monitored and recorded. In one of the larger trials in people with type 2 diabetes (called CANVAS), the medication canagliflozin significantly increased the number of amputations undertaken in the group allocated to an SGLT-2 inhibitor compared to those allocated to placebo. This increased incidence of amputation (mainly of toes) was not identified in any of the other cardiovascular outcome trials which included large numbers of participants who were broadly similar to those recruited to CANVAS. Furthermore, in a study that looked at people with kidney disease and diabetes known as CREDENCE, and which also used canagliflozin, there was no increased risk of amputation despite the fact that this group of participants were at high risk of vascular disease. Because of these findings a warning has been placed by the regulators in relation to using canagliflozin in people at high risk of amputation.

It is important to appreciate that any increased risk of amputation, if it exists, is significantly less than the benefits canagliflozin treatment would have in those individuals. It is for this reason that we recommend

that when a person has evidence of active foot disease caused by problems with the blood supply, no SGLT-2 inhibitor should be started and if they have already been commenced on one of these agents then this should be withheld. However, because of the benefits that are likely to occur in these individuals it is also a recommendation that once the active foot disease has been effectively treated, discussion should occur with the individual in relation to the risks and benefits so that a decision can be made whether the resumption of an SGLT-2 inhibitor is in the person's interests and in accordance with their wishes. Similarly, a discussion of this nature should be held prior to commencing an SGLT-2 inhibitor in a person with high risk of vascular disease.

We define active peripheral vascular disease as the presence of foot ulcers, what is termed intermittent claudication (which is when there is pain over the back of the calves associated with walking) or where there is other evidence of reduced blood supply to the legs and feet.

In order to reduce the chance of any harm occurring to a person prescribed SGLT-2 inhibitors, we recommend that all such individuals (and most particularly individuals with diabetes) should be given advice on good foot care and the importance of seeking early attention should any problems develop.

6.2.16 Bone Health

Within the CANVAS trial there was a small increase in fractures in the group allocated to canagliflozin. However, this has not been identified in any other cardiovascular outcome trial using an SGLT-2 inhibitor, nor in other studies using canagliflozin, such as CREDENCE. Therefore it is uncertain as to whether this is a real effect or just a result of the play of chance in that trial. People with CKD are at a greater risk of suffering from bone disorders and good practice already recommends that all these individuals should have measurements of good bone health monitored on a regular basis. It is our recommendation that any person with kidney disease who is commenced on an SGLT-2 inhibitor should receive the good care in relation to their bone health as recommended by national guidelines.

6.2.17 Fungal genital infections (i.e. thrush)

Because the use of SGLT-2 inhibitors results in an increase in sugar in the urine, this can provide an environment in which certain fungal infections thrive and therefore an increase in these infections locally. These infections are termed genital infections and are typified by thrush, which is not an uncommon infection, particularly in women and particularly in people with diabetes.

All SGLT-2 inhibitors increase the risk of thrush. This is not a dangerous complication, but can cause irritation and needs to be managed appropriately. Symptoms involve irritation or itchiness and redness or inflammation in the genital area (vulva-vagina for women and tip of the penis in men (inflammation of which is known as balanitis)). It is most likely that the risk is greater if the person being treated has type 2 diabetes.

Before anyone starts an SGLT-2 inhibitor they need to be counselled on the risk of fungal genital infections such as thrush and on simple measures that they can implement to maintain good genital hygiene and thereby reduce the risk of thrush. They also need to be counselled on the symptoms of genital infections and how to seek help which can often be attained simply by attending a local pharmacist to obtain an antifungal cream.

This advice is most particularly important for men as they may not have as much knowledge of thrush as women.

If genital infections such as thrush occur while a person is using an SGLT-2 inhibitor they should be treated, but they do not necessarily need to stop the SGLT-2 inhibitor. Usually once treated the genital infection does not recur but if the individual suffers recurrent infections their GP can give them treatment to prevent infections which is either a cream or a tablet they take intermittently.

6.2.18 Fournier's gangrene

Fournier's gangrene is a serious infection caused by bacteria infecting the skin in the genital area. It is exceedingly rare but theoretically it could be increased if the individual has an increased glucose concentration in the urine and it is recognised to occur, albeit very rarely, in people with diabetes. Occasional reports have been made of people suffering this complication while being treated with an SGLT-2 inhibitor and because of this the regulators have placed a warning of this complication for people using SGLT-2 inhibitors. It should be pointed out that no increased cases of Fournier's gangrene were identified in any of the trials undertaken, which included large numbers of participants. However, even though we do not know whether SGLT-2 inhibitors truly increase the risk of this disorder, we recommend that all people started on an SGLT-2 inhibitor are counselled on the symptoms of Fournier's gangrene and advised to stop their SGLT-2 inhibitor and seek urgent medical attention if they develop such symptoms. The main symptom to be aware of is severe pain on pressing the skin over the groin area.

6.2.19 Urinary infections

SGLT-2 inhibitors produce an increased amount of glucose in the urine, but despite this there is only a very small increased risk of urine infections. This may be because bacteria do not thrive in environments with high glucose or because there is an increase in urine output which works to help "flush away" bacteria in the urinary tract. There is a need to be alert to this, but it is unlikely that a person with a normal bladder and bladder function will have an increased risk of urine infection when using SGLT-2 inhibitors.

6.2.20 Special populations: Children, pregnancy and breastfeeding

There is little experience in using SGLT-2 inhibitors in children and therefore it is not recommended that these medications are used in people under the age of 18.

There is some theoretical evidence that SGLT-2 inhibitors, at much higher doses and in animal models, can potentially result in malformations and therefore the use of these medications in pregnancy should be avoided. If a person who is being treated with SGLT-2 inhibitors wishes to consider pregnancy they should discuss this with their doctor and plan the pregnancy in advance with a plan to stop the SGLT-2 inhibitor prior to becoming pregnant, or if an unplanned pregnancy occurs.

Whilst there is no real evidence to suggest that SGLT-2 inhibitors is passed in the breastmilk in important quantities, it is not recommended that these agents should be used when breastfeeding.

6.3 **Example patient information sheet for a person being initiated on an SGLT-2 inhibitor who also has diabetes**

Getting the most from your sodium glucose co-transporter-2 inhibitors (SGLT-2i)

(For people with diabetes)

Information for patients, relatives and carers

Introduction

This leaflet has been designed to give you information about sodium glucose co-transporter-2 inhibitors (SGLT-2 inhibitors) and answers some of the questions that you or those who care for you may have about these medicines. It is not meant to replace the discussion between you and your medical team but aims to help you understand more about what is discussed. If you have any questions about the information below, please contact the person who provided you with this leaflet.

What are SGLT-2 inhibitors and who benefits from using them?

You are being treated with one of the SGLT-2 inhibitor medicines, sometimes known as “gliflozins" or “flozins”. These include, canagliflozin (Invokana), dapagliflozin (Forxiga), empagliflozin (Jardiance) and ertugliflozin (Steglatro).

These medicines lower blood glucose (sugar) by increasing the amount of glucose in the urine, which is why these medicines are used for people who have diabetes. They have added benefits that include protecting the kidneys and heart, slowing the decline in kidney function and reducing the risk of heart failure and heart attacks in individuals at most risk.

Are there any side effects?

Common:

- **Hypoglycaemia (low blood glucose)** – this usually only occurs if SGLT-2 inhibitors are used in combination with certain other diabetes medicines and your doctor may therefore need to reduce other diabetes medicines. However, never stop insulin altogether if you are already on this.

- **Dehydration** – Dehydration is when your body does not have as much water as it needs. These medicines increase the amount of urine that you pass so may cause dehydration. To prevent dehydration, drink fluids when you feel any dehydration symptoms and you should drink enough during the day so your urine is a pale clear colour (unless otherwise instructed by your doctor). It is also important to drink when there is a higher risk of dehydrating, for example, if you're vomiting, sweating or you have diarrhoea.

- **Fungal genital infections** – As these medicines increase the glucose in your urine, there is an increased risk of certain infections, such as thrush around the vagina and penis.
However, this is easily treated (usually with a cream) and a pharmacist or your GP can give you advice if irritation or itching occurs in these areas. Washing your genital area with warm water, using non-perfumed soap and avoiding wearing tight underwear will reduce the risk of infection.

Uncommon:

- **An increase of acid in the blood** – SGLT-2 inhibitors may cause certain acids (ketones) to build up in the blood. This is called **diabetic ketoacidosis (DKA).** This is a rare event but can happen ***even when your blood glucose is normal.*** Symptoms include nausea and vomiting, abdominal pain, rapid breathing, and dehydration e.g. dizziness and thirst. Sufferers' breath smells like pear-drops/nail varnish remover.

  The risk of DKA is increased if you do not eat for long periods, become dehydrated, reduce your insulin dose too quickly, drink excessive alcohol or are unwell. **Please seek medical advice before starting any new diet** particularly very low carbohydrate diets (also called ketogenic diets) as these can increase the ketones in the blood.

  DKA is a serious health condition. **If you believe you are developing symptoms of DKA then please seek urgent medical assessment** reporting your concern and the medication you are taking.

- **Foot disease leading to toe or other amputation** – if you have been told you have an "at risk foot" you should clarify with your doctor if you should start or remain on one of these medicines. If you have an active foot ulcer or problem with the blood supply in your leg you should stop these medicines.

Exceedingly rare:

- **"Fournier's gangrene"** – this is an exceedingly rare infection in the groin area requiring urgent medical attention. The main symptom to be aware of is severe pain on pressing the skin over the groin area. If this develops, stop your SGLT-2 inhibitor and seek clinical advice.

Should I stop taking these tablets if I become unwell?

It is best practice to use **good sick day guidance** with these medications. You should miss them out if unwell especially in the presence of vomiting, diarrhoea or fever. You should also miss out your SGLT-2 inhibitor if you are fasting (e.g. before an elective surgical operation). You can restart them when you are better, however if you remain unwell (e.g. for longer than 48 hours), we advise you seek medical advice from your GP/Pharmacist/NHS 111.

Sick day guidance for people with diabetes

If you are unwell (vomiting, diarrhoea, fever, sweats and shaking), you should **temporarily** miss out the medicines listed below. If you are unsure or have any questions please seek medical advice.

- **blood pressure pills** – e.g. ramipril, lisinopril, losartan or other medicine sending with -sartan or -pril
- **diuretics** - (water tablets) e.g. furosemide, bumetanide, spironolactone
- **diabetes pills** -e.g. metformin, and your SGLT-2 inhibitor/'gliflozin'. Do **not** stop taking your insulin

If you have diabetes, you must increase the number of times you check your blood glucose levels. If they run too high or low, please seek medical advice.
Restart your medicines as soon as you are well and eating normally. Please seek medical advice if you continue to feel unwell after 48 hours.

6.4 Example patient information sheet for a person being initiated on an SGLT-2 inhibitor who does not have diabetes

Getting the most from your sodium glucose co-transporter-2 inhibitors (SGLT-2i)

(For people without diabetes)

Information for patients, relatives and carers

Introduction

This leaflet has been designed to give you information about sodium glucose co-transporter- 2 inhibitors (SGLT- 2 inhibitors) and answers some of the questions that you or those who care for you may have about these medicines. It is not meant to replace the discussion between you and your medical team but aims to help you understand more about what is discussed. If you have any questions about the information below, please contact the person who provided you with this leaflet.

What are SGLT-2 inhibitors and who benefits from using them?

You are being treated with one of the SGLT-2 inhibitors medicines, sometimes known as "gliflozins" or "flozins". These include, canagliflozin (Invokana), dapagliflozin (Forxiga), empagliflozin (Jardiance) and ertugliflozin (Steglatro).

These medicines were initially developed to treat people with diabetes as they lower blood glucose (sugar) by increasing the amount of glucose in the urine. They have been found to have additional benefits that include protecting the kidneys and heart, slowing the decline in kidney function and reducing the risk of heart failure and heart attacks in individuals at most risk.

Are there any side effects?

Common:

- **Dehydration** – Dehydration is when your body does not have as much water as it needs. These medicines increase the amount of urine that you pass so may cause dehydration. To prevent dehydration, drink fluids when you feel any dehydration symptoms and you should drink enough during the day so your urine is a pale clear colour (unless otherwise instructed by your doctor). It is also important to drink when there is a higher risk of dehydrating, for example, if you are vomiting, sweating or you have diarrhoea.

- **Fungal genital infections** – As these medicines increase the glucose in your urine, there is an increased risk of certain infections, such as thrush around the vagina and penis. However, this is easily treated (usually with a cream) and a pharmacist or your GP can give you advice if irritation or itching occurs in these areas. Washing your genital area with warm water, using non-perfumed soap and avoiding wearing tight underwear will reduce the risk of infection.

Uncommon side effects that are expected to be extremely rare in people without diabetes

There are a series of side effects which may almost exclusively affect people with diabetes. These are uncommon or extremely rare, and are highly unlikely to affect people without diabetes:

- **An increase of acid in the blood** – SGLT-2 inhibitors may cause certain acids (ketones) to build up in the blood. This is called **ketoacidosis**. This is an event that occurs rarely in people without diabetes. The risk of ketoacidosis is increased if you do not eat for long periods, become dehydrated, drink excessive alcohol or are severely unwell. Please seek medical advice before starting any new diet particularly very low carbohydrate diets (also called ketogenic diets) as these can increase the ketones in the blood. Ketoacidosis presents with nausea and vomiting, abdominal pain, rapid breathing, and dehydration e.g. dizziness and thirst. Sufferers' breath smells like pear-drops/nail varnish remover. Ketoacidosis requires urgent medical assessment.

- **Foot disease leading to toe or other amputation** – if you have been told you have an "at risk foot" because of poor blood supply you should clarify with your doctor if you should start or remain on one of these medicines. If you have an active foot ulcer or problem with the blood supply in your leg you should stop these medicines.

- **Hypoglycaemia (low blood glucose)** – this usually only occurs if SGLT-2 inhibitors are used in people with diabetes in combination with insulin.

- **"Fournier's gangrene**" – this is an exceedingly rare infection in the groin area requiring urgent medical attention. The main symptom to be aware of is severe pain on pressing the skin over the groin area. If this develops, stop your SGLT-2 inhibitor and seek clinical advice.

Should I stop taking these tablets if I become unwell?

It is best practice to use **good sick day guidance** with these medications. You should miss them out if unwell especially in the presence of vomiting, diarrhoea or fever. You should also miss out your SGLT-2 inhibitor if you are fasting (e.g. before an elective surgical operation). You can restart them when you are better, however if you remain unwell (e.g. longer than 48 hours), we advise you seek medical advice from your GP/Pharmacist/NHS 111.

Sick day guidance for people without diabetes

If you are unwell (vomiting, diarrhoea, fever, sweats and shaking), you should **temporarily** miss out the medicines listed below. If you are unsure or have any questions please seek medical advice.

- **blood pressure pills** – e.g. ramipril, lisinopril, losartan or other medicines ending with -sartan or -pril
- **diuretics** - (water tablets) e.g. furosemide, bumetanide, spironolactone
- **your SGLT-2 inhibitor/"gliflozin"** – i.e. canagliflozin, dapagliflozin, empagliflozin or ertugliflozin being used to treat your kidney disease

Restart your medicines as soon as you are well and eating normally. Please seek medical advice if you continue to feel unwell after 48 hours.

Section 7: Use in special populations of specific interest

7a Type 1 diabetes mellitus (DM)

7a.1 Background

7a.1.1 Summary of trial evidence

Although previously marketed for use in type 1 diabetes mellitus (DM), there are currently no SGLT-2 inhibitors that are licensed for this indication in the UK. Although the National Institute for Health & Care Excellence (NICE) have issued a Technology Appraisal on dapagliflozin and sotagliflozin in people with type 1 DM and BMI >27 kg/m^2, the indication for use in type 1 DM for those of these medications has been withdrawn by the authorisation holder (see Section 4 for more information) (1, 2). The Association of British Clinical Diabetologists (ABCD) & Diabetes UK have released some guidance on the use of SGLT-2 inhibitors in type 1 DM, which highlights the need for closer monitoring and education in this population, particularly in relation to ketone monitoring (3).

Glycaemic control

There are a number of randomised controlled trials (RCTs) evaluating the use of SGLT-2 inhibitors in people with insufficiently controlled type 1 DM. The DEPICT-1 and -2 trials demonstrated that the addition of dapagliflozin (at either 5 mg and 10 mg daily doses) to existing insulin-based regimens reduces glycosylated haemoglobin (HbA1c) and body weight compared to placebo without provoking additional hypoglycaemia (4, 5). The EASE-1 trial examined the short-term (28 days) efficacy of adding empagliflozin to insulin and found that HbA1c was significantly reduced by between −3.8 to −5.4 mmol/mol (-0.35% to -0.49%; p<0.05) when compared with placebo (6). Similarly, a trial assessing canagliflozin in people with type 1 DM found that significantly more people achieved a reduction of >0.4% in HbA1c with 100 mg and 300 mg of canagliflozin versus placebo (36.9%, 41.4% vs 14.5%; p<0.001) (7). In summary, evidence from RCTs indicates that SGLT-2 inhibitors as an adjunct to insulin improves glycaemic control in people with insufficiently controlled type 1 DM.

Kidney outcomes

There are no large clinical trials reporting kidney outcomes of SGLT-2 inhibitors in people with type 1 DM. There are ad hoc analyses of larger trials that show benefit in reduction of urinary albumin-to-creatinine ratio (uACR). DEPICT-1 and -2 trials showed that after a year, the addition of dapagliflozin to insulin resulted in a dose-dependent reduction of uACR (≥3 mg/mmol) of −13.3% (95% confidence interval [CI] −37.2 to 19.8; for the 5mg dose) and −31.1% (95%CI −49.9% to −5.2%; for the 10 mg dose), compared to placebo (4, 5).

The inTandem-1 and -2 trials evaluated the effect of sotagliflozin versus placebo in people with type 1 DM. Ad hoc analysis of the inTandem-1 and -2 trials showed that in a subgroup of participants (n = 196) with mean elevated albuminuria (uACR ≥30 mg/g [3.4 mg/mmol]) at baseline, an initial dose-dependent reduction of mean albuminuria by −31.4% (SE 11.3; p=0.003) from baseline was observed for sotagliflozin 400 mg at week 24. This reduction was attenuated (and non-significantly different to placebo) at week 52 (−18.3% [SE 13.8]; p=0.18) (8, 9). Small trial size and short follow-up means there are no data available on regulator-accepted

endpoints based on glomerular filtration rate (GFR) or need for kidney replacement therapy (or cardiovascular outcomes) in type 1 DM, even from the 1402 participant inTandem-3 trial (10).

Analyses of the effect of empagliflozin on uACR and eGFR slopes in the 68 participants with type 1 DM randomised into EMPA-KIDNEY are currently unavailable.

Safety

In the DEPICT-1 trial, insulin dose was reduced by an average (mean) of 14% in the first 2 weeks of starting dapagliflozin (4). In the DEPICT-2 trial, the reduction of insulin was between 10-11% (5). Both trials found no significant difference in the rates of hypoglycaemia between SGLT-2 inhibitors and placebo. During the first 7 days of the EASE-1 trial, insulin doses were kept as stable as possible, thereafter being adjusted according to glycaemic control. During these first 7 days, the rate of hypoglycaemia was higher in the 10 mg and 25 mg empagliflozin arm, than the 2.5 mg empagliflozin and placebo arm. After the first week however, when insulin doses were adjusted, there was no significant difference in hypoglycaemia rates (6). See section 5b for more information on severe hypoglycaemia risk with SGLT-2 inhibition.

Whilst there were no diabetic ketoacidosis (DKA) events during the short EASE-1 trial (6), the DEPICT-1 and -2 trials demonstrated an increased risk of DKA in the SGLT-2 inhibitor group when compared to placebo (46.2 versus 12.7 events per 1000 person years) (4, 5). In an 18 week trial, the DKA rates were increased in the canagliflozin 100 mg or 300 mg, at 4.3% and 6.0% in comparison to 0% in the placebo group (7). Differences in the adjudication definitions of DKA and recruited populations can influence the cross-trial comparability and generalisability of these DKA rates from trials. It is not necessarily the case that one SGLT-2 inhibitor carries greater susceptibility for DKA than another. See section 5a for more details on DKA.

7a.1.2 Quality of the evidence

There are no data as yet, indicating whether SGLT-2 inhibitors provide kidney benefits in relation to regulatory accepted kidney endpoints based on GFR in people with type 1 DM. There are data that clearly demonstrate substantial excess absolute risk of DKA in people with type 1 DM. The lack of trial evidence on kidney outcomes in people with type 1 DM precludes direct comparisons of the potential balance of the definite risks versus the potential benefits.

7a.2 Recommendations for implementation

1. **We recommend that SGLT-2 inhibitors be initiated in people with type 1 DM, only under the strict direction of the diabetes team (Grade 1C).**

2. **We suggest considering referring people with type 1 DM to the specialist diabetes team, for consideration of an SGLT-2 inhibitor, if they have an eGFR ≥20 mL/min/1.73m^2, and a uACR ≥25 mg/mmol despite being on maximum tolerated ACEi/ARB (Grade 2C).**

3. **We recommend all people with type 1 DM started on SGLT-2 inhibitors be provided with ketone monitoring, be advised on the signs and symptoms of DKA and to seek immediate medical advice if any of these symptoms develop or ketone levels are >0.6 mmol/L (Grade 1B).**

Rationale: There is currently insufficient evidence to recommend the use of SGLT-2 inhibitors as an adjunct

to existing therapies in the management of diabetic nephropathy in people with type 1 DM. Evidence of kidney benefits in people with type 2 DM makes this plausible but such results cannot be readily extrapolated to people with type 1 DM. Clinicians may wish to discuss treatment options with their patients and other specialists in cases where proteinuria persists despite current standard treatment. Thresholds for referral to the specialist diabetes team have been updated in the current version of this guideline in line with evidence available from trials of SGLT-2 inhibition irrespective of the presence or absence of diabetes. Furthermore, the grading for Recommendation number 2 has been increased from 2D to 2C, given evidence available from the InTandem3 trial (10).

7a.3 Clinical research recommendations

1. To establish whether the cardiovascular and kidney benefits of SGLT-2 inhibitors extend to those with type 1 DM.
2. To establish the safety of SGLT-2 inhibitors in people with type 1 DM and chronic kidney disease.

7b Kidney transplant recipients

7b.1 Background

7b.1.1 Summary of trial evidence

Kidney transplantation offers advantages compared to other forms of kidney replacement therapy for many people with kidney failure, including people with diabetic nephropathy. These include potentially better quality of life and longer survival (11, 12). Currently, about 17% of incident UK kidney transplants are performed for diabetic nephropathy (13), and once transplanted new onset diabetes after transplantation (NODAT [also known as post-transplant DM]) is common. NODAT is important to identify as it predicts future mortality and graft failure (14). Its incidence has fallen over the last two decades and varies by diagnostic criteria and immunosuppressive protocol, but can still be as high as 10-20% during the first year after transplantation (15).

Sodium glucose co-transporter 2 (SGLT-2) inhibitors may have the potential to prevent and treat NODAT. From their mechanism of action, they may also provide cardioprotective and kidney benefits in people with a kidney transplant (see section 1 for details of SGLT-2 inhibitors' mechanisms of action). However, kidney transplant recipients are also particularly susceptible to graft function loss with ascending urinary tract infections, and immunosuppression predisposes to genital mycotic infections. People on immunosuppression for kidney disease and those with kidney transplants have been excluded from the reported large placebo- controlled clinical outcome SGLT-2 inhibitor trials, including those specifically recruiting people with chronic kidney disease (CKD) (see Table 1.1 in section 1) (16-18).

A series of small observational studies have reported experience of prescribing SGLT-2 inhibitors to kidney transplant recipients (19, 20), but our literature review identified a single randomised trial by Halden and colleagues (21). This single-centre 24-week placebo-controlled trial was conducted in a population of people with NODAT who were at least one year since transplantation with stable graft function. Below we provide a summary of this single trial and offer a summary statement.

Effects of SGLT-2 inhibition on hyperglycaemia, weight, blood pressure, kidney function, and adverse events in NODAT from Halden et al. (21).

Halden et al. analysed 44 participants with NODAT randomised 1:1 to empagliflozin 10mg versus matching placebo. Mean baseline kidney function was just over 60 mL/min/1.73m^2 and median glycosylated haemoglobin (HbA1c) was ~6.9% (~52 mmol/mol). At 24 weeks, median change in HbA1c from baseline was -0.2% (-2.0 mmol/mol) among those allocated empagliflozin versus +0.1% (+1.0 mmol/mol) among those allocated placebo (difference in change between arms p=0.025: Table 7b.1). There were no significant differences in other glycaemic or insulin parameters, including fasting or 2-hour blood glucose, insulin or c-peptide concentrations (Table 7b.1) (21). The effect on HbA1c varied by baseline estimated glomerular filtration rate (eGFR), with the expected pattern of larger HbA1c reductions among those with an eGFR ≥60 mL/min/1.73m^2, and almost no difference in HbA1c among those with an eGFR of 40-50 mL/min/1.73m^2 (people with an eGFR <30 mL/min/1.73m^2 were excluded). This attenuated effect on HbA1c corresponded to a linear decrease in glucose excretion with lower eGFR.

Table 7b.1: Effects of empagliflozin 10mg versus placebo on glycaemia, weight, blood pressure and kidney function in kidney transplant recipient with new onset diabetes after transplantation from Halden et al. (21)

Assessment	Empagliflozin			Placebo			*P value**
	Baseline	Week 24	**Change**	Baseline	Week 24	**Change**	
**HbA1c (%)**	6.9 (6.5, 8.2)	6.7 (6.3, 7.5)	**-0.2 (-0.6, -0.1)**	6.6 (6.1, 7.2)	6.9 (6.4, 7.4	**0.1 (-0.1, 0.4)**	***0.025***
**Fasting plasma glucose (mmol/L)**	8.0 (7.3, 8.6)	7.2 (6.6, 8.1)	**-0.65 (-1.2, -0.13)**	7.3 (6.5, 8.6)	7.5 (6.8, 8.4)	**0.30 (-0.45, 0.55)**	***0.27***
**2-hour glucose after oral glucose tolerance test (mmol/L)**	15.6 (13.3, 17.7)	14.2 (12.4, 15.6)	**-1.75 (-3.7, 0.93)**	13.3 (10.3, 17.4)	14.1 (10.5, 16.9)	**-0.40 (-1.4, 1.4)**	***Not significant, approaching 1.0***
**Body weight (kg)**	92.0 (81.8, 104.5)	88.8 (79.0, 100)	**-2.5 (-4, -0.05)**	84.0 (79.3, 94.0)	85.0 (79.5, 97.5)	**1.0 (0.0, 2.0)**	***0.01***
**Mean 24-h systolic BP (mmHg)**	136 (131, 147)	142 (126, 148)	**2 (-5, 6)**	135 (127, 146)	137 (132, 143)	**2 (-7, 6)**	***Not significant, approaching 1.0***
**Mean 24-h diastolic BP (mmHg)**	76 (71, 82)	76 (70, 82)	**0 (-5, 2)**	78 (74, 85)	80 (74, 86)	**1 (-3, 4)**	***Not significant, approaching 1.0***
**eGFR (mL/min/1.73 m^2)**	66 (57, 68)	61 (56, 67)	**-3 (-7, 0)**	59 (52, 72)	59 (52, 67)	**-1.0 (-2.8, 0.75)**	***Not significant, approaching 1.0***
**Renal glucose excretion (g/24 h)**	0.45 (0.20, 1.48)	46.0 (36.8, 68.6)	**45.9 (36.1, 64.3)**	0.5 (0.1, 2.3)	1.5 (0.2, 4.5)	**0.20 (0.0, 1.6)**	***<0.001***

BP= blood pressure; eGFR=estimated glomerular filtration rate; HbA1c=glycosylated haemoglobin; * *p* values calculated from difference in change between baseline and 24 weeks among the 22 allocated empagliflozin versus the 22 allocated placebo.

At 24 weeks, there was evidence that empagliflozin reduced weight, but there was no difference in blood pressure between the study arms (Table 7b.1). Blood levels of calcineurin inhibitors and everolimus were also not significantly different between the empagliflozin and placebo arms (21). At 8 weeks of follow-up, eGFR acutely declined by an average of -4mL/min/1.73m^2 among those allocated empagliflozin versus -1 mL/min/1.73m^2 among those allocated to placebo. By 24 weeks, this difference had reduced to -3 versus -1 mL/min/1.73m^2 (Table 7b.1).

Empagliflozin was generally well tolerated. One participant allocated empagliflozin withdrew from the trial due to urosepsis. There were three urinary tract infections in each arm, and no reported episodes of rejection (21).

7b.1.2 Quality of the evidence

Currently, the reported randomised evidence of the effects of SGLT-2 inhibitors in kidney transplant recipients is limited to less than 50 trial participants with NODAT followed for less than 6 months from a single centre. There is therefore currently insufficient data to provide specific evidence-based recommendations for safe use of SGLT-2 inhibition in this population (22). There are a number of older anti-diabetic treatments with well-known safety profiles which can be used to treat hyperglycaemia in NODAT. This section of this guideline will be updated as more randomised evidence becomes available.

7b.2 Summary statements

1. **There is currently insufficient evidence on safety and efficacy to provide recommendations for use of SGLT-2 inhibition in people with a functioning kidney transplant.**

2. **Any use of SGLT-2 inhibition to treat diabetes mellitus in a kidney transplant recipient should be evaluated by multi-disciplinary discussion (Grade 2D)**

   Note: effects on glycaemic control at an eGFR <60 mL/min/1.73m^2 in people with a kidney transplant appear small and potential risk of complications from urinary tract infection should be considered.

7b.3 Clinical research recommendations

The generation of reliable randomised trial evidence for transplant recipients is a key research recommendation. Please refer to section 2.3.

7c Acute decompensated heart failure

7c.1 Background

7c.1.1 2023 Update to guideline structure

In the UKKA 2021 guideline, this section summarised evidence in populations with heart failure with preserved ejection fraction (HFpEF) and in acute decompensated heart failure (ADHF). Since then, an additional large randomised controlled trial has been reported which demonstrated the efficacy of dapagliflozin at reducing risk among people with heart failure with mildly reduced or preserved ejection fraction (the DELIVER trial (23)). Based on the findings from both DELIVER and the EMPEROR-PRESERVED trials, there is now sufficient evidence to provide grade 1A recommendations for use in HFpEF alongside heart failure with reduced ejection fraction (HFrEF) in sections 2 and 3. Consequently, this section now focuses primarily on the more limited evidence available for recommendation of use in ADHF.

7c.1.2 Summary of trial evidence in acute decompensated heart failure

Since publishing the UKKA 2021 guideline, there is new evidence supporting a role for SGLT-2 inhibitors within this context. The trials can be divided into those reporting clinical benefit versus those reporting on clinical surrogates for fluid status, biochemical markers of heart failure status or safety information, such as worsening kidney function. Relevant trials are summarised in table 7c.1.

Clinical benefits of SGLT-2 inhibition in ADHF

The largest reported trial is EMPULSE (24), which randomised 530 participants to empagliflozin 10mg once daily versus placebo. EMPULSE eligibility aimed to recruit participants with an acute heart failure admission irrespective of diabetes status, and included eGFRs down to 20 mL/min/1.73m^2. ~10% of participants had an eGFR <30 (mean eGFR 52) mL/min/1.73m^2. 32% of participants had HFpEF (i.e. left ventricular ejection fraction [LVEF] >40%). The primary outcome was a hierarchical composite based on death from any cause, number of heart failure events, time to first heart failure event, and a change in Kansas City Cardiomyopathy Questionnaire Total Symptom Score (KCCQ-TSS). The main result was a favourable win ratio for empagliflozin of 1.36 [95%CI 1.09-1.68], p=0.005. This result can be thought of as, for any random pair of participants, the odds of a better outcome was increased by 36% among those allocated to empagliflozin compared to placebo. Primary outcome events included 11 deaths (4.2%) in the empagliflozin group and 22 (8.3%) in the placebo group, and 28 participants (10.6%) in the empagliflozin group had a heart failure event versus 39 participants (14.7%) in the placebo group. For the outcome of a ≥5 point difference in the KCCQ-TSS (from baseline to day 90), there were 35.9% wins for the empagliflozin group versus 27.5% for the placebo group. The information on the benefits of empagliflozin were therefore based mostly on participant-reported KCCQ score, but there were effects on mortality and heart failure outcomes higher in the outcome hierarchy that were also consistent with benefit on these clinical outcomes. These benefits were consistent regardless of eGFR and diabetes status. The trial found no difference in serious adverse events in the empagliflozin versus the placebo group (32.3% versus 43.6%), and “acute renal failure” occurred in 7.7% of participants in the empagliflozin group versus 12.1% of participants in the placebo group, consistent with other evidence than SGLT-2 inhibition reduces risk of acute kidney injury by about a quarter (25).

Effects of SGLT-2 inhibition on markers of cardiac congestion in ADHF

The EMPULSE trial also demonstrated that several markers of cardiac congestion were improved by allocation to empagliflozin. These included weight loss, improved clinical congestion score, and reduced NT-proBNP at day 15 after randomisation (26). Of note, improvements in body weight change from baseline (including when adjusted per total daily loop diuretic dose) were maintained up to day 90 post-randomisation.

Several small prospective trials (27-31) have reported on similar surrogate markers of cardiac congestion, including NT-proBNP, weight loss, diuresis and diuretic dose. Benefits are typically identified within the first week of admission or at discharge. They indicate that the addition of SGLT-2 inhibition to standard treatment leads to reduced diuretic doses and improved diuresis, but there are variable effects on NT-proBNP levels (28, 29, 32).

The effects of SGLT-2 inhibition in ADHF on kidney function and CKD

Several trials (24, 27-32) have addressed the kidney safety of SGLT-2 inhibition in ADHF, indicating that the addition of the agent does not adversely affect kidney function when used in participants with decreased eGFR. Where declines in eGFR were observed, these do not exceed expected levels (section 1.3.2) and follow-up data from multiple trials have proven the longer term benefits on kidney disease progression outcomes and acute kidney injury in people with CKD (17, 18, 33, 34). Participants with kidney failure (i.e. eGFR <15 ml/min/1.73m^2 or on dialysis) have not been included in the ADHF trials reported to date. Table 7c.1 provides a summary of the relevant data pertaining to kidney safety by trial. Taken together, these data support the use of SGLT-2 inhibitors in ADHF to assist diuresis during admission and indicate that these agents are safe and efficacious for those presenting with an eGFR >15 mL/min/1.73m^2. Given the positive effects of these agents in chronic stable heart failure (23, 35-38), initiating their use in ADHF will help maximise implementation for grade 1 recommendations.

Upcoming trials of SGLT-2 inhibition in ADHF

Several randomised trials are currently underway to further evaluate the role of SGLT-2 inhibition in people presenting to hospital with ADHF. The largest of these, DAPA ACT HF-TIMI 68 (39) aims to randomise ~2400 participants to two months of treatment with dapagliflozin or placebo. Participants will be enrolled within 24 hours to 14 days of hospital admission if they have ADHF with intensification of therapy during admission. Individuals with eGFR ≥25 mL/min/1.73m^2 will be enrolled, irrespective of diabetes status or ejection fraction. The estimated study completion date at the time of writing is Q2/2023.

7c.1.3 Quality of the evidence

There are relatively few large randomised trials investigating SGLT-2 inhibition for the treatment of ADHF. With the exception of EMPULSE, reported trials are generally small, often open-label and with limited power to assess clinical outcomes.

Table 7c.1

Randomised, placebo controlled trials with clinical primary outcome						
**Trial**	**Population (number)**	**Kidney function (average eGFR [mL/min/1.73m²])**	**HF definition (LVEF average %)**	**Primary outcome**	**Secondary outcome**	**Kidney safety**
EMPULSE (24) Empagliflozin<br><br>Randomised, double blind placebo-controlled, multicentre	Acute HF admission (530)<br><br>Excluded eGFR <20<br><br>T2DM: 45%	Median eGFR (IQR): Empa 50.0 (36-65); placebo 54 (39-70).<br><br>eGFR <30: Empa 10.2%; placebo 9.1%	LVEF ≤40%: Empa 68.7%, placebo 64.9%<br>LVEF >40%: Empa 28.7%, placebo 35.1%	Win ratio* of clinical benefit day 90: 1.36 95% CI (1.09-1.68), p=0.0054	CVD or HF, events per 100 patient years: HR 0.69, 95%CI (0.45-10.08) favours Empa<br><br>Diuretic response (Kg weight loss per mean daily loop diuretic dose) day 30: Empa -3.80 (-5.39 to -2.20); placebo -1.01 (-2.59 to 0.57)<br><br>Change in BNP (AUC) day 30: Adjusted geometric mean ratio 0.9 (95% CI 0.82-0.98)<br><br>HHF at 30 days: NSD	No increased adverse events of AKI, "renal failure" or UTI
**Prospective trials with primary outcome for surrogate markers of congestion or fluid status**						
**Trial Intervention Design**	**Population (number)**	**Kidney function (average eGFR [mL/min/1.73m²])**	**HF definition (LVEF average %)**	**Primary outcome**	**Secondary outcome**	**Kidney safety**
EMPA-REPSONSE-AHF (31) Empagliflozin<br><br>Randomised, placebo-controlled, parallel group, multicentre	Acute HF admission (80)<br><br>Excluded eGFR <30<br><br>T2DM 83%	Mean eGFR: Empa and placebo: 55	Not measured LVEF known in 58%: average LVEF 37%	Dyspnoea VAS at day 4: NSD<br><br>Diuretic response (Kg/40mg) day 4: NSD<br><br>Change in NT-proBNP day 4: NSD<br><br>Length of stay: NSD	Worsening HF/rehospitalisation HF/death at 60 days: Empa 10%; placebo 33%, p=0.014	No increased adverse renal events or UTI
Ibrahim et al, 2020 (27) Dapagliflozin<br><br>Randomised, open label, parallel group controlled multicentre	Acute HF admission to cardiac critical care (100)<br><br>Excluded eGFR <45<br><br>All T2DM	Mean serum creatinine (±SD): Dapa 124 μmol/L (± 35); control 117 μmol/L (± 27)	LVEF <40%: All	Diuretic response in Kg/40mg furosemide: Dapa –0.089 ±0.04; control –0.042 ±0.03, p<0.001	Serum creatinine at discharge: Dapa 135 μmol/L; control 123 μmol/L; p=0.009<br><br>Length of stay: NSD	Serum creatinine level % change (mean ±SD) at discharge: Dapa 8.76 ±2.5; control 12.34 ±2.9, p=0.349
EMPAG-HF (28)	Acute HF admission	Mean eGFR ±SD: Empa 58.2 ±19.3;	Mean LVEF Empa 45%,	Urine output over 5 days: Empa	Patient reported outcomes: NSD	Change in eGFR: NSD (secondary

Empagliflozin<br><br>Randomised, double-blind, placebo controlled, single centre	(60)<br><br>Excluded eGFR <30<br><br>T2DM 38%	placebo 62.2 ± 18.2	placebo 44%.<br><br>LVEF ≤ 30%: Empa 20.7%, placebo 21.4%	10775mL; placebo 8650mL, [95%CI 840-3550], p=0.003	Change in NT-proBNP day 5: Empa –1861, placebo –727.2 pg/mL; quotient in slope 0.89 [95%CI 0.83-0.95, p=<0.001]<br><br>Body weight reduction day 5: NSD<br><br>Diuretic efficiency day 5 (mL of urine/mg furosemide) + 14.1 mL/mg for Empa [95%CI 0.6-27.7, p<0.041]	outcome)
Tamaki et al, 2021 (32)<br>Empagliflozin<br><br>Randomised, open label, single centre	Acute HF admissions (59)<br><br>Excluded eGFR <15<br><br>All T2DM	Mean (SD) eGFR: Empa 40 (31-54); control 35 (23-54)	HFrEF Empa 43%, control 55%<br>HFmrEF Empa 10%, control 17%<br>HFpEF Empa 47%, control 28%	NT-proBNP day 7: lower in Empa group, p=0.04	Change in HR, SBP: NSD<br><br>Change in body weight: NSD	Worsening renal function: NSD (secondary outcome)
Thiele et al, 2022 (29)<br>Empagliflozin<br><br>Randomised, placebo-controlled single centre	Acute HF admissions (19)<br><br>Pre-admission eGFR not specified<br><br>T2DM 26%	Mean ±SD eGFR: Empa 63 ±22, placebo 56 ±16	Mean ±SD LVEF%: Empa 34 ±11, placebo 38 ±11	Cardiac output (day 1, 3 and 7): NSD	Cumulative diuretic dose day 7: NSD	Worsening renal function day 7: NSD (creatinine and cystatin C)<br><br>Urinary concentrations of tubular kidney injury biomarkers (TIMP-2 and IGFBP-7) lower in empa group at day 7
**Prospective trials with primary outcome for change in kidney function**						
**Trial Intervention Design**	**Population (number)**	**Kidney function (average eGFR (mL/min/1.73m²))**	**HF definition (LVEF average %)**	**Primary outcome**	**Secondary outcome**	**Kidney safety**
Charaya et al, 2021 (30)<br>Dapagliflozin<br><br>Randomised, open label, parallel group, controlled, single centre	Acute HF admission (102)<br><br>Excluded eGFR <30<br><br>T2DM 32%	Mean eGFR ±SD: Dapa 55.6 ±18.17; placebo: 52.7 ±17.34.<br><br>EGFR <45: 25%	LVEF ≥50%: 39%<br>LVEF 41-49%: 17%<br>LVEF ≤40%: 43%	Change in eGFR at discharge:<br>Dapa –4.0 (-10.25 to 9.1); Placebo 2.7 (-3.85 to 9), p=0.049	Mean diuretic dose: Dapa 78.46 ±38.95 mg/day; placebo 102.82 ±31.26 mg/day; p=0.001<br><br>Weight loss at discharge: Dapa -4100g; placebo –3000g; p=0.02<br><br>No difference in mortality or rehospitalisation at 30 days	Worsening renal function (>0.3mg/dl for more than 48 hours): NSD<br><br>Dapa withdrawn in 12% for eGFR <30 and resumed in 4%<br><br>No incidence of UTI

Dapa=dapagliflozin; eGFR=estimated glomerular filtration rate; Empa=empagliflozin; HF=heart failure; HHF=hospitalisation for heart failure; HFmrEF=heart failure with mid-range ejection fraction; HFpEF=heart failure with

preserved ejection fraction; HFrEF=heart failure with reduced ejection fraction; LVEF=left ventricular ejection fraction; NSD=no significant difference; VAS=Visual Analogue Scale; SD=standard deviation; T2DM=type 2 diabetes; UTI=urinary tract infection.

*hierarchical assessment of all-cause mortality, number and time to first HHF and change in KCCQ-TSS using a stratified win ratio.

7c.2 Recommendations for implementation

We suggest initiating SGLT-2 inhibition in people with CKD (eGFR ≥20 mL/min/1.73m^2) with acute decompensated heart failure. (2B)

Rationale: Multiple small-scale randomised controlled trials provide evidence that SGLT-2 inhibition is safe in a population presenting to hospital with ADHF. Furthermore, the moderate-sized EMPULSE trial demonstrates that individuals treated with SGLT-2 inhibition for ADHF are more likely to yield clinical benefit than those treated with placebo. Trials in ADHF enrolled people with eGFR >15 mL/min/1.73m^2, with the largest trial (EMPULSE) enrolling those with eGFR ≥20 mL/min/1.73m^2. More evidence from large, well-conducted randomised controlled trials (such as the DAPA ACT HF-TIMI 68 trial) will provide more comprehensive evidence to support this recommendation.

See section 2 & 3 for recommendations for use in other forms of heart failure or to modify cardiovascular risk.

7c.3 Clinical research recommendations

Large randomised placebo-controlled clinical trials powered to assess hard clinical outcomes in people with ADHF.

7.1 References

1. Zynquista: https://www.ema.europa.eu/en/medicines/human/EPAR/zynquista (accessed 14th December 2022).

2. Dapagliflozin (Forxiga): no longer authorised for treatment of type 1 diabetes mellitus. https://www.gov.uk/drug-safety-update/dapagliflozin-forxiga-no-longer-authorised-for-treatment-of-type-1-diabetes-mellitus (Accessed 10th January 2023).

3. Dashora U, Patel DC, Gregory R, Winocour P, Dhatariya K, Rowles S, et al. Association of British Clinical Diabetologists (ABCD) and Diabetes UK joint position statement and recommendations on the use of sodium-glucose cotransporter inhibitors with insulin for treatment of type 1 diabetes (Updated October 2020). Diabet Med. 2021;38(2):e14458.

4. Dandona P, Mathieu C, Phillip M, Hansen L, Tschöpe D, Thorén F, et al. Efficacy and Safety of Dapagliflozin in Patients With Inadequately Controlled Type 1 Diabetes: The DEPICT-1 52-Week Study. Diabetes Care. 2018;41(12):2552-9.

5. Mathieu C, Dandona P, Gillard P, Senior P, Hasslacher C, Araki E, et al. Efficacy and Safety of Dapagliflozin in Patients With Inadequately Controlled Type 1 Diabetes (the DEPICT-2 Study): 24-Week Results From a Randomized Controlled Trial. Diabetes Care. 2018;41(9):1938-46.

6. Pieber TR, Famulla S, Eilbracht J, Cescutti J, Soleymanlou N, Johansen OE, et al. Empagliflozin as adjunct to insulin in patients with type 1 diabetes: a 4-week, randomized, placebo-controlled trial (EASE-1). Diabetes Obes Metab. 2015;17(10):928-35.

7. Henry RR, Thakkar P, Tong C, Polidori D, Alba M. Efficacy and Safety of Canagliflozin, a Sodium-Glucose Cotransporter 2 Inhibitor, as Add-on to Insulin in Patients With Type 1 Diabetes. Diabetes Care. 2015;38(12):2258-65.

8. Buse JB, Garg SK, Rosenstock J, Bailey TS, Banks P, Bode BW, et al. Sotagliflozin in Combination With Optimized Insulin Therapy in Adults With Type 1 Diabetes: The North American inTandem1 Study. Diabetes Care. 2018;41(9):1970-80.

9. Danne T, Cariou B, Banks P, Brandle M, Brath H, Franek E, et al. HbA(1c) and Hypoglycemia Reductions at 24 and 52 Weeks With Sotagliflozin in Combination With Insulin in Adults With Type 1 Diabetes: The European inTandem2 Study. Diabetes Care. 2018;41(9):1981-90.

10. Garg SK, Henry RR, Banks P, Buse JB, Davies MJ, Fulcher GR, et al. Effects of Sotagliflozin Added to Insulin in Patients with Type 1 Diabetes. New England Journal of Medicine. 2017;377(24):2337-48.

11. Laupacis A, Keown P, Pus N, Krueger H, Ferguson B, Wong C, et al. A study of the quality of life and cost-utility of renal transplantation. Kidney Int. 1996;50(1):235-42.

12. McDonald SP, Russ GR. Survival of recipients of cadaveric kidney transplants compared with those receiving dialysis treatment in Australia and New Zealand, 1991-2001. Nephrol Dial Transplant. 2002;17(12):2212-9.

13. UK Renal Registry (2022) UK Renal Registry 24th Annual Report – data to 31/12/2020, Bristol, UK. Available from https://ukkidney.org/audit-research/annual-report. (Accessed 10th January 2023).

14. Kasiske BL, Snyder JJ, Gilbertson D, Matas AJ. Diabetes mellitus after kidney transplantation in the United States. Am J Transplant. 2003;3(2):178-85.

15. Jenssen T, Hartmann A. Post-transplant diabetes mellitus in patients with solid organ transplants. Nat Rev Endocrinol. 2019;15(3):172-88.

16. Perkovic V, Jardine MJ, Neal B, Bompoint S, Heerspink HJL, Charytan DM, et al. Canagliflozin and Renal Outcomes in Type 2 Diabetes and Nephropathy. N Engl J Med. 2019;380(24):2295-306.

17. Heerspink HJL, Stefánsson BV, Correa-Rotter R, Chertow GM, Greene T, Hou F-F, et al. Dapagliflozin in Patients with Chronic Kidney Disease. New England Journal of Medicine. 2020;383(15):1436-46.

18. Empagliflozin in Patients with Chronic Kidney Disease. New England Journal of Medicine. 2022.

19. Chewcharat A, Prasitlumkum N, Thongprayoon C, Bathini T, Medaura J, Vallabhajosyula S, et al. Efficacy and Safety of SGLT-2 Inhibitors for Treatment of Diabetes Mellitus among Kidney Transplant Patients: A Systematic Review and Meta-Analysis. Med Sci (Basel). 2020;8(4).

20. Anderson S, Cotiguala L, Tischer S, Park JM, McMurry K. Review of Newer Antidiabetic Agents for Diabetes Management in Kidney Transplant Recipients. Ann Pharmacother. 2021;55(4):496-508.

21. Halden TAS, Kvitne KE, Midtvedt K, Rajakumar L, Robertsen I, Brox J, et al. Efficacy and Safety of Empagliflozin in Renal Transplant Recipients With Posttransplant Diabetes Mellitus. Diabetes Care. 2019;42(6):1067-74.

22. ABCD/RA guideline: Clinical Practice Guidelines for Management of Hyperglycaemia in Adults with Diabetic Kidney Disease: 2021 Update. https://abcd.care/sites/abcd.care/files/site_uploads/Resources/Position-Papers/Management-of-hyperglycaemia-in-adults%20-with-DKD.pdf (accessed 10th January 2023).

23. Solomon SD, McMurray JJV, Claggett B, de Boer RA, DeMets D, Hernandez AF, et al. Dapagliflozin in Heart Failure with Mildly Reduced or Preserved Ejection Fraction. New England Journal of Medicine. 2022;387(12):1089-98.

24. Voors AA, Angermann CE, Teerlink JR, Collins SP, Kosiborod M, Biegus J, et al. The SGLT2 inhibitor empagliflozin in patients hospitalized for acute heart failure: a multinational randomized trial. Nature Medicine. 2022;28(3):568-74.

25. Nuffield Department of Population Health Renal Studies Group and the SGLT2 inhibitor Meta-Analysis Cardio-Renal Trialists' Consortium. Impact of diabetes on the effects of sodium glucose co-transporter-2 inhibitors on kidney outcomes: collaborative meta-analysis of large placebo-controlled trials. The Lancet. 2022;400(10365):1788-801.

26. Biegus J, Voors AA, Collins SP, Kosiborod MN, Teerlink JR, Angermann CE, et al. Impact of empagliflozin on decongestion in acute heart failure: the EMPULSE trial. European Heart Journal. 2022.

27. Ibrahim A, Ghaleb R, Mansour H, Hanafy A, Mahmoud NM, Abdelfatah Elsharef M, et al. Safety and Efficacy of Adding Dapagliflozin to Furosemide in Type 2 Diabetic Patients With Decompensated Heart Failure and Reduced Ejection Fraction. Front Cardiovasc Med. 2020;7:602251.

28. Schulze PC, Bogoviku J, Westphal J, Aftanski P, Haertel F, Grund S, et al. Effects of Early Empagliflozin Initiation on Diuresis and Kidney Function in Patients With Acute Decompensated Heart Failure (EMPAG-HF). Circulation. 2022;146(4):289-98.

29. Thiele K, Rau M, Hartmann N-UK, Möller M, Möllmann J, Jankowski J, et al. Empagliflozin reduces markers of acute kidney injury in patients with acute decompensated heart failure. ESC Heart Failure. 2022;9(4):2233-8.

30. Charaya K, Shchekochikhin D, Andreev D, Dyachuk I, Tarasenko S, Poltavskaya M, et al. Impact of dapagliflozin treatment on renal function and diuretics use in acute heart failure: a pilot study. Open Heart. 2022;9(1):e001936.

31. Damman K, Beusekamp JC, Boorsma EM, Swart HP, Smilde TDJ, Elvan A, et al. Randomized, double-blind, placebo-controlled, multicentre pilot study on the effects of empagliflozin on clinical outcomes in patients with acute decompensated heart failure (EMPA-RESPONSE-AHF). Eur J Heart Fail. 2020;22(4):713-22.

32. Tamaki S, Yamada T, Watanabe T, Morita T, Furukawa Y, Kawasaki M, et al. Effect of Empagliflozin as

an Add-On Therapy on Decongestion and Renal Function in Patients With Diabetes Hospitalized for Acute Decompensated Heart Failure. Circulation: Heart Failure. 2021;14(3):e007048.

33. Perkovic V, Jardine MJ, Neal B, Bompoint S, Heerspink HJL, Charytan DM, et al. Canagliflozin and Renal Outcomes in Type 2 Diabetes and Nephropathy. New England Journal of Medicine. 2019;380(24):2295-306.

34. Bhatt DL, Szarek M, Pitt B, Cannon CP, Leiter LA, McGuire DK, et al. Sotagliflozin in Patients with Diabetes and Chronic Kidney Disease. N Engl J Med. 2021;384(2):129-39.

35. McMurray JJV, Solomon SD, Inzucchi SE, Køber L, Kosiborod MN, Martinez FA, et al. Dapagliflozin in Patients with Heart Failure and Reduced Ejection Fraction. New England Journal of Medicine. 2019;381(21):1995-2008.

36. Packer M, Anker SD, Butler J, Filippatos G, Pocock SJ, Carson P, et al. Cardiovascular and Renal Outcomes with Empagliflozin in Heart Failure. New England Journal of Medicine. 2020;383(15):1413-24.

37. Anker SD, Butler J, Filippatos G, Ferreira JP, Bocchi E, Böhm M, et al. Empagliflozin in Heart Failure with a Preserved Ejection Fraction. New England Journal of Medicine. 2021;385(16):1451-61.

38. Bhatt DL, Szarek M, Steg PG, Cannon CP, Leiter LA, McGuire DK, et al. Sotagliflozin in Patients with Diabetes and Recent Worsening Heart Failure. New England Journal of Medicine. 2020;384(2):117-28.

39. Dapagliflozin and Effect on Cardiovascular Events in Acute Heart Failure -Thrombolysis in Myocardial Infarction 68 (DAPA ACT HF-TIMI 68): Clinicaltrials.gov; 2020 [Available from: https://clinicaltrials.gov/ct2/show/NCT04363697].

Appendix I: Systematic literature review design and results

Systematic search design

The systematic search was designed using a multi-stage process to maximise sensitivity to small trials (irrespective of recruitment of individuals with kidney disease) and to permit the inclusion of additional populations of interest in future iterations of the clinical guideline. To achieve this, database search queries and inclusion criteria were designed to be broad and sensitive to give a comprehensive summary of the relevant literature.

Searches were designed primarily to identify randomised controlled trials (RCTs) (1). However, where relevant, meta-analyses of trials and pooled analyses of such trials were identified from systematic searches. Stages of systematic search were:

1. Database search and exclusion of non-relevant article types through abstract review
2. Identification of all trials randomising participants to SGLT-2 inhibition by full-text review
3. Identification of specific randomised trials of interest based on pre-defined inclusion criteria by full- text review
4. Identification of relevant meta-analyses from the systematic search
5. Provision of literature to working groups

Trials of interest

Pre-determined trials of interest identified for this guideline map to the guideline sections as follows:

- Large, placebo-controlled RCTs (comprising evidence for sections 2, 3, and 5 of the guideline)
- RCTs conducted in people with type 1 diabetes mellitus (DM) (mapping to section 7a of the guideline)
- RCTs conducted in kidney transplant recipients (mapping to section 7b of the guideline)
- RCTs conducted in people with heart failure with preserved ejection fraction (HFpEF, mapping to section 7c of the guideline)

Trials meeting primary eligibility criteria but not meeting criteria for studies of interest were documented and stored, creating a repository of relevant trials that can be interrogated in future iterations of the guideline for the use of SGLT-2 inhibitors.

Inclusion and exclusion criteria

Inclusion of identified records was mapped to broad inclusion and exclusion criteria as summarised in

Appendix Table 1. Inclusion criteria for studies of interest are detailed in Appendix Table 2.

Risk of bias

Risk of bias of primary studies of interest (comprising large placebo-controlled randomised controlled trials) was assessed using the Cochrane Risk of Bias 2 tool (2). All studies were reviewed by two reviewers (AW, AR, AK) independently and in duplicate.

Appendix table 1: Primary eligibility criteria

Inclusion criteria	Exclusion criteria
• Parallel-group randomised trials	• Phase 1 studies
• Conducted in adult participants	• Pharmacokinetic/pharmacodynamics studies
• Randomising to SGLT-2 inhibition	• Enrolling participants aged <18 years
	• Non-English language studies
	• Trials randomising to SGLT-2 inhibition with no non-SGLT-2 inhibitor comparator

Appendix table 2: Inclusion criteria for specific studies of interest

**Large-placebo controlled randomised trials**
• Randomising participants to SGLT-2 inhibition or Placebo
• Enrolling at least 1000 participants and at least 500 participants to each arm
• Randomising to SGLT-2 inhibition
**Type 1 DM**
• Randomising participants with type 1 DM
**Kidney transplant recipients**
• Randomising kidney transplant recipients
**HFpEF**
• Randomising participants with heart failure
• Reporting ejection fraction for participants by allocation
• Including participants with both heart failure and with ejection fraction >50%

Database search strategy

The Medline and Embase databases were searched on 16th February 2021 via OVID. The database search was designed to identify a) RCTs (identified using validated search filters obtained from the Cochrane Handbook of Systematic Reviews), and b) studies in SGLT-2 inhibition. The full search criteria are detailed in Appendix Tables 3 & 4.

Appendix Table 3: Search strategy for Embase (Via OVID).

	Embase search strategy
1	Randomized controlled trial/
2	Controlled clinical study/
3	random$.ti,ab.
4	randomization/
5	intermethod comparison/
6	placebo.ti,ab.
7	(compare or compared or comparison).ti.
8	((evaluated or evaluate or evaluating or assessed or assess) and (compare or compared or comparing or comparison)).ab.
9	(open adj label).ti,ab.
10	((double or single or doubly or singly) adj (blind or blinded or blindly)).ti,ab.
11	double blind procedure/
12	parallel group$1.ti,ab.
13	(crossover or cross over).ti,ab.
14	((assign$ or match or matched or allocation) adj5 (alternate or group$1 or intervention$1 or patient$1 or subject$1 or participant$1)).ti,ab.
15	(assigned or allocated).ti,ab.
16	(controlled adj7 (study or design or trial)).ti,ab.
17	(volunteer or volunteers).ti,ab.
18	human experiment/
19	trial.ti.
20	1 or 2 or 3 or 4 or 5 or 6 or 7 or 8 or 9 or 10 or 11 or 12 or 13 or 14 or 15 or 16 or 17 or 18 or 19
21	(random$ adj sampl$ adj7 ("cross section$" or questionnaire$1 or survey$ or database$1)).ti,ab. not (comparative study/ or controlled study/ or randomi?ed controlled.ti,ab. or randomly assigned.ti,ab.)
22	Cross-sectional study/ not (randomized controlled trial/ or controlled clinical study/ or controlled study/ or randomi?ed controlled.ti,ab. or control group$1.ti,ab.)
23	(((case adj control$) and random$) not randomi?ed controlled).ti,ab.
24	(Systematic review not (trial or study)).ti.
25	(nonrandom$ not random$).ti,ab.
26	"Random field$".ti,ab.
27	(random cluster adj3 sampl$).ti,ab.
28	(review.ab. and review.pt.) not trial.ti.
29	"we searched".ab. and (review.ti. or review.pt.)
30	"update review".ab.
31	(databases adj4 searched).ab.
32	(rat or rats or mouse or mice or swine or porcine or murine or sheep or lambs or pigs or piglets or rabbit or rabbits or cat or cats or dog or dogs or cattle or bovine or monkey or monkeys or trout or marmoset$1).ti. and animal experiment/
33	Animal experiment/ not (human experiment/ or human/)
34	21 or 22 or 23 or 24 or 25 or 26 or 27 or 28 or 29 or 30 or 31 or 32 or 33
35	20 not 34
36	exp Sodium-Glucose Transporter 2 Inhibitors/

37	sglt2.tw.
38	sglt-2.tw.
39	exp Sodium-Glucose Transporter 2/
40	sodium-glucose transporter$.tw.
41	sodium-glucose co-transporter$.tw.
42	sodium-glucose cotransporter$.tw.
43	(canagliflozin$ or dapagliflozin$ or empagliflozin$ or ertugliflozin$ or ipragliflozin$ or luseogliflozin$ or remogliflozin$ or sergliflozin$ or sotagliflozin$ or tofogliflozin$).tw.
44	36 or 37 or 38 or 39 or 40 or 41 or 42 or 43
45	35 and 44

Searches 1-35 comprise the sensitive Embase RCT filter derived from the Cochrane Handbook of Systematic Reviews of Interventions (1).

Appendix Table 4: Search strategy for Medline (Via OVID)

1	randomized controlled trial.pt.
2	controlled clinical trial.pt.
3	randomized.ab.
4	placebo.ab.
5	clinical trials as topic.sh.
6	randomly.ab.
7	trial.ti.
8	1 or 2 or 3 or 4 or 5 or 6 or 7
9	exp animals/ not humans.sh.
10	8 not 9
11	exp Sodium-Glucose Transporter 2 Inhibitors/
12	sglt2.tw.
13	sglt-2.tw.
14	exp Sodium-Glucose Transporter 2/
15	sodium-glucose transporter$.tw.
16	sodium-glucose co-transporter$.tw.
17	sodium-glucose cotransporter$.tw.
18	(canagliflozin$ or dapagliflozin$ or empagliflozin$ or ertugliflozin$ or ipragliflozin$ or luseogliflozin$ or remogliflozin$ or sergliflozin$ or sotagliflozin$ or tofogliflozin$).tw.
19	11 or 12 or 13 or 14 or 15 or 16 or 17 or 18
20	10 and 19

Searches 1-10 comprise the highly-sensitive Medline RCT filter derived from the Cochrane Handbook of Systematic Reviews of Interventions (1).

Stage 1 – abstract and title screening

Citations including abstracts and relevant record details were downloaded and stored in a dedicated database. Duplicate studies were digitally identified and excluded. Remaining records were screened for relevance using the title and abstract against the primary eligibility criteria (Appendix Table 1) by a single reviewer (AJR). Excluded records were categorised based on the reason for exclusion.

Stages 2 and 3 – full-text identification of relevant trials

Remaining records not excluded through abstract and title screening were exported to an excel spreadsheet to facilitate rapid review by participating reviewers (AJR, WH, SB, AR, AK, AW). The Excel spreadsheet was piloted by all reviewers prior to use. Records were divided between reviewers such that each record was reviewed by two reviewers independently and in duplicate. Records were reviewed for relevance against the primary eligibility criteria (Appendix Table 1). Where studies were included, they were categorised against the inclusion criteria for studies of interest (Appendix Table 2). Any disagreements between reviewers regarding inclusion of a record, or categorisation of records against the inclusion criteria for studies of interest, were resolved by a third reviewer independently (WH).

For all included studies of interest, multiple records of the same trial were reconciled by reference to study acronym, where present, or trial registration database number (e.g. National Clinical Trials [NCT] database number).

Stage 4 – identification of relevant meta-analyses

Meta-analyses identified at the abstract/title screening stage or at full-text review stage were screened for relevance by a single reviewer. Meta-analyses were considered relevant if they provided data on the following domains:

- Chronic kidney disease (CKD)
- Acute kidney injury (AKI)
- Amputation
- Bone fracture
- Diabetic ketoacidosis
- Genital mycotic infections
- Heart failure with preserved ejection fraction
- Transplant
- Type 1 diabetes
- Frailty/multimorbidity

Stage 5 – provision of literature to working groups

Listings of all the relevant publications were distributed to the guideline working groups to inform meeting materials and discussion. From review of the relevant literature, evidence-based guidelines were proposed and agreed upon by consensus discussion.

Stage 6 – Updated literature search following publication of EMPEROR-PRESERVED

The systematic review was updated on to 28th August 2021 following the publication of EMPEROR-PRESERVED in order to identify any new large trial primary or subsidiary publications. The result of the updated literature search is formally documented in Appendix Figure 1’s footnote (and elsewhere (3)). New subsidiary publications were reviewed for relevance by AJR & WH.

2023 UPDATED SEARCH

The database search for the 2023 update was undertaken on 5th September 2022 using the same methodology and search terms as prior iterations of the guideline (as described above). Records identified in the updated search were added to the database and duplicates removed. Newly identified large placebo-

controlled trials (DELIVER and EMPA-KIDNEY) were subject to risk of bias assessment as described above, and were found to be low risk in all domains. A summary of the updated search is listed in Appendix Figure 2. Risk of bias assessment for large placebo-controlled RCTs can be found in Appendix Figure 3.

Appendix figure 1: Summary of systematic search results

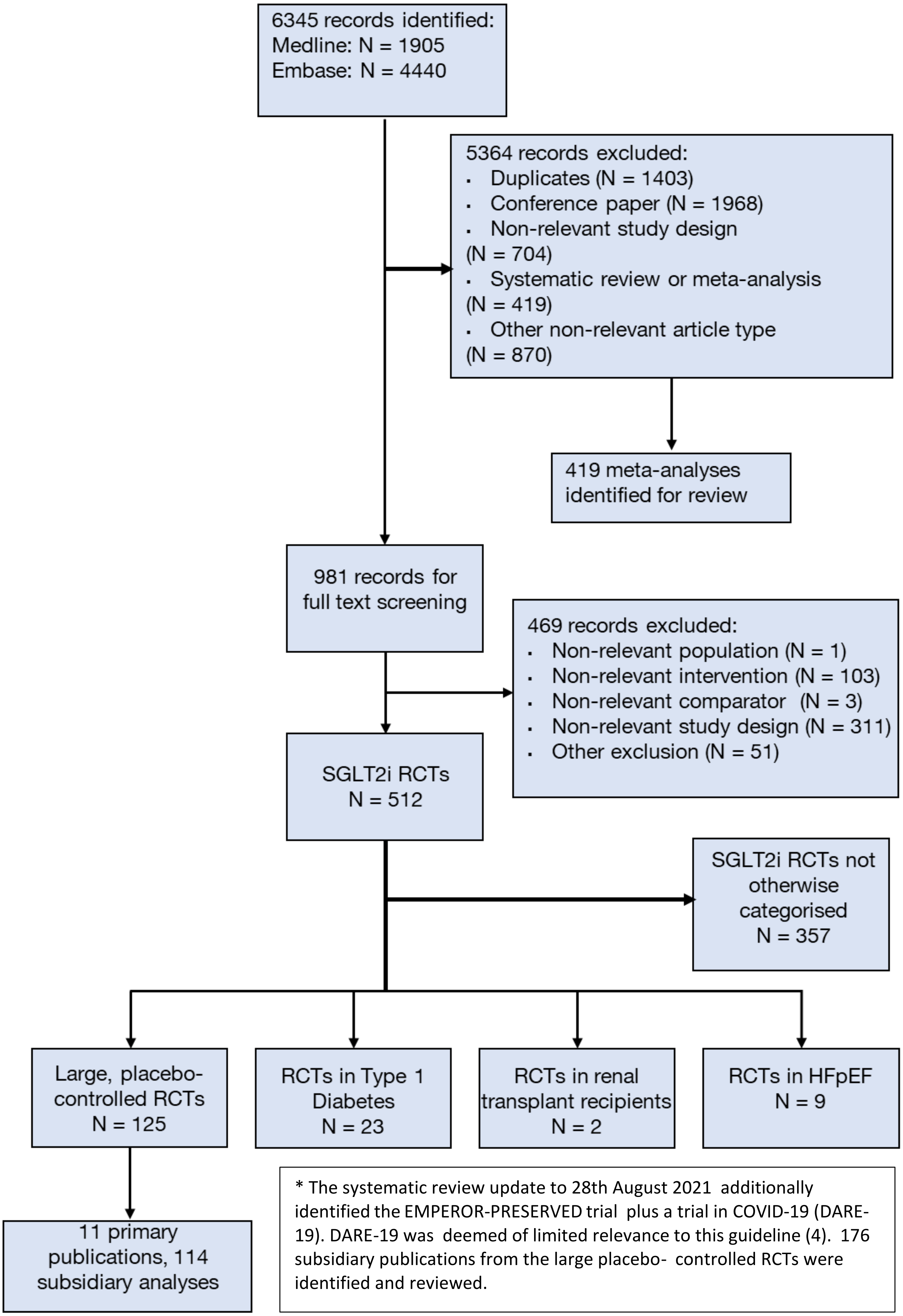

Appendix figure 2: Updated systematic search results

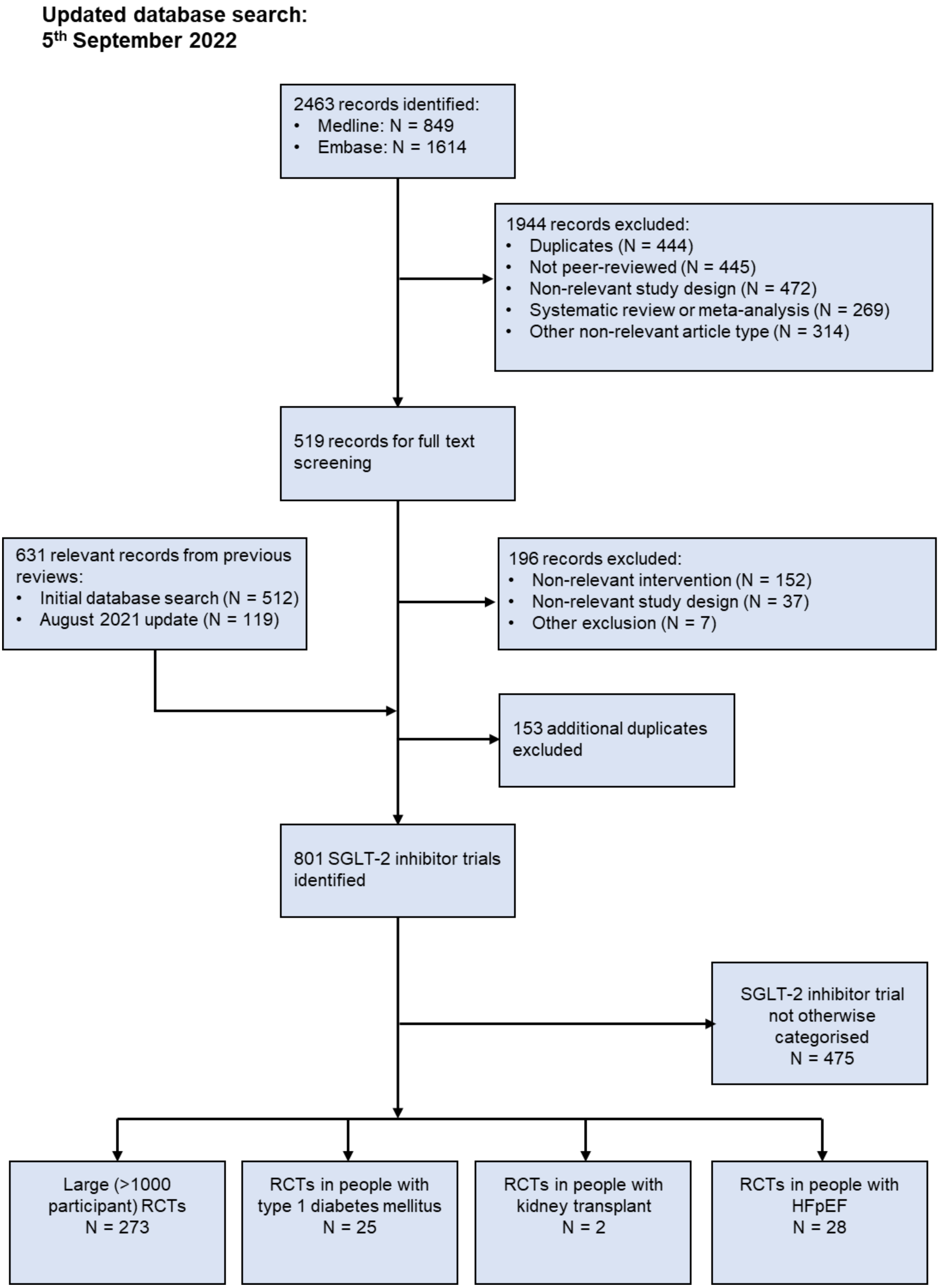

Appendix figure 3: Risk of bias assessment

Study	Intervention	Randomisation Process	Deviations from intended interventions	Missing outcome data	Measurement of the outcome	Selection of the reported result
CANVAS	Canagliflozin	+	+	+	+	+
CREDENCE	Canagliflozin	+	+	+	+	+
DAPA-CKD	Dapagliflozin	+	+	+	+	+
DAPA-HF	Dapagliflozin	+	+	+	+	+
DECLARE-TIMI 58	Dapagliflozin	+	+	+	+	+
DELIVER	Dapagliflozin	+	+	+	+	+
EMPA-KIDNEY	Empagliflozin	+	+	+	+	+
EMPA-REG OUTCOME	Empagliflozin	+	+	+	+	+
EMPEROR-PRESERVED	Empagliflozin	+	+	+	+	+
EMPEROR-REDUCED	Empagliflozin	+	+	+	+	+
InTandem3	Sotagliflozin	+	+	+	+	+
SCORED	Sotagliflozin	+	+	+	+	+
SOLOIST-WHF	Sotagliflozin	+	+	+	+	+
VERTIS CV	Ertugliflozin	+	+	+	+	+

+ = low risk of bias; ! = some concerns. - = high risk of bias.

Appendix I references

1. Higgins JPT, Thomas J, Chandler J, Cumpston M, Li T, Page MJ, Welch VA (editors). Cochrane Handbook for Systematic Reviews of Interventions version 6.3 (updated February 2022). Cochrane, 2022. Available from www.training.cochrane.org/handbook.
2. Sterne JAC, Savović J, Page MJ, Elbers RG, Blencowe NS, Boutron I, et al. RoB 2: a revised tool for assessing risk of bias in randomised trials. BMJ. 2019;366:l4898.
3. Staplin N, Roddick AJ, Emberson J, Reith C, Riding A, Wonnacott A, et al. Net effects of sodium-glucose co-transporter-2 inhibition in different patient groups: a meta-analysis of large placebo-controlled randomized trials. EClinicalMedicine. 2021;41:101163.
4. Kosiborod MN, Esterline R, Furtado RHM, Oscarsson J, Gasparyan SB, Koch GG, et al. Dapagliflozin in patients with cardiometabolic risk factors hospitalised with COVID-19 (DARE-19): a randomised, double-blind, placebo-controlled, phase 3 trial. Lancet Diabetes Endocrinol. 2021;9(9):586-94.

Appendix II: Revision history

Version number	Date	Details
-	21 July 2021	Draft for UKKA Clinical Practice Guideline Committee Review
-	10 August 2021	Draft for Public Consultation
-	21 September 2021	Draft for Clinical Practice Guideline Committee Review (containing revisions following Public Consultation, updates to NICE CKD guidance and publication of EMPEROR-PRESERVED results and the working group's meta-analysis)
1.0	18 October 2021	First released version
-	16 January 2023	Draft 2023 Update for Public Consultation
2.0	TBC	2023 Update released
2.1	1 August 2023	Removal of recommendation relating to children

Appendix III: Working group membership affiliations

Name	Role and affiliations
Andrew Frankel	Nephrologist, Imperial College Healthcare NHS Trust (co-chair)
Will Herrington	Associate Professor at the Medical Research Council Population Health Research Unit at the University of Oxford, Oxford, UK; Honorary Consultant Nephrologist, Oxford Kidney Unit, Oxford (co-chair)
Angela Watt	Patient representative
Michael Watson	Patient representative
John Roberts	Patient representative
David Webb	Academic diabetologist, College of Life Sciences, University of Leicester
Chris Carvalho	General practitioner & CCG clinical lead, London City & Hackney
Patrick Holmes	General practitioner, Darlington, UK Primary Care Diabetes Society
Donald Fraser	Academic nephrologist, Wales Kidney Research Unit, Cardiff University, Cardiff, UK
James Burton	Academic nephrologist, University of Leicester, Leicester, UK
Sunil Bhandari	Nephrologist, Hull University Teaching Hospitals NHS Trust and Hull York Medical School, Hull, UK
Eirini Lioudaki	Nephrologist, Kings College Hospital NHS Trust, London, UK
Mohsen el Kossi	Nephrologist, Doncaster Royal Infirmary, Doncaster, UK
Alex Riding	Nephrologist, Royal Free London NHS Foundation Trust, London, UK
Alexa Wonnacott	Nephrologist, Wales Kidney Research Unit, Cardiff University, Cardiff, UK
Apexa Kuverji	Nephrology trainee, John Walls Renal Unit, Leicester General Hospital, University Hospitals of Leicester, Leicester, UK
Alistair Roddick	Academic Clinical Fellow in Nephrology, University of Oxford, Oxford, UK
Matt Holloway	Renal Pharmacist, East Kent Hospitals University NHS Foundation Trust
Natalie Staplin	Associate Professor and Senior Statistician, Medical Research Council Population Health Research Unit at the University of Oxford, Oxford, UK
Sarah Crimp	Administrative support, UK Kidney Association
